# Leveraging patient derived models of FGFR2 fusion positive intrahepatic cholangiocarcinoma to identify synergistic therapies

**DOI:** 10.1038/s41698-022-00320-5

**Published:** 2022-10-23

**Authors:** Michael E. Lidsky, Zechen Wang, Min Lu, Annie Liu, S. David Hsu, Shannon J. McCall, Zhecheng Sheng, Joshua A. Granek, Kouros Owzar, Karen S. Anderson, Kris C. Wood

**Affiliations:** 1 Department of Surgery, Duke University School of Medicine, Durham, NC USA; 2 Department of Pharmacology, Yale University School of Medicine, New Haven, CT USA; 3 Department of Pharmacology and Cancer Biology, Duke University School of Medicine, Durham, NC USA; 4 Department of Medicine, Duke University School of Medicine, Durham, NC USA; 5 Department of Pathology, Duke University School of Medicine, Durham, NC USA; 6 Department of Bioinformatics, Duke University School of Medicine, Durham, NC USA; 7 Department of Molecular Biophysics and Biochemistry, Yale University School of Medicine, New Haven, CT USA

**Keywords:** Cancer models, Targeted therapies

## Abstract

Intrahepatic cholangiocarcinoma (ICC) remains a deadly malignancy lacking systemic therapies for advanced disease. Recent advancements include selective FGFR1–3 inhibitors for the 15% of ICC patients harboring fusions, although survival is limited by poor response and resistance. Herein we report generation of a patient-derived FGFR2 fusion-positive ICC model system consisting of a cell line, organoid, and xenograft, which have undergone complete histologic, genomic, and phenotypic characterization, including testing standard-of-care systemic therapies. Using these FGFR2 fusion-positive ICC models, we conducted an unbiased high-throughput small molecule screen to prioritize combination strategies with FGFR inhibition, from which HDAC inhibition together with pemigatinib was validated in vitro and in vivo as a synergistic therapy for ICC. Additionally, we demonstrate broad utility of the FGFR/HDAC combination for other FGFR fusion-positive solid tumors. These data are directly translatable and justify early phase trials to establish dosing, safety, and therapeutic efficacy of this synergistic combination.

## Introduction

Intrahepatic cholangiocarcinoma (ICC) is a highly aggressive primary liver cancer that is nearly universally fatal^1,2^. Alarmingly, there has been a 128% rise in incidence of ICC over the past 40 years in the United States^1,3^ with an associated 10-fold increase in ICC-specific mortality^4^, potentially linked to concomitant increases in diabetes, obesity, and non-alcoholic fatty liver disease^5,6^. Most ICC patients present with advanced disease for which broadly effective systemic therapies are sorely lacking and prognosis remains exceedingly poor. Patients with locally unresectable or metastatic ICC are left with first-line cytotoxic therapy consisting of gemcitabine and cisplatin as their primary modality of treatment, which results in a median progression-free survival (PFS) of 8 months and median overall survival (OS) of under 1 year^2^.

The significant molecular and genetic heterogeneity of ICC presents a challenge to discovery of efficacious therapy^7–11^. In light of this, recent efforts toward novel ICC therapies have relied upon advances in tumor sequencing^12,13^. Through these impressive efforts, actionable driver mutations are detected in up to 40% of ICC, including 15% with oncogenic translocations in *Fibroblast Growth Factor Receptor 2* (*FGFR2*) that promote proliferation, cell migration, and survival^14^. Unfortunately, therapies targeting FGFR2 have shown only modest improvements in median PFS of approximately 6–7 months and a median OS under 2 years^15,16^. These data are inclusive of the orally bio-available selective FGFR1–3 inhibitor, pemigatinib, which was the first targeted therapy approved by the Food and Drug Administration in 2020 for the treatment of ICC after progression on gemcitabine-based regimens. Although additional inhibitors have come to the clinic, efficacy remains limited and resistance is inevitable with single-agent FGFR inhibition.

Further impeding advancement in ICC is the lack of reliable, reproducible, and relevant pre-clinical models for the disease. There are very few cell lines of any genomic profile available, many of which are decades old and may not accurately resemble the primary tumor they are thought to recapitulate. Furthermore, none of these established cell lines harbor a FGFR2 fusion, which has impeded pre-clinical advancements for fusion-positive ICC patients. Until recently, the body of literature included important work utilizing engineered cell lines and a single patient-derived ICC cell line with a FGFR2 fusion, lines which were critical to the identification of point mutations as drivers of resistance to targeted therapy^17–19^. In 2022, several new patient-derived models of FGFR2 fusion-positive ICC emerged, though exclusive of organoids, and were used to identify a new combination strategy with EGFR inhibition^20^; given the known crosstalk and interaction between FGFR and EGFR signaling, this combination strategy shows promise and is being explored clinically.

Herein, we report the generation and genomic characterization of patient-derived models of FGFR2 fusion-positive ICC, including a cell line, organoid, and xenograft. We not only demonstrate the clinical relevance of these patient-derived FGFR2 fusion-positive ICC models by testing standard-of-care therapeutic strategies, but we also utilized these resources to identify and validate the synergistic effect of FGFR2 inhibition together with pan-histone deacetylase (HDAC) inhibition, a strategy that warrants further exploration in a clinical trial. Moreover, we show the effectiveness of this FGFR/HDAC combination extends to other FGFR fusion-positive solid tumors.

## Results

### Establishment of patient-derived FGFR2 fusion-positive intrahepatic cholangiocarcinoma models with histologic and immunohistochemical characterization

Representative tumor was obtained from a 50-year-old male who was diagnosed with ICC 7 months prior to tissue acquisition. The tumor, progressing on second-line chemotherapy, was unresectable due to intrahepatic metastases. Next generation sequencing^21^ identified a potentially actionable FGFR2-G3BP2 fusion but due to hepatic dysfunction, the patient was ineligible for a clinical trial. In the absence of effective systemic therapy alternatives, he was considered for hepatic artery infusion, a therapy which facilitates the delivery of high-dose cytotoxic therapy directly to the liver^22^. During this operation, a metastatic lymph node from the porta hepatis was resected, serving as the sample from which these models were derived. The patient ultimately succumbed to his disease nine months after diagnosis.

**Fig. 1:**
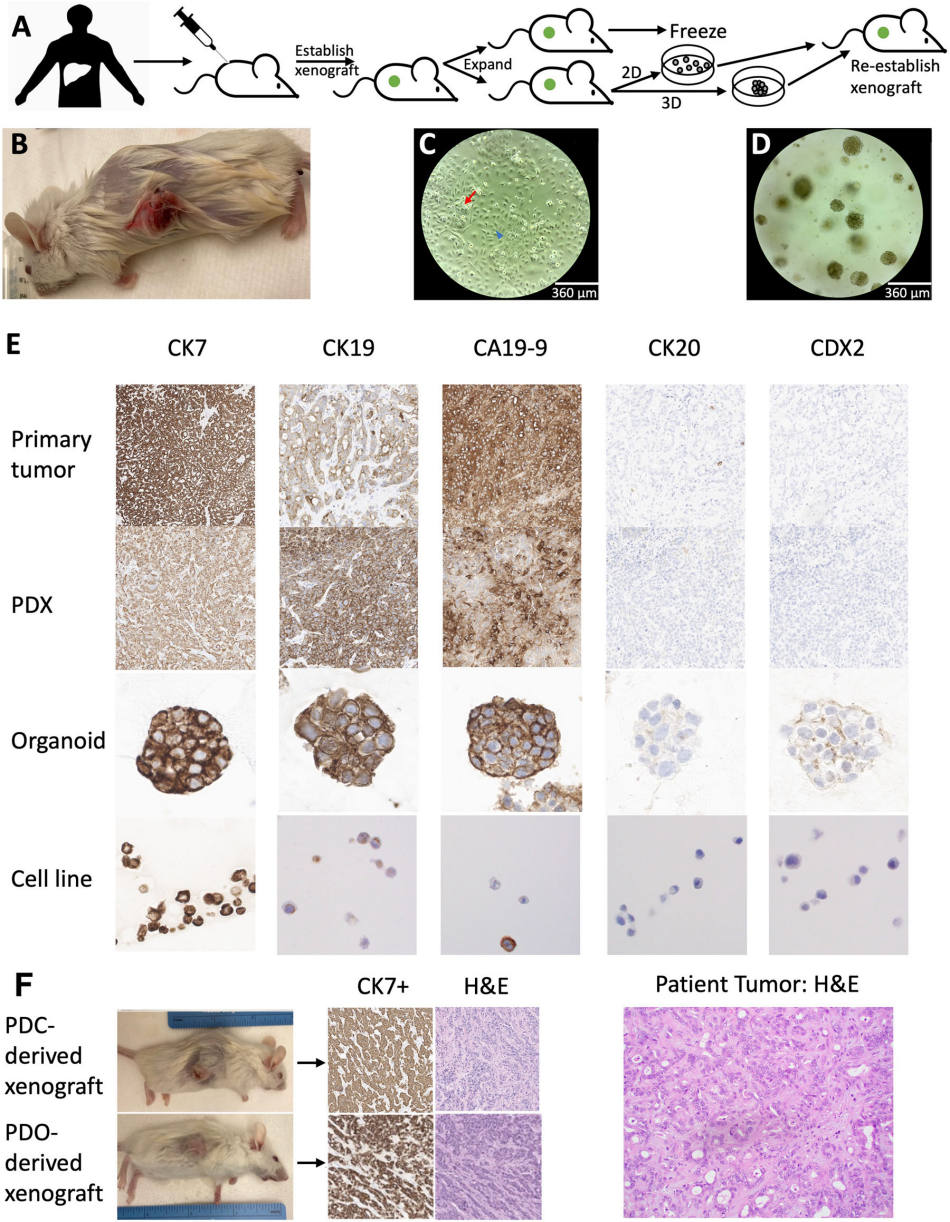
Establishment of DUC18828, patient derived FGFR2 fusion-positive xenograft (PDX), organoid (PDO), and cell line (PDC) models of intrahepatic cholangiocarcinoma (ICC). **A** Schematic of the establishment of the PDX, PDO, and PDC models. Tumor was procured from the operating room to establish the PDX, which was then expanded and/or used to derive the PDO and PDC. Both the PDC and PDO have been re-injected into mice to re-establish xenografts. **B** Representative PDX prior to passage. **C** Brightfield microscopy image of the PDC model showing two distinctive morphologies, one of which is more clustered (red arrow) and the other one is more extended (blue arrowhead). Scale bar, 360 μm. **D** Brightfield microscopy image of the PDO model that possesses a dense, solid sphere structure. Scale bar, 360 μm. **E** Immunohistochemistry (IHC) for positive (CK7, CK19, CA19-9) and negative (CK20, CDX2) markers of ICC confirms the PDX, PDO, and PDC to be consistent with the primary tumor. **F** The PDC and PDO have the ability to regrow as xenografts and re-establish histology and morphology of the patient’s tumor, as evidenced by CK7 positivity and hematoxylin and eosin (H&E) staining, respectively. Morphologically, the patient’s hepatic tumor as well as the PDC- and PDO-derived xenografts are remarkable for moderately differentiated adenocarcinoma, syncytial with areas of definitive gland formation and a prominent stromal response. Cytologically, there is a high nuclear:cytoplasmic ratio, scattered atypical mitotic figures, and significant anisonucleosis.

The patient-derived xenograft (PDX) was generated by injecting minced tumor into the flank of NSG mice (Fig. 1A)^23^. The PDX (Fig. 1B) was monitored for growth, passaged as a second generation to additional mice, and ultimately used to establish a 2-D patient derived cell line (PDC), PDC-DUC18828 (DUC = Duke University Cholangiocarcinoma). The PDC has two distinct morphologies, one of which is more clustered and the other one is more extended (Fig. 1C). The three-dimensinal (3-D) patient-derived organoid (PDO), PDO-DUC18828, was similarly derived from the PDX (Fig. 1D). We examined the histologic appearance of the embedded PDX sample and confirmed epithelioid, gland-forming morphology consistent with the primary tumor (Fig. 1E). We next examined ICC biomarkers by immunohistochemistry (IHC) to ensure fidelity of these patient-derived models. As shown in Fig. 1E, consistent with the primary tumor, the PDX, PDO, and PDC models positively express ICC markers CK7, CK19, and CA19-9 and do not express markers of alternative differentiation (CK20 and CDX2). To confirm the tumorigenic properties of our in vitro cellular and organoid models, both were re-injected into mice to confirm their ability to develop into tumors in vivo (Fig. 1A, F), and the resulting xeno-transplanted tumors recapitulate the same morphology and ICC marker expression pattern as the PDX and patient’s ICC, further supporting the relevance of the patient-derived models.

### Genomic characterization of patient-derived models of ICC confirms retention of FGFR2-G3BP2 fusion

**Fig. 2:**
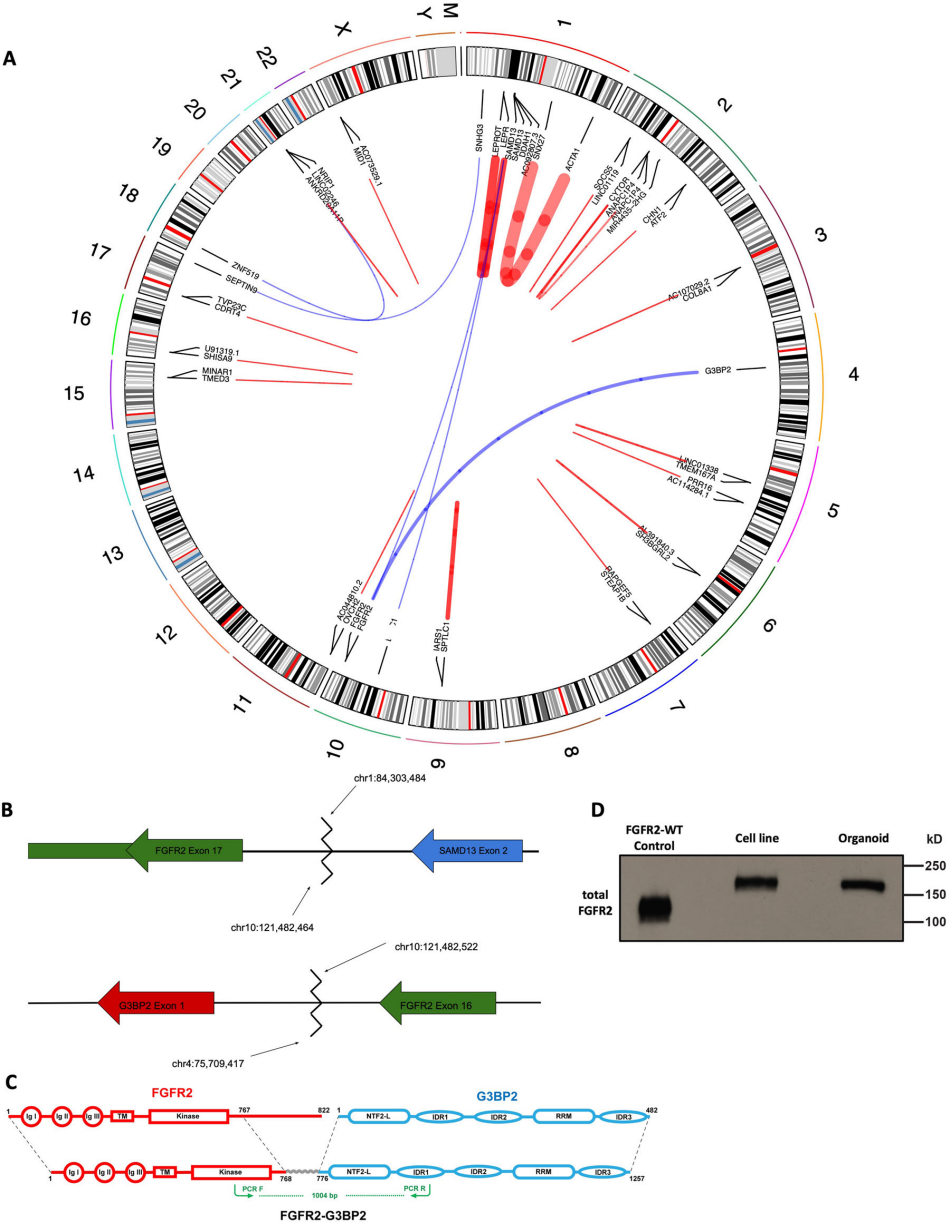
Analysis of a DNA-Sequencing library identified two FGFR2 implicated translocations. **A** Circos plot illustrating complex fusion between chromosomes 1 (SAMD13), 4 (G3BP2), and 10 (FGFR2). Additional fusions are also indicated, and a complete annotation of fusions within this model can be found in the supplementary file. Each arc represents a predicted gene fusion event, where the width of the arc is proportional to the strength of the evidence supporting the prediction. Blue arcs represent fusions between chromosomes and red arcs represent fusions within chromosomes. **B** DNA-Seq identified a complex translocation where chr10:121,482,464 is joined to chr1:84,303,484, placing FGFR2-exon 17 (including the 3’UTR, indicated by a thinner rectangle) upstream of SAMD13-exon 2 and chr4:75,709,417 is joined to chr10:121,482,522, placing FGFR2-exon 16 upstream of G3BP2-exon 1. **C** Schematic of full length FGFR2 (red), full length G3BP2 (blue), and FGFR2-G3BP2 fusion. The 8-amino acids translated from the G3BP2 5’UTR are represented by the gray wavy line. Forward and reverse PCR primers used for RT-PCR are shown in green arrows. Ig I/II/III Immunoglobulin-like domain I/II/III, TM transmembrane domain, NTF2-L nuclear transport factor 2-like domain, IDR 1/2/3 Intrinsically Disordered Domain 1/2/3, RRM, RNA-Recognition Motif. **D** Western blot showing that FGFR2 in the PDC and PDO models has a higher molecular weight compared to wildtype FGFR2 control, supporting the existence of the FGFR2-G3BP2 fusion.

The patient’s tumor was sequenced with the MSK-IMPACT assay^21^ as part of his clinical evaluation, as above, inferring a fusion between *FGFR2* and *G3BP2*. The MSK-IMPACT assay was subsequently used to analyze DUC18828. We identified a complex translocation involving chromosomes 1, 4, and 10, where part of chromosome 10 has been translocated to chromosome 1 and the other part has been translocated to chromosome 4 (Fig. 2A). The breakpoints on chromosome 10 fall within intron 16 of FGFR2 (between exons 16 and 17). The breakpoint on chromosome 4 is upstream of G3BP2. The breakpoint on chromosome 1 is within intron 2 of SAMD13 (Fig. 2B). We validated these breakpoints (chr10:121482464–chr1:84303484 and chr10:121482522–chr4:75709417 using GRCh38/hg38 genome version) by Sanger sequencing PDC genomic DNA. Based on these chromosomal rearrangements, we predicted two different fusion transcripts, one fusing most of FGFR2 to all of G3BP2 and another fusing half of SAMD13 to the 3’ terminal exon of FGFR2.

Ras-GTPase-activating protein-binding protein 2 (G3BP2) is a multi-functional protein with its best-known role in stress granule assembly, RAS signaling, the ubiquitin proteosome pathway, and RNA processing^24^. We validated the predicted FGFR2-G3BP2 fusion transcript by Sanger sequencing of cDNA generated from PDC-DUC18828 (Supplementary Fig. 1A) as well as RNA-Seq of DUC18828. This demonstrated that FGFR2 exon 16 is fused to G3BP2 exon 2 (exon 1 is presumably skipped because it lacks a good splice acceptor site). As a result the FGFR2-G3BP2 fusion transcript is expected to consist of FGFR2 exons 1–16 fused with G3BP2 exons 2–12. The resulting FGFR2-G3BP2 fusion protein is expected to contain the intact extracellular, transmembrane, and kinase domains of FGFR2, with the C-terminal 54 amino acids of FGFR2 replaced by the full length G3BP2 protein (the excluded exon 1 of G3BP2 only contains 5’UTR; Fig. 2C). The fusion transcript includes, in frame, the 24 nucleotides of the 5’UTR portion of G3BP2 exon 2. As a result, we predict that the FGFR2-G2BP2 fusion protein includes 8 amino acids between the FGFR2 and G2BP2 portions, translated from this 5’UTR, which is not normally present in the wildtype G3BP2 protein.

Immunoblotting with the antibody targeting at the N-terminus of FGFR2 found that the FGFR2 species in PDC-DUC18828 and the PDO migrate at a higher molecular weight than FGFR2-wildtype control, consistent with the FGFR2-G3BP2 fusion protein predicted based on DNA and RNA sequencing data (Fig. 2D). Taken together, these results corroborate the identification of FGFR2-G3BP2 fusion in all of our patient-derived models. FGFR2 mutations that result in a loss of the 3’UTR have been observed in other ICC tumors. There is good evidence that the FGFR2 3’UTR plays a regulatory role, indicating that loss of the 3’UTR increases total FGFR2 protein levels^12,13^.

The SAMD13-FGFR2 fusion transcript is predicted to consist of SAMD13 exons 1-2 fused 5’ of the last exon of FGFR2 (exon 17; Fig. 2B). We have not validated the SAMD13-FGFR2 fusion transcript because we do not expect it to play a role in oncogenesis: SAMD13 is not known to be a proto-oncogene and exon 17 of FGFR2 has a small open reading frame that only encodes the 54 amino acids at the C-terminal end of the protein. While these amino acids fall within the protein kinase catalytic domain, only the first five amino acids encoded by this exon are observed in the crystal structure of the FGFR2 protein, suggesting that the rest is unstructured^25^. Further, the fusion results in FGFR2 exon 17 being out of frame with SAMD13 exon 1-2.

The RNA-Seq data also suggests that the complex translocation described above includes breakpoints within the genes *BICC1* and *SAMD13* which results in a fusion transcript consisting of the 5’ end of BICC1 fused to the 3’ end of SAMD13, however, SAMD13 is fused out of frame. BICC1 has been previously reported as intrachromosomal fusion partner of FGFR2 in ICC^26–28^. This predicted BICC1-SAMD13 fusion is tentative because the evidence for it is limited to a total of 24 reads (19 spanning reads and 5 junction reads, none of which have “large anchors” on both sides of the predicted fusion point): it has not been validated by the IMPACT data or targeted Sanger sequencing.

Exon and intron numbering above refers to the following Ensembl transcripts: FGFR2 ENST00000613048.4, SAMD13 ENST00000370673.7, and G3BP2 ENST00000677171.1; these transcripts were arbitrarily selected as reference points, since our data does not uniquely identify which of the annotated transcripts were the source of sequences. Complete lists of SNVs and structural variants inferred from DNA-Sequencing of the PDC and gene fusions inferred from RNA-Sequencing of the PDC are provided in Supplementary File 1.

### Constitutive activation of FGFR2 kinase and sensitivity to FGFR inhibitors

**Fig. 3:**
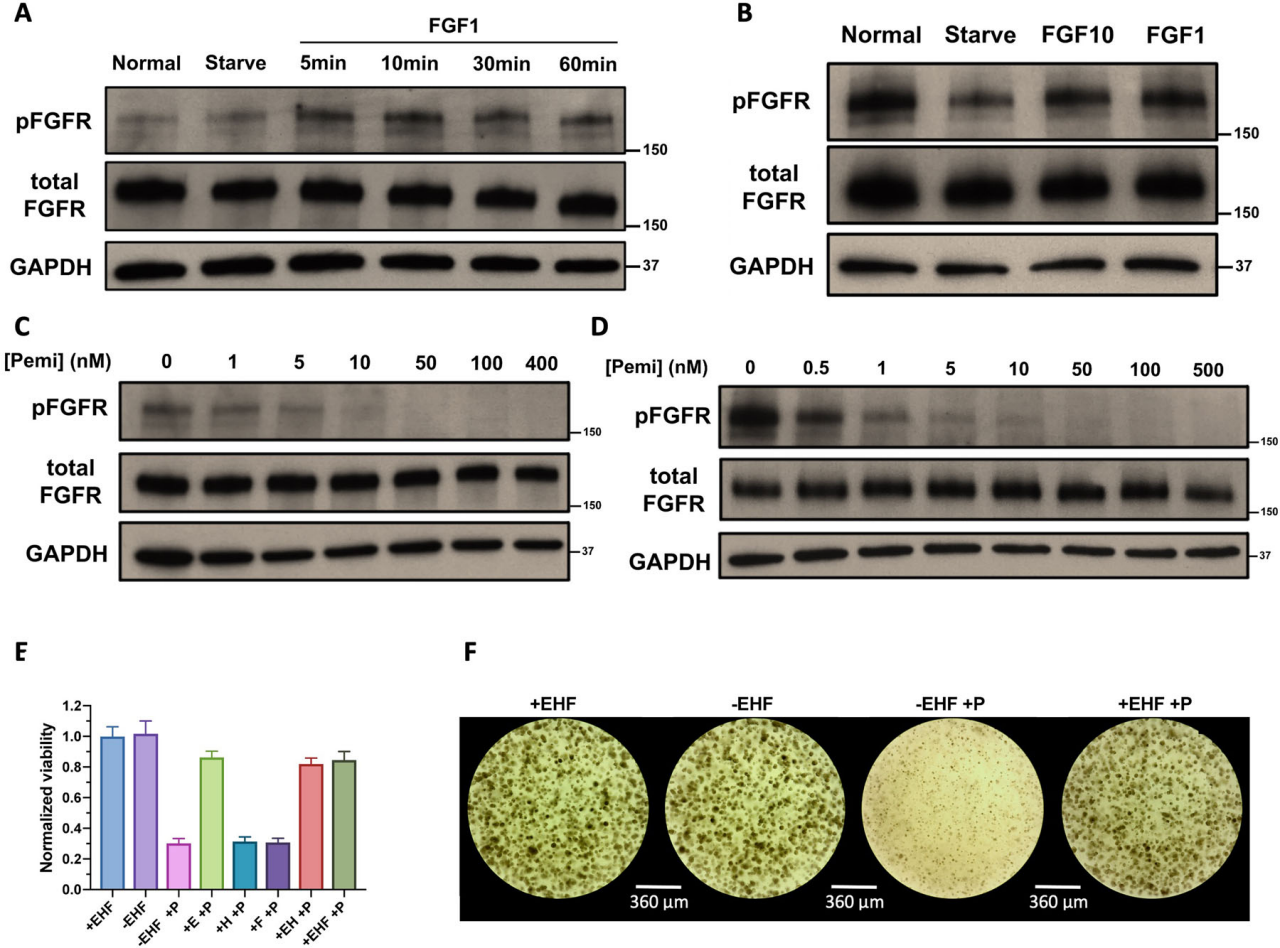
FGFR2-G3BP2 kinase is constitutively active and sensitive to FGFR inhibition. **A** FGFR2 kinase activity is constitutively active in the PDC-DUC18828 model. FGFR kinase activation is examined by western blot under the condition of complete culture media (Normal), serum-free media (Starve), and FGF1 stimulation for 5, 10, 30, and 60 min (FGF1 5, 10, 30, 60 min). FGFR is phosphorylated (activated) to a similar extent in serum-free media when compared to cells grown in complete culture media, confirming constitutive activation, which is further augmented with FGF1 stimulation, **B** FGFR2 kinase activity is constitutively active in the PDO-DUC18828 model. FGFR kinase activation is examined by western blot under the condition of complete culture media (Normal), nutrient-free media (Starve), FGF10 stimulation for 60 min (FGF10), and FGF1 stimulation for 60 min (FGF1). FGFR phosphorylation remains detectable, even in nutrient-free media, confirming constitutive activation, which is further augmented by FGF10 and FGF1 stimulation. **C** FGFR kinase activation is sensitive to pemigatinib (Pemi), an FDA-approved selective FGFR inhibitor, in the PDC-DUC18828 model. FGFR kinase activation is examined by western blot after treatment with pemigatinib (Pemi) doses shown, with significant reduction in activity starting at 10 nM. **D** FGFR kinase activation is sensitive to pemigatinib in the PDO-DUC18828 model. FGFR kinase activation is examined by western blot after treatment with pemigatinib (Pemi) doses shown, with significant reduction in activity starting at 50 nM. **E** Effect of growth factors on organoid viability, determined by Alamarblue after 7 days in culture under different conditions. All results are normalized to the +EHF condition and presented as the mean +/− standard deviation (error bars) for three biologically independent replicates. Note, addition of EHF is able to overcome FGFR inhibition (E, 100 ng/mL hEGF; H, 25 ng/mL hHGF; F, 100 ng/mL hFGF10; P, 50 nM pemigatinib). Of these three factors, hEGF has the greatest impact on rendering the PDO less sensitive to pemigatinib. **F** Brightfield microscopy images of **E** demonstrating organoid growth in the absence of EHF, loss of viability after treatment with pemigatinib in media lacking EHF, but preserved viability despite treatment with pemigatinib when EHF is present. Scale bar, 360 μm.

Most of the FGFR2 fusions reported in ICC patients have at least one dimerization or oligomerization domain in the fusion partner, which can lead to the constitutive activation of FGFR2 kinase activity. Within the FGFR2-G3BP2 fusion in DUC18828, G3BP2 contains a Nuclear Transporter Factor 2-like (NTF2-L) domain that is responsible for the dimerization of G3BP2, suggesting that the kinase domain of FGFR2 may also be constitutively active (Fig. 2C). To confirm constitutive activation of FGFR2 in the PDC and PDO, we measured FGFR2 phosphorylation by western blot in complete culture media (normal), serum-free media (starvation), and serum-free media supplemented with FGF ligands (Fig. 3A, B). As shown in Fig. 3A, removal of FBS does not affect FGFR2 phosphorylation in the PDC and adding FGF1 can further enhance the degree of FGFR2 activation which reaches maximal activation within 5 min after FGF1 stimulation. In the PDO, although FGFR2 phosphorylation decreased with starvation, low-level FGFR2 activation persisted, indicating that FGFR2 is constitutively active. As expected, adding FGF10 or FGF1 in organoid basal media stimulated FGFR2 activation to a similar level as in organoid maintenance media containing FGF10 (Fig. 3B).

We further examined whether FGFR2 activation in DUC18828 models is sensitive to FGFR2 inhibition. Cells were starved for 12 h and then treated with a range of pemigatinib concentrations for 3 h, followed by FGFR2 stimulation with FGF1 for 10 min. Similarly, the organoid was starved for 24 h followed by pemigatinib treatment for 3 h and FGF1 stimulation for 1 h. FGFR2 phosphorylation was impaired by low nanomolar pemigatinib in both the PDC and PDO models (Fig. 3C, D, respectively). Further, FGFR2 phosphorylation can be inhibited below the detection level of western blot by 50–100 nM pemigatinib. These results confirm that FGFR2 signaling is active in these patient-derived models and inhibited by pemigatinib.

### FGFR2 constitutive activation promotes organoid growth and survival independent of growth factors

Growth factors are usually required in organoid culture media for proper growth. To investigate whether the constitutive activation of FGFR2 activity permits growth factor-independent organoid growth, we first cultured the organoid with and without growth factors (EGF, FGF10, and HGF): the organoid grew equally well in the presence and absence of these growth factors (Fig. 3E, F). Further, organoids were treated with 50 nM pemigatinib in growth-factor deficient media which resulted in significant growth inhibition, while adding growth factors back restored viability. These data suggest that constitutive activation of FGFR2 in the PDO is essential for the growth factor-independent organoid viability. We also observed that among all three growth factors, EGF plays a major role in overcoming pemigatinib inhibition (Fig. 3E). Thus, to avoid interference from growth factors, the organoids were cultured in growth factor-free organoid assay media for all subsequent assays.

### Pemigatinib inhibits growth and proliferation of all three patient-derived FGFR2 fusion-positive models

**Fig. 4:**
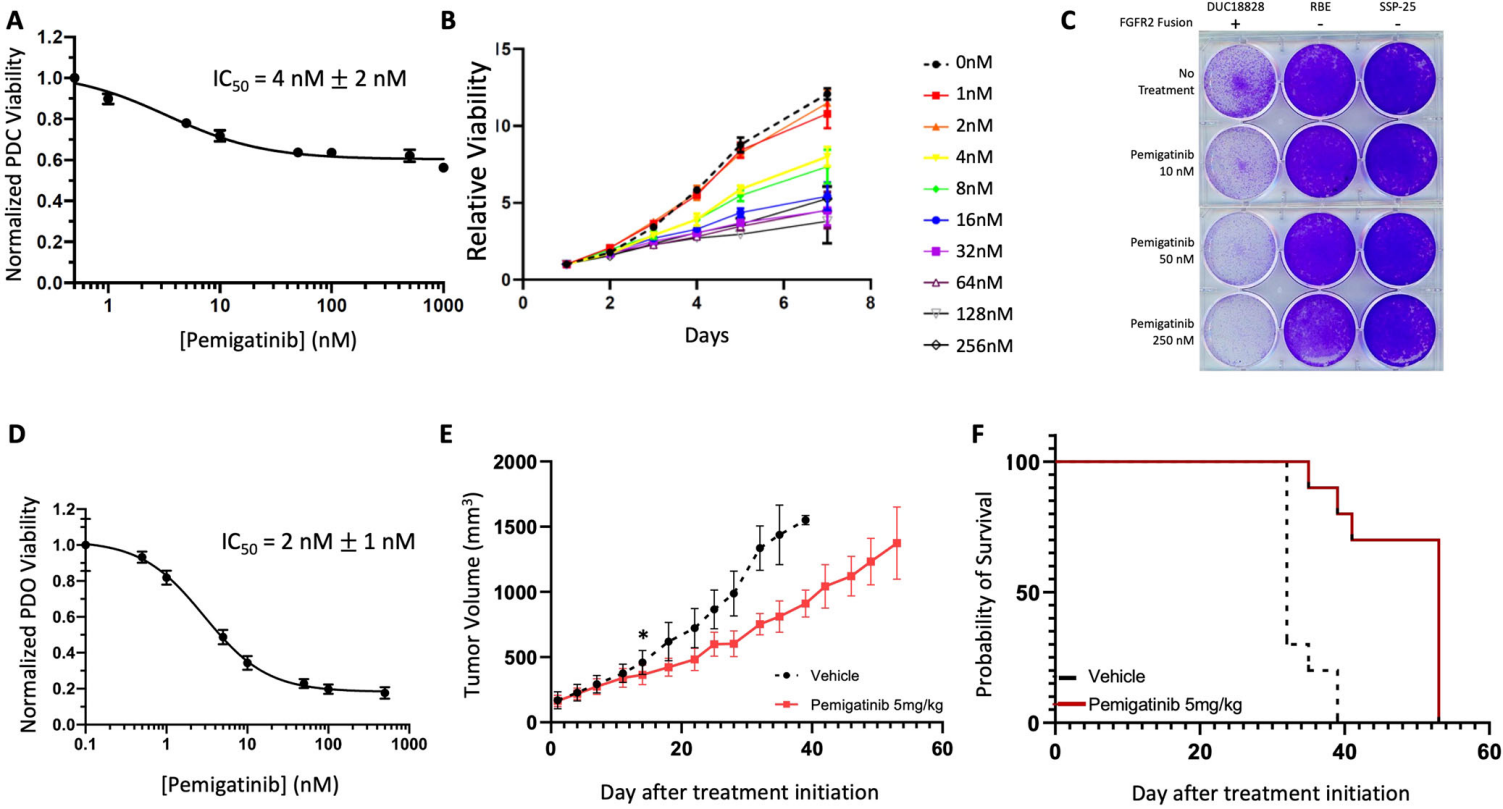
Patient-derived models of FGFR2 fusion-positive ICC are sensitive to FGFR inhibition. **A** GI_50_ assay with pemigatinib in the PDC-DUC18828 model confirmed the IC_50_ value to be 4 nM, determined by Alamarblue after 3 days treatment. **B** PDC-DUC18828 relative viability is impaired by pemigatinib in a dose-dependent manner. Triplicate results are averaged and normalized to DMSO control (0 nM pemigatinib) in this 7 day assay, determined by Cell TiterGlo. **C** Clonogenic assay demonstrating dose-dependent loss of viability in PDC-DUC18828 but not in two FGFR2 wildtype cell lines, RBE and SSP-25. **D** GI_50_ assay with pemigatinib in the PDO-DUC18828 model confirmed the IC_50_ value to be 2 nM. Viability results are normalized to DMSO control and presented as the mean +/− standard deviation (error bars) for three biologically independent replicates. **E** Pemigatinib impairs tumor growth in vivo. Mice bearing subcutaneous xenografts (~125 mm^3^) were randomized to control (sham gavage with Ora-plus suspension) vs pemigatinib (5 mg/kg oral gavage) and were treated 5 days per week. Tumor volume was measured 3×/week until study endpoints were reached. Compared to control, pemigatinib significantly impaired tumor growth, evidenced by the prolonged time to tumor volume endpoint (*p* < 0.05 beginning on day 14 of treatment, indicated by the asterisk (*); data represented as mean +/− standard error of the mean for each experimental condition, consisting of 8 mice each and compared using the Student’s *t* test). **F** Median overall survival of mice treated with pemigatinib (from Fig. 4E), determined by Kaplan–Meier method, is 53 days, compared to 32 days for mice treated with sham gavage (*p* < 0.0001). All in vitro (**A**, **B**, **D**) results are normalized to DMSO control and presented as the mean +/− standard deviation (error bars) for three biologically independent replicates.

After confirming that pemigatinib inhibits FGFR2 activation DUC18828, we subsequently investigated whether growth and proliferation are also impaired by pemigatinib in these patient-derived models. Concordant with FGFR2 signaling inhibition, pemigatinib inhibits cell viability in vitro with a 50% inhibitory concentration (IC_50_) of 4 nM in the PDC (Fig. 4A), whereas the IC_50_ of pemigatinib is >400 nM in SSP25 cells, an established ICC cell line with no FGFR alterations (Supplementary Fig. 2A). Further, pemigatinib causes a dose-dependent loss of viability with significant inhibitory effects on cell proliferation at only 4 nM, and the clonogenic ability of the PDC, compared to RBE and SSP-25 which lack FGFR2 fusions, is markedly suppressed by low nanomolar pemigatinib (Fig. 4B, C). To further verify the translatable relevance of PDC-DUC18828, we assessed sensitivity to FGFR inhibition using another selective FGFR inhibitor, BGJ398 (infigratinib), which exhibited a low nanomolar IC_50_ (~12 nM) and suppresses proliferation at doses as low as 10 nM in vitro (Supplementary Fig. 2B, C).

Consistent with the cell line, the PDO is also sensitive to pemigatinib with an IC_50_ of 2 nM (Fig. 4D), and addition of growth factors back into the organoid assay media results in a >1000-fold shift in the pemigatinib IC_50_ (Supplementary Fig. 2D). Notably, while pemigatinib can suppress cell proliferation at low nanomolar concentrations, Fig. 4A reveals that pemigatinib can only achieve ~30–40% maximal inhibition in the cell line. In contrast, pemigatinib can reach ~80–90% maximal inhibition in the organoid (Fig. 4D). Efforts are ongoing to fully understand the underlying mechanisms of this differential sensitivity to FGFR inhibitors between these two model systems.

To further validate the relevance of these patient-derived models, we investigated the therapeutic response to pemigatinib in vivo. Fig. 4E demonstrates that pemigatinib impairs tumor growth (*p* < 0.05 beginning on treatment day 14), which resulted in a 1.7-fold improvement in median survival (32 vs 53 days, *p* < 0.0001, Fig. 4F).

### Leveraging patient-derived models to test sensitivity to standard-of-care cytotoxic and targeted therapy: Combining gemcitabine and pemigatinib yields additive impairment on viability

**Fig. 5:**
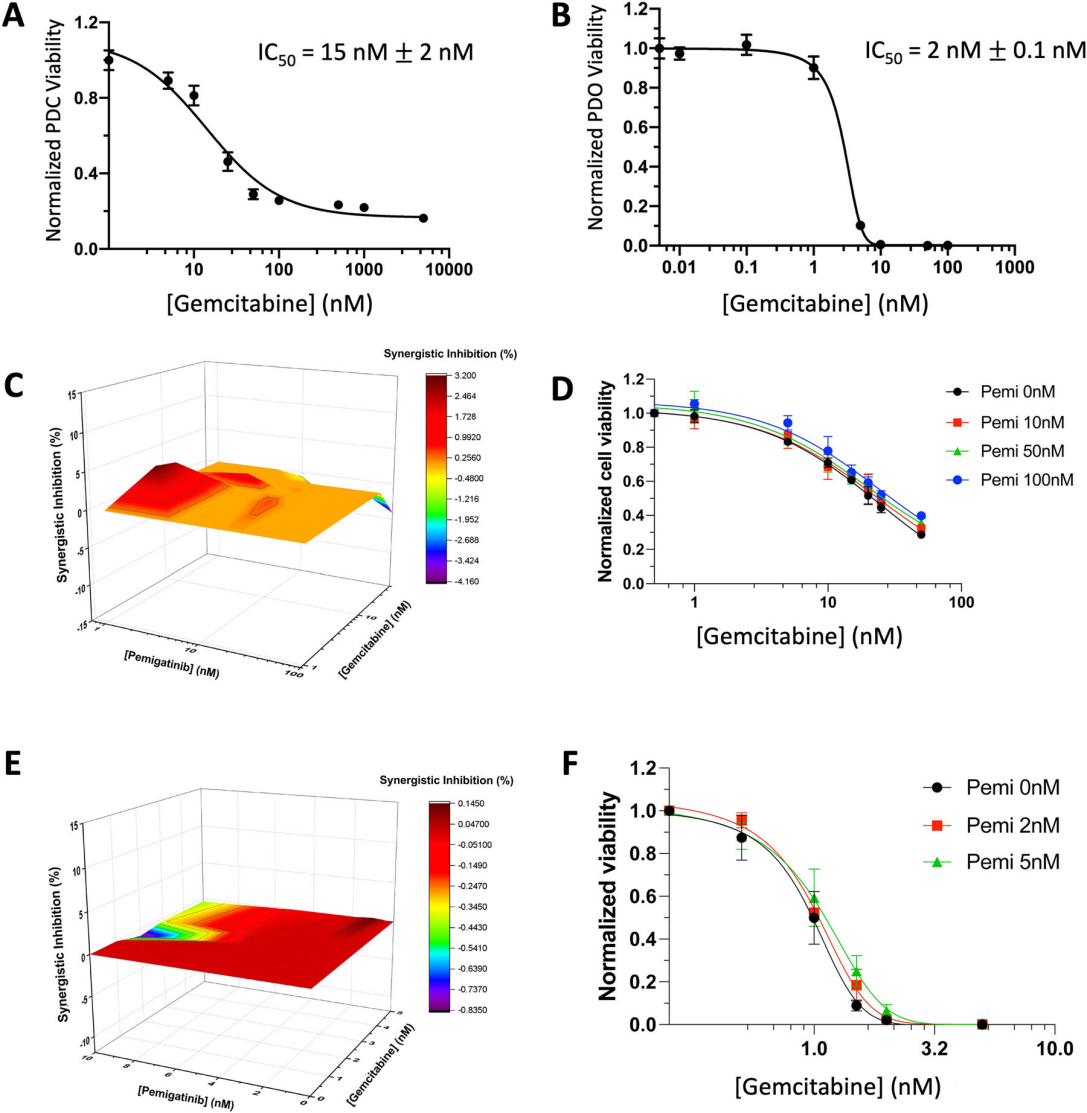
Patient-derived FGFR2 fusion-positive ICC cell line and organoid models are sensitive to gemcitabine, with additive impairment in viability when combined with FGFR inhibition. **A** GI_50_ assay with gemcitabine in the PDC model confirmed the IC_50_ value to be 15 nM. **B** GI_50_ assay with gemcitabine in the PDO model confirmed the IC_50_ value to be 2 nM. **C** Gemcitabine and pemigatinib have additive effects on viability in the PDC model based on MacSynergy II calculation (95% confidence interval). Results are color-coded for range of synergy from purple (least synergistic) to dark red (most synergistic). Data below the 0 plane are antagonistic, in the plane are additive, and above the plane are synergistic. **D** Dose–response curves confirm additive effects of gemcitabine in the PDC model when combined with different concentrations of pemigatinib. All curves are normalized to 0 nM gemcitabine under the corresponding concentrations of pemigatinib. **E** Gemcitabine and pemigatinib have additive effects on viability in the PDO model based on MacSynergy II calculation (95% confidence interval). Data are represented as in **C**. **F** Dose–response curves confirm additive effects of gemcitabine in the PDO model when combined with different concentrations of pemigatinib (normalized as per **D**). Viability results are normalized to DMSO control and presented as the mean +/− standard deviation (error bars) for three biologically independent replicates.

Since 2010, the standard-of-care first-line treatment for advanced ICC has been gemcitabine and cisplatin^2^. Accordingly, we evaluated whether the combination of gemcitabine and pemigatinib yields additional benefit. We first confirmed sensitivity to gemcitabine in DUC18828 PDC and PDO, which exhibit IC_50_ values of 15 and 2 nM, respectively (Fig. 5A, B). Next, we assessed the combined efficacy of pemigatinib and gemcitabine by treating the PDC and PDO with a concentration array of each drug. The 3-D plots and lack of shift on the dose-response curves confirmed additive effects (Fig. 5C–F).

### High-throughput drug screen reveals that HDAC inhibition is synergistic with pemigatinib

**Fig. 6:**
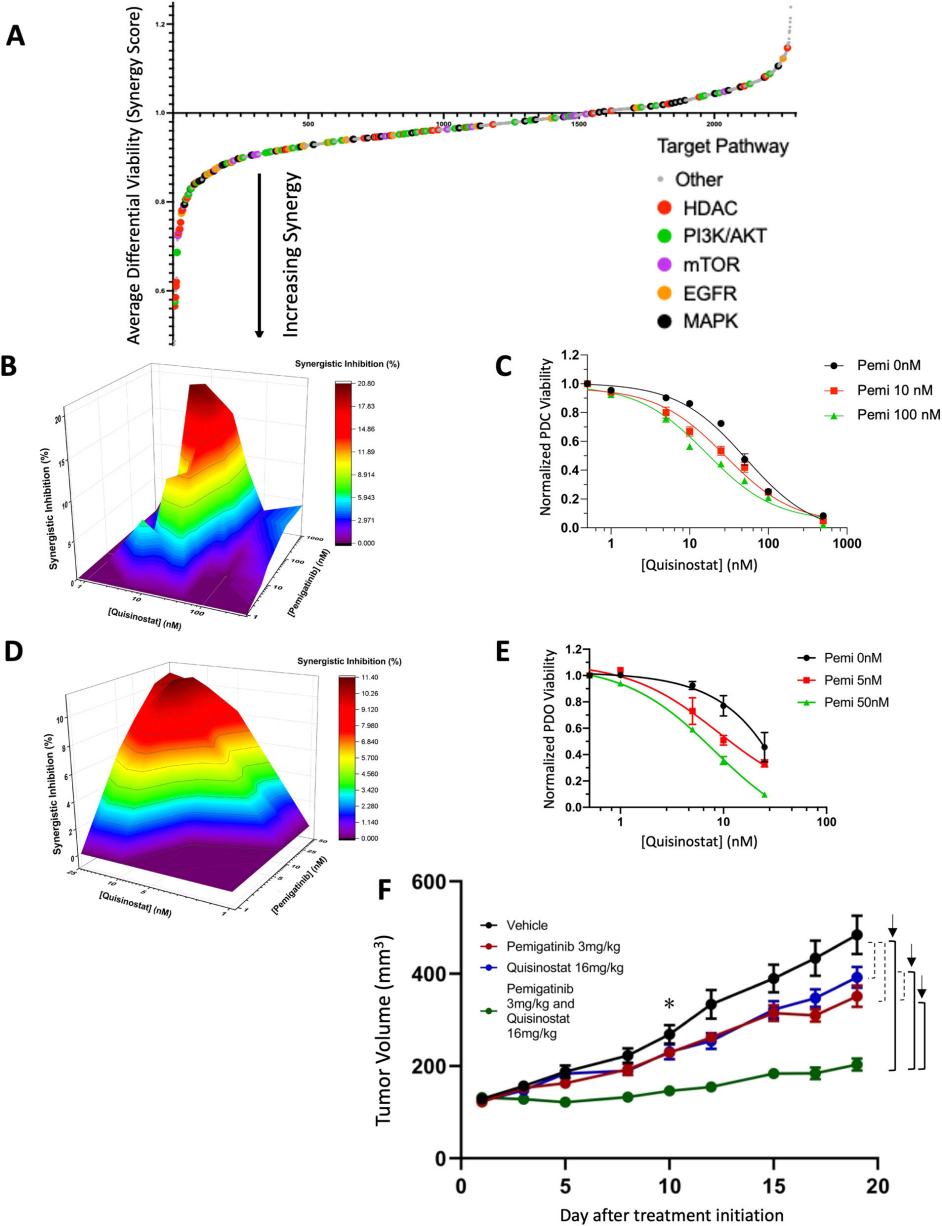
HDAC and FGFR inhibition are synergistic in patient-derived FGFR2 fusion-positive ICC models. **A** High-throughput small molecule screen inclusive of >2100 clinically relevant anticancer drugs suggest targets synergistic with pemigatinib. Each dot represents a compound plotted against that drug’s synergy score on the *y*-axis. Smaller differential viability is synonymous with increased synergy. Targets of commonly synergistic pathways are specifically highlighted. **B** Quisinostat and pemigatinib have synergistic effects on viability in the PDC-DUC18828 model based on MacSynergy II calculation (95% confidence interval). Results are color-coded from purple (most antagonistic) to dark red (most synergistic). **C** Dose–response curves in the PDC-DUC18828 model shift left with increasing concentrations of pemigatinib, resulting in significant reduction in the IC_50_ value of quisinostat, concordant with synergy observed in **B**. All curves are normalized to 0 nM quisinostat under the corresponding concentrations of pemigatinib. **D** Quisinostat and pemigatinib have synergistic effects on viability in the PDO-DUC18828 model based on MacSynergy II calculation (95% confidence interval). Data are represented as in **B**. **E** Dose–response curves in the PDO model shift left with increasing concentrations of pemigatinib, resulting in significant reduction in the IC_50_ value of quisinostat, concordant with synergy observed in **D** (normalized as per **C**). Viability results are normalized to DMSO control and presented as the mean +/− standard deviation (error bars) for three biologically independent replicates. **F** Quisinostat and pemigatinib have synergistic effects on tumor growth in vivo. Mice bearing subcutaneous xenografts (~125 mm^3^) were randomized to control (sham/sham gavage with Ora-plus suspension) vs pemigatinib (3 mg/kg oral gavage + sham gavage with Ora-plus suspension) vs quisinostat (16 mg/kg oral gavage + sham gavage with Ora-plus suspension) vs combination treatment (pemigatinib 3 mg/kg oral gavage + quisinostat 16 mg/kg oral gavage) and were treated 5 days per week. Tumor volume was measured 3×/week until study endpoints were reached. Compared to the control cohort (sham/sham), combined treatment with pemigatinib and quisinostat impaired tumor growth by 59.4%, compared to 23.8% with pemigatinib alone and 15.1% with quisinostat alone. Solid brackets (also recognized by arrows) indicate *p* < 0.05 between treatment arms starting on day 10 of treatment, indicated by the asterisk (*), whereas dashed brackets were not significant. Data are represented as mean +/− standard error of the mean for each experimental condition, consisting of 12 mice each and compared using the Student’s *t* test.

A high throughput small molecule screening effort was carried out with PDC-DUC18828, inclusive of >2100 oncologically relevant compounds^29^. Figure 6A shows a drug rank-order plot of average differential viability (synergy score) with prioritized target pathways highlighted. Drugs specifically targeting HDAC, PI3K/AKT, mTOR, EGFR, and MAPK signaling were selected for further investigation as these were amongst the strongest hits (full dataset can be found in Supplementary Table 1). A comprehensive synergy array experiment was carried out to validate synergy observed in the screen. One of the most promising hits was the HDAC pathway inhibitor, quisinostat, a second-generation broad spectrum inhibitor. As shown in the 3-D plots, quisinostat and pemigatinib are synergistic in the PDC and PDO (Fig. 6B, D, respectively). Synergistic effects were observed with 10–50 nM quisinostat and 10–100 nM pemigatinib in the PDC (Fig. 6B). Dose–response curves in the PDC verify that pemigatinib decreases the IC_50_ of quisinostat by more than 50%, from ~71 to ~31 nM (Fig. 6C). Importantly, similar synergistic effects were also observed in the PDO (Fig. 6D, E). We further confirmed in vitro synergy with pemigatinib and another pan-HDAC inhibitor, panobinostat (Supplementary Fig. 3), suggesting broad applicability of pan-HDAC inhibitors with FGFR targeted therapies in FGFR2 fusion-positive ICC.

The utility of the FGFR/HDAC combination therapy was then evaluated in vivo using PDX-DUC18828. Figure 6F validates the synergistic effect of quisinostat and pemigatinib: compared to the control arm, we observed a reduction in normalized tumor volume by 23.8% with pemigatinib and 15.1% with quisinostat, but 59.4% with combination therapy (p < 0.05 beginning on treatment day 10). Also, it should be noted that the pemigatinib dose for this experiment is lower than that used for the monotherapy experiment above, and the quisinostat dose is lower than the maximum tolerated dose based on previous pre-clinical data^30^. As illustrated in Supplementary Fig. 4, the broad applicability of the synergistic effects of FGFR/HDAC co-inhibition for other FGFR-fusion positive solid tumors was established by examining a urothelial bladder cancer cell line (UBC), RT4, and an estrogen receptor-negative, progesterone receptor-negative, HER2-positive breast cancer cell line, SUM185PE, both of which harbor a FGFR3–TACC3 fusion commonly found in a number of solid tumors^31^.

### Pemigatinib and quisinostat synergistically inhibit proliferation and cause cell cycle arrest and apoptosis

**Fig. 7:**
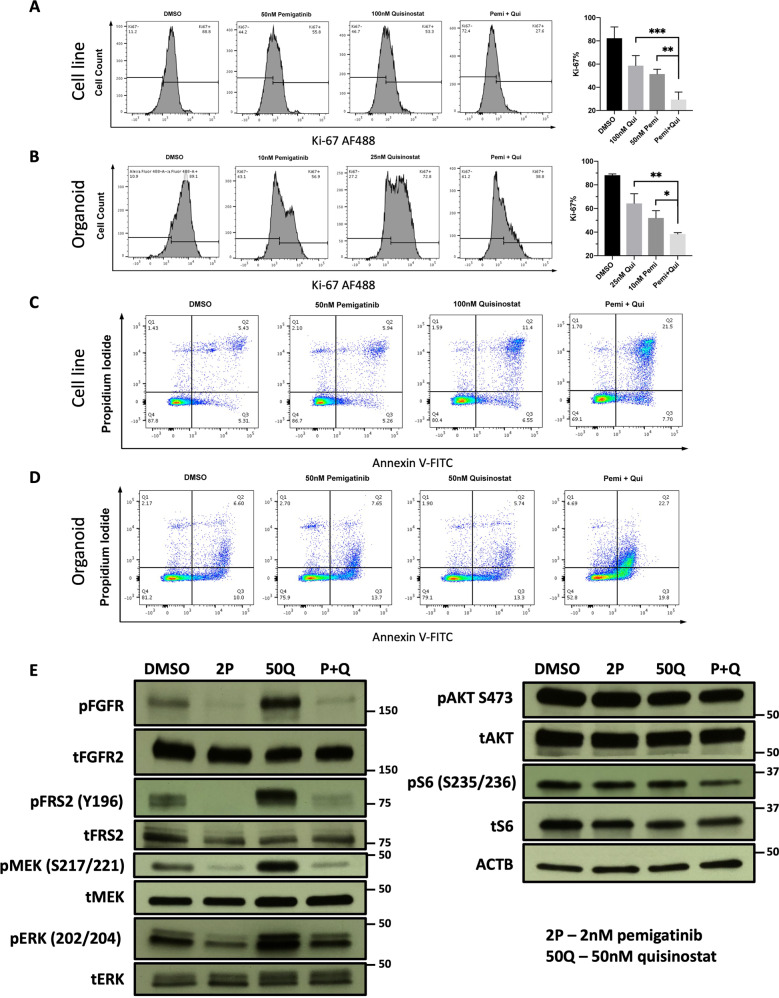
HDAC and FGFR inhibition synergistically inhibit proliferation and enhance apoptosis and impair FGFR-dependent signaling. **A** Quisinostat and pemigatinib synergistically lower the proliferative index (Ki-67) in the PDC-DUC18828 model. The percentage of Ki-67-positive cells was determined and flow cytometry data are shown on the left with corresponding Ki-67 index for each condition shown in the bar graph on the right. Compared to DMSO control, 50 nM pemigatinib decreases the Ki-67 index by ~30%, 100 nM quisinostat decreases the Ki-67 index by ~24%, while 50 nM pemigatinib + 100 nM quisinostat (Pemi + Qui) synergistically decrease the Ki-67 index by ~53% (**p < 0.01 and ***p < 0.001). **B** Quisinostat and pemigatinib synergistically lower the Ki-67 in the PDO-DUC18828 model. Data are presented as in **A**. Compared to DMSO control, 10 nM pemigatinib decreases the Ki-67 by ~36%, 25 nM quisinostat decreases the Ki-67 by ~24%, while 10 nM pemigatinib + 25 nM quisinostat (Pemi + Qui) synergistically decreases the Ki-67 index by ~50% (*p < 0.05 and **p < 0.01). **C**, **D** Quisinostat and pemigatinib synergistically enhance apoptosis in the PDC- and PDO-DUC18828 models, respectively. In both models, under the conditions indicated, combined inhibition of HDAC and FGFR increases the proportion of apoptotic cells, especially late-stage apoptosis, but also the early apoptotic population, compared to monotherapy or DMSO control. Q1, dead cells; Q2, late apoptotic/dead cells; Q3, early-apoptotic cells; Q4, healthy cells. **E** Western blot analysis of PDC-DUC18828 with pemigatinib (2P = 2 nM) monotherapy, quisinostat (50Q = 50 nM) monotherapy, and combination therapy (P + Q = 2 nM pemigatinib + 50 nM quisinostat). Compared to DMSO control, quisinostat monotherapy increases FGFR2, FRS2, MEK, and ERK activation, while pemigatinib monotherapy inhibits FGFR2 signaling, including downstream nodes, FRS2, MEK, and ERK. Combination therapy similarly impairs FGFR2, FRS2, MEK, and ERK activity. Neither monotherapy nor combination therapy alter AKT activity.

The impact of pemigatinib and quisinostat mono- and combination therapy on proliferation and apoptosis was determined using flow cytometry (Fig. 7). For proliferation, either monotherapy can only achieve proliferative inhibition to a moderate extent, reflected by a decrease in the Ki-67-positive population (from ~80% to ~50–60% for the PDC and from ~90% to ~50–60% for the PDO), whereas combining pemigatinib with quisinostat induces significant proliferative inhibition (from ~80% to ~30% for the PDC and from ~90% to ~40% for the PDO) (Fig. 7A, B). Similarly, while pemigatinib and quisinostat monotherapy cause low levels of apoptosis, combination therapy markedly increases the percentage of apoptotic cells, especially the late-stage apoptotic population, both in the PDC and PDO (Fig. 7C, D). These data suggest that pemigatinib and quisinostat achieve synergy by simultaneously inhibiting proliferation and enhancing apoptosis.

### Quisinostat increases FGFR signaling which can be blocked by pemigatinib

To determine the impact of pemigatinib and quisinostat mono- and combination therapy on FGFR signaling, western blot analysis was performed for FGFR2 and downstream proteins in their total and active (phosphorylated) states, under different treatment conditions. As shown in Fig. 7E, quisinostat monotherapy results in an increase in FGFR2, FRS2, MEK, and ERK activation, while pemigatinib monotherapy inhibits FGFR2 signaling, including downstream nodes, FRS2, MEK, and ERK. Combination therapy similarly impairs FGFR2, FRS2, MEK, and ERK activity. Neither monotherapy nor combination therapy alter AKT, suggesting this pathway is not responsible for the impaired viability observed under these treatment conditions. There is slight inhibition of S6 signaling noted with the combination treatment likely through a mechanism distinct from AKT. These data lead us to hypothesize that HDAC inhibition forces cells into a FGFR2 dependent state, such that FGFR inhibition blocks this dependency resulting in the synergistic impairment in viability observed in these models.

## Discussion

Herein, we report and genomically characterize new and unique patient-derived models of FGFR2 fusion-positive ICC. We demonstrate the utility of these models to test therapeutic efficacy of standard regimens, including gemcitabine and FGFR inhibition, as well as to investigate synergistic strategies. This is the first report of a high-throughput small molecule screen performed in a patient-derived model of FGFR2 fusion-positive ICC in the background of the FGFR inhibitor pemigatinib. This screen yielded multiple synergistic hits, including the pan-HDAC inhibitor quisinostat. Synergy between quisinostat and pemigatinib was subsequently validated in DUC18828 cell lines, organoids, and in vivo subcutaneous xenografts. These data support further investigation of FGFR and HDAC inhibition as a strategy for this otherwise deadly disease.

Pre-clinical models for FGFR2 fusion-positive ICC have not been widely reported previously although several non-ICC models engineered to express FGFR fusions and FGFR point mutations have be used to investigate therapeutic resistance^17,18^. Additional contributions to the field include studies suggesting that the PI3K/AKT pathway is upregulated in FGFR inhibitor-resistant non-ICC models, which may be overcome by co-targeting with an mTOR inhibitor^19^. Very recently, several patient-derived models of FGFR2 fusion-positive ICC were reported in elegant work by Wu and colleagues^20^. These new models were used in a small 111 compound drug screen in the background of three different FGFR inhibitors, infigratinib, futibatinib, and rogaratinib, yielding a combination strategy with EGFR inhibition that improved efficacy as well as the ability to rescue resistance to FGFR inhibitors in ICC models^20^. These important contributions comprise the entirety of pre-clinical models of FGFR2 fusion-positive ICC, thus there is a desperate need to develop novel patient-derived models of this disease to optimize the likelihood of translatability in any data generated, and to use these models to discover additional therapeutic strategies with the potential for improved response and durability beyond standard regimens. As such, the models described herein, inclusive of a patient-derived cell line, organoid, and xenograft, genomically, histologically, and morphologically characterized and confirmed to retain a constitutively active FGFR2 fusion. We also demonstrated the dependence of these models on FGFR signaling, which can be impaired pharmacologically, resulting in impaired growth and viability. Further, EGF in the culture media appears to contribute to pemigatinib resistance in the PDO. We can extrapolate from these data that the culture media for the PDC, which contains serum and therefore growth factors, likely contributes to the relative insensitivity to pemigatinib, illustrated by failure to achieve complete cell population death in vitro, despite the PDC having an IC_50_ in the low nanomolar range. As new and important resources for the ICC community, these well-characterized models are especially important for a disease that is relatively uncommon, making advancement in the clinical arena laborious and time consuming. Herein we have demonstrated that the DUC18828 models are clinically relevant and can be leveraged to investigate the intricacies of altered FGFR signaling, as well as to identify and validate novel therapeutic strategies through unbiased approaches to precision pharmacology.

It should be noted that G3BP2 is a somewhat novel fusion partner with FGFR2, identified in only one patient previously^32^, yet its relevance was clearly illustrated in these data. While there are 16 well-characterized fusion partners in FGFR fusion-positive malignancies, recent high-throughput sequencing efforts have detected 37% to be novel^33^. So long as the fusion partner contains a dimerization domain and the breakpoint and fusion are in-frame with FGFR2, resulting in a constitutively active protein, the specific fusion partner appears less relevant. While variation in the fusion partner may impact the degree of tumorigenicity and/or drug response, the requirement for an in-frame fusion was the exact inclusion criteria required in the FIGHT-202 trial, confirming the clinical and biological relevance of the FGFR2-G3BP2 fusion retained in the DUC18828 models^15^.

Due to the ongoing limitations of FGFR inhibition for ICC, specifically inevitable resistance and inadequate PFS outcomes of less than 7 months and OS that fails to reach 2 years, there is a critical need to identify strategies to improve these persistently dismal oncologic outcomes^15,16^. The recent study by Wu and colleagues demonstrated that the EGFR inhibitor, afatinib, in combination with FGFR inhibition with infigratinib, led to significant reduction in tumor growth.

In the current study, leveraging our patient-derived models, we conducted for the first time a high throughput small molecule screen inclusive of over 2100 clinically relevant agents specifically to identify synergistic combinations with pemigatinib, the first FDA-approved FGFR inhibitor. Those drugs with strong synergy scores were prioritized for further analysis, from which quisinostat (synergy score 0.59, top 0.48% of hits), a pan-HDAC inhibitor, was ultimately validated to be synergistic with pemigatinib in vitro and in vivo. We also observed synergistic effects with the EGFR inhibitor, afatinib (synergy score 0.85), however, the synergistic signal was stronger with HDAC inhibition, thereby driving prioritization of that combination for further validation.

Combining HDAC and FGFR inhibition for FGFR2 fusion-positive ICC is a distinct sensitizing and synergistic therapy, which has not been previously reported. HDAC inhibition has been studied in the pre-clinical setting for ICC, where it has been shown to impair epithelial–mesenchymal transition, and therefore migration and invasion^34^. Additional work has suggested HDAC inhibition to improve response to standard cytotoxic therapies in ICC in vitro and in vivo, by way of altered Hippo pathway signaling^35^. These prior reports corroborate our finding, determined through an unbiased approach, of synergy between quisinostat and pemigatinib in FGFR2 fusion-positive ICC models. Of note, we further validated the broad applicability of the FGFR/HDAC inhibitor combination in other FGFR-fusion positive cancers by confirming the synergistic effects in urothelial bladder cancer cells and breast cancer cells containing the FGFR3-TACC3 fusion. Importantly, this combination strategy, which has not been reported for any FGFR fusion-positive malignancy, not only synergistically impaired cell and organoid viability and tumor growth, but also decreased proliferation and resulted in increased apoptosis when compared to either agent alone. These data, in combination with the observed alterations in FGFR signaling, lead us to hypothesize that HDAC inhibition, through a mechanism not yet known, is forcing the cells into a FGFR2 dependent state, such that FGFR inhibition blocks the required pro-survival pathways, thereby resulting in significant impairment in viability. While beyond the scope of the current study, additional investigation is underway to fully characterize the molecular mechanism of synergy and interaction between HDAC and FGFR signaling.

Only one clinical trial (NCT03250273) has investigated HDAC inhibition in ICC. In this study 13 cholangiocarcinoma patients received an HDAC inhibitor together with nivolumab. While the results have not been published, such treatment regimens are well known to be limited by toxicity^36^. To mitigate the high rate of adverse events and severe toxicity related to HDAC inhibition that inevitably result in required treatment holidays, morbidity, and general intolerance, there exists potential to combine this therapy with FGFR inhibition, using both drugs at doses lower than either monotherapy dose required to elicit a response, to yield synergistic efficacy but with an improvement in the toxicity profile. Indeed, the data herein support clinical investigation combining FGFR inhibition with a pan-HDAC inhibitor.

These results highlight the utility of patient-derived models of FGFR2 fusion-positive ICC to investigate standard-of-care anti-cancer treatment and discover therapeutic strategies for this nearly universally deadly disease. By means of unbiased approaches, we demonstrate for the first time for any FGFR2 fusion-positive solid tumor, and specifically for ICC, that combined FGFR and HDAC inhibition is synergistic in these clinically relevant models. Moreover, the effectiveness of this combination may extend to a number of other FGFR fusion-positive solid tumors. These data are directly translatable, and in the context of limited survival outcomes for patients with this disease, an early phase prospective study combining FGFR and HDAC inhibition for patients with FGFR2 fusion-positive ICC is justified.

## Methods

### Acquisition of patient sample, patient-derived xenograft development, and xenograft maintenance

Tumor samples were obtained under a Duke Institutional Review Board (IRB) approved access protocol (Pro00101943) from the Duke BioRepository and Precision Pathology Center, a core resource available to Duke Cancer Institute (DCI) members that obtains broad consent from patients for biospecimen and clinical data collection in support of research (Pro00035974)^37^. This broad consent also includes permission for sharing of de-identified tumor sequencing data. Surgically resected samples undergo standard of care pathological processing and representative samples are procured in a sterile fashion for generation of Patient Derived Xenografts (PDX) under an Institutional Animal Care and Use Committee (IACUC) approved protocol as previously described^37,38^. Patient tumor is washed in phosphate-buffered saline (PBS), minced into 2 mm pieces, and injected into the flank of 8 week old NSG mice (Jackson Laboratories) at 150 mg/mL in PBS. Tumors are measured three times weekly with calipers. Tumor volumes were calculated using the formula, *V* = ((*L*^2^ × *W*))/2 (*L* = longest diameter, *W* = shortest diameter). Once tumors grow to 1000 mm^3^, xenografts are harvested, viably frozen, as well as minced and passaged into additional mice for expansion. The xenograft included herein was assigned the unique identifier DUC18828 (DUC = Duke University Cholangiocarcinoma).

### Establishment of patient-derived cell line and cell culture

Xenograft tissue was harvested, minced into 2 mm pieces, and plated on 10 cm petri dishes. DUC18828 was cultured at 37 °C in 5% CO_2_ and grown in DMEM/F12 with 10% fetal bovine serum (FBS) and 1% penicillin/streptomycin (P/S). Samples were maintained in culture media and passaged until cells were immortalized. Short tandem repeat (STR) analysis was performed by the Duke University DNA Analysis Facility on PDC-DUC18828 to establish a baseline result if future validation is necessary (Chromatography found in Supplementary Fig. 5).

### Establishment of patient-derived organoid and organoid culture

Organoids were established from PDX tissue based on previous description with some modifications^39–41^. Briefly, PDX tissue was collected in organoid basal media (Advanced DMEM/F12, 1× GlutaMax, 10 mM HEPES, 1× Antibiotic-Antimycotic). The tissue was minced and incubated in digestion buffer (DMEM, 2.5% HI-FBS, 0.0125% dispase type II, 0.0125% collagenase type XI, 1× Antibiotic–Antimycotic) for ~2 h at 37 °C. The residual tissue was allowed to settle by gravity for ~5 min, then the supernatant was centrifuged at 1200 rpm and 4 °C for 5 min. The pellet was washed with ice-cold DPBS and then treated with 10 mL ice-cold RBC lysis buffer for 10 min to remove red blood cells, followed by repeat wash and resuspension in matrigel, which was then seeded (50 μL/well) into 24-well plates and solidified at 37 °C for 15 min. Each well was then covered with 500 μL organoid maintenance media (Advanced DMEM/F12, 1× GlutaMax, 10 mM HEPES, 1× Antibiotic–Antimycotic, 1× N2 supplement, 1× B27 supplement, 100 ng/mL human EGF, 1.25 mM *N*-acetylcysteine, 10 nM gastrin, 10 mM nicotinamide, 5 μM A83-01, 10 μM forskolin, 10 μM Y-27632, 10% Rspo-1 condition media, 100 ng/mL human FGF-10, 25 ng/mL human HGF). Medium was changed every 2–3 days.

Organoids were split 1:5-1:10 every 7–10 days after initial establishment. To passage, matrigel was dissolved in Cell Recovery Solution (Corning, #354253) supplied with 10 μM Y-27632 at 4 °C for 30 min. Organoids were then pelleted at 2000 rpm and 4 °C for 10 min, followed by digestion into single cells in 500 μL TrypLE Express at 37 °C for 10 min. The digestion was then diluted in 500 μL ICC maintenance media and centrifuged at 2000 rpm and 4 °C for 10 min. The digested cells were then resuspended in matrigel, re-seeded, solidified, and covered in ICC maintenance media as above.

### Other cell lines

ICC cell lines RBE and SSP-25 were obtained from Lawrence Kwong, PhD, at MD Anderson Cancer Center, and maintained in DMEM with 10% FBS and 1% P/S. RT4 cells were generously gifted by Dr. Darryl Martin’s lab (Yale University, New Haven, CT) and cultured in McCoy’s 5A medium supplied with 10% HI-FBS and 1× antibiotic-antimycotic. SUM185PE (Catalog No: 0003008) was purchased from BioIVT (Wesbury, NY, USA) and cultured in Ham’s F-12 medium supplied with 10% HI-FBS, 10 nM HEPES, 1 μg/mL hydrocortisone, and 5 μg/mL insulin. All cells were cultured at 37 °C in a humidified atmosphere with 5% CO_2_.

### Microscopy and image acquisition

Cells and organoids were imaged by Revolve Fluorescence microscope (Echo), using ×10 objective (Olympus). Slides for IHC were imaged on the Aperio GT450 Slide scanner (×40 objective). Images were viewed using Aperio ImageScope Software (V12.4.3.5008) and acquired with Objective View software (Ontario, Canada).

### Immunohistochemistry

The patient tumor and derived xenograft, organoid, and cell line underwent immunohistochemistry (IHC) for positive (Cytokeratin 7 [CK], CK19, Carbohydrate Antigen 19-9 [CA]) and negative (CK20 and CDX2) markers of cholangiocarcinoma. PDX tissue was fixed in 10% formaldehyde overnight and then paraffin embedded. To embed the organoid, culture media was carefully aspirated. Then the matrigel dome was washed twice with room temperature 1× DPBS for 5 min. The matrigel-embedded organoid was then fixed in 4% formaldehyde at room temperature overnight, dehydrated, and then paraffin embedded. Cell lines were fixed in 10% formaldehyde for 20 min and then paraffin embedded as a pellet, after centrifuging at 2000 rpm ant 4 °C for 5 min. IHC was then performed in the histology core of the pathology department at Duke using the following primary antibodies: monoclonal mouse anti-human antibodies to CK7 (Clone OV-TL 12/30, Cat# M701801-2) and CK19 (Clone RCK108, Cat# IR61561-2) from Dako (Santa Clara, CA), and CK20 (Ks20.8, Cat# PA0022), CA19-9 (C241:5:1:4, Cat# PA0424), and CDX2 (EP25, Cat# PA0375) from Leica Biosystems (Newcastle, United Kingdom). All antibodies were ready-to-use, except CK7 which was diluted 1:100.

### Targeted high-throughput genomic DNA sequencing

Patient tumor was sequenced using the Memorial Sloan Kettering-Integrated Mutation Profiling of Actionable Cancer Targets (MSK-IMPACT) platform^21^. This same platform was used to analyze the DUC18828 cell line. Genomic DNA was extracted using the Qiagen DNA extraction kit (Cat# 13343). DNA was then sent to MSKCC, de-identified, for analysis.

ddPCR was used to determine the percentage of human cells in the PDX sample. Specifically, 9 ng of gDNA from the sample was combined with primers specific to *Mus musculus* and *Homo sapiens* PTGER2, FAM- and HEX-labeled probes, MseI, and digital PCR Supermix for probes (no dUTP). The reaction was performed on a QX200 ddPCR system (Bio-Rad cat# 1864001) and partitioned into ~16,000 droplets using the QX200 droplet generator. The emulsified PCR was run on a 96-well thermal cycler (95 °C 10’; 40 cycles of 94 °C 30’ and 60 °C 1’; 98 °C 10’; 4 °C hold). The plate was read and analyzed with the QuantaSoft software to assess the number of droplets positive for human or mouse. Human percentage was calculated at 99.6%.

After PicoGreen quantification, 100 ng of DNA were used to prepare libraries using the KAPA Hyper Prep Kit (Kapa Biosystems KK8504) with 8 cycles of PCR. 110–160 ng of each barcoded library were captured by hybridization in a pool of 6 samples using the IMPACT assay (Nimblegen SeqCap), designed to capture all protein-coding exons and select introns of 505 commonly implicated oncogenes, tumor suppressor genes, and members of pathways deemed actionable by targeted therapies. Captured pools were sequenced on a HiSeq 4000 in a PE100 run using the HiSeq 3000/4000 SBS Kit (Illumina) producing an average of 502× coverage per sample.

### High-throughput RNA sequencing

PDC-DUC18828 (1 × 10^6^ cells) cultures, in triplicate, were dissociated and resuspended in cell media, centrifuged and washed in PBS, and pelleted by centrifugation. RNA was extracted using Qiagen RNeasy Kits (Cat #74106) according to the manufacturer’s protocol. Sequencing libraries were prepared from purified RNA using the Kapa Stranded mRNA-seq kit (Roche, Cat #KR0960 – v6.17). The three libraries were pooled and sequenced on a single lane of a NovaSeq 6000 SP flow cell, to generate 50 bp paired-end reads. Library preparation and sequencing were performed by The Sequencing and Genomic Technologies Core, part of the Duke University School of Medicine.

### PCR and Sanger sequencing

FGFR2-G3BP2 and SAMD13-FGFR2 genomic breakpoints were validated by PCR and Sanger sequencing. PDC-DUC18828 (1 × 10^6^ cells) were dissociated and resuspended in cell media, centrifuged and washed in PBS, and then pelleted by centrifugation. Genomic DNA was then extracted using Qiagen Blood and Cell Culture DNA Kits (Cat #13323) according to the manufacturer’s protocol. PCR was performed on purified DNA using GoTaq (Promega) PCR master mix using primer pairs FGFR2_SAMD13_F/FGFR2_SAMD13_R and FGFR2_G3BP2_F/FGFR2_G3BP2_R at an annealing temperature of 55 °C. Crude PCR fragments were purified using Ampure XP beads (Beckman Coulter) according to the manufacturer’s protocol. Purified PCR fragment was subjected to Sanger DNA sequencing with the same forward and reverse primers used for PCR. DNA sequencing was performed using BigDye v1.1 and electrophoresed on an ABI 3730xl instrument using POP7 according to the manufacturer’s protocol. PCR and Sanger DNA sequencing was performed at the Duke University DNA Analysis Facility, part of the Duke University School of Medicine. Primer sequences are FGFR2_SAMD13_F: TGAACCGTTCCTTTCCTTTCAAC and FGFR2_SAMD13_R: AGGAAAATGGCTCTGTCGGT (expected product 402 bp); FGFR2_G3BP2_F: AACAAAACAACCAGCCAGGC and FGFR2_G3BP2_R: TCGTCTGTCACTCACTGTGC (expected product 542 bp).

### RT-PCR and Sanger sequencing

RT-PCR and Sanger sequencing were used to validate the FGFR2-G3BP2 fusion transcript. The PDC was harvested with 0.25% Trypsin and pelleted at 1200 rpm for 5 min at room temperature. To harvest the PDO, 50 μL Matrigel was first disrupted in 1 mL ice-cold 1× DPBS and transferred to a 15 mL tube pre-filled with 10 mL ice-cold 1× DPBS, and the organoid was pelleted at 2000 rpm for 5 min at 4 °C.

RNA was extracted from both PDC and PDO samples using RNeasy mini kit (QIAGEN, #74004) following the manufacturer’s protocol, and cDNA was synthesized by SuperScript IV first-strand synthesis system (Invitrogen, #18091050) according to the protocol. PCR was performed by Platinum SuperFi II DNA polymerase (Invitrogen, #12361010) on a 96-well thermal cycler (98 °C 30 s; 40 cycles of 98 °C 10 s, 60 °C for 10 s, and 72 °C for 1’, followed by final extension at 72 °C 5’. The PCR products were run on 0.8% agarose gel stained by GelRed Nucleic Acid Gel Stain (Biotium, #41003). The following primers are used: FGFR2 Forward: 5’-AGCAGACTTTGGACTCGCC-3’ and G3BP2 Reverse: 5’-ATTCTGGCTCAGGTTCAGGTT-3’. PCR products were then cut from the gel and extracted by QIAquick Gel Extraction Kit (QIAGEN, #28704) following the manufacturer’s protocol. Purified PCR products were subjected to Sanger sequencing by the Keck DNA Sequencing Facility at Yale.

### Western blotting and antibodies

The cell line was first seeded and cultured until ~70–80% confluency, followed by starvation in serum-free media for 18 h followed by treatment with inhibitor or DMSO control for 3 h. FGFR kinase activity was then stimulated with 50 ng/mL FGF-1 and 100ug/mL heparin, at which time cells were lysed and prepared for immunoblotting. Similarly, organoids were embedded in 50 μL/well matrigel in 24-well plates and cultured in ICC maintenance media for 6 days. Subsequently, organoids were starved in organoid basal media for 24 h and treated with inhibitor or DMSO control for 3 h. FGFR2 kinase activity was then stimulated by 50 ng/mL FGF-1 or 100 ng/mL FGF-10 with 100 μg/mL heparin. After stimulation, matrigel was disrupted in 1 mL ice-cold 1× DPBS supplied with 1 mM sodium orthovanadate and 25 mM sodium fluoride and transferred to a 15 mL tube pre-filled with 10 mL ice-cold 1× DPBS with 1 mM sodium orthovanadate and 25 mM sodium fluoride, and the organoid was pelleted at 2000 rpm at 4 °C for 5 min.

Cells and organoids were lysed at 4 °C for 30 min by 100 μL/well RIPA lysis buffer (Millipore, #20–188), supplied with 0.1% SDS, protease inhibitor cocktail (Roche, #11836153001), 1 mM sodium orthovanadate, 2 mM beta-glycerophosphate, 25 mM sodium fluoride, 1 mM sodium pyrophosphate. Then the cell lysate was cleared at 15,000 rpm at 4 °C for 10 min.

The impact of HDAC and FGFR inhibition on FGFR2 signaling was assessed in PDC-DUC11828: cells were first treated by indicated concentration of quisinostat or DMSO for 2 days, followed by the treatment of pemigatinib as described above. Cells were then lysed and prepared for immunoblotting.

Protein lysate concentration was determined by BCA assay (Thermo Fisher, #23225), and then mixed with 4× Laemmli Sample buffer and boiled at 95 °C for 10 min. Ten micrograms of protein lysate was separated by 4–20% SDS-PAGE gel and transferred onto nitrocellulose membrane. The membrane was blocked by 5% non-fat milk in 1× TBST at room temperature for 1 h and then immunoblotted by the following antibodies (All antibodies are from Cell Signaling Technology, CST, unless indicated otherwise) at 4 °C overnight. Membranes were probed with primary antibody recognizing phospho-FGFR (Cat# 3476), FGFR2 (Cat# 23328), GAPDH (Cat# 3683), phospho-FRS2 Y196 (Cat# 3864), FRS2 (R&D, Cat# MAB4069), phospho-MEK S217/221 (Cat# 9154), MEK (Cat# 4694), phospho-ERK T202/Y204 (Cat# 4370), ERK (Cat# 4695), phospho-AKT S473 (Cat# 4060), AKT(Cat# 4691), phospho-S6 S235/236 (Cat# 4858), S6 (Cat# 2317) at 1:1000 dilution and GAPDH at 1:1000 at 4 °C overnight.

After that, membranes were washed three times by 1× TBST and incubated with anti-mouse (CST, Cat# 7076) or anti-rabbit HRP-conjugated secondary antibodies (CST, Cat# 7074) at room temperature for 1 h. The immunoblots were visualized by SuperSignal™ West Femto Maximum Sensitivity Substrate (Thermo Fisher Scientific, Cat# 34095) and exposed to X-ray film. Unprocessed blots are included as a supplementary file.

### GI50 and cell viability assays

Cells in DMEM were plated in 96-well plates at a cell density of 5000 cells/well. After 24 h, cells were treated with a serial dilution (GI50: pemigatinib/BGJ398 3.215-1600 nM, gemcitabine 0.32-5000 nM; Cell viability assay: 1–256 nM for all drugs) of indicated drug in DMSO or DMSO control. CellTiter Glo luminescent viability assay (Promega) was used to measure viability after 72 h of treatment for the GI50 assays and daily for 7 days for the viability assays. Relative viability was determined by averaging triplicate results and normalizing to the DMSO control, analyzed using the GraphPad/Prism software (San Diego, CA). GI50 was calculated by fitting each experiment into a 4-parameter logistic curve, identifying the dose at which relative cell viability equals 50% between DMSO control and the highest drug concentration. Viability data was similarly normalized to DMSO control and plotted against time.

### Cell line proliferation assay

After reaching ~70–80% confluence, cells were starved in serum-free media for ~24 h. Then 1 × 10^5^ cells/well were seeded in 6-well plates with desired concentrations of pemigatinib or DMSO control. Cells were trypsinized and mixed with 0.4% Trypan Blue. The number of living cells was counted by Countess II automated cell counter (Invitrogen).

### Organoid growth assay

Organoids were harvested and digested as described above. Then 10,000 cells/well in 10 μL Matrigel were seeded into 48-well plates. Organoids were cultured in 150 μL/well organoid assay media with different combinations of growth factors for 7 days, replenished on Day 4. Organoid viability was then determined by alamarBlue Cell Viability Reagent (Invitrogen) according to the manufacturer’s protocol.

### Clonogenic assay

Clonogenic assays were conducted as previously described^42^ by seeding 40,000 cells in 6-well plates in DMEM. After 24 h, cells were treated with pemigatinib 10, 50, or 250 nM, and DMSO control. When control wells reached near-confluence, 500 μL per well of colony fixation staining solution (Methanol 80%, crystal violet 0.5% and H_2_0) was added, incubated for 20 min at room temperature, washed with tap water and then allowed to dry. Plates were then digitally imaged.

### Drugs

Pemigatinib (Cat# T12401), BGJ398 (infigratinib, Cat# T1975) and quisinostat (Cat# T6055) were purchased from TargetMol Chemicals (Boston, MA). Gemcitabine (Cat# LY-188011), panobinostat (Cat# LBH589), T6061 (LMK-235, Cat# S7569), trichostatin A (Cat# S1045), PF-04691502 (Cat# S2743), GDC-0032 (Taselisib, Cat# S7103), ciclopriox olamine (Cat# S3019), S-Ruxolitinib (Cat# A2902), SP2509 (Cat# S7680), dinaciclib (Cat# S2768), and Trametinib (Cat# S2673) were purchased from Selleck Chemicals (Houston, TX).

### Small molecule screen

The Selleck Bioactive Compound Library was used for the high-throughput small molecule screen, in conjunction with the Duke Functional Genomics Shared Resource, as previously described^29^. Pilot experiments were conducted to determine optimal cell plating density (500 cells/well) and pemigatinib concentration (1 nM). The screen was performed in triplicate with 1 μM library compound in the absence or presence of 1 nM pemigatinib. Cells were incubated for 72 h and then cell viability was assessed with CellTiter Glo (CTG) Luminescent Viability Assay (Promega). Percent killing was quantified by normalizing to DMSO controls on each plate.

### Drug sensitivities and calculation of synergism in vitro

After reaching ~70–80% confluence, cells were starved in serum-free media for ~24 h. Then 10,000 cells/well were seeded in 96-well black wall/clear bottom plates with different concentrations of the inhibitors or DMSO control. Cells were treated for ~72 h and the cell viabilities were determined by alamarBlue Cell Viability Reagent (Invitrogen, #DAL1025) according to the manufacturer’s protocol.

Organoids were harvested and digested as described above. Then 10–20,000 cells/well in 10 μL Matrigel were seeded into 48-well plates. Organoids were cultured in 150 μL/well organoid assay media (Advanced DMEM/F12, 1× GlutaMax, 10 mM HEPES, 1× Antibiotic–Antimycotic, 1× N2 supplement, 1× B27 supplement, 1.25 mM *N*-acetylcysteine, 10 nM gastrin, 10 mM nicotinamide, 5 μM A83-01, 10 μM forskolin, 10 μM Y-27632, 10% Rspo-1 condition media) with different concentrations of the inhibitors or DMSO control for 7 days. Culture conditions were replenished on day 4. The organoid viability was then determined as above.

Dose–response curves are normalized to 0 nM gemcitabine or 0 nM quisinostat under the corresponding concentrations of pemigatinib. IC_50_ values were determined by Graphpad Prism 8.1.0. Synergism was calculated by MacSynergy II. Values that are above the 0 plane are considered synergistic, parallel to 0 as additive, and below 0 as antagonistic.

### Flow cytometry

Cells were seeded in 10-cm dishes at 1:10 ratio for 2 days after reaching ~70–80% confluence. Media was then refreshed to include pemigatinib and/or quisinostat at desired concentrations. Cells are treated for ~48 h before analysis. For Ki-67 analyses, cells were harvested, pelleted, washed, and fixed in 70% ethanol for 30 min at 4 °C. After fixation, cells were washed twice with flow buffer (1× DPBS + 0.5% BSA) and stained by AlexaFluor 488-conjugated Ki-67 antibody (1:50, CST, Cat# 11882) for 30 min. Then the cells were washed twice and finally resuspended in flow buffer with 100 μg/mL RNase A (QIAGEN, Cat# 1031301) and 50 μg/mL propidium iodide (PI, Sigma-Aldrich, Cat# P4170). For apoptosis analyses, cell culture media was collected in 15 mL tubes and cells were dissociated by 0.25% trypsin. Both the cell culture media and dissociated cells were pelleted at 2000 rpm at room temperature for 5 min. Then the pellets were washed twice in ice-cold 1× DPBS supplied with 1% HI-FBS. Cells were stained with Annexin V-FITC early apoptosis detection kit (CST, Cat# 6592) according to the manufacturer’s protocol.

Organoids were split 1:5–1:10 in 24-well plates and cultured in ICC assay media for 3 days after reaching near-confluence. Media was then refreshed to include pemigatinib and/or quisinostat at desired concentration, and the organoids were treated for ~72 h before analysis. To harvest organoids, matrigel was first disrupted in 1 mL/50 μL ice-cold 1× DPBS and transferred to a 15 mL tube pre-filled with 10 mL ice-cold 1× DPBS, and the organoid was pelleted at 2000 rpm for 5 min at 4 °C. Organoids were then digested into single cells by TrypLE Express at 37 °C for 10 min. Digested organoids were washed twice in ice-cold flow buffer and samples were prepared as described above for Ki-67 and apoptosis analyses. Flow cytometry data was collected by BD LSRII system and data were analyzed by FlowJo.

### In vivo experiments

Under the same IACUC-approved protocol, DUC18828 cells (4 × 10^6^) were injected in 100 μL of PBS/matrigel (1:1) into the flank of each NSG mouse (Jackson Lab). Tumor volume was measured 3x/ week until volume reached ~125 mm^3^. To test response of this model to pemigatinib in vivo, mice were randomized to pemigatinib (5 mg/kg oral gavage) or control (sham gavage with Ora-Plus suspension vehicle (Perrigo). To confirm synergy with quisinostat, mice were randomized to receive control (7% DMSO vehicle in Ora-Plus suspension), pemigatinib only (3 mg/kg), quisinostat only (16 mg/kg), and pemigatinib plus quisinostat (3 and 16 mg/kg, respectively). For all experiments, once treatment was initiated, tumors were measured 3×/week and animal weight was assessed 5×/week. Mice were sacrificed per protocol at trial endpoints.

### Bioinformatics analysis

The quality of the DNA-Seq and RNA-seq data from the PDC was evaluated using FastQC (https://www.bioinformatics.babraham.ac.uk/projects/fastqc/) version 0.11.9. The DNA sequencing libraries were aligned to the hg38 reference sequence obtained from the GATK^43,44^ v0 bundle using the BWA-MEM algorithm^45^ version 0.17.7. The resulting reads were sorted by coordinate and deduplicated using picard tools (https://broadinstitute.github.io/picard/) version 2.26.10. The base quality scores of resulting bam file were recalibrated using the BaseRecalibrator tool from GATK version 4.1.4.1. For the latter step, SNP and indel annotation files from the GATK hg38 v0 bundle were used. DELLY^46^ was used to identify structural variants in DNA-Seq data from these bam files. Specifically, structural variants were identified from the recalibrated bam files with delly2, version 0.9.1, and the BCF files generated by delly2 were annotated with sansa, version 0.0.8. Somatic single-nucleotide variants (SNVs) from the DNA-Seq data using b37 genomic coordinates were generated by the Marie-Josée and Henry R. Kravis Center for Molecular Oncology^21,47^.

STAR-Fusion^48,49^ version 1.10.1 was used to identify gene fusions and predict coding sequences of fusion transcripts. The inferred versions were validated in silico using FusionInspector^50^ version 2.6.0. The plug and play version of the Trinity Cancer Transcriptome Analysis Toolkit (CTAT) resource bundle built against GENCODE^51^ version 37 (March 01, 2021) annotation was used.

Primers for validating the genomic breakpoints were designed by first predicting the sequences of the translocated chromosomes flanking the breakpoints with custom R code using the Rsamtools package^52^. These sequences were then used to design primers with Primer3Plus and Primer-BLAST^53,54^. Breakpoints were confirmed by assembling Sanger sequencing results on the predicted sequences using SeqMan Ultra^TM^ (DNASTAR, Inc. Madison, Wisconsin USA). Chromatograms of breakpoints were generated with custom R code using the sangerseqR package^55^.

### Statistical considerations

For in vitro and in vivo experiments, basic descriptive statistics were used to determine significance in cell line, organoid, and murine experiments. The Student’s *t* test was used to compare two outcomes, and analysis of variance was used when three or more outcomes were compared. For measures of cancer biology, including cell line proliferation and apoptosis, three independent replicates (triplicates) were generated prior to data analysis. An unadjusted two-sided level of 0.05 was used for testing each hypothesis. For in vivo experiments comparing FGFR2 inhibition vs control (vehicle), a power analysis using linear mixed models and implemented via powerlmm in R^56^ indicated a sample size of 8 mice would be adequate to detect a 50% reduction in tumor volume in the treatment group relative to control (power = 0.80, alpha = 0.05). For the experiments evaluating a combined effect of FGFR2 inhibition and HDAC inhibition, 12 mice per arm were required to detect a 1/4 reduction in rate of increase in tumor volume in the combination therapy relative to monotherapy (power = 0.80, alpha = 0.05).

### Reporting summary

Further information on research design is available in the Nature Research Reporting Summary linked to this article.

## Supplementary information

Supplementary Material

REPORTING SUMMARY

Supplementary Data

## Data Availability

Data generated by the authors are included within the text and supplementary files or will be made available upon request (without restriction) to the corresponding author. The raw DNA-Seq, RNA-Seq, and Sanger sequencing data generated from the PDC have been deposited with links to BioProject accession number PRJNA854934 in the NCBI BioProject database (https://www.ncbi.nlm.nih.gov/bioproject/). Sequencing data from the patient’s tumor, concordant with that of the patient-derived models, are not available for distribution, as they are protected by HIPAA as part of the clinical record.

## Code availability

The scripts to reproduce the discovery analysis from the RNA-Seq and DNA-Seq data from the PDC, as well as the primer design are available through a public source code repository (https://gitlab.oit.duke.edu/dcibioinformatics/pubs/lidsky-fgfr2-fusion-paper). The latter also contains the recipe files for creating the singularity container images used to perform the analyses^57^.
