# Supplementary material for: Leveraging patient derived models of FGFR2 fusion positive intrahepatic cholangiocarcinoma to identify synergistic therapies
Source: NPJ Precis Oncol. 2022 Oct 23;6:75. doi: 10.1038/s41698-022-00320-5 (PMC9588766; doi:10.1038/s41698-022-00320-5)

Supplementary Figure 1

A

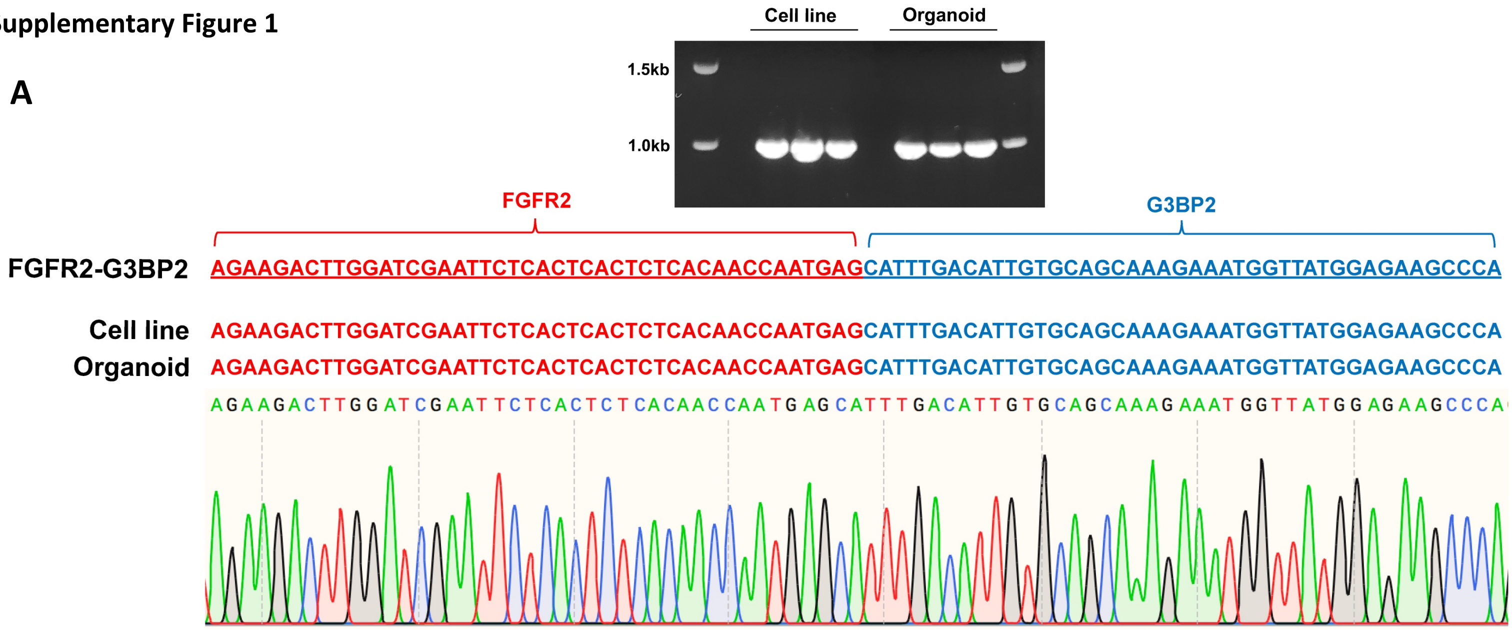

B

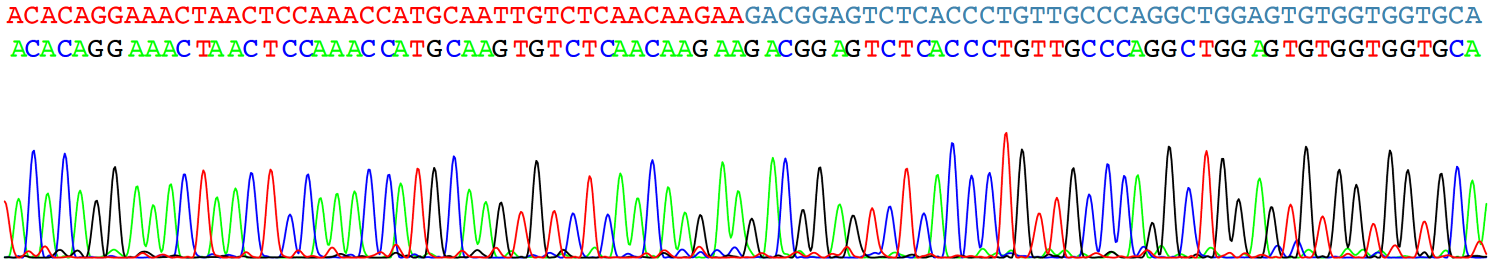

C

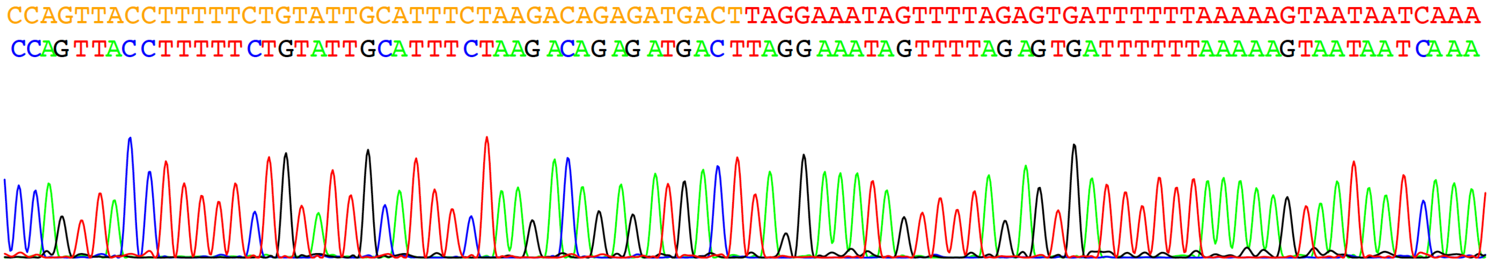

Supplementary Figure 1. PCR and Sanger sequencing confirm fusion and breakpoint sequences. (1A) DNA agarose gel of the RT-PCR products, in triplicate, for the PDC and PDO models, using primers spanning 1004 base pairs inclusive of the FGFR2-G3BP2 fusion. The RT-PCR products ran to the exact position as predicted (~1kb). The sanger sequencing chromatograms of the RT-PCR products are also shown. **(1B-C)** Chromatograms from Sanger sequencing of genomic DNA validate the breakpoints predicted by DNA-Seq. Above the sequence observed from the chromatogram, the predicted sequence flanking the breakpoint is shown for the FGFR2-G3BP2 breakpoint (B, FGFR2 flank in red text, G3BP2 flank in blue text) and the SAMD13-FGFR2 breakpoint (C, SAMD13 flank in orange text, FGFR2 flank in red text).

Supplementary Figure 2

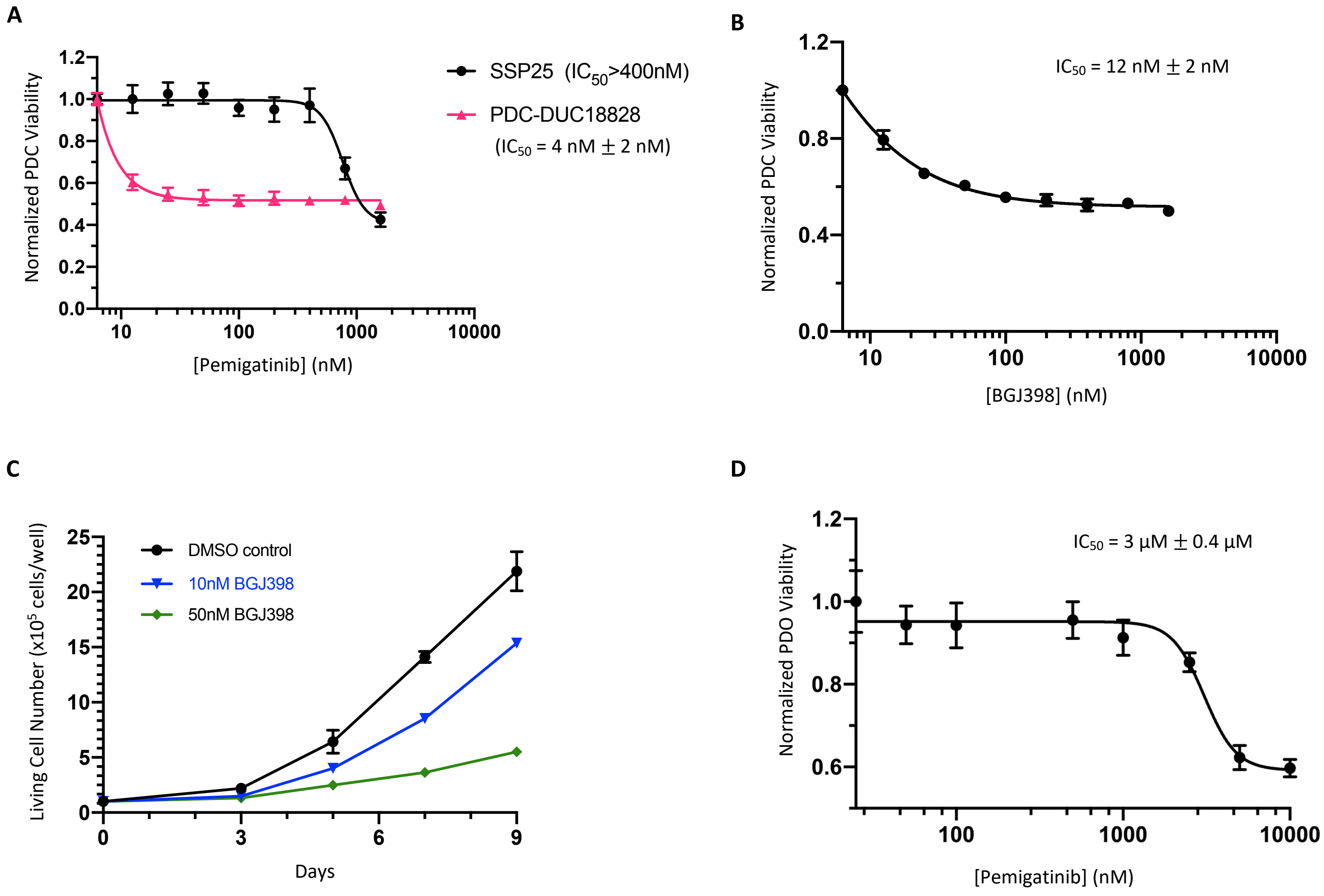

Supplementary Figure 2. FGFR inhibition in FGFR2 fusion-positive and wildtype models of ICC. (2A) Differential sensitivity to FGFR inhibition with pemigatinib in FGFR2 fusion-positive and wildtype cell lines. GI_{50} assay with pemigatinib in the PDC-DUC18828 model confirmed the IC_{50} value to be 4 nM, compared to > 400 nM in SSP-25, an established ICC cell line with no FGFR alterations. These assays were performed with Alamarblue after 3 days treatment **(2B-C)** The patient-derived FGFR2 fusion-positive DUC18828 cell line is sensitive to BGJ398, another selective FGFR inhibitor. **(2B)** GI_{50} assay with BGJ398 in the PDC-DUC18828 model confirmed the IC_{50} value to be 12 nM. This assay was performed with Alamarblue after 3 days treatment. **(2C)** Sensitivity of PDC-DUC18828 to BGJ398 is dose-dependent. In this *in vitro* proliferation assay, the number of living cells counted at Day 3, 5, 7, and 9 are plotted against days of treatment, demonstrating that increasing dose of BGJ398 is associated with a concordant impairment in viability in the PDC model. **(2D)** Growth factors in organoid culture media cause resistance to FGFR inhibition. GI_{50} assay with pemigatinib in the presence of 100 ng/mL hEGF, 25 ng/mL hHGF, and 100 ng/mL hFGF10 confirmed the IC_{50} value to be 3 uM in the PDO-DUC18828 model, more than 1000-fold higher than the IC_{50} in this model when growth factors are removed from culture media. Results are normalized to DMSO control and presented as the mean +/- deviation (error bars) for three biologically independent replicates.

Supplementary Figure 3

A

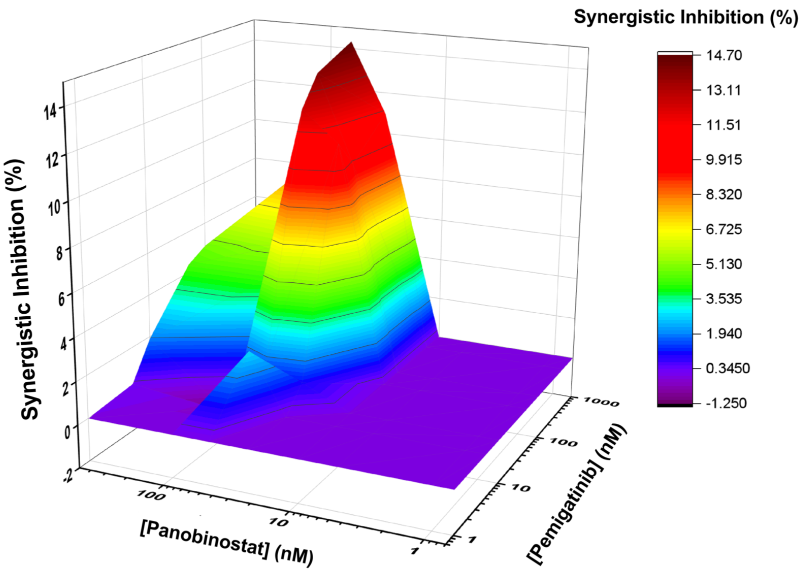

B

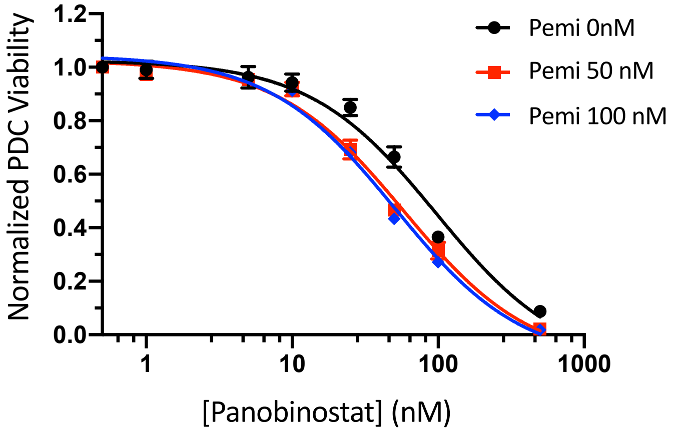

Supplementary Figure 3. Confirmation of HDAC and FGFR synergy with panobinostat and pemigatinib. (3A) Panobinostat, another pan-HDAC inhibitor, and pemigatinib have synergistic effects on viability in the PDC-DUC18828 model based on MacSynergy II calculation (95% confidence interval). Results are color-coded from purple (most antagonistic) to dark red (most synergistic). Data below the 0 plane are antagonistic, in the plane are additive, and above the plane are synergistic. **(3B)** Dose-response curves in the PDC-DUC18838 model shift left with increasing concentrations of pemigatinib, resulting in reduction in the IC_{50} value of panobinostat, concordant with synergy observed in (Supplementary Figure 3A). All curves are normalized to 0nM panobinostat under the corresponding concentrations of pemigatinib and presented as the mean +/- standard deviation (error bars) for three biologically independent replicates.

Supplementary Figure 4

A

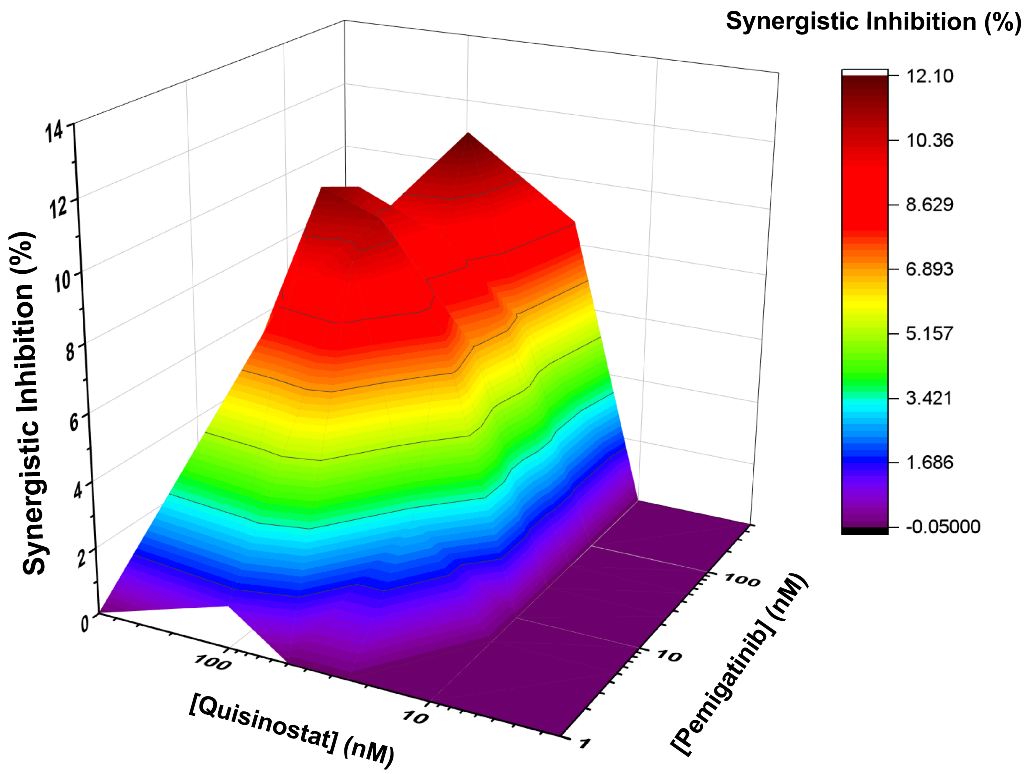

B

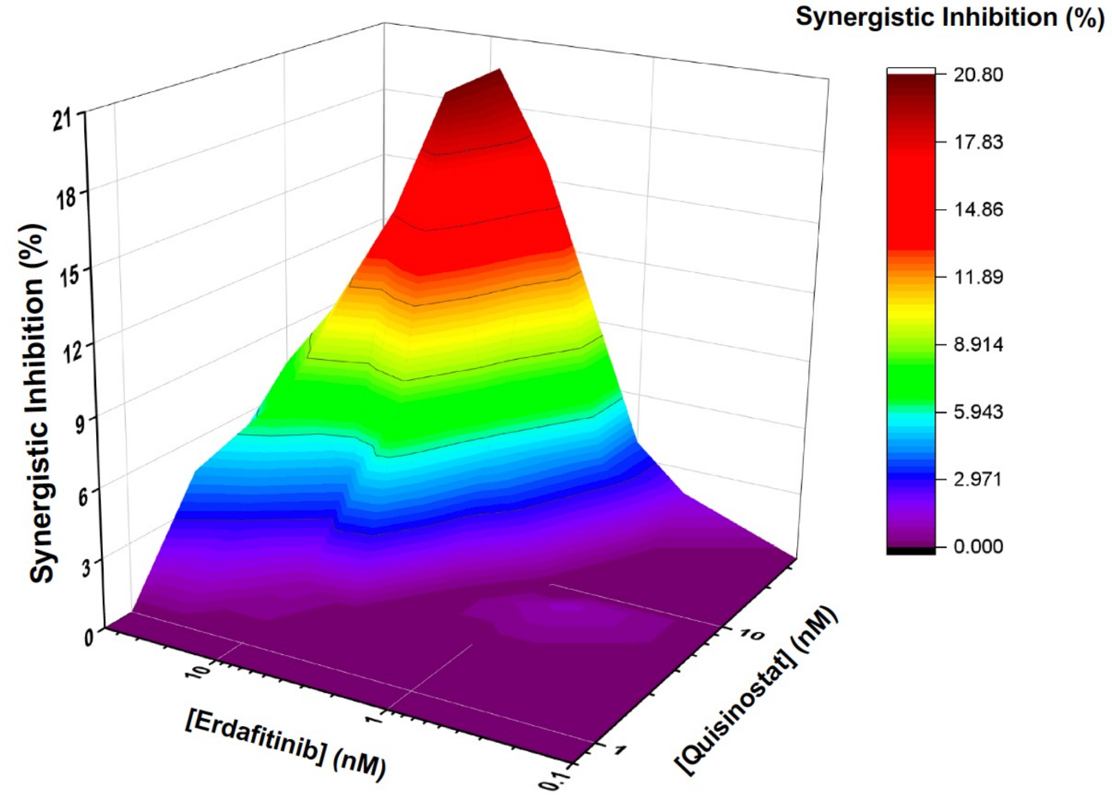

Supplementary Figure 4. FGFR and HDAC inhibition are synergistic in FGFR fusion-positive urothelial bladder cancer and breast cancer. (4A) Quisinostat and pemigatinib synergistically impair viability of RT4 cells (patient-derived bladder cancer cell line that possesses a FGFR3-TACC3 fusion), based on MacSynergy II calculation (95% confidence interval). **(4B)** Quisinostat and erdafitinib (FGFR1-4 inhibitor) synergistically impair viability in SUM185PE cells (estrogen receptor-negative, progesterone receptor-negative, HER2-positive breast cancer cell line that possesses a FGFR3-TACC3 fusion), based on MacSynergy II calculation (95% confidence interval). Results for Supplementary Figure 4A-B are color-coded from purple (most antagonistic) to dark red (most synergistic). Data below the 0 plane are antagonistic, in the plane are additive, and above the plane are synergistic.

Supplementary Figure 5

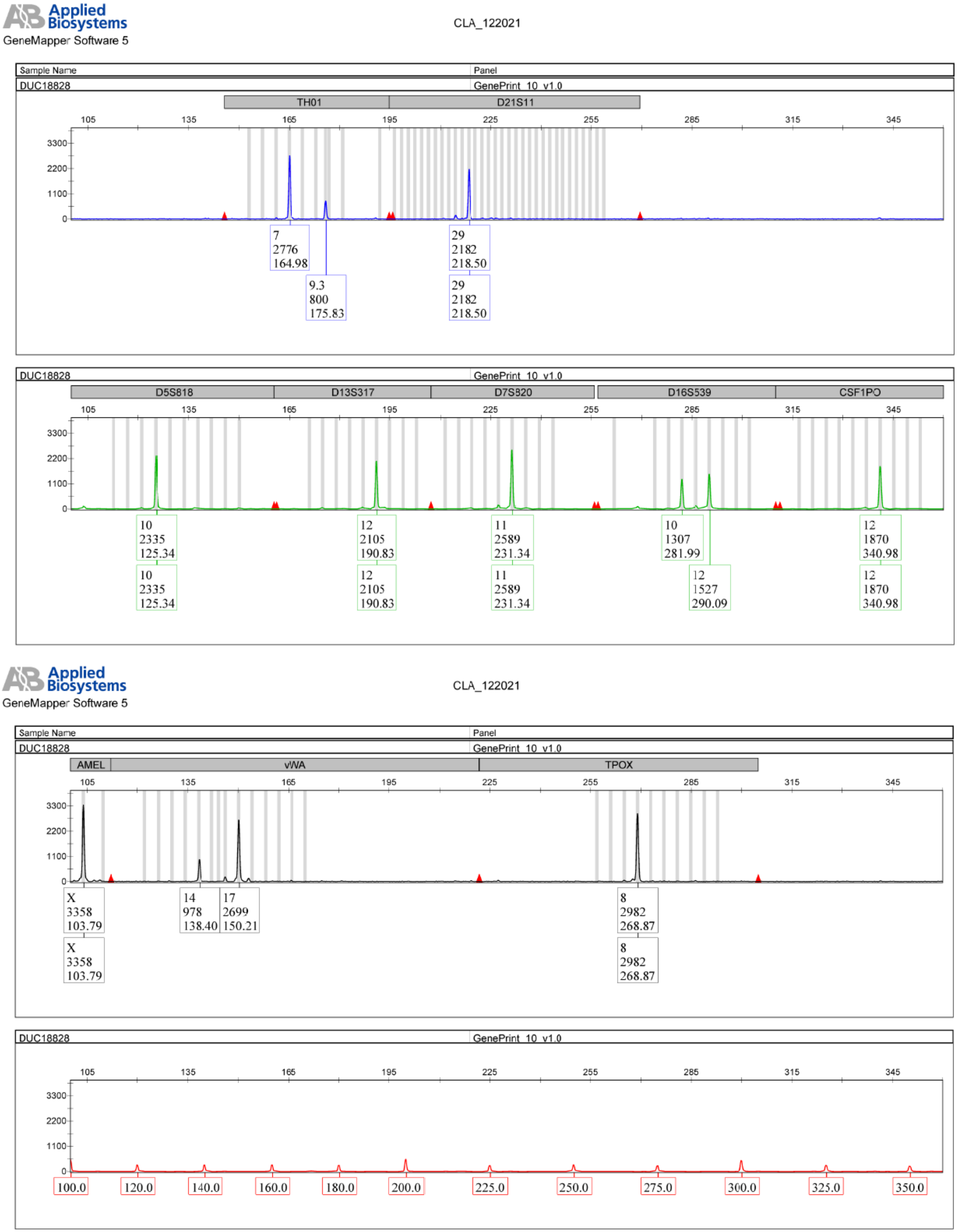

Supplementary Figure 5. Short tandem repeat analysis of PDC-DUC18828, a patient-derived model of FGFR2 fustion-positive ICC. Chromatographs from STR analysis are provided.

Supplementary Figure 6

A

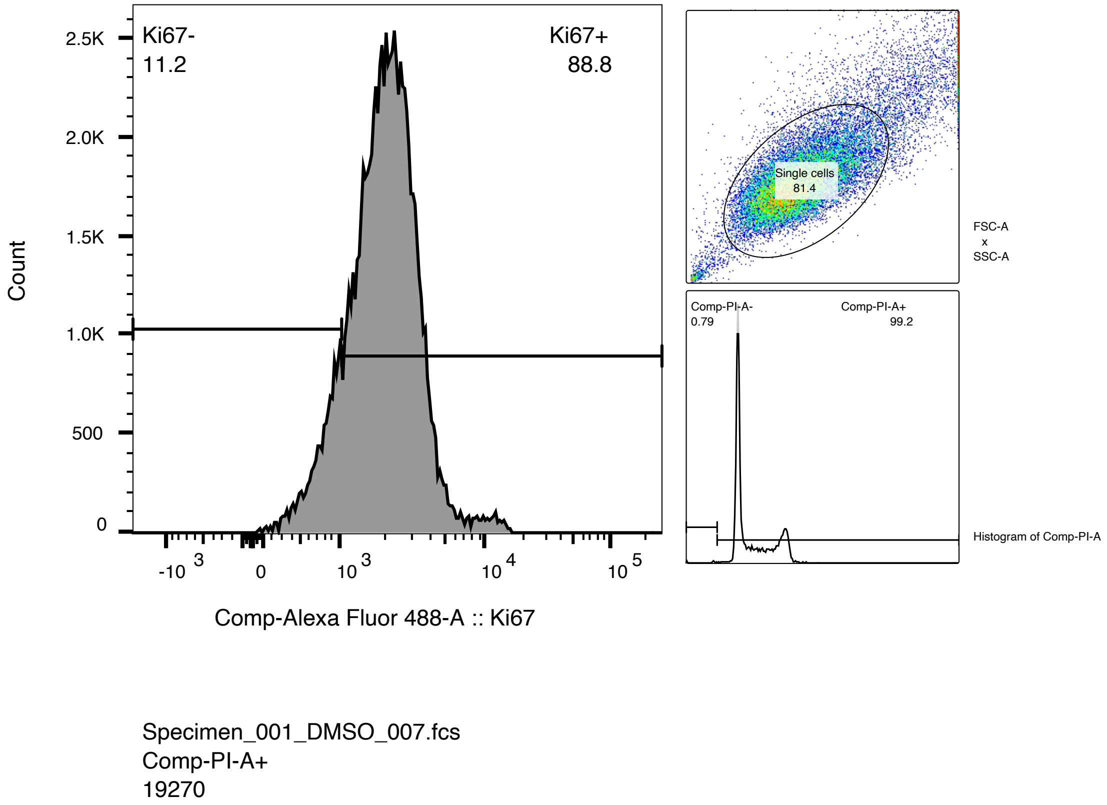

B

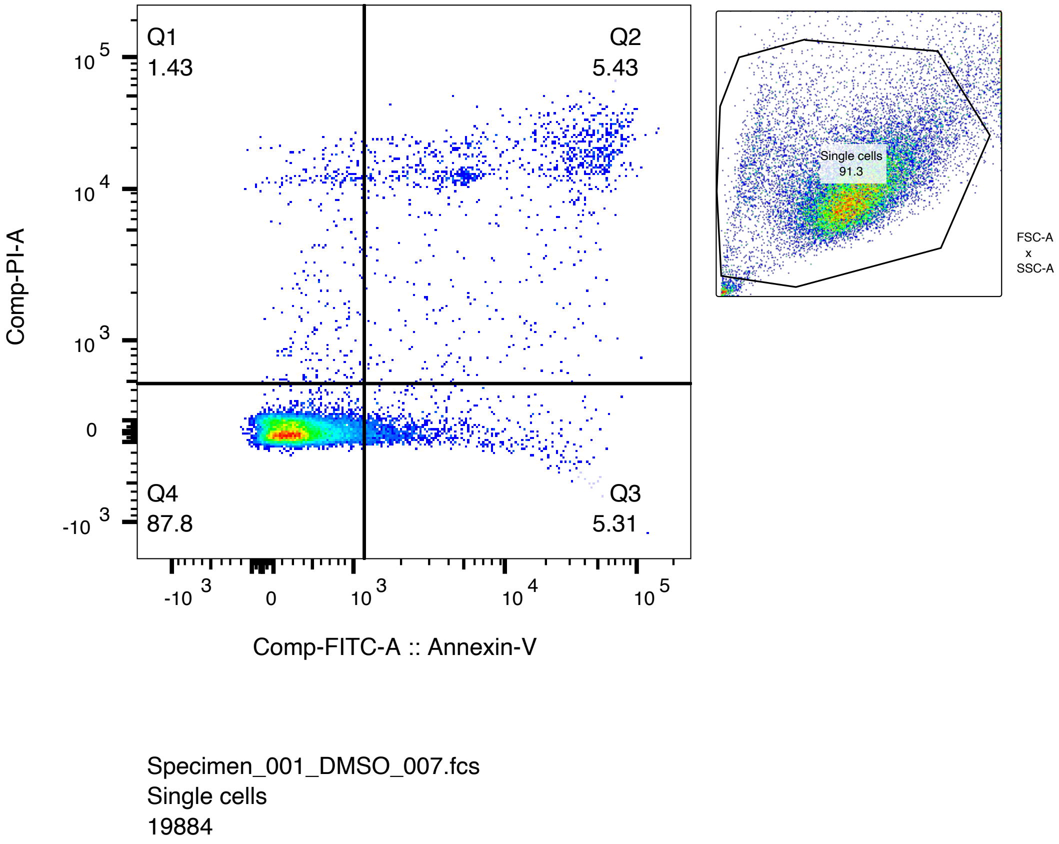

Supplementary Figure 6. Flow cytometry gating strategies. (A) Gating strategy for Ki67, corresponding to main text figure 7A-B. **(B)** Gating strategy for Annexin-V, corresponding to main text figure 7C-D.

Supplementary File 1. Reporting of all inferred structural variants and gene fusions from from PDC DUC18828 DNA- and RNA-sequencing data. Tables 1 and 2 of this supplemental report provide a listing of inferred single nucleotide variant (SNV) somatic mutations and structural variants from the PDC-DUC18828 DNA-Sequencing data, respectively. Table 3 provides a listing of inferred gene fusions from the PDC-DUC18828 RNA-Sequencing data.
(Link to html file: https://gitlab.oit.duke.edu/dcibioinformatics/pubs/lidsky-fgfr2-fusion-paper/-/raw/main/Report/DNA-RNA-Seq-DUC18828.html?inline=false)

Supplementary Table 1. Results from high-throughput small molecule screen. Table includes the small molecule/drug tested with pemigatinib, the known target/pathway of that small molecule/drug, raw cell viability data (in triplicate) for 1 uM library compound alone and in combination with 1 nM pemigatinib (determined by CellTiter Glo, CTG), normalized viability to DMSO control, averaged normalized viability, and the synergy score resulting from these normalized and averaged triplicate data comparing mono- versus combination therapy.

Small Molecule	Target(s)	Cell Viability (1 uM library compound only)			Cell Viability (1 uM library compound + 1 nM pemigatinib)			Cell Viability normalized to DMSO control (1 uM library compound only)			Cell Viability normalized to DMSO control (1 uM library compound + 1 nM pemigatinib)			Average normalized cell viability (1 uM library compound only)	Average normalized cell viability (1 uM library compound + 1 nM pemigatinib)	Synergy Score
		Replicate 1	Replicate 2	Replicate 3	Replicate 1	Replicate 2	Replicate 3	Replicate 1	Replicate 2	Replicate 3	Replicate 1	Replicate 2	Replicate 3			
Dinaciclib (SCH727965)	CDK	56673	58992	59264	11721	10583	12146	1.09	1.09	1.14	0.27	0.25	0.30	1.10	0.29	0.26
Disulfiram	Dehydrogenase	13375	11577	17570	4076	6605	3843	0.25	0.22	0.33	0.09	0.14	0.09	0.26	0.11	0.40
SC144	Others	5649	4649	5519	1628	2675	1733	0.11	0.09	0.11	0.04	0.06	0.04	0.10	0.05	0.46
SP2509	Histone Demethylase	24168	28199	24847	9445	12469	10322	0.47	0.55	0.47	0.21	0.27	0.23	0.50	0.24	0.48
XL888	HSP (e.g. HSP90)	56744	17751	18817	13519	13577	11020	1.11	0.34	0.36	0.31	0.31	0.26	0.60	0.29	0.49
Panobinostat (LBH589)	HDAC	5554	6034	5782	2769	2850	3134	0.11	0.11	0.11	0.06	0.06	0.07	0.11	0.06	0.57
CUDC-907	PI3K,HDAC	2629	2517	3161	1439	1056	1090	0.05	0.05	0.06	0.03	0.03	0.03	0.05	0.03	0.58
Ciclopirox ethanolamin	ATPase	33374	30697	28916	14025	13110	14237	0.64	0.57	0.56	0.33	0.31	0.35	0.59	0.34	0.58
Quisinostat (JNJ-26481	HDAC	5329	5954	5990	3438	2964	2587	0.11	0.11	0.11	0.08	0.06	0.06	0.11	0.07	0.59
Dopamine HCl	Dopamine Receptor	29432	23741	27193	13981	13424	14334	0.56	0.46	0.51	0.31	0.29	0.32	0.51	0.31	0.60
Romidepsin (FK228, De	HDAC	3113	2929	3035	1616	1152	1309	0.06	0.05	0.06	0.04	0.03	0.03	0.06	0.03	0.61
Trichostatin A (TSA)	HDAC	6427	7971	8758	3707	4248	4810	0.13	0.15	0.17	0.08	0.09	0.10	0.15	0.09	0.62
PD 151746	Cysteine Protease	137844	50633	55060	45448	46486	41762	2.69	0.99	1.04	1.02	1.02	0.93	1.57	0.99	0.63
PI-103	PI3K,DNA-PK,Autophagy,mTOR	21680	33134	22780	16480	15040	15693	0.44	0.63	0.44	0.37	0.33	0.34	0.50	0.34	0.69
GDC-0032	PI3K	48435	42205	40554	27638	23260	24067	0.95	0.80	0.77	0.63	0.53	0.57	0.84	0.58	0.69
Brefeldin A	ATPase,Autophagy	1161	1148	1180	622	816	632	0.02	0.02	0.02	0.01	0.02	0.01	0.02	0.02	0.71
Dasatinib	Bcr-Abl,Src,c-Kit	19471	24756	22065	13715	14783	14046	0.40	0.47	0.42	0.30	0.32	0.30	0.43	0.31	0.72
PF-04691502	mTOR,Akt,PI3K	11559	11783	11770	7017	7104	6377	0.22	0.22	0.23	0.16	0.17	0.16	0.22	0.16	0.72
Zinc Pyrithione	Proton Pump	8010	8223	6462	2436	4521	6102	0.15	0.15	0.12	0.06	0.11	0.15	0.14	0.10	0.72
M344	HDAC	38858	39670	42371	23105	26136	23347	0.74	0.73	0.81	0.54	0.62	0.57	0.76	0.56	0.73
Tyrphostin AG 879	HER2	46195	50612	46892	28871	30503	26812	0.88	0.93	0.90	0.67	0.72	0.66	0.91	0.67	0.74
OSI-027	mTOR	17845	15688	17884	11670	11477	9636	0.34	0.31	0.34	0.26	0.25	0.22	0.33	0.24	0.74
MK-5108 (VX-689)	Aurora Kinase	26362	24937	24972	16141	15547	13542	0.51	0.46	0.48	0.38	0.37	0.33	0.48	0.35	0.74
GSK2126458 (GSK458)	mTOR,PI3K	14478	13684	14469	9127	9176	8950	0.28	0.27	0.27	0.20	0.20	0.20	0.27	0.20	0.74
LMK-235	HDAC	33085	28856	34167	20357	20813	20584	0.64	0.56	0.65	0.46	0.46	0.46	0.62	0.46	0.74
Etoposide	Topoisomerase	30276	34681	52664	26096	26105	25860	0.62	0.66	1.01	0.58	0.57	0.56	0.76	0.57	0.75
SCR7	DNA/RNA Synthesis	83726	55462	58826	42214	43607	43534	1.63	1.08	1.12	0.94	0.96	0.97	1.28	0.96	0.75
Pracinostat (SB939)	HDAC	29650	32432	32293	21545	21371	20437	0.61	0.61	0.62	0.48	0.47	0.44	0.61	0.46	0.75
Paclitaxel	Autophagy,Microtubule Associated	12663	15185	16375	11430	9165	9644	0.26	0.29	0.31	0.25	0.20	0.21	0.29	0.22	0.77
AT101	Bcl-2	36242	32231	34575	21399	22173	20598	0.69	0.59	0.66	0.50	0.53	0.51	0.65	0.50	0.77
AZ5104	EGFR	49589	52655	54283	34352	33394	32865	0.97	1.00	1.03	0.78	0.77	0.78	1.00	0.78	0.77
Omaveloxolone (RTA-4	Others	2603	2856	2940	1663	1870	2146	0.05	0.06	0.06	0.04	0.04	0.05	0.05	0.04	0.78
JNK Inhibitor IX	JNK	20846	18690	16883	12130	13562	12680	0.41	0.36	0.32	0.27	0.30	0.28	0.36	0.28	0.78
Icotinib	EGFR	51021	57286	54318	35155	33767	31794	0.98	1.06	1.04	0.82	0.80	0.78	1.03	0.80	0.78
Givinostat (ITF2357)	HDAC	26725	29370	28488	19023	18755	19534	0.51	0.57	0.54	0.42	0.41	0.44	0.54	0.42	0.78
Ro3280	PLK	14147	17895	17488	11218	10247	10833	0.28	0.34	0.33	0.26	0.24	0.26	0.32	0.25	0.79
MLN0905	PLK	12506	14601	13924	9156	8962	7864	0.24	0.27	0.27	0.21	0.21	0.19	0.26	0.20	0.79
Cephalomannine	Others	11991	12721	12948	7343	9886	8536	0.23	0.25	0.24	0.16	0.22	0.19	0.24	0.19	0.79
Ciclopirox	ATPase	37332	35670	36599	22023	26414	26714	0.71	0.70	0.69	0.49	0.58	0.60	0.70	0.55	0.79
BGT226 (NVP-BGT226)	mTOR,PI3K	1037	1072	736	552	452	631	0.02	0.02	0.01	0.01	0.01	0.02	0.02	0.01	0.79
Saracatinib (AZD0530)	Bcr-Abl,Src	44951	42837	46994	32115	31397	31882	0.92	0.81	0.90	0.71	0.69	0.69	0.88	0.70	0.79
TAK-733	MEK	33089	30048	40932	24060	23661	23763	0.63	0.59	0.77	0.53	0.52	0.53	0.66	0.53	0.79
Epothilone B (EPO906,	Microtubule Associated	12005	13846	14997	9941	9229	9755	0.25	0.26	0.29	0.22	0.20	0.21	0.27	0.21	0.80
Dexamethasone Acetat	Autophagy,IL Receptor	50974	53311	51863	34081	34888	31588	0.98	0.98	1.00	0.79	0.83	0.78	0.98	0.79	0.80
Rigosertib (ON-01910)	PLK	13613	12965	14657	8424	9625	11346	0.28	0.25	0.28	0.19	0.21	0.25	0.27	0.21	0.80
Azacyclonol	Others	51593	53009	54656	35572	37667	32005	0.99	0.98	1.05	0.83	0.89	0.79	1.00	0.81	0.80
Trametinib (GSK11202	MEK	47759	46721	44860	33995	33369	29921	0.91	0.91	0.85	0.75	0.73	0.67	0.89	0.72	0.81
(+)-JQ1	Epigenetic Reader Domain	43852	49258	39984	30244	29368	29555	0.86	0.94	0.76	0.69	0.67	0.70	0.85	0.69	0.81
Varlitinib	EGFR	49659	54378	54356	36697	32593	30719	0.95	1.00	1.04	0.86	0.77	0.76	1.00	0.81	0.81
BKM120 (NVP-BKM120	PI3K	35799	36474	33488	24373	24952	24800	0.68	0.71	0.63	0.54	0.54	0.56	0.68	0.55	0.81
ML161	Others	52937	54699	53579	36030	38542	32675	1.01	1.01	1.03	0.84	0.91	0.80	1.02	0.82	0.81
Sofosbuvir (PSI-7977, G	DNA/RNA Synthesis	67876	57413	54755	41719	36485	35214	1.30	1.06	1.05	0.97	0.86	0.87	1.14	0.92	0.81
CAY10603	HDAC	29828	29564	28534	18484	21982	21523	0.58	0.58	0.54	0.41	0.48	0.48	0.57	0.46	0.81
Sertaconazole nitrate	Others	52199	49575	53619	34512	3350	32147	1.00	0.91	1.03	0.80	0.08	0.79	0.98	0.80	0.81
AZD8931 (Sapitinib)	EGFR,HER2	52040	51568	51833	35563	36288	37888	0.99	1.01	0.98	0.79	0.79	0.85	0.99	0.81	0.81
Torin 2	mTOR,ATM/ATR	13008	13087	13250	9072	8142	7862	0.25	0.24	0.25	0.21	0.19	0.19	0.25	0.20	0.82
GNE-317	PI3K	13238	14897	14716	9765	9843	10825	0.26	0.29	0.28	0.22	0.22	0.24	0.28	0.23	0.82

Artesunate	Others	60810	35825	38521	32609	34198	28974	1.16	0.70	0.73	0.72	0.75	0.65	0.86	0.71	0.82
Sulfamerazine	Others	55466	55975	55429	37019	169	35094	1.06	1.03	1.06	0.86	0.00	0.86	1.05	0.86	0.82
Methylclothiazide	Others	55240	61021	57126	41381	43441	33663	1.06	1.12	1.10	0.96	1.03	0.83	1.09	0.90	0.82
KW-2478	HSP (e.g. HSP90)	44001	42002	37536	30469	31150	26468	0.84	0.82	0.71	0.67	0.68	0.59	0.79	0.65	0.82
ARQ 621	Kinesin	17967	18112	17026	11684	13352	13003	0.35	0.35	0.32	0.26	0.29	0.29	0.34	0.28	0.82
BI 2536	PLK	19859	19659	20267	13329	16470	14128	0.41	0.37	0.39	0.30	0.36	0.31	0.39	0.32	0.82
PD0325901	MEK	39766	41203	39244	30888	26613	31338	0.81	0.78	0.75	0.68	0.58	0.68	0.78	0.65	0.83
NVP-AEW541	IGF-1R	47053	48167	49988	37229	31600	38492	0.96	0.91	0.96	0.83	0.69	0.83	0.94	0.78	0.83
BYL719	PI3K	49607	50596	53004	36256	35858	30816	0.95	0.93	1.02	0.85	0.85	0.76	0.97	0.80	0.83
PF-4989216	PI3K	36853	35020	38389	26104	26267	27246	0.72	0.68	0.73	0.58	0.58	0.61	0.71	0.59	0.83
TAK-285	HER2,EGFR	52239	54214	55189	37621	32825	33174	1.00	1.00	1.06	0.88	0.78	0.82	1.02	0.85	0.83
Tripelennamine HCl	Histamine Receptor	52999	55851	50368	37302	149	32413	1.02	1.03	0.97	0.87	0.00	0.80	1.00	0.83	0.83
AMG-458	c-Met	52356	53608	51800	35286	34141	33734	1.00	0.99	0.99	0.82	0.81	0.83	1.00	0.83	0.83
SB705498	TRPV	60014	59508	57462	41435	38121	36184	1.15	1.10	1.10	0.97	0.90	0.89	1.12	0.93	0.83
Azatadine dimaleate	Histamine Receptor	40148	40623	44484	28800	26005	26137	0.77	0.75	0.85	0.67	0.62	0.64	0.79	0.66	0.83
GSK461364	PLK	17154	17390	16379	11822	12651	12289	0.33	0.34	0.31	0.26	0.28	0.28	0.33	0.27	0.83
Atovaquone	Others	55507	54348	55766	40707	124	32207	1.06	1.00	1.07	0.95	0.00	0.79	1.05	0.87	0.83
HSP990 (NVP-HSP990)	HSP (e.g. HSP90)	17784	19630	18069	11917	13606	12931	0.35	0.37	0.34	0.27	0.31	0.31	0.36	0.30	0.83
G007-LK	PARP	45439	43721	47929	30527	33995	30780	0.89	0.83	0.91	0.70	0.78	0.73	0.88	0.74	0.84
Pidotimod	Others	48912	53995	51906	35208	159	33131	0.94	1.00	1.00	0.82	0.00	0.82	0.98	0.82	0.84
PD168393	EGFR	36878	38874	33847	25212	25444	25669	0.72	0.74	0.64	0.58	0.58	0.61	0.70	0.59	0.84
RO5126766 (CH512676	Raf	44798	46182	41414	29830	32416	30162	0.88	0.88	0.79	0.68	0.74	0.71	0.85	0.71	0.84
Pimasertib (AS-703026	MEK	46001	51828	51358	37448	37053	37102	0.94	0.98	0.98	0.83	0.81	0.80	0.97	0.81	0.84
Picropodophyllin (PPP)	IGF-1R	26180	24690	26645	15710	19697	21308	0.51	0.48	0.51	0.35	0.43	0.47	0.50	0.42	0.84
Homatropine Bromide	AChR	54141	54613	56623	39394	37621	34000	1.04	1.01	1.09	0.92	0.89	0.84	1.04	0.88	0.84
Carfilzomib (PR-171)	Proteasome	983	1502	1689	787	1055	1053	0.02	0.03	0.03	0.02	0.02	0.03	0.03	0.02	0.84
CI994 (Tacedinaline)	HDAC	52262	52527	52063	35341	35706	34285	1.00	0.97	1.00	0.82	0.85	0.84	0.99	0.83	0.84
Mirabegron	Adrenergic Receptor	50887	53393	53017	37370	36982	32547	0.97	0.98	1.02	0.87	0.88	0.80	0.99	0.84	0.84
Ropinirole HCl	Dopamine Receptor	53022	53757	53573	35170	36128	35962	1.02	0.99	1.03	0.82	0.86	0.89	1.01	0.85	0.84
S-Ruxolitinib (INCB018	JAK	57096	59184	57308	41426	36938	35767	1.09	1.09	1.10	0.97	0.87	0.88	1.09	0.92	0.84
AZD8186	PI3K	38743	38833	44079	29915	28454	30814	0.76	0.76	0.84	0.67	0.63	0.69	0.78	0.66	0.84
Halobetasol Propionate	Phospholipase (e.g. PLA)	48348	48663	49959	34145	33419	31349	0.93	0.90	0.96	0.80	0.79	0.77	0.93	0.78	0.85
Afatinib (BIBW2992) Di	HER2,EGFR	44498	46201	42606	32795	30430	34850	0.87	0.90	0.81	0.73	0.67	0.78	0.86	0.73	0.85
LFM-A13	BTK	53752	50022	54631	38650	37168	40685	1.05	0.98	1.04	0.86	0.82	0.91	1.02	0.86	0.85
SB415286	GSK-3	51310	58454	53197	37450	40705	35125	0.98	1.08	1.02	0.87	0.96	0.87	1.03	0.87	0.85
Afatinib (BIBW2992)	EGFR,HER2	40687	44714	48217	34768	33997	31855	0.83	0.85	0.92	0.77	0.74	0.69	0.87	0.73	0.85
ONX-0914 (PR-957)	Proteasome	1185	1051	931	625	701	898	0.02	0.02	0.02	0.01	0.02	0.02	0.02	0.02	0.85
Guanabenz Acetate	Adrenergic Receptor	56730	55801	57752	41288	39345	34792	1.09	1.03	1.11	0.96	0.93	0.86	1.07	0.91	0.85
PD318088	MEK	53082	50394	51442	37245	34598	38971	0.98	0.95	0.96	0.81	0.74	0.89	0.96	0.81	0.85
Mechlorethamine HCl	DNA/RNA Synthesis	53104	55399	52926	35558	36688	41143	1.04	1.05	1.01	0.81	0.84	0.97	1.03	0.88	0.85
KX2-391	Src	14382	15088	13592	11020	11099	9514	0.27	0.29	0.26	0.24	0.24	0.21	0.28	0.23	0.85
Pentamidine	Others	53684	57859	53885	37280	43117	36500	1.03	1.07	1.03	0.87	1.02	0.90	1.04	0.88	0.85
Propranolol HCl	Adrenergic Receptor	55643	58910	54956	38527	39673	37117	1.07	1.09	1.05	0.90	0.94	0.91	1.07	0.91	0.85
Niflumic acid	GABA Receptor,COX	51810	54780	50284	37480	35855	32650	0.99	1.01	0.97	0.87	0.85	0.80	0.99	0.84	0.85
CH5138303	HSP (e.g. HSP90)	19791	17744	20554	14893	13623	14337	0.39	0.35	0.39	0.33	0.30	0.32	0.37	0.32	0.85
Bisacodyl	Others	49744	60580	55986	38987	33742	35335	0.95	1.12	1.07	0.91	0.80	0.87	1.05	0.89	0.85
ICG-001	Wnt/beta-catenin	53348	48435	73950	46864	39332	42776	1.01	0.95	1.40	1.04	0.86	0.96	1.12	0.95	0.85
Selumetinib (AZD6244)	MEK	44506	46571	51561	35662	36836	35373	0.91	0.88	0.99	0.79	0.81	0.77	0.93	0.79	0.85
PU-H71	HSP (e.g. HSP90)	28095	34089	27013	22365	22320	21311	0.55	0.67	0.51	0.50	0.49	0.47	0.57	0.49	0.85
CCT137690	Aurora Kinase	41873	40805	44763	31886	32802	25353	0.80	0.75	0.86	0.74	0.78	0.62	0.80	0.68	0.85
Ciproxifan	Histamine Receptor	51345	59395	53414	40022	35391	33598	0.98	1.09	1.03	0.93	0.84	0.83	1.03	0.88	0.85
Entacapone	Histone Methyltransferase	53542	54128	54256	40250	107	32801	1.03	1.00	1.04	0.94	0.00	0.81	1.02	0.87	0.85
PF-4981517	P450 (e.g. CYP17)	52379	51716	56352	36474	33123	35785	1.00	0.95	1.08	0.85	0.78	0.88	1.01	0.87	0.85
Ouabain	Sodium Channel	7033	6771	6363	5260	2031	3859	0.13	0.12	0.12	0.12	0.05	0.10	0.13	0.11	0.86
LY2090314	GSK-3	32540	33626	32963	23747	21950	24739	0.64	0.64	0.63	0.54	0.50	0.58	0.64	0.54	0.86
Carbimazole	Others	56609	55274	57487	41104	162	35449	1.08	1.02	1.10	0.96	0.00	0.87	1.07	0.92	0.86
WZ811	CXCR	51160	58559	53938	40880	38774	33137	0.98	1.08	1.04	0.95	0.92	0.82	1.03	0.88	0.86
Darifenacin HBr	AChR	55493	55556	54116	40254	200	34479	1.06	1.02	1.04	0.94	0.00	0.85	1.04	0.89	0.86
Ethynodiol diacetate	Estrogen/progestogen Receptor	54866	58068	55311	41493	138	34664	1.05	1.07	1.06	0.97	0.00	0.85	1.06	0.91	0.86
L-Ascorbyl 6-palmitate	Others	56209	52594	51986	39534	41227	38862	1.07	1.03	0.98	0.87	0.90	0.87	1.03	0.88	0.86
AZD8330	MEK	32103	27742	30238	21274	23343	20872	0.59	0.52	0.56	0.46	0.50	0.48	0.56	0.48	0.86
Nocodazole	Autophagy,Microtubule Associated	20493	18747	18961	13132	12988	13193	0.39	0.35	0.36	0.31	0.31	0.32	0.37	0.32	0.86
WHI-P154	JAK,EGFR	48761	46186	50184	35069	35670	30734	0.93	0.85	0.96	0.82	0.84	0.76	0.92	0.79	0.86
CEP-33779	JAK	49283	54262	54331	35123	37376	36235	0.94	1.00	1.04	0.82	0.89	0.89	1.00	0.86	0.86
Moguisteine	Others	52721	52946	51877	38952	42617	32569	1.01	0.98	1.00	0.91	1.01	0.80	0.99	0.86	0.86
IOX2	HIF	51659	53708	55778	38961	36341	34235	0.99	0.99	1.07	0.91	0.86	0.84	1.02	0.88	0.86
TAK-715	p38 MAPK	50954	51106	47879	35579	36063	32473	0.98	0.94	0.92	0.83	0.85	0.80	0.95	0.81	0.86
Cobimetinib (GDC-0973	MEK	35894	35559	35217	27180	26492	26277	0.70	0.69	0.67	0.61	0.58	0.59	0.69	0.59	0.86

Butein	EGFR	56731	51201	58060	42037	40010	42289	1.11	1.00	1.10	0.94	0.88	0.94	**1.07**	**0.92**	0.86
TW-37	Bcl-2	9902	10092	8466	6473	7913	7512	0.20	0.19	0.16	0.14	0.17	0.16	**0.19**	**0.16**	0.86
Lithocholic acid	FXR	52912	56167	50825	39775	39068	32923	1.01	1.04	0.98	0.93	0.93	0.81	**1.01**	***0.87***	0.86
OSI-420	EGFR	56788	53540	53430	41632	38378	42294	1.08	1.05	1.01	0.92	0.84	0.95	**1.05**	**0.90**	0.86
Estradiol Cypionate	Estrogen/progestogen Receptor	51464	54374	53915	38807	37211	33858	0.99	1.00	1.03	0.90	0.88	0.83	**1.01**	***0.87***	0.86
Trifluoperazine 2HCl	Autophagy	51502	50049	50503	40011	35705	29477	0.99	0.92	0.97	0.93	0.85	0.73	**0.96**	***0.83***	0.86
Droperidol	Others	54476	59573	58092	41310	36294	37100	1.04	1.10	1.12	0.96	0.86	0.91	**1.09**	***0.94***	0.86
HMN-214	PLK	25942	31790	31863	21973	21871	25066	0.53	0.60	0.61	0.49	0.48	0.54	**0.58**	**0.50**	0.86
Dehydroepiandrosteron	Estrogen/progestogen Receptor,Androgen I	61887	56945	55363	43695	43468	43366	1.18	1.11	1.05	0.97	0.95	0.97	**1.11**	**0.96**	0.86
Butenafine HCl	Others	50032	54646	54698	39267	37085	33474	0.96	1.01	1.05	0.92	0.88	0.82	**1.01**	***0.87***	0.87
Atomoxetine HCl	5-HT Receptor	54776	53864	56945	41168	199	34480	1.05	0.99	1.09	0.96	0.00	0.85	**1.05**	***0.90***	0.87
GDC-0068	Akt	54235	51523	53389	39023	33091	33680	1.04	0.95	1.02	0.91	0.78	0.83	**1.00**	***0.87***	0.87
Flavopiridol (Alvocidib)	CDK	12300	12462	14765	9962	10243	10275	0.25	0.24	0.28	0.22	0.22	0.22	**0.26**	**0.22**	0.87
Gefitinib (ZD1839)	EGFR	47056	51133	52219	38646	38417	38934	0.96	0.97	1.00	0.86	0.84	0.84	**0.98**	**0.85**	0.87
Apoptosis Activator 2	Caspase	45703	48755	47577	34082	30688	30784	0.88	0.90	0.91	0.79	0.73	0.76	**0.90**	***0.78***	0.87
AG-1478 (Tyrphostin A(	EGFR	49311	46766	53085	37016	35662	31253	0.94	0.86	1.02	0.86	0.84	0.77	**0.94**	***0.82***	0.87
ONO-4059	BTK	53031	47136	52584	39409	36931	38816	1.03	0.92	1.00	0.88	0.81	0.86	**0.98**	**0.85**	0.87
PRT062607 (P505-15, B	Syk	48934	47262	51506	34416	37057	39966	0.95	0.92	0.98	0.77	0.81	0.89	**0.95**	**0.82**	0.87
GSK923295	Kinesin	21089	18786	21453	15516	14035	14666	0.41	0.36	0.41	0.35	0.32	0.35	**0.39**	**0.34**	0.87
Istradefylline	Adenosine Receptor	53747	54628	57542	40847	37128	35128	1.03	1.01	1.10	0.95	0.88	0.87	**1.05**	***0.91***	0.87
Trimethoprim	Others	52013	54848	58605	40895	172	34888	1.00	1.01	1.13	0.95	0.00	0.86	**1.04**	***0.91***	0.87
GNF-2	Bcr-Abl	56192	55627	53538	41176	38354	34582	1.08	1.03	1.03	0.96	0.91	0.85	**1.04**	***0.91***	0.87
Methazolamide	Carbonic Anhydrase	55329	58738	54537	41936	42384	35298	1.06	1.08	1.05	0.98	1.00	0.87	**1.06**	***0.92***	0.87
5-hydroxytryptophan (5-	Others	56337	53464	57866	42939	41381	41825	1.07	1.04	1.09	0.95	0.90	0.94	**1.07**	**0.93**	0.87
MEK162 (ARRY-162, AR	MEK	48701	48543	46331	34087	34825	34718	0.96	0.92	0.88	0.78	0.80	0.82	**0.92**	**0.80**	0.87
Foscarnet Sodium	Reverse Transcriptase	54932	55155	55258	40563	203	35265	1.05	1.02	1.06	0.95	0.00	0.87	**1.04**	***0.91***	0.87
Galeterone	P450 (e.g. CYP17),Androgen Receptor	53324	58679	56044	43732	37668	33554	1.02	1.08	1.08	1.02	0.89	0.83	**1.06**	***0.92***	0.87
Vortioxetine (Lu AA210	5-HT Receptor	53081	54398	53808	40920	38558	42719	1.03	1.06	1.02	0.92	0.85	0.95	**1.04**	**0.90**	0.87
GW4064	FXR	52479	52234	55836	39145	37574	34741	1.01	0.96	1.07	0.91	0.89	0.86	**1.01**	***0.88***	0.87
Rapamycin (Sirolimus)	mTOR,Autophagy	31604	33087	33085	25037	25718	25300	0.65	0.63	0.63	0.55	0.56	0.55	**0.64**	**0.56**	0.87
Ticagrelor	P2 Receptor	53110	53485	55186	38414	23265	36050	1.02	0.99	1.06	0.90	0.55	0.89	**1.02**	***0.89***	0.87
Docetaxel	Microtubule Associated	14251	14016	14802	10944	11985	10625	0.29	0.27	0.28	0.24	0.26	0.23	**0.28**	**0.24**	0.87
Poziotinib (HM781-36B	EGFR	33534	32365	30289	23596	25055	24556	0.65	0.63	0.57	0.53	0.55	0.55	**0.62**	**0.54**	0.87
GSK2334470	PDK-1	39082	44655	40561	30143	29184	30862	0.77	0.85	0.77	0.69	0.67	0.73	**0.80**	**0.70**	0.87
empty		58229	55723	52757	43076	43912	39432	1.11	1.09	1.00	0.95	0.96	0.88	**1.06**	**0.93**	0.87
Fenoprofen calcium hy(	Others	50520	54118	55234	41417	36523	32457	0.97	1.00	1.06	0.97	0.87	0.80	**1.01**	***0.88***	0.87
Aspirin	COX	49651	51550	53808	37538	41759	33971	0.95	0.95	1.03	0.88	0.99	0.84	**0.98**	***0.86***	0.88
Valdecoxib	COX	54628	56286	54296	42265	176	34160	1.05	1.04	1.04	0.99	0.00	0.84	**1.04**	***0.91***	0.88
PMSF	Cysteine Protease,Serine Protease	50036	52422	54392	37160	36453	35261	0.96	0.97	1.04	0.87	0.86	0.87	**0.99**	***0.87***	0.88
Carbazochrome sodium	Others	53724	61099	51205	42335	34980	34437	1.03	1.13	0.98	0.99	0.83	0.85	**1.05**	***0.92***	0.88
Lumiracoxib	COX	50478	54172	49777	37130	36274	34260	0.97	1.00	0.96	0.87	0.86	0.84	**0.97**	***0.85***	0.88
WZ8040	EGFR	39351	44960	47155	36064	32022	34587	0.81	0.85	0.90	0.80	0.70	0.75	**0.85**	**0.75**	0.88
CCG 50014	Others	48266	51647	48728	33530	37199	35099	0.92	0.95	0.94	0.78	0.88	0.86	**0.94**	***0.82***	0.88
Rivaroxaban	Factor Xa	48846	54837	52897	38437	35036	34018	0.94	1.01	1.02	0.90	0.83	0.84	**0.99**	***0.87***	0.88
Sodium Nitroprusside	Others	49585	56759	57101	41325	171	34394	0.95	1.05	1.10	0.96	0.00	0.85	**1.03**	***0.91***	0.88
(-)-Parthenolide	E3 Ligase	45626	41819	42203	32643	32960	33031	0.87	0.82	0.80	0.72	0.72	0.74	**0.83**	**0.73**	0.88
Tinidazole	Others	53954	57447	59170	43277	198	35811	1.03	1.06	1.14	1.01	0.00	0.88	**1.08**	***0.95***	0.88
Anagrelide HCl	PDE	48303	51985	53469	37321	36920	33883	0.93	0.96	1.03	0.87	0.87	0.83	**0.97**	***0.85***	0.88
IWR-1-endo	Wnt/beta-catenin	41865	43713	40879	29308	30305	32651	0.82	0.83	0.78	0.67	0.70	0.77	**0.81**	**0.71**	0.88
Mepivacaine HCl	Others	53320	53930	53653	39885	35922	34719	1.02	0.99	1.03	0.93	0.85	0.86	**1.02**	***0.89***	0.88
ML133 HCl	Potassium Channel	49416	55990	57338	40399	36088	35160	0.95	1.03	1.10	0.94	0.85	0.87	**1.03**	***0.90***	0.88
Salidroside	Others	52778	52055	49092	40572	38504	38424	1.00	1.02	0.93	0.90	0.84	0.86	**0.98**	**0.87**	0.88
Eltrombopag	Others	51970	53701	57362	40299	38922	40115	1.02	1.02	1.09	0.92	0.89	0.95	**1.04**	**0.92**	0.88
JNJ-1661010	FAAH	56480	57194	54236	41177	37862	36885	1.08	1.05	1.04	0.96	0.90	0.91	**1.06**	***0.93***	0.88
Amidopyrine	Others	52616	57646	51257	40257	46347	34829	1.01	1.06	0.98	0.94	1.10	0.86	**1.02**	***0.90***	0.88
VS-5584 (SB2343)	PI3K	13639	14543	14593	11278	10694	9390	0.27	0.28	0.28	0.26	0.25	0.22	**0.27**	**0.24**	0.88
GDC-0349	mTOR	25086	24474	29059	20193	20968	19131	0.49	0.48	0.55	0.45	0.46	0.43	**0.51**	**0.45**	0.88
GDC-0994	ERK	52996	50029	52744	40757	39318	39533	1.03	0.98	1.00	0.91	0.86	0.88	**1.00**	**0.89**	0.88
(+,-)-Octopamine HCl	Others	54211	54626	59510	43319	38668	35213	1.04	1.01	1.14	1.01	0.92	0.87	**1.06**	***0.94***	0.88
Azlocillin sodium salt	Others	56036	54632	53575	40291	35460	36196	1.07	1.01	1.03	0.94	0.84	0.89	**1.04**	***0.92***	0.88
AZD2014	mTOR	15384	15568	14845	11502	11056	9841	0.29	0.29	0.28	0.27	0.26	0.24	**0.29**	***0.26***	0.88
GDC-0623	MEK	29539	32470	27871	22312	24003	22877	0.58	0.63	0.53	0.50	0.53	0.51	**0.58**	**0.51**	0.88
Sasapyrine	Others	58871	55636	56239	41447	41227	42729	1.16	1.06	1.07	0.95	0.95	1.01	**1.09**	**0.97**	0.88
Sirtinol	Sirtuin	54202	59200	58984	43996	37080	36429	1.04	1.09	1.13	1.03	0.88	0.90	**1.09**	***0.96***	0.88
Levobetaxolol HCl	Adrenergic Receptor	56247	57191	58591	42555	36865	37680	1.08	1.05	1.12	0.99	0.87	0.93	**1.09**	***0.96***	0.88
Magnolol	Others	53054	54380	52360	42084	39618	40799	1.01	1.06	0.99	0.93	0.86	0.91	**1.02**	**0.90**	0.88
Ulixertinib (BVD-523, V	ERK	41053	43672	42737	31583	32992	33555	0.80	0.85	0.81	0.71	0.73	0.75	**0.82**	**0.73**	0.88
AP26113	ALK	30502	33597	33316	24682	22990	23897	0.60	0.64	0.63	0.56	0.53	0.57	**0.62**	**0.55**	0.89

Yohimbine HCl	Adrenergic Receptor	51688	52403	53562	43432	40230	37310	0.98	1.02	1.01	0.96	0.88	0.84	1.01	0.89	0.89
Dydrogesterone	Estrogen/progestogen Receptor	56285	58283	58303	43126	45957	37645	1.08	1.07	1.12	1.01	1.09	0.93	1.09	0.97	0.89
Difluprednate	Others	51247	48851	51275	36967	36139	33756	0.98	0.90	0.98	0.86	0.86	0.83	0.96	0.85	0.89
Halcinonide	Others	49494	51176	53117	40420	35048	31567	0.95	0.94	1.02	0.94	0.83	0.78	0.97	0.86	0.89
Hordenine	Others	56496	54549	54392	44776	41756	40549	1.07	1.07	1.03	0.99	0.91	0.91	1.06	0.94	0.89
Brinzolamide	Carbonic Anhydrase	51451	51270	53078	38688	35792	34176	0.99	0.95	1.02	0.90	0.85	0.84	0.98	0.87	0.89
HS-173	PI3K	25294	24574	21389	17716	18218	19106	0.49	0.48	0.41	0.40	0.40	0.43	0.46	0.41	0.89
Asaraldehyde	COX	57295	54724	52715	44153	41689	40790	1.09	1.07	1.00	0.98	0.91	0.91	1.05	0.93	0.89
Salubrinal	PERK	52022	50952	54155	37175	36807	36272	1.00	0.94	1.04	0.87	0.87	0.89	0.99	0.88	0.89
Coumarin	Others	54412	63300	58420	40376	48938	40458	1.07	1.20	1.11	0.92	1.12	0.96	1.13	1.00	0.89
Alfuzosin HCl	Adrenergic Receptor	50278	54341	56287	43149	43100	40881	1.03	1.03	1.08	0.96	0.94	0.89	1.05	0.93	0.89
Abscisic Acid (Dormin)	Others	54103	56741	47851	38977	41623	42063	1.05	1.11	0.91	0.87	0.92	0.94	1.02	0.91	0.89
Pirfenidone	TGF-beta/Smad	53723	52670	53497	40579	37442	34314	1.03	0.97	1.03	0.95	0.89	0.85	1.01	0.90	0.89
(-)-MK 801 Maleate	GluR	52091	54928	58343	37527	36266	39713	1.00	1.01	1.12	0.87	0.86	0.98	1.04	0.93	0.89
Voreloxin (SNS-595)	Topoisomerase	37114	37686	39318	27878	31356	28928	0.72	0.74	0.75	0.62	0.69	0.64	0.73	0.65	0.89
Febuxostat	Others	60385	63121	57050	46852	44557	44312	1.11	1.19	1.06	1.02	0.96	1.01	1.12	1.00	0.89
SGC-CBP30	Epigenetic Reader Domain	55026	51231	54139	40552	39746	38108	1.08	0.97	1.03	0.93	0.91	0.90	1.03	0.91	0.89
Azaguanine-8	Others	58545	54010	56196	40055	43643	40892	1.15	1.03	1.07	0.91	1.00	0.97	1.08	0.96	0.89
Fluticasone propionate	Glucocorticoid Receptor	51957	47132	52103	36015	39019	38570	0.96	0.89	0.97	0.78	0.84	0.88	0.94	0.83	0.89
MK-8745	Aurora Kinase	20414	18416	17959	14785	14090	13104	0.40	0.35	0.34	0.34	0.32	0.31	0.36	0.32	0.89
CNX-2006	EGFR	46376	48376	47603	36897	35085	33128	0.91	0.92	0.91	0.84	0.81	0.78	0.91	0.81	0.89
Tylosin tartrate	Others	53101	55170	58118	41522	208	36490	1.02	1.02	1.12	0.97	0.00	0.90	1.05	0.93	0.89
NVP-BHG712	Raf,Bcr-Abl,Src,Ephrin receptor	54493	52368	53053	43413	41306	38542	1.04	1.02	1.00	0.96	0.90	0.86	1.02	0.91	0.89
AZD9291	EGFR	50638	52450	47466	37700	39832	33855	0.99	1.00	0.90	0.86	0.92	0.80	0.97	0.86	0.89
Diphenidol HCl	AChR	53885	56088	52417	42466	40013	37719	1.06	1.07	1.00	0.97	0.92	0.89	1.04	0.93	0.89
Amoxicillin	Others	52293	48587	51538	39171	35967	32593	1.00	0.90	0.99	0.91	0.85	0.80	0.96	0.86	0.89
Baricitinib (LY3009104,	JAK	54891	51377	55729	40422	39977	35799	1.05	0.95	1.07	0.94	0.95	0.88	1.02	0.91	0.89
Mequinol	Others	56219	58735	60549	43076	506	39410	1.08	1.08	1.16	1.00	0.01	0.97	1.11	0.99	0.89
Piperacillin Sodium	Others	51424	56898	56605	40372	43638	38230	1.01	1.08	1.08	0.92	1.00	0.90	1.06	0.94	0.89
CH5132799	mTOR,PI3K	46596	46676	46886	36775	37061	34605	0.89	0.91	0.89	0.81	0.81	0.78	0.89	0.80	0.89
Oleuropein	Others	51751	49463	56173	41472	40704	40006	1.01	0.97	1.07	0.93	0.89	0.89	1.01	0.90	0.89
SNX-2112 (PF-0492847	HSP (e.g. HSP90)	16672	18635	16338	14149	12929	12927	0.32	0.36	0.31	0.31	0.28	0.29	0.33	0.29	0.89
MK-1775	Wee1	23307	20484	21008	17668	16544	14819	0.43	0.39	0.39	0.38	0.36	0.34	0.40	0.36	0.89
MK-8776 (SCH 900776)	Chk,CDK	50447	54599	54601	40182	39780	35068	0.97	1.01	1.05	0.94	0.94	0.86	1.01	0.90	0.89
Griseofulvin	Microtubule Associated	54239	62362	52627	41379	165	38255	1.04	1.15	1.01	0.96	0.00	0.94	1.07	0.95	0.89
Aripiprazole	5-HT Receptor	58379	57640	60045	42444	44616	46064	1.08	1.08	1.12	0.92	0.96	1.05	1.09	0.98	0.89
BV-6	IAP	49306	55350	54158	43021	41180	39366	0.96	1.08	1.03	0.96	0.91	0.88	1.02	0.91	0.89
Phloretin	Others	58825	57032	55675	44308	46275	42432	1.12	1.11	1.05	0.98	1.01	0.95	1.10	0.98	0.89
Mefenamic Acid	COX	57118	57390	54468	41970	44615	37725	1.09	1.06	1.05	0.98	1.06	0.93	1.07	0.95	0.89
Promethazine HCl	Histamine Receptor	55773	56031	57728	42580	43357	40109	1.10	1.07	1.10	0.97	1.00	0.95	1.09	0.97	0.89
ABT-737	Bcl-2,Autophagy	39887	43996	43716	28642	36494	36669	0.82	0.83	0.84	0.63	0.80	0.79	0.83	0.74	0.90
Tiratricol	Others	56074	56846	57783	43394	43554	40034	1.10	1.08	1.10	0.99	1.00	0.95	1.09	0.98	0.90
LDE225 (NVP-LDE225,E	Hedgehog/Smoothened	70489	57281	54348	46042	46070	45785	1.30	1.08	1.01	1.00	0.99	1.04	1.13	1.01	0.90
Desonide	Glucocorticoid Receptor	50189	53083	49223	36560	39072	39868	0.93	1.00	0.92	0.80	0.84	0.91	0.95	0.85	0.90
CRT0044876	DNA/RNA Synthesis	53986	53404	50557	38975	43999	40307	1.05	1.04	0.96	0.87	0.97	0.90	1.02	0.91	0.90
Miconazole	Others	53450	52400	52711	43718	41503	38055	1.02	1.02	1.00	0.97	0.90	0.85	1.01	0.91	0.90
JNK-IN-8	JNK	52006	49948	49854	34879	40837	37427	1.02	0.95	0.95	0.80	0.94	0.89	0.97	0.87	0.90
Diclofenac Diethylamin	Others	55448	53311	54806	42771	140	34731	1.06	0.98	1.05	1.00	0.00	0.86	1.03	0.93	0.90
Ethambutol HCl	Others	51542	52253	51963	39779	39903	33964	0.99	0.96	1.00	0.93	0.95	0.84	0.98	0.88	0.90
Golgicide A	ATPase	55378	53760	56536	40415	41340	41722	1.09	1.02	1.08	0.92	0.95	0.99	1.06	0.95	0.90
TCS 359	FLT3	59155	56867	59498	43390	46512	47143	1.15	1.11	1.13	0.97	1.02	1.05	1.13	1.01	0.90
Methenamine	Others	50872	53304	55975	40755	36614	35075	0.97	0.98	1.07	0.95	0.87	0.86	1.01	0.91	0.90
PD98059	MEK	52530	52725	53833	41523	43741	42058	1.08	1.00	1.03	0.92	0.96	0.91	1.04	0.93	0.90
ISRIB (trans-isomer)	PERK	55983	57667	59422	44734	46188	44206	1.09	1.13	1.13	1.00	1.02	0.98	1.11	1.00	0.90
SU6656	Src	45052	44410	69690	42424	41094	40406	0.88	0.87	1.32	0.95	0.90	0.90	1.02	0.92	0.90
Caffeic Acid	Others	58432	51539	52576	44643	42066	39775	1.11	1.01	0.99	0.99	0.92	0.89	1.04	0.93	0.90
Closantel Sodium	Others	56523	54627	55454	41624	40130	37301	1.08	1.01	1.06	0.97	0.95	0.92	1.05	0.94	0.90
Erlotinib HCl (OSI-744)	EGFR,Autophagy	45705	49398	48236	38161	38224	38354	0.94	0.94	0.92	0.85	0.84	0.83	0.93	0.84	0.90
empty		49905	51092	55170	43544	39684	41799	1.02	0.97	1.06	0.97	0.87	0.91	1.02	0.91	0.90
Famciclovir	Others	53247	47279	49834	41224	38954	36897	1.01	0.92	0.94	0.91	0.85	0.83	0.96	0.86	0.90
Sulbactam sodium	Others	54228	54192	53898	44772	40780	40957	1.03	1.06	1.02	0.99	0.89	0.92	1.04	0.93	0.90
Chlorogenic Acid	Others	54549	55345	53026	46803	42793	37488	1.04	1.08	1.00	1.03	0.93	0.84	1.04	0.94	0.90
Canagliflozin	SGLT	48724	55588	55469	39302	41067	36404	0.93	1.02	1.06	0.92	0.97	0.90	1.01	0.91	0.90
Levobupivacaine HCl	Sodium Channel	52155	55486	55209	42270	21453	35042	1.00	1.02	1.06	0.99	0.51	0.86	1.03	0.92	0.90
Wortmannin	PI3K,ATM/ATR,Autophagy	40678	45071	41083	32734	29918	27435	0.78	0.83	0.79	0.76	0.71	0.68	0.80	0.72	0.90
SH-4-54	STAT	41594	43649	54648	36372	36330	36622	0.81	0.85	1.04	0.81	0.80	0.82	0.90	0.81	0.90
Moclobemide (Ro 1111	MAO	49692	49087	52340	36904	37108	34766	0.95	0.90	1.00	0.86	0.88	0.86	0.95	0.86	0.90
P5091 (P005091)	DUB	53914	58012	55856	42182	43414	39818	1.06	1.10	1.06	0.96	1.00	0.94	1.08	0.97	0.90

Nutlin-3	E3 Ligase ,Mdm2	53505	61565	60180	46924	44182	49302	1.10	1.17	1.15	1.04	0.97	1.07	**1.14**	**1.02**	0.90
KN-93 Phosphate	CaMK	57850	53500	59121	44487	43951	45036	1.13	1.04	1.12	0.99	0.97	1.00	**1.10**	**0.99**	0.90
LB42708	Transferase	38985	40891	35639	27853	31717	31039	0.76	0.80	0.68	0.62	0.70	0.69	**0.74**	**0.67**	0.90
BI-D1870	S6 Kinase	40655	40712	44240	31582	30478	28107	0.78	0.75	0.85	0.74	0.72	0.69	**0.79**	***0.71***	0.90
DMSO		52952	51973	53543	37347	38780	37837	1.01	0.96	1.03	0.87	0.92	0.93	**1.00**	***0.90***	0.90
Torcetrapib	CETP	56611	56581	56895	42710	38196	38153	1.08	1.04	1.09	1.00	0.90	0.94	**1.07**	***0.97***	0.90
SNS-314 Mesylate	Aurora Kinase	32476	35284	39361	28606	28304	29062	0.66	0.67	0.75	0.63	0.62	0.63	**0.70**	**0.63**	0.90
Fenticonazole Nitrate	Others	57983	55999	51851	41431	43160	41955	1.07	1.05	0.97	0.90	0.93	0.96	**1.03**	**0.93**	0.90
Penicillin G Sodium	Others	50817	54899	53686	40377	38695	40278	1.00	1.04	1.02	0.92	0.89	0.95	**1.02**	**0.92**	0.90
Bifonazole	Others	52223	53045	53443	42348	38753	40014	0.96	1.00	0.99	0.92	0.83	0.91	**0.99**	**0.89**	0.90
Aclidinium Bromide	AChR	53792	53049	55896	40847	145	36588	1.03	0.98	1.07	0.95	0.00	0.90	**1.03**	***0.93***	0.90
U-104	Carbonic Anhydrase	49904	56133	52513	39268	37734	36082	0.96	1.03	1.01	0.92	0.89	0.89	**1.00**	***0.90***	0.90
AZ 3146	Kinesin	51052	55527	54937	41986	42689	34937	0.98	1.02	1.05	0.98	1.01	0.86	**1.02**	***0.92***	0.90
Praziquantel	Others	55226	53972	56624	41136	42291	43190	1.02	1.01	1.05	0.90	0.91	0.98	**1.03**	**0.93**	0.90
Varespladib (LY315920)	Phospholipase (e.g. PLA)	48507	54655	54118	42438	38981	45044	0.99	1.04	1.04	0.94	0.85	0.98	**1.02**	**0.92**	0.90
Ibrutinib (PCI-32765)	BTK	53247	52097	49562	40451	42864	38001	1.01	1.02	0.94	0.89	0.93	0.85	**0.99**	**0.89**	0.90
Vanillylacetone	Others	54380	54530	50754	42667	40108	42193	1.03	1.06	0.96	0.94	0.87	0.95	**1.02**	**0.92**	0.90
Birinapant	IAP	52248	47081	46834	34775	36049	38851	1.03	0.90	0.89	0.79	0.83	0.92	**0.94**	**0.85**	0.90
Scopolamine HBr	AChR	54864	56622	53477	44498	41336	43316	1.04	1.11	1.01	0.98	0.90	0.97	**1.05**	**0.95**	0.90
Prasugrel	P2 Receptor	45085	57664	53318	42881	41122	41318	0.92	1.09	1.02	0.95	0.90	0.90	**1.01**	**0.91**	0.90
Dalcetrapib (JTT-705, R	CETP	53694	54841	61871	44649	36674	36679	1.03	1.01	1.19	1.04	0.87	0.90	**1.08**	***0.97***	0.90
SANT-1	Hedgehog/Smoothened	49411	54431	58418	41330	39490	40857	0.97	1.04	1.11	0.94	0.91	0.97	**1.04**	**0.94**	0.90
Cyproterone Acetate	Androgen Receptor	54772	56785	58682	45239	43081	41932	1.01	1.07	1.09	0.99	0.93	0.95	**1.06**	**0.96**	0.90
Valganciclovir HCl	Others	53563	53974	54409	40882	125	36366	1.03	0.99	1.04	0.95	0.00	0.90	**1.02**	***0.92***	0.90
Bortezomib (PS-341)	Proteasome	1043	981	1366	890	801	1042	0.02	0.02	0.03	0.02	0.02	0.02	**0.02**	**0.02**	0.90
Nadifloxacin	Others	56976	51077	54847	41484	158	36315	1.09	0.94	1.05	0.97	0.00	0.89	**1.03**	***0.93***	0.90
Flumethasone	Glucocorticoid Receptor	48825	53588	52380	36963	34393	36745	0.94	0.99	1.01	0.86	0.81	0.90	**0.98**	***0.88***	0.90
WAY-600	mTOR	36737	32414	33296	26663	27401	26241	0.70	0.63	0.63	0.59	0.60	0.59	**0.65**	**0.59**	0.90
Dexamethasone (DHAP	IL Receptor,Autophagy	50089	53633	49799	40084	41817	41930	1.03	1.02	0.95	0.89	0.91	0.91	**1.00**	**0.90**	0.90
Skepinone-L	p38 MAPK	50999	53402	52129	39597	39308	38744	1.00	1.02	0.99	0.90	0.90	0.92	**1.00**	**0.91**	0.90
Refametinib (RDEA119,	MEK	45114	46306	47894	35928	37004	39500	0.92	0.88	0.92	0.80	0.81	0.86	**0.91**	**0.82**	0.91
MLN9708	Proteasome	1067	1207	1157	762	910	1019	0.02	0.02	0.02	0.02	0.02	0.02	**0.02**	**0.02**	0.91
Benztropine mesylate	Dopamine Receptor	49795	53027	52461	39855	19172	34291	0.95	0.98	1.01	0.93	0.45	0.84	**0.98**	***0.89***	0.91
Phlorizin	Others	51041	57432	50177	40089	44271	40267	0.97	1.12	0.95	0.89	0.96	0.90	**1.01**	**0.92**	0.91
ML130 (Nodinitib-1)	NOD1	48193	51668	50172	39790	38470	31911	0.92	0.95	0.96	0.93	0.91	0.79	**0.95**	***0.86***	0.91
CHIR-98014	GSK-3	54624	56217	57462	41509	40437	38794	1.05	1.04	1.10	0.97	0.96	0.96	**1.06**	***0.96***	0.91
empty		55155	52757	55606	42312	42313	44170	1.07	1.03	1.05	0.95	0.93	0.98	**1.05**	**0.95**	0.91
HC-030031	Others	52870	51953	46256	38765	35251	33379	1.01	0.96	0.89	0.90	0.83	0.82	**0.95**	***0.86***	0.91
RS-127445	5-HT Receptor	52577	55185	56675	46377	41103	41550	1.00	1.08	1.07	1.03	0.90	0.93	**1.05**	**0.95**	0.91
Clofazimine	Phospholipase (e.g. PLA)	54576	53452	50687	43150	36005	32794	1.05	0.99	0.97	1.01	0.85	0.81	**1.00**	***0.91***	0.91
WYE-125132 (WYE-132	mTOR	14438	12984	12662	10821	10294	10336	0.27	0.25	0.24	0.24	0.22	0.23	**0.26**	**0.23**	0.91
Escitalopram Oxalate	5-HT Receptor	53301	50351	55922	39545	38079	36682	1.02	0.93	1.07	0.92	0.90	0.90	**1.01**	***0.91***	0.91
VU 0357121	GluR	54075	52169	57855	40196	39959	38163	1.04	0.96	1.11	0.94	0.95	0.94	**1.04**	***0.94***	0.91
YH239-EE	Mdm2	55662	52609	51470	40465	43368	42160	1.08	1.03	0.98	0.90	0.95	0.94	**1.03**	**0.93**	0.91
Vandetanib (ZD6474)	VEGFR	48641	52072	54959	41394	42967	41299	1.00	0.99	1.05	0.92	0.94	0.90	**1.01**	**0.92**	0.91
Rotundine	Dopamine Receptor	52600	48482	48587	41784	40943	34883	1.00	0.95	0.92	0.92	0.89	0.78	**0.96**	**0.87**	0.91
Aminothiazole	Others	54601	57583	57444	42711	42385	42572	1.07	1.10	1.09	0.98	0.97	1.01	**1.09**	**0.99**	0.91
Bromfenac Sodium	COX	52577	50539	52613	41509	38594	37252	1.03	0.96	1.00	0.95	0.89	0.88	**1.00**	**0.91**	0.91
Bosutinib (SKI-606)	Src	41793	45656	41685	31883	37135	35366	0.86	0.87	0.80	0.71	0.81	0.77	**0.84**	**0.76**	0.91
Cobicistat (GS-9350)	P450 (e.g. CYP17)	50891	52014	56611	41926	37122	34466	0.97	0.96	1.09	0.98	0.88	0.85	**1.01**	***0.91***	0.91
PP242	mTOR,Autophagy	18499	18310	17589	14330	14543	13893	0.35	0.36	0.33	0.32	0.32	0.31	**0.35**	**0.32**	0.91
XL335	FXR	56683	58174	54426	45858	43304	43919	1.08	1.14	1.03	1.01	0.94	0.98	**1.08**	**0.98**	0.91
Imidapril HCl	RAAS	62946	60579	60995	46015	46210	49268	1.16	1.14	1.14	1.00	0.99	1.12	**1.15**	**1.04**	0.91
Sotrastaurin	PKC	53037	59739	54734	43751	39890	36381	1.02	1.10	1.05	1.02	0.94	0.90	**1.06**	***0.96***	0.91
Adrenalone HCl	Adrenergic Receptor	50134	51757	52027	40204	35633	33485	0.96	0.95	1.00	0.94	0.84	0.82	**0.97**	***0.88***	0.91
Genipin	Others	57619	53812	47830	42693	40761	41766	1.10	1.05	0.91	0.94	0.89	0.94	**1.02**	**0.92**	0.91
Biotin (Vitamin B7)	Others	49326	54674	53952	39263	162	36234	0.94	1.01	1.04	0.92	0.00	0.89	**1.00**	***0.90***	0.91
Olsalazine Sodium	Others	55275	55879	54513	43614	188	35726	1.06	1.03	1.05	1.02	0.00	0.88	**1.05**	***0.95***	0.91
Sotalol	Adrenergic Receptor	53998	50359	56629	44600	43223	38764	1.03	0.98	1.07	0.99	0.94	0.87	**1.03**	**0.93**	0.91
Clarithromycin	P450 (e.g. CYP17)	59260	53190	50724	45282	42180	40887	1.13	1.04	0.96	1.00	0.92	0.92	**1.04**	**0.95**	0.91
Tivantinib (ARQ 197)	c-Met	23546	23670	27078	18525	18542	17045	0.45	0.44	0.52	0.43	0.44	0.42	**0.47**	***0.43***	0.91
Coelenterazine	Others	50977	47435	50797	38048	40752	39055	0.99	0.93	0.96	0.85	0.90	0.87	**0.96**	**0.87**	0.91
Pitavastatin Calcium	Others	42037	49266	48538	15898	46978	44361	0.78	0.93	0.90	0.35	1.01	1.01	**0.87**	**0.79**	0.91
Etofibrate	Others	50394	55619	49540	38542	40780	38023	0.99	1.06	0.94	0.88	0.94	0.90	**1.00**	**0.91**	0.91
Trilostane	Dehydrogenase	52044	55157	57246	42463	45861	44765	1.07	1.05	1.10	0.94	1.00	0.97	**1.07**	**0.97**	0.91
Carbenicillin disodium	Others	51827	54866	55368	41813	41114	35843	0.99	1.01	1.06	0.97	0.97	0.88	**1.02**	***0.93***	0.91
Pacritinib (SB1518)	FLT3,JAK	47444	42004	47368	35109	33555	39397	0.92	0.82	0.90	0.79	0.74	0.88	**0.88**	**0.80**	0.91
Vorapaxar	Protease-activated Receptor	52244	51735	55446	42872	41785	41297	1.02	1.01	1.05	0.96	0.92	0.92	**1.03**	**0.93**	0.91

Tempol	Others	56632	54606	51930	42362	35331	35861	1.08	1.01	1.00	0.99	0.84	0.88	1.03	0.94	0.91
L-Adrenaline	Adrenergic Receptor	55158	53655	53654	44576	41781	41630	1.05	1.05	1.02	0.99	0.91	0.93	1.04	0.94	0.91
Manidipine	Calcium Channel	52869	48290	51829	41138	40379	38975	1.01	0.94	0.98	0.91	0.88	0.87	0.98	0.89	0.91
PF-3845	FAAH	50480	52114	55617	44875	41389	38416	0.96	1.02	1.05	0.99	0.90	0.86	1.01	0.92	0.91
Lorcaserin HCl	5-HT Receptor	55381	53817	53320	40450	39684	37454	1.06	0.99	1.02	0.94	0.94	0.92	1.03	0.93	0.91
Fexofenadine HCl	Histamine Receptor	50100	56730	51705	42495	37965	33618	0.96	1.05	0.99	0.99	0.90	0.83	1.00	0.91	0.91
Dibucaine HCl	Sodium Channel	52281	56680	52281	44330	37773	33162	1.00	1.04	1.00	1.03	0.89	0.82	1.02	0.93	0.91
SKI II	S1P Receptor	51125	49254	47307	36172	40183	35374	1.00	0.94	0.90	0.83	0.92	0.84	0.95	0.86	0.91
empty		53459	57872	55548	44763	44437	46133	1.09	1.10	1.06	0.99	0.97	1.00	1.09	0.99	0.91
N6022	Others	48505	52505	54072	41702	40562	40479	0.95	1.02	1.03	0.93	0.89	0.90	1.00	0.91	0.91
Taxifolin (Dihydroquerc	Others	55254	51280	51841	43673	40786	40426	1.05	1.00	0.98	0.97	0.89	0.91	1.01	0.92	0.91
GS-9973	Syk	46735	43884	42446	35910	34000	35492	0.91	0.86	0.80	0.80	0.75	0.79	0.86	0.78	0.91
AS-604850	PI3K	61369	54971	57413	48167	45128	43725	1.17	1.07	1.09	1.06	0.98	0.98	1.11	1.01	0.91
Cyromazine	Others	57655	57000	54973	42747	44186	41424	1.13	1.08	1.05	0.98	1.02	0.98	1.09	0.99	0.91
Sunitinib Malate	VEGFR,PDGFR,c-Kit	47178	52973	58713	42513	43138	43049	0.97	1.00	1.12	0.94	0.94	0.93	1.03	0.94	0.91
Hematoxylin	Others	49351	52519	52438	38498	41982	41303	0.94	1.03	0.99	0.85	0.91	0.93	0.99	0.90	0.91
Ki16198	LPA Receptor	47431	46759	51301	38304	35158	31675	0.91	0.86	0.98	0.89	0.83	0.78	0.92	0.84	0.91
Xylazine HCl	Adrenergic Receptor	55660	52557	55839	45692	42186	41562	1.06	1.03	1.06	1.01	0.92	0.93	1.05	0.95	0.91
Lomerizine HCl	Calcium Channel	54883	52048	55068	40112	35799	37676	1.05	0.96	1.06	0.94	0.85	0.93	1.02	0.93	0.91
Dofetilide	Potassium Channel	53184	57583	55851	37641	45510	45247	0.98	1.08	1.04	0.82	0.98	1.03	1.03	0.94	0.91
GSK2636771	PI3K	44612	50603	49592	36231	39984	38619	0.87	0.99	0.94	0.81	0.88	0.86	0.93	0.85	0.91
CX-4945 (Silmitasertib)	Casein Kinase	55858	54096	52690	46795	41185	40491	1.06	1.06	1.00	1.03	0.90	0.91	1.04	0.95	0.91
Desvenlafaxine	5-HT Receptor	52978	49748	55288	37871	40557	38010	1.01	0.92	1.06	0.88	0.96	0.94	1.00	0.91	0.91
PP121	PDGFR,mTOR,DNA-PK	18049	19219	19562	14386	14501	15988	0.34	0.38	0.37	0.32	0.32	0.36	0.36	0.33	0.91
PNU-120596	AChR	59087	56182	57688	45200	45515	45895	1.12	1.10	1.09	1.00	0.99	1.03	1.10	1.01	0.91
Afuresertib (GSK21101	Akt	42514	41505	45077	31705	33396	37261	0.83	0.81	0.85	0.71	0.73	0.83	0.83	0.76	0.91
Cephalexin	Others	50704	53697	56039	44978	42688	42658	1.04	1.02	1.07	1.00	0.93	0.92	1.04	0.95	0.91
KY02111	Wnt/beta-catenin	52044	52130	54452	41626	40127	38468	1.02	0.99	1.04	0.95	0.92	0.91	1.02	0.93	0.91
Rofecoxib	COX	46752	49269	51991	34405	34400	36636	0.90	0.91	1.00	0.80	0.81	0.90	0.93	0.85	0.91
Dolutegravir (GSK1349	Integrase	58253	53261	59727	47116	44772	43437	1.11	1.04	1.13	1.04	0.98	0.97	1.09	1.00	0.91
Azelastine HCl	Histamine Receptor	58044	56710	53442	46750	44732	41599	1.10	1.11	1.01	1.03	0.97	0.93	1.07	0.98	0.91
MLN8054	Aurora Kinase	25140	23859	24989	21313	18535	20405	0.51	0.45	0.48	0.47	0.41	0.44	0.48	0.44	0.91
IPI-145 (INK1197)	PI3K	45249	44779	45459	35095	33975	33695	0.89	0.85	0.86	0.80	0.78	0.80	0.87	0.79	0.91
Estradiol valerate	Estrogen/progestogen Receptor	50802	50161	51917	39052	29764	34568	0.97	0.92	1.00	0.91	0.71	0.85	0.96	0.88	0.91
Prednisone	Glucocorticoid Receptor	59519	58954	57892	42024	47324	46844	1.10	1.11	1.08	0.92	1.02	1.07	1.09	1.00	0.91
IKK-16 (IKK Inhibitor VII	IκB/IKK	45579	47600	50782	36283	33611	33017	0.87	0.88	0.97	0.85	0.80	0.81	0.91	0.83	0.91
GSK-LSD1 2HCl	Histone Demethylase	54366	51336	57760	45418	42980	41391	1.06	1.00	1.10	1.02	0.94	0.92	1.05	0.96	0.91
Diminazene Aceturate	Others	52461	53103	49774	39557	38803	35230	1.01	0.98	0.96	0.92	0.92	0.87	0.98	0.89	0.91
Tyrphostin AG 1296	PDGFR,FGFR,c-Kit	60842	59264	57437	48939	49422	42806	1.19	1.16	1.09	1.09	1.09	0.95	1.14	1.04	0.91
Maprotiline HCl	Adrenergic Receptor	52571	54353	52376	44072	41866	40214	1.00	1.06	0.99	0.97	0.91	0.90	1.02	0.93	0.91
GW842166X	Cannabinoid Receptor	49251	52430	54934	41262	38112	34257	0.94	0.97	1.05	0.96	0.90	0.84	0.99	0.90	0.91
Meprednisone	Glucocorticoid Receptor	56770	55910	55374	44220	43583	42149	1.05	1.05	1.03	0.96	0.94	0.96	1.04	0.95	0.91
Efaproxiral Sodium	Others	59668	52610	53968	42526	42810	40966	1.17	1.00	1.03	0.97	0.98	0.97	1.07	0.97	0.91
GSK J4 HCl	Histone Demethylase	47286	45363	47760	35974	37565	33106	0.93	0.86	0.91	0.82	0.86	0.78	0.90	0.82	0.91
SB742457	5-HT Receptor	54399	54443	53703	41442	36502	36874	1.04	1.00	1.03	0.97	0.86	0.91	1.03	0.94	0.91
ME0328	PARP	54956	55449	54593	43179	43047	44954	1.07	1.08	1.04	0.97	0.95	1.00	1.06	0.97	0.91
YM155 (Sepantronium	Survivin	563	539	565	512	437	409	0.01	0.01	0.01	0.01	0.01	0.01	0.01	0.01	0.91
Linagliptin	DPP-4	50984	47078	46508	38081	36808	31672	0.98	0.87	0.89	0.89	0.87	0.78	0.91	0.83	0.91
GW441756	Trk receptor	52370	55130	55627	41646	39687	36968	1.00	1.02	1.07	0.97	0.94	0.91	1.03	0.94	0.91
Pifithrin-α (PFTα)	Autophagy,p53	52363	56782	51396	40853	35576	36461	1.00	1.05	0.99	0.95	0.84	0.90	1.01	0.93	0.91
HTH-01-015	AMPK	53290	49981	54715	41056	40209	38745	1.05	0.95	1.04	0.94	0.92	0.92	1.01	0.93	0.91
SB239063	p38 MAPK	53725	50027	51975	41133	40419	42295	1.05	0.98	0.99	0.92	0.89	0.94	1.00	0.92	0.91
Gliquidone	Potassium Channel	47694	49069	53758	36446	35589	36037	0.91	0.90	1.03	0.85	0.84	0.89	0.95	0.87	0.91
empty		53625	57348	56081	43876	48837	36712	1.03	1.06	1.08	1.02	1.16	0.90	1.05	0.96	0.91
VU 0361737	GluR	51139	53950	54509	39174	37327	37691	0.98	0.99	1.05	0.91	0.88	0.93	1.01	0.92	0.91
Ranolazine 2HCl	Calcium Channel	53293	53008	61264	43662	46621	46308	1.09	1.01	1.17	0.97	1.02	1.00	1.09	1.00	0.91
Acemetacin	COX	53706	55416	52370	42409	42763	42956	1.02	1.08	0.99	0.94	0.93	0.96	1.03	0.94	0.92
Retapamulin	Others	50188	56353	54480	41193	37099	36510	0.96	1.04	1.05	0.96	0.88	0.90	1.02	0.93	0.92
CL-387785 (EKI-785)	EGFR	32157	30107	33283	24935	24996	26157	0.63	0.59	0.63	0.56	0.55	0.58	0.62	0.56	0.92
Sulfaguanidine	Others	58882	58354	55751	46276	44885	40567	1.16	1.11	1.06	1.06	1.03	0.96	1.11	1.02	0.92
Tazarotene	Others	56953	60659	56566	48945	44035	42119	1.05	1.14	1.05	1.07	0.95	0.96	1.08	0.99	0.92
Clomifene citrate	Estrogen/progestogen Receptor	50522	48711	52359	42168	38938	39158	0.96	0.95	0.99	0.93	0.85	0.88	0.97	0.89	0.92
Cathepsin Inhibitor 1	Cysteine Protease	52677	54162	50213	40668	39088	35184	1.01	1.00	0.96	0.95	0.93	0.87	0.99	0.91	0.92
TH287	Others	45578	41279	39869	33059	32801	35178	0.89	0.81	0.76	0.74	0.72	0.78	0.82	0.75	0.92
Cyclo (-RGDfK)	Integrin	51875	51286	56256	42356	40540	44064	1.01	1.00	1.07	0.95	0.89	0.98	1.03	0.94	0.92
TAK-901	Aurora Kinase	17646	15678	17863	12774	10255	11963	0.34	0.29	0.34	0.30	0.24	0.29	0.32	0.30	0.92
AZD2461	PARP	47483	50855	52229	38610	36864	39025	0.93	0.97	0.99	0.88	0.85	0.92	0.96	0.88	0.92
Ornidazole	Others	53015	50668	51488	39033	36719	35923	1.02	0.93	0.99	0.91	0.87	0.88	0.98	0.90	0.92

Tamibarotene	Retinoid Receptor	49540	52510	51745	39905	39418	37725	0.97	1.00	0.98	0.91	0.91	0.89	0.99	0.90	0.92
Methacycline HCl	Others	59088	52480	53424	48331	41633	41003	1.12	1.02	1.01	1.07	0.91	0.92	1.05	0.97	0.92
RITA (NSC 652287)	E3 Ligase ,p53	49597	50657	52379	38678	36497	35063	0.95	0.93	1.01	0.90	0.86	0.86	0.96	0.88	0.92
Nizatidine	Histamine Receptor	56296	55213	53262	44350	41048	42342	1.04	1.04	0.99	0.97	0.88	0.96	1.02	0.94	0.92
PIK-293	PI3K	52478	52923	50364	39215	42358	42154	1.00	1.03	0.95	0.87	0.92	0.95	0.99	0.91	0.92
Diethylstilbestrol	Estrogen/progestogen Receptor	56882	52607	53631	43240	42745	40562	1.05	0.99	1.00	0.94	0.92	0.92	1.01	0.93	0.92
Dirithromycin	Others	58631	53882	51982	40303	42377	42626	1.15	1.03	0.99	0.92	0.97	1.01	1.06	0.97	0.92
Detomidine HCl	Adrenergic Receptor	56759	56157	55074	43824	44892	41672	1.05	1.06	1.03	0.95	0.96	0.95	1.04	0.96	0.92
empty		49810	54859	57384	39145	47010	46198	1.02	1.04	1.10	0.87	1.03	1.00	1.05	0.97	0.92
NMS-873	p97	45236	44295	45223	33921	34908	33807	0.89	0.84	0.86	0.77	0.80	0.80	0.86	0.79	0.92
Bemegride	GABA Receptor	56396	59043	57892	44890	44219	42923	1.11	1.12	1.10	1.02	1.02	1.01	1.11	1.02	0.92
Pergolide mesylate	Dopamine Receptor	51218	51111	53263	38979	40600	36237	0.98	0.94	1.02	0.91	0.96	0.89	0.98	0.90	0.92
Ropivacaine HCl	Others	54801	56140	58654	43185	159	38840	1.05	1.03	1.13	1.01	0.00	0.96	1.07	0.98	0.92
Tadalafil	PDE	53779	60187	58092	45885	47368	47313	1.10	1.14	1.11	1.02	1.04	1.03	1.12	1.03	0.92
SB269970 HCl	5-HT Receptor	52207	56241	51230	40431	42494	36733	1.00	1.04	0.98	0.94	1.01	0.90	1.01	0.92	0.92
Resminostat	HDAC	58558	52213	53227	45158	43504	41700	1.11	1.02	1.01	1.00	0.95	0.94	1.05	0.96	0.92
Adiphenine HCl	Others	61593	58701	56494	46476	45565	45233	1.14	1.10	1.05	1.01	0.98	1.03	1.10	1.01	0.92
Moroxydine HCl	Others	59296	49276	51998	44322	42849	40503	1.13	0.96	0.98	0.98	0.93	0.91	1.02	0.94	0.92
AG-490 (Tyrphostin B42	JAK,EGFR	50696	53672	54401	41604	43344	44979	1.04	1.02	1.04	0.92	0.95	0.97	1.03	0.95	0.92
LY2228820	p38 MAPK	43544	50902	49607	38532	37116	42090	0.89	0.97	0.95	0.85	0.81	0.91	0.94	0.86	0.92
Estradiol Benzoate	Estrogen/progestogen Receptor	51869	54543	53859	40389	37140	37163	0.99	1.01	1.03	0.94	0.88	0.92	1.01	0.93	0.92
Entinostat (MS-275)	HDAC	51350	55858	57326	42041	44198	48386	1.05	1.06	1.10	0.93	0.97	1.05	1.07	0.98	0.92
DMSO		49029	50899	53813	41253	40911	40181	0.93	0.99	1.02	0.91	0.89	0.90	0.98	0.90	0.92
Closantel	Others	54693	56779	58977	44601	46996	38006	1.05	1.05	1.13	1.04	1.11	0.94	1.08	0.99	0.92
Daclatasvir (BMS-7900!	HCV Protease	45384	47793	47253	36122	37694	41191	0.93	0.91	0.91	0.80	0.82	0.89	0.91	0.84	0.92
Pyridoxine HCl	Others	51910	53924	48600	40967	126	33862	0.99	0.99	0.93	0.96	0.00	0.83	0.97	0.89	0.92
Hydroxyzine 2HCl	Histamine Receptor	54554	57383	59884	44821	142	38461	1.05	1.06	1.15	1.04	0.00	0.95	1.08	1.00	0.92
Medroxyprogesterone a	Estrogen/progestogen Receptor	54930	51955	51491	41622	42497	41956	1.04	1.01	0.97	0.92	0.93	0.94	1.01	0.93	0.92
Azomycin	Others	55409	56108	53879	44028	46141	41591	1.05	1.10	1.02	0.97	1.01	0.93	1.06	0.97	0.92
Vidofludimus	Dehydrogenase	55021	58397	51524	40476	44827	40622	1.08	1.11	0.98	0.92	1.03	0.96	1.06	0.97	0.92
Phenylbutazone	Others	56561	59228	54527	45567	44274	42678	1.04	1.11	1.01	0.99	0.95	0.97	1.06	0.97	0.92
TAPI-1	Others	54371	54537	55146	44054	43892	43239	1.06	1.06	1.05	0.99	0.96	0.96	1.06	0.97	0.92
Bezafibrate	PPAR	54542	53858	53867	41214	41398	41323	1.07	1.02	1.02	0.94	0.95	0.98	1.04	0.96	0.92
AST-1306	EGFR	43578	41608	40778	32452	31467	36367	0.83	0.81	0.77	0.72	0.69	0.82	0.80	0.74	0.92
Procainamide HCl	DNA Methyltransferase,Sodium Channel	52871	55063	50408	40520	40693	39741	1.04	1.05	0.96	0.93	0.94	0.94	1.02	0.93	0.92
Rimonabant	Cannabinoid Receptor	47572	51371	53039	38487	39098	35198	0.91	0.95	1.02	0.90	0.93	0.87	0.96	0.88	0.92
Acitretin	Others	52027	55106	53431	43008	46257	42412	1.07	1.04	1.02	0.95	1.01	0.92	1.04	0.96	0.92
(+)-Matrine	Opioid Receptor	55027	51794	52009	42117	41222	43270	1.05	1.01	0.98	0.93	0.90	0.97	1.01	0.93	0.92
ADX-47273	GluR	55207	57815	53036	45229	44727	42585	1.05	1.13	1.00	1.00	0.97	0.95	1.06	0.98	0.92
Miconazole Nitrate	Others	49152	55847	49348	42938	40942	36573	0.91	1.05	0.92	0.94	0.88	0.83	0.96	0.88	0.92
IPA-3	PAK	48409	53651	56695	41083	37682	42497	0.95	1.02	1.08	0.94	0.87	1.00	1.02	0.94	0.92
CYT997 (Lexibulin)	Microtubule Associated	15792	15795	13939	11675	11014	13620	0.30	0.31	0.26	0.26	0.24	0.31	0.29	0.27	0.92
Lacidipine	Calcium Channel	58838	55149	53220	41237	43456	45489	1.09	1.04	0.99	0.90	0.93	1.04	1.04	0.96	0.92
Posaconazole	Others	46945	45131	51606	38897	42954	36132	0.96	0.86	0.99	0.86	0.94	0.78	0.94	0.86	0.92
AMG319	PI3K	49250	46270	49202	40939	37630	37371	0.96	0.90	0.93	0.92	0.83	0.83	0.93	0.86	0.92
Triclabendazole	Microtubule Associated	56847	50521	55098	44699	40232	34448	1.09	0.93	1.06	1.04	0.95	0.85	1.03	0.95	0.92
Phenformin HCl	AMPK	55142	52471	54233	44320	45457	39489	1.05	1.02	1.03	0.98	0.99	0.89	1.03	0.95	0.92
PF-04929113 (SNX-542	HSP (e.g. HSP90)	18438	17494	16732	14793	14458	12821	0.35	0.34	0.32	0.33	0.32	0.29	0.34	0.31	0.92
Tizanidine HCl	Adrenergic Receptor	51111	54203	53173	41444	46290	42456	1.05	1.03	1.02	0.92	1.01	0.92	1.03	0.95	0.92
Entrectinib (RXDX-101)	ALK,Trk receptor	45980	43291	48559	35837	37206	37441	0.90	0.84	0.92	0.80	0.82	0.83	0.89	0.82	0.92
SirReal2	Sirtuin	48279	47874	47391	36768	40445	37950	0.94	0.93	0.90	0.82	0.89	0.85	0.92	0.85	0.92
Vinflunine Tartrate	Microtubule Associated	15682	13487	15783	10813	11684	13351	0.30	0.26	0.30	0.24	0.25	0.30	0.29	0.26	0.92
Telatinib	VEGFR,PDGFR,c-Kit	55392	56913	54078	45213	44808	42955	1.05	1.11	1.02	1.00	0.98	0.96	1.06	0.98	0.92
Pifithrin-µ	p53	46412	48851	47577	36022	35405	33363	0.89	0.90	0.91	0.84	0.84	0.82	0.90	0.83	0.92
Ezetimibe	Others	59887	56906	56322	43519	45527	45954	1.11	1.07	1.05	0.95	0.98	1.05	1.07	0.99	0.92
Epinastine HCl	Histamine Receptor	50350	55186	48840	37317	38826	41989	0.99	1.05	0.93	0.85	0.89	0.99	0.99	0.91	0.92
Bergenin	Others	49666	56828	52230	44361	42351	40240	0.94	1.11	0.99	0.98	0.92	0.90	1.01	0.94	0.92
Semaxanib (SU5416)	VEGFR	51022	53285	54312	42679	43511	34554	0.98	0.98	1.04	1.00	1.03	0.85	1.00	0.92	0.92
DMSO		49919	53669	57652	42142	41650	45007	0.95	1.05	1.09	0.93	0.91	1.01	1.03	0.95	0.92
empty		54869	50427	52594	41663	43279	41775	1.07	0.98	1.00	0.93	0.95	0.93	1.02	0.94	0.92
Linezolid	Others	50290	56016	60535	42883	48127	45979	1.03	1.06	1.16	0.95	1.05	1.00	1.08	1.00	0.92
Sulconazole Nitrate	Others	50992	52503	52126	37524	40544	38024	0.98	0.97	1.00	0.87	0.96	0.94	0.98	0.91	0.92
Tetrahydrozoline HCl	Adrenergic Receptor	48361	52797	55085	41856	979	34219	0.93	0.97	1.06	0.98	0.02	0.84	0.99	0.91	0.92
Azacitidine	DNA Methyltransferase	54621	51620	50937	37419	39238	45752	1.01	0.97	0.95	0.82	0.84	1.04	0.98	0.90	0.92
empty		53871	54784	54884	41132	42940	41234	1.06	1.04	1.04	0.94	0.99	0.98	1.05	0.97	0.92
TG100-115	PI3K	47822	55146	58489	41925	44990	45626	0.98	1.05	1.12	0.93	0.98	0.99	1.05	0.97	0.92
empty		48554	50527	58848	44698	42216	42835	0.99	0.96	1.13	0.99	0.92	0.93	1.03	0.95	0.92
Dexmedetomidine	Adrenergic Receptor	52510	50622	51065	39209	33170	35832	1.01	0.93	0.98	0.91	0.79	0.88	0.97	0.90	0.92

Tofacitinib (CP-690550,	JAK	51805	53132	51617	40984	35962	35247	0.99	0.98	0.99	0.96	0.85	0.87	0.99	0.91	0.92
Tetrahydropapaverine I	Hydroxylase	53941	52674	50655	43765	42433	39680	1.03	1.03	0.96	0.97	0.92	0.89	1.00	0.93	0.92
empty		50253	52772	51571	39051	35547	36157	0.96	0.97	0.99	0.91	0.84	0.89	0.98	0.90	0.92
Histamine 2HCl	Histamine Receptor	52617	53929	55011	42939	38336	35795	1.01	0.99	1.06	1.00	0.91	0.88	1.02	0.94	0.92
P276-00	CDK	33890	31792	28916	26892	24147	25013	0.66	0.62	0.55	0.60	0.53	0.56	0.61	0.56	0.92
3-Deazaneplanocin A (D	Histone Methyltransferase	37034	37842	37882	28561	29882	28093	0.73	0.72	0.72	0.65	0.69	0.66	0.72	0.67	0.92
SL-327	MEK	50811	56910	57227	44631	46303	44747	1.04	1.08	1.10	0.99	1.01	0.97	1.07	0.99	0.92
S- (+)-Rolipram	PDE	60804	56598	56166	48725	44960	42125	1.12	1.06	1.05	1.06	0.97	0.96	1.08	1.00	0.92
Fosaprepitant dimeglur	Others	47236	52170	49594	38899	35327	33706	0.90	0.96	0.95	0.91	0.84	0.83	0.94	0.87	0.92
Elvitegravir (GS-9137, J	Integrase	58058	54823	59700	46774	44803	43454	1.07	1.03	1.11	1.02	0.96	0.99	1.07	0.99	0.92
Sodium Picosulfate	Others	52624	53387	52204	38768	160	38244	1.01	0.98	1.00	0.90	0.00	0.94	1.00	0.92	0.92
Cyclosporine	Others	49724	51392	52211	39592	44409	42386	1.02	0.97	1.00	0.88	0.97	0.92	1.00	0.92	0.92
Aloin	Others	49827	53009	52269	42470	42542	39335	0.95	1.04	0.99	0.94	0.93	0.88	0.99	0.92	0.92
4-Methylumbelliferone	Others	55057	54256	56544	48099	43883	40973	1.05	1.06	1.07	1.06	0.96	0.92	1.06	0.98	0.93
Olmesartan Medoxomi	RAAS	58142	61159	54245	46321	44849	44718	1.07	1.15	1.01	1.01	0.96	1.02	1.08	1.00	0.93
Vorinostat (SAHA, MK0	HDAC,Autophagy	51422	51927	53977	44608	41862	43306	1.05	0.98	1.03	0.99	0.92	0.94	1.02	0.95	0.93
AZD6482	PI3K	48593	46631	49849	36769	42231	40815	0.99	0.88	0.96	0.82	0.92	0.88	0.94	0.87	0.93
Erlotinib	EGFR	47110	42253	47721	35665	35797	38859	0.92	0.82	0.90	0.80	0.79	0.87	0.88	0.82	0.93
Milrinone	ATPase,PDE	57870	53595	55065	46123	43109	44262	1.10	1.05	1.04	1.02	0.94	0.99	1.06	0.98	0.93
Lapatinib (GW-572016)	EGFR,HER2	46185	53613	50317	38889	42328	42494	0.95	1.02	0.96	0.86	0.93	0.92	0.98	0.90	0.93
Cimetidine	Histamine Receptor	55048	56021	53387	42858	43122	42831	1.02	1.05	0.99	0.93	0.93	0.98	1.02	0.95	0.93
KNK437	HSP (e.g. HSP90)	50852	47498	48112	41187	35841	40904	0.99	0.93	0.91	0.92	0.79	0.91	0.94	0.87	0.93
Erythromycin Cyclocarb	Others	52302	54195	50824	41402	43252	42128	1.02	1.06	0.96	0.93	0.95	0.94	1.01	0.94	0.93
Mifepristone	Estrogen/progestogen Receptor	53502	54559	52808	45038	40920	43106	1.02	1.07	1.00	1.00	0.89	0.97	1.03	0.95	0.93
LY2835219	CDK	35527	35703	35437	28438	26597	27023	0.70	0.68	0.67	0.65	0.61	0.64	0.68	0.63	0.93
IMD 0354	IκB/IKK	10598	9653	10579	7350	7991	7687	0.20	0.18	0.20	0.17	0.19	0.19	0.19	0.18	0.93
FT-207 (NSC 148958)	DNA/RNA Synthesis	48546	52706	50487	41259	41198	42805	0.99	1.00	0.97	0.91	0.90	0.93	0.99	0.91	0.93
MM-102	Histone Methyltransferase	57188	56329	53841	46797	42415	39729	1.12	1.07	1.02	1.07	0.97	0.94	1.07	0.99	0.93
Cabazitaxel	Microtubule Associated	16061	14579	15003	11557	12116	10742	0.31	0.27	0.29	0.27	0.29	0.26	0.29	0.27	0.93
VUF 10166	5-HT Receptor	52941	48490	47491	35954	32766	36694	1.01	0.89	0.91	0.84	0.78	0.90	0.94	0.87	0.93
Mesalamine	IκB/IKK	57457	53825	53337	41413	42343	45176	1.06	1.01	0.99	0.90	0.91	1.03	1.02	0.95	0.93
Tolterodine tartrate	AChR	53226	48703	52022	44766	40644	38216	1.01	0.95	0.98	0.99	0.89	0.86	0.98	0.91	0.93
Splitomicin	Sirtuin	52031	49417	53003	42908	41103	40539	1.01	0.96	1.00	0.96	0.90	0.90	0.99	0.92	0.93
Phenylephrine HCl	Adrenergic Receptor	48153	52294	48893	39216	41141	39719	0.92	1.02	0.93	0.87	0.90	0.89	0.95	0.88	0.93
Urapidil HCl	5-HT Receptor	53502	60244	54122	47541	42811	41514	0.99	1.13	1.01	1.04	0.92	0.95	1.04	0.97	0.93
Barasertib (AZD1152-H	Aurora Kinase	41689	38821	42816	33415	35664	32987	0.85	0.74	0.82	0.74	0.78	0.71	0.80	0.75	0.93
Tropicamide	AChR	55346	54576	56188	41246	44456	44611	1.02	1.03	1.05	0.90	0.96	1.02	1.03	0.96	0.93
TH-302	Others	54145	53550	56326	43287	40407	37016	1.04	0.99	1.08	1.01	0.96	0.91	1.04	0.96	0.93
empty		48550	53809	53395	41554	43313	43826	0.99	1.02	1.02	0.92	0.95	0.95	1.01	0.94	0.93
SGI-1027	DNA Methyltransferase	53652	55132	53557	38980	43286	42777	1.05	1.05	1.02	0.89	0.99	1.01	1.04	0.97	0.93
PF-00562271	FAK	56923	52403	51666	41158	47600	40746	1.08	1.02	0.98	0.91	1.04	0.91	1.03	0.95	0.93
TAK-875	GPR	59032	57137	52606	47520	47133	41157	1.12	1.12	1.00	1.05	1.03	0.92	1.08	1.00	0.93
Vitamin B12	Others	56372	52967	53603	44963	41061	41883	1.04	1.00	1.00	0.98	0.88	0.95	1.01	0.94	0.93
VX-765	Caspase	57751	56702	56846	49067	42829	45802	1.10	1.11	1.08	1.08	0.93	1.03	1.09	1.01	0.93
ZM 306416	VEGFR	45577	51395	45739	36671	36863	33084	0.87	0.95	0.88	0.85	0.87	0.81	0.90	0.83	0.93
SB408124	OX Receptor	56504	54457	51950	43407	42978	41578	1.04	1.02	0.97	0.95	0.92	0.95	1.01	0.94	0.93
U73122	Phospholipase (e.g. PLA)	49381	45642	39442	40421	35700	32562	0.96	0.89	0.75	0.90	0.78	0.73	0.87	0.80	0.93
Synephrine HCl	Adrenergic Receptor	53706	49391	54829	46719	40668	39568	1.02	0.96	1.04	1.03	0.89	0.89	1.01	0.94	0.93
CB-839	Others	45674	45476	48213	37486	39055	35986	0.89	0.89	0.91	0.84	0.86	0.80	0.90	0.83	0.93
Captopril	RAAS	62904	55888	59622	49052	45680	45408	1.16	1.05	1.11	1.07	0.98	1.03	1.11	1.03	0.93
FPH2 (BRD-9424)	Others	54119	52810	52590	42560	41285	45005	1.05	1.03	1.00	0.95	0.91	1.00	1.03	0.95	0.93
5-hydroxymethyl Tolter	AChR	58514	53659	57093	50331	42108	43674	1.11	1.05	1.08	1.11	0.92	0.98	1.08	1.00	0.93
3-Methyladenine	PI3K,Autophagy	56482	55567	52331	42200	40141	38265	1.08	1.02	1.00	0.98	0.95	0.94	1.04	0.96	0.93
PD184352 (CI-1040)	MEK	46201	46690	49926	40649	37498	40066	0.95	0.89	0.96	0.90	0.82	0.87	0.93	0.86	0.93
Luliconazole	Others	52777	54481	52034	41415	39035	42387	1.04	1.04	0.99	0.95	0.90	1.00	1.02	0.95	0.93
Nevirapine	Reverse Transcriptase	57104	55036	56662	48085	41320	43223	1.05	1.03	1.05	1.05	0.89	0.98	1.05	0.97	0.93
Naloxone HCl	Opioid Receptor	50275	49597	55267	38407	161	37525	0.96	0.91	1.06	0.90	0.00	0.92	0.98	0.91	0.93
Pheniramine Maleate	Others	52464	53733	55706	40278	41240	38942	1.01	0.99	1.07	0.94	0.98	0.96	1.02	0.95	0.93
A-674563	PKA,Akt,CDK	47950	46837	44522	40337	35271	36522	0.91	0.91	0.84	0.89	0.77	0.82	0.89	0.83	0.93
BIX 01294	Histone Methyltransferase	52123	51487	52674	42374	42266	41654	1.02	1.00	1.00	0.95	0.93	0.93	1.01	0.93	0.93
Favipiravir (T-705)	DNA/RNA Synthesis	55721	52971	52424	42198	45427	42649	1.09	1.03	0.99	0.94	1.00	0.95	1.04	0.96	0.93
PYR-41	E1 Activating	54273	50256	53516	41528	40704	39787	1.07	0.96	1.02	0.95	0.94	0.94	1.01	0.94	0.93
Ampiroxicam	COX	52115	53420	50493	41040	38709	35413	1.00	0.98	0.97	0.96	0.92	0.87	0.98	0.91	0.93
Triptolide (PG490)	Others	12747	12969	11959	10546	9500	7948	0.24	0.24	0.23	0.25	0.23	0.20	0.24	0.22	0.93
CPI-169	Histone Methyltransferase	49590	48766	52884	38863	39626	43725	0.97	0.95	1.00	0.87	0.87	0.97	0.97	0.90	0.93
empty		51897	55788	52315	43491	41444	44438	1.01	1.09	0.99	0.97	0.91	0.99	1.03	0.96	0.93
Milnacipran HCl	Others	46922	57561	54065	41206	39085	36415	0.90	1.06	1.04	0.96	0.93	0.90	1.00	0.93	0.93
Baicalein	P450 (e.g. CYP17)	57930	56959	53583	45613	46094	44037	1.10	1.11	1.01	1.01	1.00	0.99	1.08	1.00	0.93

AZD2858	GSK-3	56216	61785	54568	45550	44452	43245	1.10	1.18	1.04	1.04	1.02	1.02	1.11	1.03	0.93
Progesterone	Estrogen/progestogen Receptor	54610	53235	52570	42981	39941	43151	1.01	1.00	0.98	0.94	0.86	0.98	1.00	0.93	0.93
Ethacridine lactate mor	Others	52308	48524	48193	40999	38368	35856	1.03	0.92	0.92	0.94	0.88	0.85	0.96	0.89	0.93
empty		54899	54775	53660	41176	46139	38829	1.05	1.01	1.03	0.96	1.09	0.96	1.03	0.96	0.93
Tanshinone IIA	Others	58592	55521	51989	47829	44730	41322	1.11	1.08	0.98	1.06	0.97	0.93	1.06	0.99	0.93
Decitabine	DNA Methyltransferase	45781	43625	49002	38244	38358	38184	0.94	0.83	0.94	0.85	0.84	0.83	0.90	0.84	0.93
Dacarbazine	DNA/RNA Synthesis	47885	54383	50502	40680	43164	42706	0.98	1.03	0.97	0.90	0.94	0.93	0.99	0.92	0.93
empty		51114	50519	50075	39831	41846	41158	1.00	0.99	0.95	0.89	0.92	0.92	0.98	0.91	0.93
Suvorexant (MK-4305)	OX Receptor	51368	53206	58612	41435	43059	41558	1.01	1.01	1.12	0.95	0.99	0.98	1.05	0.97	0.93
PHA-665752	c-Met	52190	49284	56165	46460	41955	42356	1.07	0.93	1.08	1.03	0.92	0.92	1.03	0.95	0.93
NXY-059	Others	55211	47160	49057	40686	38605	37910	1.08	0.90	0.93	0.93	0.89	0.90	0.97	0.90	0.93
PF-562271	FAK	48467	51048	48967	39249	36748	33618	0.93	0.94	0.94	0.92	0.87	0.83	0.94	0.87	0.93
Quinacrine 2HCl	Others	52612	51705	53301	38699	43115	40036	1.03	0.98	1.01	0.88	0.99	0.95	1.01	0.94	0.93
Rotigotine	Dopamine Receptor	56825	56749	57554	45182	45446	41748	1.12	1.08	1.10	1.03	1.04	0.99	1.10	1.02	0.93
Nefiracetam	GABA Receptor	59689	54959	55016	45705	42824	45007	1.10	1.03	1.02	1.00	0.92	1.02	1.05	0.98	0.93
Acarbose	Others	50158	54183	52911	45058	41638	43687	1.03	1.03	1.01	1.00	0.91	0.95	1.02	0.95	0.93
Nutlin-3a	Mdm2	56270	50259	52146	41303	43100	44143	1.10	0.98	0.99	0.92	0.95	0.98	1.02	0.95	0.93
Clindamycin HCl	Others	56711	53301	47123	41900	44752	40245	1.08	1.04	0.89	0.93	0.98	0.90	1.00	0.93	0.93
PFI-4	Epigenetic Reader Domain	53974	51382	51715	40495	45756	41088	1.05	1.00	0.98	0.91	1.01	0.92	1.01	0.94	0.93
Naphazoline HCl	Adrenergic Receptor	53002	53568	52722	44476	41987	42102	1.01	1.05	1.00	0.98	0.91	0.94	1.02	0.95	0.93
Zileuton	Others	51706	59571	55445	44628	48133	45520	1.06	1.13	1.06	0.99	1.05	0.99	1.08	1.01	0.93
Myricitrin	PKC	51840	51333	53688	44756	39000	42793	0.99	1.00	1.02	0.99	0.85	0.96	1.00	0.93	0.93
BMS-777607	c-Met,TAM Receptor	54998	56965	57225	43515	46145	43791	1.02	1.07	1.07	0.95	0.99	1.00	1.05	0.98	0.93
ORY-1001 (RG-6016)	Histone Demethylase	49730	48869	49144	40484	38507	40803	0.97	0.95	0.93	0.91	0.85	0.91	0.95	0.89	0.93
Y-320	Others	53642	48846	54530	42738	40837	43687	1.05	0.95	1.03	0.96	0.90	0.97	1.01	0.94	0.93
Chlorpropamide	Others	55445	58874	60134	47874	44450	42787	1.09	1.12	1.14	1.09	1.02	1.01	1.12	1.04	0.93
SB525334	TGF-beta/Smad	53264	54317	59802	47516	45523	45992	1.09	1.03	1.15	1.05	1.00	1.00	1.09	1.02	0.93
Rosiglitazone maleate	PPAR	55239	53874	54804	43558	44518	44335	1.05	1.05	1.04	0.96	0.97	0.99	1.05	0.98	0.93
Naringin	P450 (e.g. CYP17)	54576	50103	52188	46335	40838	39530	1.04	0.98	0.99	1.02	0.89	0.89	1.00	0.93	0.93
RG108	Transferase,DNA Methyltransferase	48787	50052	49094	38523	37214	34198	0.93	0.92	0.94	0.90	0.88	0.84	0.93	0.87	0.93
Secnidazole	Others	53492	56599	53591	47022	44796	40549	1.02	1.11	1.01	1.04	0.98	0.91	1.05	0.97	0.93
RN486	BTK	52681	49912	50409	40754	41048	36873	1.03	0.95	0.96	0.93	0.94	0.87	0.98	0.92	0.93
BMS-345541	IκB/IKK	59506	54003	58271	42614	48948	47893	1.16	1.05	1.10	0.95	1.08	1.07	1.11	1.03	0.93
Go 6983	PKC	49691	54156	48677	40254	34382	34758	0.95	1.00	0.93	0.94	0.81	0.86	0.96	0.90	0.93
AZD1208	Pim	50182	48496	41848	37690	37669	33734	0.99	0.92	0.80	0.86	0.87	0.80	0.90	0.84	0.93
WZ3146	EGFR	30855	34415	35011	26824	28103	28402	0.63	0.65	0.67	0.59	0.61	0.62	0.65	0.61	0.93
Moexipril HCl	RAAS	60170	56110	53026	45628	43903	44129	1.11	1.05	0.99	0.99	0.94	1.00	1.05	0.98	0.93
Adenine HCl	DNA/RNA Synthesis	55015	53549	56040	43024	42894	44015	1.02	1.01	1.04	0.94	0.92	1.00	1.02	0.95	0.93
Ketotifen Fumarate	Histamine Receptor	54718	51404	50491	41471	40870	41315	1.01	0.97	0.94	0.90	0.88	0.94	0.97	0.91	0.93
PI-3065	PI3K	47270	50360	39977	37065	36199	38626	0.92	0.98	0.76	0.83	0.80	0.86	0.89	0.83	0.93
Tiopronin	Others	58032	54204	55917	45695	44839	42350	1.07	1.02	1.04	1.00	0.96	0.96	1.04	0.97	0.93
Oxfendazole	Others	54770	49057	54865	39761	42418	43089	1.01	0.92	1.02	0.87	0.91	0.98	0.98	0.92	0.93
Exemestane	Aromatase	48900	51830	54549	38296	44727	46251	1.00	0.98	1.05	0.85	0.98	1.00	1.01	0.94	0.93
Zolmitriptan	5-HT Receptor	58247	56745	56131	46114	44995	44140	1.08	1.07	1.04	1.00	0.97	1.00	1.06	0.99	0.93
SC75741	NF-κB	52674	51871	55800	43182	44802	36553	1.03	0.99	1.06	0.99	1.03	0.86	1.03	0.96	0.93
Dyclonine HCl	Others	58393	58480	55534	48437	44529	43391	1.08	1.10	1.03	1.06	0.96	0.99	1.07	1.00	0.93
CO-1686 (AVL-301)	EGFR	47138	46038	52229	36503	37164	39093	0.93	0.88	0.99	0.83	0.85	0.92	0.93	0.87	0.93
LY2157299	TGF-beta/Smad	57359	56972	57610	50408	45376	43441	1.09	1.11	1.09	1.11	0.99	0.97	1.10	1.03	0.93
Clindamycin	Others	50929	52395	50590	39983	36738	35831	0.98	0.97	0.97	0.93	0.87	0.88	0.97	0.91	0.93
Itraconazole	P450 (e.g. CYP17)	52975	48775	50518	43616	41167	38525	1.01	0.95	0.96	0.96	0.90	0.86	0.97	0.91	0.93
IM-12	GSK-3	50699	43904	49926	40096	35611	41760	0.99	0.86	0.95	0.90	0.78	0.93	0.93	0.87	0.93
EPZ004777	Histone Methyltransferase	48812	52909	50064	42971	38439	42030	0.95	1.03	0.95	0.96	0.85	0.94	0.98	0.91	0.93
Batimastat (BB-94)	MMP	51606	50756	53298	41378	40825	38736	1.01	0.97	1.01	0.94	0.94	0.92	1.00	0.93	0.94
SRT2104 (GSK2245840)	Sirtuin	51386	47062	50670	39901	38507	42861	1.00	0.92	0.96	0.89	0.85	0.95	0.96	0.90	0.94
Iniparib (BSI-201)	PARP	50065	54457	60400	44004	45275	48047	1.02	1.03	1.16	0.98	0.99	1.04	1.07	1.00	0.94
DMSO		53017	61254	55066	44641	42872	38802	1.02	1.13	1.06	1.04	1.02	0.96	1.07	1.00	0.94
DMSO		52725	62665	54482	45470	49774	46188	1.08	1.19	1.04	1.01	1.09	1.00	1.10	1.03	0.94
Fisetin	Sirtuin	53515	52350	49840	42168	43132	40933	1.02	1.02	0.94	0.93	0.94	0.92	0.99	0.93	0.94
A66	PI3K	56356	49807	52378	45465	41410	41552	1.07	0.97	0.99	1.01	0.90	0.93	1.01	0.95	0.94
Tiplaxtinin (PAI-039)	Others	53400	51521	50465	45123	39839	41497	1.04	1.01	0.96	1.01	0.88	0.92	1.00	0.94	0.94
Loxapine Succinate	5-HT Receptor,Dopamine Receptor	53856	56490	55893	45293	8961	36830	1.03	1.04	1.07	1.06	0.21	0.91	1.05	0.98	0.94
Riluzole	Sodium Channel,GluR	64504	55219	57093	47451	45451	47009	1.19	1.04	1.06	1.03	0.98	1.07	1.10	1.03	0.94
Cisatracurium Besylate	Adrenergic Receptor	57977	55342	58901	44848	44990	46489	1.07	1.04	1.10	0.98	0.97	1.06	1.07	1.00	0.94
Primidone	Sodium Channel	59360	55834	55618	45581	43651	45984	1.10	1.05	1.04	0.99	0.94	1.05	1.06	0.99	0.94
Alvelestat (AZD9668)	Serine Protease	52397	55470	52907	39203	42389	43354	1.03	1.06	1.01	0.90	0.97	1.03	1.03	0.96	0.94
Epinephrine HCl	Adrenergic Receptor	50996	49681	48083	38297	38908	35123	0.98	0.92	0.92	0.89	0.92	0.86	0.94	0.88	0.94
Xanthone	Others	55541	54388	50787	44489	44199	41813	1.06	1.06	0.96	0.98	0.96	0.94	1.03	0.96	0.94
MK-0752	Gamma-secretase,Beta Amyloid	58839	55991	53603	49388	45759	41639	1.12	1.09	1.01	1.09	1.00	0.93	1.08	1.01	0.94

Bindarit	Others	50440	48621	50461	39353	37197	34548	0.97	0.90	0.97	0.92	0.88	0.85	**0.94**	***0.88***	0.94
Olopatadine HCl	Histamine Receptor	56969	50953	51532	46081	42154	41179	1.08	1.00	0.98	1.02	0.92	0.92	**1.02**	**0.95**	0.94
DL-Carnitine HCl	Others	55869	51325	54740	43405	43034	44942	1.06	1.00	1.04	0.96	0.94	1.01	**1.03**	**0.97**	0.94
Thiamet G	Others	55668	56184	51383	42046	44246	40819	1.09	1.07	0.98	0.96	1.02	0.97	**1.05**	**0.98**	0.94
empty		52104	51806	51528	43753	41924	41062	1.02	1.01	0.98	0.98	0.92	0.91	**1.00**	**0.94**	0.94
Ranitidine	Histamine Receptor	56042	56542	54587	44328	45286	43020	1.03	1.06	1.02	0.97	0.97	0.98	**1.04**	**0.97**	0.94
Isoprenaline HCl	Adrenergic Receptor	51642	51877	49619	41288	42395	40742	0.98	1.01	0.94	0.91	0.92	0.91	**0.98**	**0.92**	0.94
PF-3758309	PAK	26582	28641	27729	22362	22840	19421	0.52	0.54	0.53	0.51	0.52	0.46	**0.53**	**0.50**	0.94
Imatinib (STI571)	PDGFR	53116	53828	52518	46534	41250	41749	1.01	1.05	0.99	1.03	0.90	0.94	**1.02**	**0.95**	0.94
T0901317	Liver X Receptor	47713	50287	48544	34690	40688	38655	0.94	0.96	0.92	0.79	0.93	0.91	**0.94**	**0.88**	0.94
Liproxstatin-1	Ferroptosis	47482	50384	51752	41332	39077	41570	0.93	0.98	0.98	0.92	0.86	0.93	**0.96**	**0.90**	0.94
Biochanin A	FAAH	52873	54038	51808	43158	43244	42582	1.01	1.06	0.98	0.95	0.94	0.95	**1.01**	**0.95**	0.94
SC-514	IκB/IKK	55688	53298	52349	42023	41873	41800	1.09	1.01	1.00	0.96	0.96	0.99	**1.03**	**0.97**	0.94
Oltipraz	Others	48080	48182	52572	39898	38707	42743	0.94	0.94	1.00	0.89	0.85	0.95	**0.96**	**0.90**	0.94
Antipyrine	Others	51241	52155	51323	42044	35414	34557	0.98	0.96	0.99	0.98	0.84	0.85	**0.98**	***0.92***	0.94
Domiphen Bromide	Others	53436	52068	51256	41838	40834	39545	1.05	0.99	0.98	0.96	0.94	0.94	**1.01**	**0.94**	0.94
Safinamide Mesylate	MAO	50677	54207	57921	42576	46783	46699	1.04	1.03	1.11	0.94	1.02	1.01	**1.06**	**0.99**	0.94
BMS-754807	IGF-1R,Trk receptor,c-Met	36881	37897	37955	30561	32925	30806	0.76	0.72	0.73	0.68	0.72	0.67	**0.73**	**0.69**	0.94
Benidipine HCl	Calcium Channel	55766	54264	53616	44687	40866	44257	1.03	1.02	1.00	0.97	0.88	1.01	**1.02**	**0.95**	0.94
WY-14643 (Pirinixic Aci	PPAR	53380	52713	56364	43882	45392	43307	1.04	1.03	1.07	0.98	1.00	0.96	**1.05**	**0.98**	0.94
Marimastat(BB-2516)	MMP	55073	54179	53890	44437	41659	41117	1.08	1.03	1.03	1.01	0.96	0.97	**1.05**	**0.98**	0.94
GDC-0941	PI3K	46245	46244	49083	46784	36971	34534	0.95	0.88	0.94	1.04	0.81	0.75	**0.92**	**0.86**	0.94
Spautin-1	Autophagy	49239	43301	46917	40972	37278	35571	0.96	0.85	0.89	0.92	0.82	0.79	**0.90**	**0.84**	0.94
PIK-294	PI3K	52876	55313	51938	48889	42554	38864	1.01	1.08	0.98	1.08	0.93	0.87	**1.02**	**0.96**	0.94
Tasisulam	Caspase	56454	53918	57292	45297	43635	41840	1.11	1.03	1.09	1.03	1.00	0.99	**1.07**	**1.01**	0.94
empty		53861	54718	52958	44434	42656	44824	1.05	1.07	1.00	0.99	0.94	1.00	**1.04**	**0.98**	0.94
Eltrombopag Olamine	Others	53182	52904	52654	45290	39684	44070	1.01	1.03	1.00	1.00	0.86	0.99	**1.01**	**0.95**	0.94
Bazedoxifene HCl	Estrogen/progestogen Receptor	58649	52968	58598	45051	45545	44580	1.08	1.00	1.09	0.98	0.98	1.01	**1.06**	**0.99**	0.94
empty		57347	53700	53297	41181	45818	43540	1.06	1.01	0.99	0.90	0.98	0.99	**1.02**	**0.96**	0.94
Dapagliflozin	SGLT	57989	60035	56409	48612	46121	43932	1.07	1.13	1.05	1.06	0.99	1.00	**1.08**	**1.02**	0.94
Dicoumarol	Others	54414	55325	52222	40921	42453	42910	1.07	1.05	0.99	0.93	0.98	1.01	**1.04**	**0.97**	0.94
Oxybutynin chloride	AChR	50197	49050	48492	38780	39030	34385	0.96	0.90	0.93	0.90	0.92	0.85	**0.93**	***0.88***	0.94
empty		53764	51425	49667	42484	44626	38985	1.02	1.00	0.94	0.94	0.97	0.87	**0.99**	**0.93**	0.94
Bepotastine Besilate	Histamine Receptor	49328	53490	51311	40968	42916	35355	0.95	0.99	0.99	0.96	1.02	0.87	**0.97**	***0.91***	0.94
Emtricitabine	Reverse Transcriptase	57784	55137	56130	44575	44969	44783	1.07	1.04	1.04	0.97	0.97	1.02	**1.05**	**0.99**	0.94
Rasagiline Mesylate	MAO	56275	56973	58289	47213	42345	46672	1.04	1.07	1.08	1.03	0.91	1.06	**1.06**	**1.00**	0.94
Pelitinib (EKB-569)	EGFR	37828	41312	42905	32517	36494	33082	0.77	0.78	0.82	0.72	0.80	0.72	**0.79**	**0.75**	0.94
DMSO		56254	58591	57770	47170	45028	45026	1.04	1.10	1.08	1.03	0.97	1.02	**1.07**	**1.01**	0.94
BPTES	Others	51213	49841	47970	42050	38897	40860	1.00	0.97	0.91	0.94	0.86	0.91	**0.96**	**0.90**	0.94
Genistein	EGFR,Topoisomerase	47446	52306	53675	42554	42491	43312	0.97	0.99	1.03	0.94	0.93	0.94	**1.00**	**0.94**	0.94
Tioxolone	Carbonic Anhydrase	58223	52682	57097	48992	44797	42945	1.11	1.03	1.08	1.08	0.98	0.96	**1.07**	**1.01**	0.94
Acetanilide	Others	53035	47969	49635	45656	39811	37176	1.01	0.94	0.94	1.01	0.87	0.83	**0.96**	**0.90**	0.94
BX-795	IκB/IKK,PDK-1	35598	36435	35605	29449	29835	30916	0.73	0.69	0.68	0.65	0.65	0.67	**0.70**	**0.66**	0.94
Nafcillin Sodium	Others	49068	54023	55213	42373	137	36095	0.94	1.00	1.06	0.99	0.00	0.89	**1.00**	***0.94***	0.94
MLN2238	Proteasome	782	793	1208	560	984	722	0.01	0.02	0.02	0.01	0.02	0.02	**0.02**	**0.02**	0.94
Go6976	JAK,FLT3,PKC	51109	48769	49843	39697	38873	38358	1.00	0.93	0.95	0.91	0.89	0.91	**0.96**	**0.90**	0.94
Naproxen	COX	59130	58562	59097	46238	47469	46871	1.09	1.10	1.10	1.01	1.02	1.07	**1.10**	**1.03**	0.94
DMSO		50733	48840	50451	41408	41821	39444	0.99	0.95	0.96	0.93	0.92	0.88	**0.97**	**0.91**	0.94
L-685,458	Gamma-secretase	52180	50928	47902	42042	40860	40630	1.02	0.99	0.91	0.94	0.90	0.91	**0.97**	**0.91**	0.94
Esculin	Others	56771	54592	54647	47357	45285	42588	1.08	1.07	1.03	1.05	0.99	0.95	**1.06**	**1.00**	0.94
ABT-751 (E7010)	Microtubule Associated	21234	24086	23874	18252	17537	22145	0.43	0.46	0.46	0.40	0.38	0.48	**0.45**	**0.42**	0.94
Tolnaftate	Others	55469	57226	58113	45446	46468	44019	1.02	1.08	1.08	0.99	1.00	1.00	**1.06**	**1.00**	0.94
Alizarin	P450 (e.g. CYP17)	53791	47406	54408	41896	46507	38391	1.02	0.93	1.03	0.93	1.01	0.86	**0.99**	**0.93**	0.94
SBE 13 HCl	PLK	49610	49635	47652	41187	37821	41168	0.97	0.97	0.90	0.92	0.83	0.92	**0.95**	**0.89**	0.94
empty		54187	49879	52126	41020	42256	44513	1.06	0.97	0.99	0.92	0.93	0.99	**1.01**	**0.95**	0.94
WP1066	JAK	51262	53326	52897	41239	38787	36838	0.98	0.98	1.02	0.96	0.92	0.91	**0.99**	***0.93***	0.94
Homatropine Methylbrc	AChR	52565	52660	48746	40286	40180	36035	1.01	0.97	0.94	0.94	0.95	0.89	**0.97**	***0.91***	0.94
Y-27632 2HCl	ROCK,Autophagy	54684	56048	60635	48055	50125	45399	1.12	1.06	1.16	1.07	1.10	0.98	**1.11**	**1.05**	0.94
NU7441 (KU-57788)	DNA-PK,PI3K	54078	52845	51471	43776	43713	41627	1.03	1.03	0.97	0.97	0.95	0.93	**1.01**	**0.95**	0.94
Laetrile	Others	55744	57267	58204	47666	46703	45187	1.06	1.12	1.10	1.05	1.02	1.01	**1.09**	**1.03**	0.94
Lomeguatrib	DNA Methyltransferase	55493	52968	49214	43145	43049	42910	1.08	1.03	0.93	0.96	0.95	0.96	**1.02**	**0.96**	0.94
Ziprasidone HCl	5-HT Receptor,Dopamine Receptor	50260	50355	55564	43195	44331	43411	1.03	0.95	1.06	0.96	0.97	0.94	**1.02**	**0.96**	0.94
Acebutolol HCl	Adrenergic Receptor	49609	54065	53177	42031	38452	35786	0.95	1.00	1.02	0.98	0.91	0.88	**0.99**	***0.93***	0.94
Anisomycin	JNK	11997	12392	11940	9446	10453	9849	0.23	0.24	0.23	0.21	0.23	0.22	**0.23**	**0.22**	0.94
Tubacin	HDAC	55986	54130	53492	46153	43627	43588	1.06	1.06	1.01	1.02	0.95	0.98	**1.04**	**0.98**	0.94
GW5074	Raf	50297	52454	52264	41192	37509	35729	0.96	0.97	1.00	0.96	0.89	0.88	**0.98**	***0.92***	0.94
Zoxazolamine	Others	50922	53117	53148	43132	37622	42053	1.00	1.01	1.01	0.98	0.86	0.99	**1.01**	**0.95**	0.94
Licochalcone A	Estrogen/progestogen Receptor	48061	51617	52833	40207	43230	41423	0.94	1.01	1.00	0.90	0.95	0.92	**0.98**	**0.92**	0.94

Amprenavir	HIV Protease	56396	53279	59457	48773	38960	46752	1.04	1.00	1.11	1.06	0.84	1.06	**1.05**	**0.99**	0.94
Mezlocillin Sodium	Others	53279	53328	59108	45459	42236	41862	1.05	1.01	1.12	1.04	0.97	0.99	**1.06**	**1.00**	0.94
PIK-75	DNA-PK,PI3K	46885	51385	49067	41993	41004	40520	0.96	0.97	0.94	0.93	0.90	0.88	**0.96**	**0.90**	0.94
empty		52073	54095	51795	42026	40833	36378	1.00	1.00	0.99	0.98	0.97	0.90	**1.00**	***0.94***	0.94
Dexlansoprazole	Proton Pump	52380	56443	54438	42821	38247	38187	1.00	1.04	1.05	1.00	0.91	0.94	**1.03**	***0.97***	0.94
Loratadine	Histamine Receptor	50170	56279	56250	46147	43425	46807	1.03	1.07	1.08	1.02	0.95	1.01	**1.06**	**1.00**	0.94
Chlorhexidine HCl	Others	48600	48406	52286	39368	186	34792	0.93	0.89	1.00	0.92	0.00	0.86	**0.94**	***0.89***	0.94
LY294002	Autophagy,PI3K	46786	48857	54935	42385	40418	43479	0.96	0.93	1.05	0.94	0.88	0.94	**0.98**	**0.92**	0.94
Fenofibrate	PPAR	59116	55616	54761	44563	46212	44281	1.09	1.05	1.02	0.97	0.99	1.01	**1.05**	**0.99**	0.94
Apatinib	VEGFR	55183	54294	55186	46883	46821	40752	1.05	1.06	1.04	1.04	1.02	0.91	**1.05**	**0.99**	0.94
MK-2866 (GTx-024)	Androgen Receptor	52476	54464	54443	43478	47536	44494	1.07	1.03	1.04	0.96	1.04	0.96	**1.05**	**0.99**	0.94
Flumequine	Topoisomerase	51490	51241	50990	39331	40849	36966	0.99	0.94	0.98	0.92	0.97	0.91	**0.97**	***0.91***	0.94
empty		49419	65773	51875	43458	42659	44421	0.97	1.25	0.99	0.99	0.98	1.05	**1.07**	**1.01**	0.94
Bacitracin	Others	51701	52866	54530	42307	40163	41978	1.02	1.01	1.04	0.97	0.92	0.99	**1.02**	**0.96**	0.94
Bosentan	Endothelin Receptor	50524	53778	53189	39783	42972	40447	0.99	1.02	1.01	0.91	0.99	0.96	**1.01**	**0.95**	0.94
Adenosine	Others	59197	57434	57078	46434	45191	46790	1.09	1.08	1.06	1.01	0.97	1.07	**1.08**	**1.02**	0.94
Uridine	DNA/RNA Synthesis	55725	56614	53504	44796	43384	44033	1.03	1.06	1.00	0.98	0.93	1.00	**1.03**	**0.97**	0.94
Fesoterodine Fumarate	AChR	58759	52604	55715	45778	46519	44090	1.12	1.03	1.05	1.01	1.01	0.99	**1.07**	**1.00**	0.94
Pioglitazone HCl	P450 (e.g. CYP17)	56560	62137	57462	46911	48536	45136	1.04	1.17	1.07	1.02	1.04	1.03	**1.09**	**1.03**	0.94
AEE788 (NVP-AEE788)	VEGFR,EGFR,HER2	51117	48749	50023	39477	48374	38198	1.05	0.92	0.96	0.88	1.06	0.83	**0.98**	**0.92**	0.94
PF-03084014 (PF-3084(	Gamma-secretase	53477	50471	49334	42478	37623	45549	1.04	0.98	0.94	0.95	0.83	1.01	**0.99**	**0.93**	0.94
Empagliflozin (BI 1077:	SGLT	54391	52032	53739	42610	42831	45858	1.06	1.02	1.02	0.95	0.94	1.02	**1.03**	**0.97**	0.94
Rosiglitazone	PPAR	51090	50686	51289	40043	43646	41345	0.97	0.99	0.97	0.89	0.95	0.93	**0.98**	**0.92**	0.94
Crenolanib (CP-868596)	PDGFR	58543	55385	59363	45829	42011	40361	1.12	1.02	1.14	1.07	1.00	0.99	**1.09**	***1.03***	0.94
Doxazosin Mesylate	Adrenergic Receptor	47118	50468	48181	37877	40834	43796	0.96	0.96	0.92	0.84	0.89	0.95	**0.95**	**0.89**	0.94
Telmisartan	RAAS	61551	55434	56917	47251	48423	43140	1.14	1.04	1.06	1.03	1.04	0.98	**1.08**	**1.02**	0.94
Suprofen	Others,COX	58634	54400	53698	39428	44752	48579	1.08	1.02	1.00	0.86	0.96	1.11	**1.03**	**0.98**	0.94
Flunixin Meglumin	COX	54987	60876	54333	47657	44735	43461	1.02	1.14	1.01	1.04	0.96	0.99	**1.06**	**1.00**	0.94
Meclofenamate Sodiun	COX	52004	51430	51394	42055	41519	37768	1.02	0.98	0.98	0.96	0.95	0.89	**0.99**	**0.94**	0.94
VX-661	CFTR	48223	55049	50620	41770	39249	39448	0.95	1.05	0.96	0.95	0.90	0.93	**0.99**	**0.93**	0.94
WIKI4	Wnt/beta-catenin	44789	44211	42575	35670	36345	35946	0.87	0.86	0.81	0.80	0.80	0.80	**0.85**	**0.80**	0.94
Tariquidar	P-gp	58674	52283	56515	46033	47169	44173	1.14	1.02	1.07	1.03	1.04	0.98	**1.08**	**1.02**	0.94
empty		56182	51855	56245	44423	45134	45181	1.09	1.01	1.07	0.99	0.99	1.01	**1.06**	**1.00**	0.94
Olanzapine	5-HT Receptor,Dopamine Receptor	52627	53180	53191	49510	42723	37758	1.00	1.04	1.01	1.09	0.93	0.85	**1.02**	**0.96**	0.94
Curcumol	JAK	51362	53435	50755	45368	40373	41389	0.98	1.04	0.96	1.00	0.88	0.93	**0.99**	**0.94**	0.94
Decamethonium Bromi	AChR	48259	54657	55091	40797	245	37700	0.92	1.01	1.06	0.95	0.01	0.93	**1.00**	***0.94***	0.94
Santacruzamate A (CAY	HDAC	52511	56465	51036	43471	44460	43418	1.02	1.10	0.97	0.97	0.98	0.97	**1.03**	**0.97**	0.94
Resveratrol	Sirtuin,Autophagy	52046	53888	56768	46651	42758	47313	1.07	1.02	1.09	1.03	0.93	1.03	**1.06**	**1.00**	0.94
Lonidamine	Others	53643	54445	52027	43429	44409	43055	1.02	1.06	0.98	0.96	0.97	0.97	**1.02**	**0.96**	0.94
Labetalol HCl	Adrenergic Receptor	56504	54347	54985	41549	46254	42173	1.11	1.03	1.05	0.95	1.06	1.00	**1.06**	**1.00**	0.94
Amorolfine HCl	Others	53181	52400	55289	43410	42078	42873	0.98	0.98	1.03	0.95	0.90	0.98	**1.00**	**0.94**	0.94
Piracetam	GluR	50008	51353	51124	35998	39084	39620	0.96	0.95	0.98	0.84	0.93	0.98	**0.96**	***0.91***	0.94
Tamoxifen Citrate	Autophagy,Estrogen/progestogen Receptor	53138	48559	50630	40362	40461	40715	0.98	0.91	0.94	0.88	0.87	0.93	**0.95**	**0.89**	0.94
Paeonol	MAO	57898	54457	55184	45052	47118	44797	1.10	1.06	1.04	1.00	1.03	1.00	**1.07**	**1.01**	0.94
Dextrose	Others	57792	51780	62846	45248	46274	46053	1.07	0.97	1.17	0.99	0.99	1.05	**1.07**	**1.01**	0.94
Vardenafil HCl Trihydra	PDE	51163	52086	52436	44569	41924	40800	0.97	1.02	0.99	0.99	0.91	0.91	**0.99**	**0.94**	0.94
6H05	Rho	52154	55222	52518	42249	42823	40251	1.02	1.05	1.00	0.96	0.98	0.95	**1.02**	**0.97**	0.94
Gastrodin	Others	56419	52283	50193	46709	43295	39947	1.07	1.02	0.95	1.03	0.94	0.90	**1.01**	**0.96**	0.94
BI-847325	MEK,Aurora Kinase	21231	21491	19787	15177	16449	19711	0.41	0.42	0.38	0.34	0.36	0.44	**0.40**	**0.38**	0.94
Oxymatrine	Others	54551	53623	53481	46359	43430	42403	1.04	1.05	1.01	1.02	0.95	0.95	**1.03**	**0.97**	0.94
BQ-123	Endothelin Receptor	51159	52324	56020	42657	44905	43359	1.00	1.02	1.06	0.95	0.99	0.97	**1.03**	**0.97**	0.94
Indomethacin	COX	56161	55612	53304	43943	43478	44360	1.04	1.05	0.99	0.96	0.93	1.01	**1.02**	**0.97**	0.94
Pranoprofen	COX	55799	56854	55409	45981	43747	44471	1.03	1.07	1.03	1.00	0.94	1.01	**1.04**	**0.98**	0.94
LY2784544	JAK	20663	19506	19766	16747	17466	14831	0.39	0.38	0.37	0.37	0.38	0.33	**0.38**	**0.36**	0.94
Enoxolone	Dehydrogenase	55397	49319	50789	40971	44098	42101	1.05	0.96	0.96	0.91	0.96	0.94	**0.99**	**0.94**	0.94
Oleanolic Acid	Others	51387	52973	50229	42763	41806	41911	0.98	1.03	0.95	0.95	0.91	0.94	**0.99**	**0.93**	0.94
Climbazole	Others	53308	58992	56520	44357	43794	44149	1.05	1.12	1.08	1.01	1.01	1.04	**1.08**	**1.02**	0.94
Flunarizine 2HCl	Calcium Channel	60429	54851	56606	48400	45287	43654	1.12	1.03	1.05	1.05	0.97	0.99	**1.07**	**1.01**	0.94
5-Aminolevulinic acid H	Others	53779	46964	50030	41851	42313	39180	1.02	0.92	0.95	0.93	0.92	0.88	**0.96**	**0.91**	0.94
DMSO		55428	58942	55256	43897	43640	40484	1.06	1.09	1.06	1.02	1.03	1.00	**1.07**	***1.01***	0.94
Tenofovir Alafenamide	Reverse Transcriptase	46792	45573	49765	40802	34775	41081	0.91	0.89	0.94	0.91	0.76	0.92	**0.91**	**0.86**	0.94
A-966492	PARP	57199	51707	50528	47451	40074	42880	1.09	1.01	0.96	1.05	0.87	0.96	**1.02**	**0.96**	0.94
ML323	DUB	51733	51174	47269	40547	43613	39338	1.01	1.00	0.90	0.91	0.96	0.88	**0.97**	**0.91**	0.94
Flupirtine maleate	DNA/RNA Synthesis	49343	52590	53374	41919	42561	46241	1.01	1.00	1.02	0.93	0.93	1.00	**1.01**	**0.95**	0.94
Ethamsylate	Others	53502	52838	55412	43811	44194	38998	1.05	1.01	1.05	1.00	1.02	0.92	**1.04**	**0.98**	0.94
Chloroprocaine HCl	Others	58107	57181	56051	46940	45185	42431	1.14	1.09	1.07	1.07	1.04	1.00	**1.10**	**1.04**	0.94
VU 0364770	GluR	46731	53131	51508	39754	38415	35598	0.90	0.98	0.99	0.93	0.91	0.88	**0.95**	***0.90***	0.94
WZ4003	AMPK	56397	53594	54113	45648	39298	43866	1.11	1.02	1.03	1.04	0.90	1.04	**1.05**	**0.99**	0.94

DMSO		53869	53364	53364	46382	43804	41826	1.05	1.04	1.01	1.04	0.96	0.93	1.03	0.98	0.94
empty		51471	55980	54984	44416	42583	46490	1.00	1.09	1.04	0.99	0.94	1.04	1.05	0.99	0.94
PQ 401	IGF-1R	51107	53310	53052	39661	43342	46491	1.00	1.04	1.01	0.89	0.95	1.04	1.01	0.96	0.95
PD173955	Bcr-Abl	53111	49590	47924	38578	41434	38340	1.04	0.94	0.91	0.88	0.95	0.91	0.97	0.91	0.95
DMSO		48843	53466	53505	40769	42098	39431	0.96	1.02	1.02	0.93	0.97	0.93	1.00	0.94	0.95
Losmapimod (GW8565	p38 MAPK	53949	55441	53784	43840	40700	43572	1.06	1.05	1.02	1.00	0.94	1.03	1.05	0.99	0.95
Sclareolide	Others	54315	50378	51808	44806	44499	38949	1.03	0.98	0.98	0.99	0.97	0.87	1.00	0.94	0.95
empty		51851	52625	57265	44564	42574	36204	0.99	0.97	1.10	1.04	1.01	0.89	1.02	0.97	0.95
DEL-22379	ERK	49948	50889	54076	41940	40157	45255	0.97	0.99	1.03	0.94	0.88	1.01	1.00	0.94	0.95
Droxinostat	HDAC	48478	50967	53605	45641	36356	46921	0.99	0.97	1.03	1.01	0.79	1.02	1.00	0.94	0.95
Noradrenaline bitartrat	Adrenergic Receptor	57498	58912	56199	45256	47678	48511	1.09	1.15	1.06	1.00	1.04	1.09	1.10	1.04	0.95
Piperlongumine	Others	42630	44268	42640	37628	36117	32836	0.83	0.86	0.81	0.84	0.79	0.73	0.83	0.79	0.95
Tretinoin	Retinoid Receptor	51248	45967	49313	39394	38673	39123	0.95	0.86	0.92	0.86	0.83	0.89	0.91	0.86	0.95
Isovaleramide	Dehydrogenase	55198	57627	53715	45545	47976	37558	1.06	1.06	1.03	1.06	1.14	0.92	1.05	0.99	0.95
GSK126	Histone Methyltransferase	49152	51530	50559	42148	38562	38160	0.97	0.98	0.96	0.96	0.89	0.90	0.97	0.92	0.95
Ivacaftor (VX-770)	CFTR	46140	49893	50994	39917	42629	41367	0.94	0.95	0.98	0.88	0.93	0.90	0.96	0.90	0.95
Macitentan	Endothelin Receptor	48872	51094	54227	40278	41027	45555	0.95	1.00	1.03	0.90	0.90	1.01	0.99	0.94	0.95
Mefloquine HCl	Others	57191	53007	52288	42899	42942	41957	1.12	1.01	0.99	0.98	0.99	0.99	1.04	0.99	0.95
ODM-201	Androgen Receptor	53654	51746	45317	43409	41341	39446	1.05	1.01	0.86	0.97	0.91	0.88	0.97	0.92	0.95
Isoliquiritigenin	Others	49299	48228	46802	43251	36387	38695	0.94	0.94	0.89	0.96	0.79	0.87	0.92	0.87	0.95
LGK-974	Wnt/beta-catenin	50542	53778	53846	44346	42018	38036	0.99	1.02	1.02	1.01	0.97	0.90	1.01	0.96	0.95
LGX818	Raf	49876	49342	48431	40176	37972	37972	0.98	0.94	0.92	0.92	0.87	0.90	0.95	0.90	0.95
empty		48046	49377	49925	40943	37624	37297	0.94	0.94	0.95	0.93	0.86	0.88	0.94	0.89	0.95
HPOB	HDAC	57214	55510	53348	46302	41208	43144	1.12	1.06	1.01	1.06	0.95	1.02	1.06	1.01	0.95
Zotarolimus(ABT-578)	mTOR	29521	30432	29968	22145	25292	23258	0.58	0.58	0.57	0.51	0.58	0.55	0.58	0.55	0.95
MC1568	HDAC	52818	58256	55825	46016	45843	48904	1.08	1.10	1.07	1.02	1.00	1.06	1.09	1.03	0.95
Topiramate	Carbonic Anhydrase	51964	53996	52905	43802	46052	44236	1.06	1.02	1.01	0.97	1.01	0.96	1.03	0.98	0.95
Doripenem Hydrate	Others	51455	51967	57623	45239	45459	45188	1.05	0.99	1.10	1.00	0.99	0.98	1.05	0.99	0.95
Diphemanil Methylsulfa	AChR	53153	55325	53908	42116	156	38902	1.02	1.02	1.03	0.98	0.00	0.96	1.02	0.97	0.95
Duloxetine HCl	5-HT Receptor	59293	61227	60110	46612	50354	47817	1.09	1.15	1.12	1.02	1.08	1.09	1.12	1.06	0.95
Phentolamine Mesylate	Adrenergic Receptor	57874	56139	59561	44558	49437	45158	1.07	1.06	1.11	0.97	1.06	1.03	1.08	1.02	0.95
Asiatic Acid	p38 MAPK	57454	52413	52038	46764	41821	44229	1.09	1.02	0.98	1.03	0.91	0.99	1.03	0.98	0.95
DMSO		55297	54895	56477	45394	46238	42055	1.02	1.03	1.05	0.99	0.99	0.96	1.03	0.98	0.95
Reboxetine mesylate	Others	49333	57287	54078	41701	40590	38450	0.95	1.06	1.04	0.97	0.96	0.95	1.01	0.96	0.95
Guanidine HCl	Others	54151	55279	51727	42914	182	37569	1.04	1.02	0.99	1.00	0.00	0.93	1.02	0.96	0.95
Beclomethasone diprop	Glucocorticoid Receptor	46705	48213	43014	37059	155	31828	0.89	0.89	0.83	0.86	0.00	0.78	0.87	0.82	0.95
Diclofenac Potassium	Others	51753	52394	50047	42342	38521	34749	0.99	0.97	0.96	0.99	0.91	0.86	0.97	0.92	0.95
OC000459	GPR	50682	47452	49673	40871	36115	33103	0.97	0.87	0.95	0.95	0.86	0.82	0.93	0.88	0.95
empty		56726	56442	55857	44759	46022	42322	1.11	1.07	1.06	1.02	1.06	1.00	1.08	1.03	0.95
Marbofloxacin	Topoisomerase	53976	57608	60556	48396	49544	47332	1.11	1.09	1.16	1.07	1.08	1.03	1.12	1.06	0.95
PluriSIn #1 (NSC 14613)	Dehydrogenase	52877	50061	51896	42992	41305	43327	1.03	0.98	0.98	0.96	0.91	0.97	1.00	0.94	0.95
FTI 277 HCl	Transferase	46293	47222	50118	38347	40768	39273	0.90	0.92	0.95	0.86	0.90	0.87	0.92	0.88	0.95
Raltegravir (MK-0518)	Integrase	58291	50335	56361	44380	42634	45144	1.08	0.95	1.05	0.97	0.92	1.03	1.02	0.97	0.95
Cetylpyridinium Chlorid	Others	34073	39113	37251	30291	28337	28268	0.67	0.74	0.71	0.69	0.65	0.67	0.71	0.67	0.95
AGK2	Sirtuin	49770	51400	54476	43655	43584	41048	0.97	1.00	1.03	0.98	0.96	0.91	1.00	0.95	0.95
Uracil	Others	55892	59674	52971	45503	41426	45703	1.10	1.14	1.01	1.04	0.95	1.08	1.08	1.02	0.95
empty		47383	52046	51604	37442	39262	37882	0.91	0.96	0.99	0.87	0.93	0.93	0.95	0.90	0.95
MHY1485	mTOR,Autophagy	51975	51322	52253	42781	41721	43756	1.01	1.00	0.99	0.96	0.92	0.97	1.00	0.95	0.95
Filgotinib (GLPG0634)	JAK	51532	49641	47951	41626	38473	42903	1.00	0.97	0.91	0.93	0.85	0.96	0.96	0.91	0.95
P7C3	NAMPT	51917	50656	51965	43012	41194	43229	1.01	0.99	0.99	0.96	0.91	0.96	1.00	0.94	0.95
Manidipine 2HCl	Calcium Channel	49605	51361	53528	41883	39104	45827	0.94	1.00	1.01	0.93	0.85	1.03	0.99	0.94	0.95
Cyclosporin A	Others	46824	49284	44772	38433	38820	38556	0.89	0.96	0.85	0.85	0.85	0.86	0.90	0.85	0.95
Roscovitine (Seliciclib,C	CDK	50270	55762	52854	44102	46918	43158	1.03	1.06	1.01	0.98	1.03	0.94	1.03	0.98	0.95
Fostamatinib (R788)	Syk	46162	40041	45062	39658	35384	32758	0.88	0.78	0.85	0.88	0.77	0.73	0.84	0.79	0.95
Felbamate	Others	49111	53315	57916	44845	43500	47026	1.01	1.01	1.11	0.99	0.95	1.02	1.04	0.99	0.95
PF-562271 HCl	FAK	50118	47938	53432	42474	42754	39705	0.98	0.94	1.01	0.95	0.94	0.88	0.98	0.92	0.95
Raloxifene HCl	Estrogen/progestogen Receptor	42711	50736	50801	39261	43293	39129	0.87	0.96	0.97	0.87	0.95	0.85	0.94	0.89	0.95
Idoxuridine	Others	55318	51002	53790	41951	41993	44427	1.02	0.96	1.00	0.91	0.90	1.01	0.99	0.94	0.95
SKF38393 HCl	Dopamine Receptor	55444	52690	46315	41848	41752	43969	1.08	1.03	0.88	0.94	0.92	0.98	1.00	0.94	0.95
D-Mannitol	Others	52200	51880	49967	45362	41560	39745	0.99	1.01	0.95	1.00	0.91	0.89	0.98	0.93	0.95
ZM 323881 HCl	VEGFR	48034	54825	47654	39530	37330	35655	0.92	1.01	0.91	0.92	0.88	0.88	0.95	0.90	0.95
Ketoconazole	P450 (e.g. CYP17)	45257	50153	52347	39476	44319	41005	0.93	0.95	1.00	0.88	0.97	0.89	0.96	0.91	0.95
Thiabendazole	Others	55391	55744	54115	44583	46426	41781	1.02	1.05	1.01	0.97	1.00	0.95	1.03	0.97	0.95
Oxcarbazepine	Sodium Channel	47593	52022	56617	43186	47046	41708	0.97	0.99	1.08	0.96	1.03	0.90	1.02	0.96	0.95
AZ 628	Raf	45633	47773	45430	35381	34505	33996	0.87	0.88	0.87	0.82	0.82	0.84	0.88	0.83	0.95
Cloxacillin Sodium	Others	54431	54058	48526	43237	44951	41051	1.03	1.06	0.92	0.96	0.98	0.92	1.00	0.95	0.95
Loxistatin Acid (E-64C)	Cysteine Protease	56643	53567	56008	49086	44106	44018	1.10	1.05	1.06	1.10	0.97	0.98	1.07	1.02	0.95
AZD8055	mTOR	13791	13482	13187	11121	10273	11076	0.25	0.25	0.25	0.24	0.22	0.25	0.25	0.24	0.95

3-Indolebutyric acid (IB.	Others	53146	49076	54206	45399	42351	40875	1.01	0.96	1.03	1.00	0.92	0.92	1.00	0.95	0.95
Tasquinimod	HDAC	50350	46594	46149	40010	40405	37823	0.98	0.91	0.88	0.89	0.89	0.84	0.92	0.88	0.95
Piperine	P450 (e.g. CYP17)	54546	52046	50356	42299	43166	43651	1.04	1.02	0.95	0.94	0.94	0.98	1.00	0.95	0.95
Naringenin	P450 (e.g. CYP17)	53823	49196	48775	42239	41427	41218	1.02	0.96	0.92	0.93	0.90	0.92	0.97	0.92	0.95
empty		52259	53242	58769	42833	37822	39421	1.00	0.98	1.13	1.00	0.90	0.97	1.04	0.98	0.95
StemRegenin 1 (SR1)	AhR	51548	54533	53979	41185	41389	38879	0.99	1.01	1.04	0.96	0.98	0.96	1.01	0.96	0.95
Dipyridamole	PDE	53264	50457	47913	43416	39357	39092	0.98	0.95	0.89	0.95	0.85	0.89	0.94	0.89	0.95
Terazosin HCl	Adrenergic Receptor	60276	59983	57112	46666	48180	47674	1.11	1.13	1.06	1.02	1.04	1.09	1.10	1.05	0.95
Chrysophanic Acid	EGFR,mTOR	49085	47566	49436	40861	40060	39286	0.93	0.93	0.94	0.90	0.87	0.88	0.93	0.89	0.95
empty		54878	47592	52321	43095	44708	39583	1.04	0.93	0.99	0.95	0.97	0.89	0.99	0.94	0.95
DMSO		57424	52023	54306	43864	45051	45802	1.09	1.02	1.03	0.97	0.98	1.03	1.05	0.99	0.95
Oxytetracycline Dihydra	Others	58809	58800	55088	46758	46292	45769	1.09	1.11	1.03	1.02	0.99	1.04	1.07	1.02	0.95
Scopine	Adrenergic Receptor	50616	53014	51387	42645	41998	42981	0.96	1.04	0.97	0.94	0.92	0.96	0.99	0.94	0.95
SB271046	5-HT Receptor	51088	53338	52721	43266	40931	35546	0.98	0.98	1.01	1.01	0.97	0.88	0.99	0.94	0.95
empty		52507	50821	54668	40377	44267	45399	1.00	0.99	1.03	0.89	0.96	1.02	1.01	0.96	0.95
DMSO		59258	56950	58353	47654	47532	45241	1.09	1.07	1.09	1.04	1.02	1.03	1.08	1.03	0.95
Amprolium HCl	Others	53256	53794	53165	41640	43837	41036	1.05	1.02	1.01	0.95	1.01	0.97	1.03	0.98	0.95
Malotilate	Others	48069	55419	54186	43382	43422	46611	0.98	1.05	1.04	0.96	0.95	1.01	1.02	0.97	0.95
O4I2	Others	54267	55291	53769	44754	45679	44661	1.06	1.08	1.02	1.00	1.00	1.00	1.05	1.00	0.95
Allylthiourea	Others	55283	52216	56584	42992	162	39282	1.06	0.96	1.09	1.00	0.00	0.97	1.04	0.98	0.95
PD123319	RAAS	50037	51589	54546	43642	39151	40508	0.98	0.98	1.04	1.00	0.90	0.96	1.00	0.95	0.95
Dienogest	Estrogen/progestogen Receptor	50207	52732	57222	44308	46199	45129	1.03	1.00	1.10	0.98	1.01	0.98	1.04	0.99	0.95
BIO	GSK-3	55134	57426	61100	47908	44352	44848	1.08	1.09	1.16	1.09	1.02	1.06	1.11	1.06	0.95
DMSO		47738	49786	52222	43769	40297	42798	0.98	0.94	1.00	0.97	0.88	0.93	0.97	0.93	0.95
Cyclandelate	Others	52332	54506	51601	42149	40811	42174	1.03	1.04	0.98	0.96	0.94	1.00	1.02	0.97	0.95
Lamotrigine	5-HT Receptor,Sodium Channel	48005	49417	47040	39582	33644	32933	0.92	0.91	0.90	0.92	0.80	0.81	0.91	0.87	0.95
Sulphadimethoxine	Others	57295	58228	55007	47716	46069	43608	1.06	1.09	1.02	1.04	0.99	0.99	1.06	1.01	0.95
Acesulfame Potassium	Others	51506	50524	51783	39677	38333	37455	0.99	0.93	0.99	0.93	0.91	0.92	0.97	0.92	0.95
Doxercalciferol	Others	46243	50568	56491	43148	41245	45459	0.95	0.96	1.08	0.96	0.90	0.99	1.00	0.95	0.95
Andrographolide	NF-κB	54201	50337	52879	44682	45174	39969	1.03	0.98	1.00	0.99	0.98	0.90	1.00	0.96	0.95
AS-252424	PI3K	53330	53777	53954	45009	43122	44657	1.01	1.05	1.02	0.99	0.94	1.00	1.03	0.98	0.95
Fulvestrant	Estrogen/progestogen Receptor	44512	51462	52686	40274	45281	40326	0.91	0.98	1.01	0.89	0.99	0.87	0.97	0.92	0.95
Sclareol	Others	54703	57945	56044	48254	47298	43665	1.04	1.13	1.06	1.07	1.03	0.98	1.08	1.03	0.95
Salicylanilide	Reverse Transcriptase	57365	55287	55012	46373	42067	44164	1.13	1.05	1.05	1.06	0.97	1.04	1.08	1.02	0.95
empty		48408	52492	54516	41373	44644	45773	0.99	1.00	1.04	0.92	0.98	0.99	1.01	0.96	0.95
Amiloride HCl	Sodium Channel	57406	54238	52032	42546	44356	44860	1.06	1.02	0.97	0.93	0.95	1.02	1.02	0.97	0.95
NPS-2143	CaSR	48657	54306	53828	46357	41469	41540	0.93	1.06	1.02	1.02	0.90	0.93	1.00	0.95	0.95
SBC-115076	Others	50989	56369	55828	44222	44443	46458	0.99	1.10	1.06	0.99	0.98	1.04	1.05	1.00	0.95
DMSO		48458	50743	49750	39546	44368	42517	0.99	0.96	0.95	0.88	0.97	0.92	0.97	0.92	0.95
Ozagrel	P450 (e.g. CYP17)	50731	52158	47234	42640	40800	40488	0.96	1.02	0.89	0.94	0.89	0.91	0.96	0.91	0.95
LCL161	IAP	51698	50028	52672	40163	41019	40942	1.02	0.95	1.00	0.92	0.94	0.97	0.99	0.94	0.95
MRS 2578	P2 Receptor	53391	45964	55318	39920	38164	37812	1.02	0.85	1.06	0.93	0.90	0.93	0.98	0.93	0.95
PJ34 HCl	PARP	54324	54003	53209	42747	44578	40551	1.07	1.03	1.01	0.98	1.02	0.96	1.04	0.99	0.95
Cyclizine 2HCl	Histamine Receptor	53462	52473	52990	44188	41417	40210	1.05	1.00	1.01	1.01	0.95	0.95	1.02	0.97	0.95
KU-55933 (ATM Kinase	ATM/ATR	48350	59219	53741	44203	44350	48179	0.99	1.12	1.03	0.98	0.97	1.04	1.05	1.00	0.95
RG2833 (RGFP109)	HDAC	57732	57020	52725	45395	44786	42449	1.13	1.08	1.00	1.04	1.03	1.00	1.07	1.02	0.95
Pravastatin sodium	HMG-CoA Reductase	49720	50767	51013	41292	37424	34870	0.95	0.94	0.98	0.96	0.89	0.86	0.96	0.91	0.95
CORM-3	Others	51186	48587	52583	38436	44019	43841	1.00	0.95	1.00	0.86	0.97	0.98	0.98	0.93	0.95
Pimecrolimus	Others	54861	53075	52864	41435	42050	43767	1.08	1.01	1.01	0.95	0.97	1.03	1.03	0.98	0.95
Econazole nitrate	Calcium Channel	48074	51154	45070	42229	39852	37180	0.91	1.00	0.85	0.93	0.87	0.83	0.92	0.88	0.95
CCT128930	Akt	59180	52987	54619	48237	45797	43663	1.13	1.03	1.03	1.07	1.00	0.98	1.06	1.01	0.95
Avagacestat (BMS-708:	Beta Amyloid,Gamma-secretase	41654	51105	51195	39056	40582	42380	0.85	0.97	0.98	0.87	0.89	0.92	0.93	0.89	0.95
Toremifene Citrate	Estrogen/progestogen Receptor	48254	51483	48110	37369	39615	42160	0.89	0.97	0.90	0.81	0.85	0.96	0.92	0.88	0.95
Cortisone acetate	Glucocorticoid Receptor	49124	45616	47277	40521	38193	38542	0.93	0.89	0.89	0.90	0.83	0.86	0.91	0.86	0.95
Cytidine	Others	60219	57260	49567	46133	46610	42128	1.11	1.08	0.92	1.01	1.00	0.96	1.04	0.99	0.95
Idasanutlin (RG-7388)	Mdm2	53106	53485	47392	42654	41765	37678	1.04	1.02	0.90	0.97	0.96	0.89	0.99	0.94	0.95
Pizotifen Malate	5-HT Receptor	46458	50329	56320	42624	44339	42994	0.95	0.95	1.08	0.94	0.97	0.93	0.99	0.95	0.95
Apremilast (CC-10004)	PDE	52345	51225	52098	43686	42081	43356	1.02	1.00	0.99	0.98	0.93	0.97	1.00	0.96	0.95
Bufexamac	COX	44617	47255	45942	37005	39134	32286	0.85	0.87	0.88	0.86	0.93	0.80	0.87	0.83	0.95
Dynasore	Dynamin	51985	49095	54480	40128	43291	45599	1.01	0.96	1.03	0.90	0.95	1.02	1.00	0.95	0.95
empty		55756	54228	60703	46916	44299	46443	1.03	1.02	1.13	1.02	0.95	1.06	1.06	1.01	0.95
TIC10 Analogue	Akt	51070	51578	56132	42239	42309	41211	1.00	0.98	1.07	0.96	0.97	0.97	1.02	0.97	0.95
SU11274	c-Met	41510	52720	54375	41787	44326	39790	0.85	1.00	1.04	0.93	0.97	0.86	0.96	0.92	0.95
Triamterene	Sodium Channel	53139	57206	48097	41915	37234	37677	1.02	1.05	0.92	0.98	0.88	0.93	1.00	0.95	0.95
Ambroxol HCl	Sodium Channel	45797	49050	51769	38257	188	35449	0.88	0.90	0.99	0.89	0.00	0.87	0.93	0.88	0.95
Trifluridine	DNA/RNA Synthesis	54858	53800	52010	43497	44429	41833	1.01	1.01	0.97	0.95	0.95	0.95	1.00	0.95	0.95
DMSO		50095	50859	50374	42320	39908	43357	0.98	0.99	0.96	0.95	0.88	0.97	0.97	0.93	0.95
Tranylcypromine (2-PCF	MAO	49019	50000	52135	40135	37779	41794	0.96	0.95	0.99	0.92	0.87	0.99	0.97	0.92	0.95

Levofloxacin	Topoisomerase	56684	57910	50659	43555	46932	43045	1.05	1.09	0.94	0.95	1.01	0.98	1.03	0.98	0.95
Oxybutynin	AChR	56326	54615	56629	43951	46523	44839	1.04	1.03	1.05	0.96	1.00	1.02	1.04	0.99	0.95
2-Thiouracil	Others	50347	50321	51428	40646	33240	35913	0.96	0.93	0.99	0.95	0.79	0.88	0.96	*0.92*	0.95
Fluocinonide	Glucocorticoid Receptor	50941	51459	47511	37904	44561	41594	0.97	1.00	0.90	0.84	0.97	0.93	0.96	0.91	0.95
Sesamin	Others	54299	48345	49171	43137	41663	40755	1.03	0.94	0.93	0.95	0.91	0.91	0.97	0.93	0.95
Zidovudine	Reverse Transcriptase	53942	51226	54191	45358	44967	41518	1.03	1.00	1.03	1.00	0.98	0.93	1.02	0.97	0.95
Apocynin	Others	54198	53546	51066	45401	45857	40231	1.03	1.05	0.97	1.00	1.00	0.90	1.01	0.97	0.95
Prucalopride	5-HT Receptor	46756	49326	53210	39433	33500	35732	0.90	0.91	1.02	0.92	0.79	0.88	0.94	*0.90*	0.95
YK-4-279	DNA/RNA Synthesis	39740	41212	39398	32425	32392	35179	0.77	0.80	0.75	0.73	0.71	0.78	0.78	0.74	0.95
XMD8-92	ERK	50662	47679	50105	42161	41863	39314	0.99	0.93	0.95	0.94	0.92	0.88	0.96	0.91	0.96
Clonidine HCl	Autophagy,Adrenergic Receptor	48941	49397	50866	42350	41782	39371	0.93	0.96	0.96	0.94	0.91	0.88	0.95	0.91	0.96
Mupirocin	DNA/RNA Synthesis	47662	60936	46684	41233	42064	39826	0.94	1.16	0.89	0.94	0.97	0.94	0.99	0.95	0.96
DMSO		49726	56463	54021	46235	44326	45672	1.02	1.07	1.03	1.02	0.97	0.99	1.04	0.99	0.96
Endoxifen HCl	Estrogen/progestogen Receptor	52228	53385	51268	45288	39617	45445	1.02	1.04	0.97	1.01	0.87	1.01	1.01	0.97	0.96
Doxofylline	PDE	51090	54202	52588	39536	44005	41690	1.00	1.03	1.00	0.90	1.01	0.99	1.01	0.97	0.96
Candesartan Cilexetil	RAAS	57204	52337	57647	46647	44303	44183	1.06	0.98	1.07	1.02	0.95	1.01	1.04	0.99	0.96
Miglitol	Others	54571	51537	56427	48320	42193	43969	1.04	1.01	1.07	1.07	0.92	0.99	1.04	0.99	0.96
Irsogladine	AChR,PDE	55544	59230	51315	46160	47011	41318	1.03	1.11	0.96	1.01	1.01	0.94	1.03	0.99	0.96
Sorbitol	Others	51507	45165	49217	43071	40138	37579	0.98	0.88	0.93	0.95	0.87	0.84	0.93	0.89	0.96
Silymarin	Others	51510	48977	53270	41754	43775	41793	0.98	0.96	1.01	0.92	0.95	0.94	0.98	0.94	0.96
Ondansetron HCl	5-HT Receptor	48226	52725	56691	46670	44717	42732	0.99	1.00	1.09	1.03	0.98	0.93	1.02	0.98	0.96
Baicalin	GABA Receptor	56734	54978	54076	45381	48165	43863	1.08	1.07	1.02	1.00	1.05	0.98	1.06	1.01	0.96
Solifenacin succinate	AChR	48969	50653	54002	39186	10026	38196	0.94	0.93	1.04	0.91	0.24	0.94	0.97	*0.93*	0.96
BMY 7378	5-HT Receptor,Adrenergic Receptor	53475	54496	53383	45706	43252	44773	1.02	1.06	1.01	1.01	0.94	1.00	1.03	0.99	0.96
Honokiol	MEK,Akt	50778	51020	52460	43107	39889	44777	0.97	1.00	0.99	0.95	0.87	1.00	0.98	0.94	0.96
Mdivi-1	Dynamin	51762	51118	52279	43116	42150	38124	1.02	0.97	0.99	0.98	0.97	0.90	0.99	0.95	0.96
CH-223191	AhR	50404	50185	53147	43686	42130	42108	0.98	0.98	1.01	0.98	0.93	0.94	0.99	0.95	0.96
Triciribine	Akt	37632	42567	42772	34541	34898	35313	0.77	0.81	0.82	0.77	0.76	0.77	0.80	0.76	0.96
Erythritol	Others	53264	50375	51604	40576	40097	42724	1.05	0.96	0.98	0.93	0.92	1.01	1.00	0.95	0.96
Quercetin Dihydrate	Others	47002	46533	53160	42858	41637	37161	0.89	0.91	1.01	0.95	0.91	0.83	0.94	0.90	0.96
Idebenone	Others	56821	53612	55823	49021	46730	42163	1.08	1.05	1.06	1.08	1.02	0.95	1.06	1.02	0.96
Zibotentan (ZD4054)	Endothelin Receptor	54789	58800	54778	47619	49021	46991	1.12	1.11	1.05	1.06	1.07	1.02	1.10	1.05	0.96
Pimobendan	PDE	56452	53721	52703	45426	41421	44982	1.04	1.01	0.98	0.99	0.89	1.02	1.01	0.97	0.96
Dorsomorphin (Compou	AMPK	50037	47167	53821	41869	41213	42630	0.98	0.92	1.02	0.94	0.91	0.95	0.97	0.93	0.96
Altretamine	Others	45703	50351	53011	44997	41519	40484	0.94	0.95	1.02	1.00	0.91	0.88	0.97	0.93	0.96
HA14-1	Bcl-2	50807	55109	64093	47581	49363	47917	1.04	1.04	1.23	1.05	1.08	1.04	1.10	1.06	0.96
Nifedipine	Calcium Channel	55996	53232	53069	43541	43349	44526	1.03	1.00	0.99	0.95	0.93	1.01	1.01	0.96	0.96
TCID	DUB	53596	54794	51920	42112	42608	42772	1.05	1.04	0.99	0.96	0.98	1.01	1.03	0.98	0.96
Ambrisentan	Endothelin Receptor	60175	57367	53939	47129	45743	46043	1.11	1.08	1.00	1.03	0.98	1.05	1.06	1.02	0.96
Rocuronium Bromide	AChR	46955	52984	53456	41567	43691	45554	0.96	1.00	1.02	0.92	0.96	0.99	1.00	0.95	0.96
CGI1746	BTK	48887	51150	49558	39847	37594	41494	0.96	0.97	0.94	0.91	0.86	0.98	0.96	0.92	0.96
DMSO		55066	53046	51860	44780	40975	41601	1.08	1.01	0.99	1.02	0.94	0.98	1.03	0.98	0.96
AR-A014418	GSK-3	52115	52619	46544	42652	41287	42222	1.02	1.03	0.88	0.95	0.91	0.94	0.97	0.93	0.96
GSK1904529A	IGF-1R	51070	53506	56888	43983	45022	48879	1.05	1.01	1.09	0.97	0.98	1.06	1.05	1.01	0.96
Cyclobenzaprine HCl	Others	52030	50929	54745	43120	41821	40614	1.02	0.97	1.04	0.98	0.96	0.96	1.01	0.97	0.96
Phosphoramidon Disodi	Others	54735	51751	52960	43566	45447	43944	1.07	1.01	1.00	0.97	1.00	0.98	1.03	0.98	0.96
Clindamycin palmitate	Others	57314	53203	55227	46287	45186	46108	1.09	1.04	1.04	1.02	0.98	1.03	1.06	1.01	0.96
Tubastatin A HCl	HDAC	58916	49404	53874	46145	45320	43162	1.12	0.96	1.02	1.02	0.99	0.97	1.03	0.99	0.96
empty		55520	55731	53844	43644	45050	45196	1.03	1.05	1.00	0.95	0.97	1.03	1.02	0.98	0.96
Tofacitinib (CP-690550)	JAK	52012	50854	47478	38163	42169	39440	1.02	0.97	0.90	0.87	0.97	0.93	0.96	0.92	0.96
Tenatoprazole	Proton Pump	51762	53243	52120	40054	43998	41069	1.02	1.01	0.99	0.91	1.01	0.97	1.01	0.97	0.96
Ganetespib (STA-9090)	HSP (e.g. HSP90)	16766	15844	18349	13667	15815	14098	0.34	0.30	0.35	0.30	0.35	0.31	0.33	0.32	0.96
Lubiprostone	CFTR	54685	56204	56492	45245	46001	44593	1.01	1.06	1.05	0.99	0.99	1.02	1.04	1.00	0.96
Ketanserin	5-HT Receptor	53665	53955	50619	44524	45065	41975	1.02	1.05	0.96	0.98	0.98	0.94	1.01	0.97	0.96
empty		54602	52833	55350	46003	45192	44586	1.06	1.03	1.05	1.03	0.99	0.99	1.05	1.01	0.96
Bromodeoxyuridine (Br	DNA/RNA Synthesis	52737	49294	53595	43657	40593	45538	1.03	0.96	1.02	0.98	0.89	1.01	1.00	0.96	0.96
empty		51990	52200	50839	39821	39217	38492	1.00	0.96	0.98	0.93	0.93	0.95	0.98	*0.94*	0.96
Ifosfamide	DNA/RNA Synthesis	48903	49158	51936	42272	44554	41430	1.00	0.93	1.00	0.94	0.97	0.90	0.98	0.94	0.96
GSK690693	Akt	44927	45637	49206	38363	39168	41992	0.92	0.87	0.94	0.85	0.86	0.91	0.91	0.87	0.96
Isosorbide	Others	53830	54977	56535	43875	45623	42263	1.06	1.05	1.08	1.00	1.05	1.00	1.06	1.02	0.96
Sucralose	Others	50453	53856	52970	40429	44302	40568	0.99	1.02	1.01	0.92	1.02	0.96	1.01	0.97	0.96
GSK J1	Histone Demethylase	50518	49412	50765	43876	37323	44509	0.98	0.96	0.96	0.98	0.82	0.99	0.97	0.93	0.96
Oligomycin A	ATPase	37996	39752	35894	30828	32534	33910	0.78	0.75	0.69	0.68	0.71	0.73	0.74	0.71	0.96
UNC0631	Histone Methyltransferase	49466	52888	52262	39554	44157	45385	0.96	1.03	0.99	0.88	0.97	1.01	1.00	0.96	0.96
Gimeracil	Dehydrogenase	55495	53401	52249	45750	44405	40795	1.02	1.00	0.97	1.00	0.95	0.93	1.00	0.96	0.96
Meropenem	Others	52585	54860	56297	47689	48298	43994	1.08	1.04	1.08	1.06	1.06	0.95	1.07	1.02	0.96
SGX-523	c-Met	52659	53509	57616	46353	49027	44685	1.08	1.01	1.10	1.03	1.07	0.97	1.07	1.02	0.96
CGK 733	ATM/ATR	49918	48497	47911	39253	40639	36839	0.98	0.92	0.91	0.90	0.93	0.87	0.94	0.90	0.96

MK-801 (Dizocilpine)	GluR	52431	51203	50202	41042	39737	36804	1.00	0.94	0.96	0.96	0.94	0.91	**0.97**	***0.93***	0.96
BIBR 1532	Telomerase	53382	56645	54933	46525	47443	47144	1.09	1.07	1.05	1.03	1.04	1.02	**1.07**	**1.03**	0.96
Glimepiride	Potassium Channel	53305	55294	56413	45006	47318	48876	1.09	1.05	1.08	1.00	1.03	1.06	**1.07**	**1.03**	0.96
Xylose	Others	60554	58318	55313	43584	49629	48210	1.12	1.10	1.03	0.95	1.07	1.10	**1.08**	**1.04**	0.96
empty		52245	52389	50158	43883	41617	43782	1.02	1.02	0.95	0.98	0.91	0.98	**1.00**	**0.96**	0.96
(-)-Epigallocatechin Ga	Others	55723	55899	52485	50260	41489	44749	1.06	1.09	0.99	1.11	0.90	1.00	**1.05**	**1.01**	0.96
Moxonidine	Others	58133	58189	54668	46849	45304	46710	1.07	1.09	1.02	1.02	0.97	1.06	**1.06**	**1.02**	0.96
empty		56932	59233	55474	50278	43761	45419	1.05	1.11	1.03	1.10	0.94	1.03	**1.07**	**1.02**	0.96
DMSO		48539	46753	56677	39545	43827	40148	0.90	0.88	1.05	0.86	0.94	0.91	**0.94**	**0.91**	0.96
AZD5363	Akt	42102	49701	47512	39628	37048	39677	0.82	0.97	0.90	0.89	0.81	0.88	**0.90**	**0.86**	0.96
GNE-0877	LRRK2	52764	53382	51778	45693	42562	43698	1.03	1.04	0.98	1.02	0.94	0.97	**1.02**	**0.98**	0.96
Cefditoren Pivoxil	Others	52952	55336	54731	44250	45524	42800	0.98	1.04	1.02	0.96	0.98	0.97	**1.01**	**0.97**	0.96
Degrasyn (WP1130)	Bcr-Abl,DUB	45036	46353	48391	42090	37587	36664	0.86	0.91	0.92	0.93	0.82	0.82	**0.89**	**0.86**	0.96
EPZ015666	Histone Methyltransferase	47620	46341	47919	37393	41559	39644	0.93	0.90	0.91	0.84	0.91	0.88	**0.91**	**0.88**	0.96
PFI-1 (PF-6405761)	Epigenetic Reader Domain	47652	52743	49352	40072	47329	40759	0.98	1.00	0.95	0.89	1.03	0.88	**0.97**	**0.94**	0.96
BRD73954	HDAC	44196	46877	47728	38260	39080	38641	0.86	0.91	0.90	0.86	0.86	0.86	**0.89**	**0.86**	0.96
Betaxolol	Adrenergic Receptor	55918	52201	54543	47989	41350	42857	1.03	0.98	1.02	1.05	0.89	0.98	**1.01**	**0.97**	0.96
Scriptaid	HDAC	46985	45818	47132	37638	41416	37931	0.92	0.89	0.89	0.84	0.91	0.85	**0.90**	**0.87**	0.96
Purmorphamine	Hedgehog/Smoothened	45085	52333	50616	40524	39319	34458	0.86	0.96	0.97	0.94	0.93	0.85	**0.93**	***0.90***	0.96
GDC-0152	IAP	49040	47150	47972	39581	36710	38767	0.96	0.90	0.91	0.90	0.84	0.92	**0.92**	**0.89**	0.96
JZL184	Others	54524	51572	49916	44058	40045	40504	1.07	0.98	0.95	1.01	0.92	0.96	**1.00**	**0.96**	0.96
C646	Histone Acetyltransferase	54260	53048	59792	45460	43411	44451	1.07	1.01	1.14	1.04	1.00	1.05	**1.07**	**1.03**	0.96
Benzoic Acid	Others	51059	52748	57247	45001	41155	42340	1.00	1.00	1.09	1.03	0.95	1.00	**1.03**	**0.99**	0.96
Enalapril Maleate	RAAS	54739	57055	55860	41880	48709	45748	1.01	1.07	1.04	0.91	1.05	1.04	**1.04**	**1.00**	0.96
Pancuronium dibromide	AChR	53408	51894	54141	45459	44622	42671	1.02	1.01	1.02	1.00	0.97	0.96	**1.02**	**0.98**	0.96
Chlorzoxazone	Others	51470	55179	55655	44240	42747	42529	1.01	1.05	1.06	1.01	0.98	1.01	**1.04**	**1.00**	0.96
K-Ras(G12C) inhibitor 1	Rho	41009	40459	38804	31605	32201	32225	0.81	0.77	0.74	0.72	0.74	0.76	**0.77**	**0.74**	0.96
PHA-793887	CDK	42526	50263	47170	40005	40064	39630	0.87	0.95	0.90	0.89	0.88	0.86	**0.91**	**0.87**	0.96
Rifampin	DNA/RNA Synthesis	57520	59145	57341	46925	48852	45822	1.06	1.11	1.07	1.02	1.05	1.04	**1.08**	**1.04**	0.96
Allopurinol Sodium	Others	56395	58845	55647	46243	46090	46643	1.04	1.11	1.04	1.01	0.99	1.06	**1.06**	**1.02**	0.96
Doxylamine Succinate	Histamine Receptor	51453	52773	49250	42954	39619	40017	1.01	1.00	0.94	0.98	0.91	0.95	**0.98**	**0.95**	0.96
Cilengitide	Integrin	48758	52331	50306	41968	40362	38582	0.96	1.00	0.96	0.96	0.93	0.91	**0.97**	**0.93**	0.96
Tetracaine HCl	Calcium Channel	50745	48905	52501	44597	41960	40185	0.96	0.96	0.99	0.99	0.91	0.90	**0.97**	**0.93**	0.96
BLZ945	CSF-1R	54610	48387	50313	40626	45527	42171	1.06	0.94	0.95	0.91	1.00	0.94	**0.99**	**0.95**	0.96
Costunolide	Telomerase	50382	46023	49899	43508	42165	39884	1.03	0.87	0.96	0.96	0.92	0.86	**0.95**	**0.92**	0.96
Dexrazoxane HCl (ICRF-	Others	50145	56949	54251	44804	48175	45177	1.03	1.08	1.04	0.99	1.05	0.98	**1.05**	**1.01**	0.96
Lafutidine	Histamine Receptor	56416	53191	53075	45866	40571	45789	1.04	1.00	0.99	1.00	0.87	1.04	**1.01**	**0.97**	0.96
Rocilinostat (ACY-1215)	HDAC	45229	49544	51015	38848	42128	40986	0.88	0.97	0.97	0.87	0.93	0.91	**0.94**	**0.90**	0.96
Cinchophen	Others	53840	52986	55580	45162	43743	40911	1.06	1.01	1.06	1.03	1.01	0.97	**1.04**	**1.00**	0.96
Amisulpride	Dopamine Receptor	48587	49566	52249	44976	41125	42805	0.99	0.94	1.00	1.00	0.90	0.93	**0.98**	**0.94**	0.96
Nicotinamide (Vitamin	Sirtuin	56842	55759	53895	43622	47189	44727	1.05	1.05	1.00	0.95	1.01	1.02	**1.03**	**0.99**	0.96
DMSO		52985	48397	56860	44754	42650	44916	1.03	0.94	1.08	1.00	0.94	1.00	**1.02**	**0.98**	0.96
Ospemifene	Estrogen/progestogen Receptor	55042	51785	50705	41708	43640	40642	1.08	0.99	0.96	0.95	1.00	0.96	**1.01**	**0.97**	0.96
PFI-3	Epigenetic Reader Domain	54112	61468	57129	45580	48256	44145	1.06	1.17	1.09	1.04	1.11	1.04	**1.11**	**1.06**	0.96
Bafetinib (INNO-406)	Bcr-Abl	47678	50121	48018	40995	38967	45139	0.98	0.95	0.92	0.91	0.85	0.98	**0.95**	**0.91**	0.96
empty		49638	55137	51973	40743	44524	45982	0.97	1.08	0.99	0.91	0.98	1.02	**1.01**	**0.97**	0.96
Anidulafungin (LY30336	Others	48035	50782	53322	43054	42342	36269	0.94	0.97	1.01	0.98	0.97	0.86	**0.97**	**0.94**	0.96
empty		51301	49725	48196	40332	42463	41644	0.98	0.97	0.91	0.89	0.93	0.93	**0.95**	**0.92**	0.96
Shikimic Acid	Others	52436	55606	50987	45432	46434	40886	1.00	1.09	0.96	1.00	1.01	0.92	**1.02**	**0.98**	0.96
Calpeptin	Cysteine Protease	52401	57520	52738	46533	45022	44671	1.02	1.12	1.00	1.04	0.99	1.00	**1.05**	**1.01**	0.96
Deoxycorticosterone ac	Others	51431	51371	48216	42404	38231	40141	1.01	0.98	0.92	0.97	0.88	0.95	**0.97**	**0.93**	0.96
IWP-L6	Wnt/beta-catenin	54285	54673	53240	43279	43361	43042	1.07	1.04	1.01	0.99	1.00	1.02	**1.04**	**1.00**	0.96
DDR1-IN-1	Others	50566	53585	49291	40254	43256	45041	0.99	1.05	0.93	0.90	0.95	1.00	**0.99**	**0.95**	0.96
Antazoline HCl	Others	48575	56749	55749	44079	43014	41615	0.95	1.08	1.06	1.01	0.99	0.98	**1.03**	**0.99**	0.96
empty		51748	51990	52391	41815	43620	41783	0.96	0.98	0.98	0.91	0.94	0.95	**0.97**	**0.93**	0.96
Methylthiouracil	Others	45648	50457	51015	37481	39196	37076	0.87	0.93	0.98	0.87	0.93	0.91	**0.93**	***0.89***	0.96
Valaciclovir HCl	Others	56742	58066	50439	47726	45182	41847	1.05	1.09	0.94	1.04	0.97	0.95	**1.03**	**0.99**	0.96
Natamycin	Others	47244	53090	55595	43383	45562	44679	0.97	1.01	1.07	0.96	1.00	0.97	**1.01**	**0.98**	0.96
Nobiletin	MMP	53723	47383	46001	43721	39642	39342	1.02	0.93	0.87	0.97	0.86	0.88	**0.94**	**0.90**	0.96
Lomitapide Mesylate	Others	50763	46327	44964	39301	38785	40953	0.99	0.90	0.85	0.88	0.85	0.91	**0.92**	**0.88**	0.96
DMSO		54230	57068	51431	41755	40062	40710	1.04	1.05	0.99	0.97	0.95	1.00	**1.03**	***0.99***	0.96
Meclofenoxate (Centro	Others	54378	49518	50708	42071	42303	39411	1.07	0.94	0.96	0.96	0.97	0.93	**0.99**	**0.95**	0.96
Synephrine	Adrenergic Receptor	53077	52883	54504	46771	43010	44074	1.01	1.03	1.03	1.03	0.94	0.99	**1.02**	**0.99**	0.96
PF-06463922	ALK	55457	51106	42507	40131	39680	45231	1.08	1.00	0.81	0.90	0.87	1.01	**0.96**	**0.93**	0.96
Bimatoprost	Others	53564	52897	54929	42385	45864	50448	1.10	1.00	1.05	0.94	1.00	1.09	**1.05**	**1.01**	0.96
Piceatannol	Syk	44982	47925	49157	39685	36088	32529	0.86	0.88	0.94	0.93	0.85	0.80	**0.90**	***0.86***	0.96
Erythromycin Ethylsucci	Others	53197	54391	54487	44481	172	37867	1.02	1.00	1.05	1.04	0.00	0.93	**1.02**	***0.98***	0.96
Desloratadine	Histamine Receptor	48093	57840	54324	42569	42630	38704	0.92	1.07	1.04	0.99	1.01	0.95	**1.01**	***0.97***	0.96

LDC000067	CDK	53048	50805	51332	41130	44793	44141	1.03	0.99	0.97	0.92	0.98	0.98	1.00	0.96	0.96
ZM 39923 HCl	JAK	53633	54910	54404	42582	43306	50631	1.05	1.07	1.03	0.95	0.95	1.13	1.05	1.01	0.96
R428 (BGB324)	TAM Receptor	39523	42883	44330	32746	34508	31542	0.76	0.79	0.85	0.76	0.82	0.78	0.80	*0.77*	0.96
GSK1059615	mTOR,PI3K	48813	49333	54143	44202	43521	42986	1.00	0.94	1.04	0.98	0.95	0.93	0.99	0.95	0.96
Org 27569	Cannabinoid Receptor	57192	58126	54140	44804	46770	46519	1.06	1.09	1.01	0.98	1.01	1.06	1.05	1.01	0.96
Vitamin D3	Others	50752	51457	50960	40676	40508	37089	0.97	0.95	0.98	0.95	0.96	0.91	0.97	*0.93*	0.96
Ginkgolide B	PAFR	51211	54097	52683	44394	45814	45446	1.05	1.03	1.01	0.98	1.00	0.98	1.03	0.99	0.96
Evacetrapib (LY248459	CETP	49336	48883	50848	41134	39140	34663	0.95	0.90	0.98	0.96	0.93	0.85	0.94	*0.91*	0.96
Metolazone	Others	60855	56887	54752	48881	46203	45575	1.12	1.07	1.02	1.07	0.99	1.04	1.07	1.03	0.96
Gestodene	Estrogen/progestogen Receptor	47431	51502	52288	42992	45162	41628	0.97	0.98	1.00	0.95	0.99	0.90	0.98	0.95	0.96
Carmofur	DNA/RNA Synthesis	47155	46685	50764	37498	43694	43131	0.97	0.89	0.97	0.83	0.96	0.93	0.94	0.91	0.96
XL019	JAK	49795	56201	48029	41282	40775	41216	0.98	1.07	0.91	0.94	0.94	0.97	0.99	0.95	0.96
Naratriptan	5-HT Receptor	50706	55515	57527	47087	43086	50395	1.04	1.05	1.10	1.04	0.94	1.09	1.06	1.03	0.96
Dibenzothiophene	Others	56574	56296	54474	46049	46019	42033	1.11	1.07	1.04	1.05	1.06	0.99	1.07	1.03	0.96
Epinephrine Bitartrate	Adrenergic Receptor	55490	51824	52371	46683	46581	40195	1.06	1.01	0.99	1.03	1.02	0.90	1.02	0.98	0.96
Isoniazid	Others	58201	55734	50315	47407	43021	43561	1.07	1.05	0.94	1.03	0.92	0.99	1.02	0.98	0.96
Isoconazole nitrate	Others	52360	42578	50785	42042	40257	39313	1.00	0.83	0.96	0.93	0.88	0.88	0.93	0.90	0.96
Busulfan	Others	56265	56403	51452	46717	45074	42207	1.04	1.06	0.96	1.02	0.97	0.96	1.02	0.98	0.96
Bupivacaine HCl	Sodium Channel	50222	48908	45500	39707	40117	41026	0.95	0.96	0.86	0.88	0.87	0.92	0.92	0.89	0.96
Flopropione	Others	50883	50224	53144	42536	39554	41434	1.00	0.96	1.01	0.97	0.91	0.98	0.99	0.95	0.96
Tangeretin	Others	52563	48325	49976	44982	42297	38776	1.00	0.94	0.95	0.99	0.92	0.87	0.96	0.93	0.96
SB431542	TGF-beta/Smad	51526	51302	52305	42125	44951	46388	1.05	0.97	1.00	0.93	0.98	1.01	1.01	0.97	0.96
Z-VAD-FMK	Caspase	50417	49303	53109	43485	38859	40118	0.99	0.94	1.01	0.99	0.89	0.95	0.98	0.94	0.96
Cyclophosphamide Mor	Others	59911	54141	55744	47432	48335	42941	1.11	1.02	1.04	1.03	1.04	0.98	1.05	1.02	0.96
Sulfamethizole	Others	57201	52080	49698	43010	42027	44602	1.06	0.98	0.93	0.94	0.90	1.02	0.99	0.95	0.96
Staurosporine	PKC	5907	5895	6732	5811	4775	5341	0.12	0.11	0.13	0.13	0.10	0.12	0.12	0.12	0.96
Camostat Mesilate	Sodium Channel	52354	52070	53052	42314	36461	37808	1.00	0.96	1.02	0.99	0.86	0.93	0.99	*0.96*	0.96
Emodin	Dehydrogenase	53186	53235	56069	46015	47898	42002	1.01	1.04	1.06	1.02	1.04	0.94	1.04	1.00	0.96
Roxithromycin	Others	53901	48356	50542	41736	43487	42494	1.02	0.94	0.96	0.92	0.95	0.95	0.98	0.94	0.96
LY2584702 Tosylate	S6 Kinase	48534	51634	48378	40600	42888	41276	0.95	1.01	0.92	0.91	0.94	0.92	0.96	0.92	0.97
Gabexate Mesylate	Serine Protease	55155	59423	55210	45618	46740	46299	1.02	1.12	1.03	0.99	1.00	1.05	1.05	1.02	0.97
YM201636	PI3K	37051	36749	38212	31239	33830	31331	0.76	0.70	0.73	0.69	0.74	0.68	0.73	0.70	0.97
UNC1215	Epigenetic Reader Domain	53068	50618	50498	42833	40409	40428	1.04	0.96	0.96	0.98	0.93	0.96	0.99	0.95	0.97
AZD1080	GSK-3	62575	58371	56306	46457	44654	50957	1.23	1.11	1.07	1.06	1.03	1.20	1.14	1.10	0.97
Cetirizine DiHCl	Histamine Receptor	50864	51565	54182	41996	45842	46955	1.04	0.98	1.04	0.93	1.00	1.02	1.02	0.98	0.97
Altrenogest	Estrogen/progestogen Receptor	47858	53922	50436	40235	39663	37136	0.92	0.99	0.97	0.94	0.94	0.91	0.96	*0.93*	0.97
IWP-2	Wnt/beta-catenin	56172	55256	54544	42730	44504	45808	1.10	1.05	1.04	0.98	1.02	1.08	1.06	1.03	0.97
empty		52492	55085	55568	47027	46784	46549	1.07	1.04	1.06	1.04	1.02	1.01	1.06	1.02	0.97
Drospirenone	Estrogen/progestogen Receptor	45054	50252	47931	39582	42796	40813	0.92	0.95	0.92	0.88	0.94	0.88	0.93	0.90	0.97
Clorprenaline HCl	Adrenergic Receptor	48936	53782	55974	41826	41934	43390	0.96	1.02	1.06	0.96	0.96	1.03	1.02	0.98	0.97
AdipoRon	Others	55627	54057	48860	47146	42601	43526	1.08	1.05	0.93	1.05	0.94	0.97	1.02	0.99	0.97
6-Mercaptopurine (6-M	DNA/RNA Synthesis	46772	52100	45409	41161	39018	35637	0.92	0.99	0.86	0.94	0.90	0.84	0.92	0.89	0.97
Benzydamine HCl	Others	53923	56428	58567	45886	45230	44373	1.06	1.07	1.11	1.05	1.04	1.05	1.08	1.05	0.97
Benzethonium Chloride	AChR	50872	51818	53091	41991	41072	41893	1.00	0.99	1.01	0.96	0.94	0.99	1.00	0.96	0.97
Lenalidomide (CC-5013	TNF-alpha	48791	53873	50960	41266	45215	45773	1.00	1.02	0.98	0.91	0.99	0.99	1.00	0.97	0.97
CGP 57380	Others	49846	48367	50183	41454	41924	41339	0.97	0.94	0.95	0.93	0.92	0.92	0.96	0.92	0.97
INK 128 (MLN0128)	mTOR	10607	10546	10988	8449	7889	7917	0.20	0.19	0.21	0.20	0.19	0.19	0.20	*0.20*	0.97
Vemurafenib (PLX4032,	Raf	47376	49202	55513	42948	45889	42039	0.97	0.93	1.06	0.95	1.00	0.91	0.99	0.96	0.97
DASA-58	Others	55163	53043	54120	45940	46584	43972	1.08	1.04	1.03	1.03	1.02	0.98	1.05	1.01	0.97
PRX-08066 Maleic acid	5-HT Receptor	50538	48889	52106	46621	39782	40934	0.98	0.95	0.99	1.04	0.87	0.91	0.98	0.94	0.97
Vitamin C	Others	49214	50426	50943	41588	160	35210	0.94	0.93	0.98	0.97	0.00	0.87	0.95	*0.92*	0.97
Amlodipine	Calcium Channel	53131	54948	48419	44972	43114	40033	0.98	1.03	0.90	0.98	0.93	0.91	0.97	0.94	0.97
LY2409881	IκB/IKK	50755	45849	47451	42537	41497	37130	0.99	0.89	0.90	0.95	0.91	0.83	0.93	0.90	0.97
Neohesperidin	Others	54577	49191	54077	44432	43747	43943	1.04	0.96	1.02	0.98	0.95	0.99	1.01	0.97	0.97
GW788388	TGF-beta/Smad	52586	53825	57288	43873	39465	39575	1.01	0.99	1.10	1.02	0.93	0.97	1.03	*1.00*	0.97
Cabotegravir (GSK744,	Integrase	53589	54678	56835	45784	47271	45778	1.04	1.07	1.08	1.02	1.04	1.02	1.06	1.03	0.97
Entecavir Hydrate	Reverse Transcriptase	49405	45387	50594	39585	43015	42868	1.01	0.86	0.97	0.88	0.94	0.93	0.95	0.92	0.97
Ataluren (PTC124)	CFTR	46538	46657	52112	40000	39135	37547	0.91	0.89	0.99	0.91	0.90	0.89	0.93	0.90	0.97
Sodium ascorbate	Others	51502	50633	51414	44298	40883	38237	1.01	0.96	0.98	1.01	0.94	0.90	0.98	0.95	0.97
DMSO		50021	53126	52597	43945	43698	43348	0.97	1.04	1.00	0.98	0.96	0.97	1.00	0.97	0.97
Triamcinolone Acetonic	Glucocorticoid Receptor	54844	47571	49429	40192	42358	41612	1.01	0.89	0.92	0.88	0.91	0.95	0.94	0.91	0.97
Aztreonam	Others	46831	54763	50310	42584	45538	42617	0.96	1.04	0.96	0.94	1.00	0.92	0.99	0.95	0.97
AZ20	ATM/ATR	36856	41808	42998	32622	33180	31851	0.72	0.80	0.82	0.74	0.76	0.75	0.78	0.75	0.97
Triamcinolone	Glucocorticoid Receptor	49701	51885	47306	41587	38180	41983	0.92	0.98	0.88	0.91	0.82	0.96	0.92	0.89	0.97
Ascomycin (FK520)	Others	50395	50794	50830	41899	42898	43121	0.98	0.99	0.96	0.94	0.94	0.96	0.98	0.95	0.97
Conivaptan HCl	Vasopressin Receptor	57911	56415	59222	46196	49257	46615	1.07	1.06	1.10	1.01	1.06	1.06	1.08	1.04	0.97
Estradiol	Estrogen/progestogen Receptor	52406	50452	54365	41862	45585	41308	0.97	0.95	1.01	0.91	0.98	0.94	0.98	0.94	0.97
XL147	PI3K	49390	47572	52214	42934	44000	41745	1.01	0.90	1.00	0.95	0.96	0.90	0.97	0.94	0.97

NMS-E973	HSP (e.g. HSP90)	18893	18009	16499	13952	13682	15271	0.37	0.34	0.31	0.32	0.31	0.36	0.34	0.33	0.97
Nicotinic Acid	Others	56179	54769	55085	46762	46440	42800	1.04	1.03	1.03	1.02	1.00	0.97	1.03	1.00	0.97
DMSO		53551	57619	49288	46039	44319	44256	1.02	1.13	0.93	1.02	0.97	0.99	1.03	0.99	0.97
Clopidol	Others	58512	54586	58804	46092	47195	44914	1.15	1.04	1.12	1.05	1.08	1.06	1.10	1.07	0.97
Diltiazem HCl	Calcium Channel	51048	50166	54627	41517	41572	44367	0.94	0.94	1.02	0.90	0.89	1.01	0.97	0.94	0.97
Acadesine	AMPK	54339	53933	50090	43701	43450	42516	1.00	1.01	0.93	0.95	0.93	0.97	0.98	0.95	0.97
Mocetinostat (MGCD01	HDAC	36090	38673	39827	33731	30344	34701	0.74	0.73	0.76	0.75	0.66	0.75	0.75	0.72	0.97
Troxipide	Others	52488	56718	59414	45408	46758	43339	1.03	1.08	1.13	1.04	1.07	1.02	1.08	1.05	0.97
TG003	CDK	49950	48906	51513	38054	43348	39478	0.98	0.93	0.98	0.87	1.00	0.93	0.96	0.93	0.97
EPZ5676	Histone Methyltransferase	49568	49548	47540	40324	38615	38987	0.97	0.94	0.90	0.92	0.89	0.92	0.94	0.91	0.97
Esmolol HCl	Adrenergic Receptor	50016	51788	50972	41411	41745	36554	0.96	0.95	0.98	0.97	0.99	0.90	0.96	0.93	0.97
Telaprevir (VX-950)	HCV Protease	56922	58363	55627	48971	43292	47616	1.05	1.10	1.04	1.07	0.93	1.08	1.06	1.03	0.97
TIC10	Akt	45295	45971	44451	37626	36937	39734	0.88	0.90	0.84	0.84	0.81	0.89	0.87	0.85	0.97
INCB024360	IDO	53047	51703	49199	42374	43120	44242	1.03	1.01	0.93	0.95	0.95	0.99	0.99	0.96	0.97
（6-）ε-Aminocaproic	Others	55308	53646	51643	44565	44220	42818	1.02	1.01	0.96	0.97	0.95	0.97	1.00	0.97	0.97
Sulfamethoxypyridazine	Others	52391	52097	54732	44657	45074	38443	1.03	0.99	1.04	1.02	1.04	0.91	1.02	0.99	0.97
GSK3787	PPAR	50529	55296	53287	42306	46515	45250	0.98	1.08	1.01	0.95	1.02	1.01	1.02	0.99	0.97
Betamethasone	Glucocorticoid Receptor	47234	55837	48820	45484	44062	41338	0.97	1.06	0.94	1.01	0.96	0.90	0.99	0.96	0.97
DMSO		50764	50710	50633	44878	42360	40417	0.97	0.99	0.96	0.99	0.92	0.91	0.97	0.94	0.97
BRL-54443	5-HT Receptor	51110	52718	48699	40066	39168	37720	0.98	0.97	0.93	0.93	0.93	0.93	0.96	0.93	0.97
Flavopiridol HCl	CDK	15169	14356	12827	12776	12095	10697	0.29	0.28	0.24	0.28	0.26	0.24	0.27	0.26	0.97
Dapivirine (TMC120)	Reverse Transcriptase	47714	45416	45944	37392	36328	33636	0.91	0.84	0.88	0.87	0.86	0.83	0.88	0.85	0.97
Tepotinib (EMD 121406	c-Met	53402	52248	56210	45964	43071	41235	1.05	0.99	1.07	1.05	0.99	0.98	1.04	1.00	0.97
Cilomilast	PDE	49608	56725	54557	45961	46403	46347	1.02	1.08	1.05	1.02	1.01	1.00	1.05	1.01	0.97
Oxaliplatin	DNA/RNA Synthesis	46236	48212	51969	42891	42499	40951	0.95	0.91	1.00	0.95	0.93	0.89	0.95	0.92	0.97
XL388	mTOR	37341	36872	43774	30942	32222	31723	0.73	0.70	0.83	0.71	0.74	0.75	0.76	0.73	0.97
NMDA (N-Methyl-D-asp	GluR	53745	50720	51084	42547	41763	40947	1.06	0.97	0.97	0.97	0.96	0.97	1.00	0.97	0.97
CGS 21680 HCl	Adenosine Receptor	55165	54297	55173	43522	45819	45592	1.02	1.02	1.03	0.95	0.98	1.04	1.02	0.99	0.97
STF-083010	Others	50885	52148	49911	40231	46641	42144	0.99	1.02	0.95	0.90	1.03	0.94	0.99	0.95	0.97
Pentoxyverine Citrate	AChR	52585	55702	53931	43206	42721	44562	1.03	1.06	1.03	0.99	0.98	1.05	1.04	1.01	0.97
Fingolimod (FTY720) H	S1P Receptor	47615	48675	49954	38770	40357	38572	0.94	0.93	0.95	0.89	0.93	0.91	0.94	0.91	0.97
Reserpine	Others	60183	60160	52415	49364	44899	47342	1.11	1.13	0.98	1.08	0.96	1.08	1.07	1.04	0.97
AMG-900	Aurora Kinase	34748	31249	32734	25445	27949	24972	0.67	0.58	0.63	0.59	0.66	0.61	0.62	0.60	0.97
Motesanib Diphosphate	PDGFR,VEGFR,c-Kit	51212	48931	49647	42457	42458	44673	1.05	0.93	0.95	0.94	0.93	0.97	0.98	0.95	0.97
Methyl-Hesperidin	Others	54875	47288	48537	41807	40652	44000	1.04	0.92	0.92	0.92	0.89	0.99	0.96	0.93	0.97
Mometasone furoate	Glucocorticoid Receptor	48185	51391	48333	38995	41649	40679	0.89	0.97	0.90	0.85	0.89	0.93	0.92	0.89	0.97
Cinepazide maleate	Calcium Channel	48607	49583	53926	39014	40786	38647	0.93	0.91	1.04	0.91	0.97	0.95	0.96	0.93	0.97
empty		54462	56254	51743	44815	45224	43279	1.01	1.06	0.96	0.98	0.97	0.99	1.01	0.98	0.97
Sal003	Others	49498	50933	50020	42510	42010	42372	0.96	0.99	0.95	0.95	0.92	0.94	0.97	0.94	0.97
Fluvoxamine maleate	5-HT Receptor	48061	50469	52520	42918	44053	43565	0.98	0.96	1.01	0.95	0.96	0.94	0.98	0.95	0.97
Sodium 4-Aminosalicyl	NF-κB	48153	57377	51719	41539	39581	38744	0.92	1.06	0.99	0.97	0.94	0.95	0.99	0.96	0.97
Ginkgolide A	GABA Receptor	57055	54306	55459	45749	47068	44174	1.05	1.02	1.03	1.00	1.01	1.01	1.04	1.00	0.97
Ronidazole	Others	53125	54443	52819	41879	41973	40072	1.02	1.00	1.01	0.98	0.99	0.99	1.01	0.98	0.97
DMSO		50945	51923	50878	44645	43357	45012	1.04	0.98	0.97	0.99	0.95	0.98	1.00	0.97	0.97
Optovin	Others	51412	47725	49890	44067	40500	41206	1.00	0.93	0.95	0.99	0.89	0.92	0.96	0.93	0.97
Ethinyl Estradiol	Estrogen/progestogen Receptor	57195	58338	55564	48311	45580	46575	1.06	1.10	1.03	1.05	0.98	1.06	1.06	1.03	0.97
AG-18	EGFR	50857	52815	54681	45114	43480	45031	0.99	1.03	1.04	1.01	0.96	1.00	1.02	0.99	0.97
Lopinavir	HIV Protease	51779	55050	54137	44206	48782	46221	1.06	1.04	1.04	0.98	1.07	1.00	1.05	1.02	0.97
GSK2578215A	LRRK2	47663	49923	48614	43216	39153	41046	0.93	0.97	0.92	0.97	0.86	0.91	0.94	0.91	0.97
Norethindrone	Others	56067	54305	55979	45482	606	39697	1.07	1.00	1.07	1.06	0.01	0.98	1.05	1.02	0.97
AZD3839	BACE	48178	46757	47916	38348	37486	44760	0.94	0.91	0.91	0.86	0.82	1.00	0.92	0.89	0.97
Nithiamide	Others	52424	55000	50573	44126	43157	40172	1.03	1.05	0.96	1.01	0.99	0.95	1.01	0.98	0.97
Smoothened Agonist (S	Hedgehog/Smoothened	48792	50816	49565	41761	38790	45389	0.95	0.99	0.94	0.93	0.85	1.01	0.96	0.93	0.97
DMSO		50007	55158	50755	42777	45225	46906	1.02	1.05	0.97	0.95	0.99	1.02	1.01	0.98	0.97
Pyrimethamine	DHFR	54855	48997	53808	44604	43130	41782	1.01	0.92	1.00	0.97	0.93	0.95	0.98	0.95	0.97
Fluocinolone Acetonide	Glucocorticoid Receptor	48074	44743	47151	40232	39211	38275	0.91	0.87	0.89	0.89	0.85	0.86	0.89	0.87	0.97
Chloramphenicol	Others	53669	51286	52115	45577	42303	41193	0.99	0.96	0.97	0.99	0.91	0.94	0.97	0.95	0.97
SP600125	JNK	49025	51583	51894	42312	44346	45319	1.00	0.98	0.99	0.94	0.97	0.98	0.99	0.96	0.97
Nelarabine	DNA/RNA Synthesis	50277	48522	52369	46957	43535	40346	1.03	0.92	1.00	1.04	0.95	0.87	0.98	0.96	0.97
Sildenafil Citrate	PDE	51767	54755	55547	44804	48039	47409	1.06	1.04	1.06	0.99	1.05	1.03	1.05	1.02	0.97
AM251	Cannabinoid Receptor	48569	52469	50179	38298	41080	38962	0.93	0.97	0.96	0.89	0.97	0.96	0.95	0.93	0.97
PI-1840	Proteasome	56138	51760	53527	45402	44512	46480	1.09	1.01	1.01	1.02	0.98	1.04	1.04	1.01	0.97
Sulfameter	DHFR	61546	51855	58626	45817	47472	47961	1.14	0.97	1.09	1.00	1.02	1.09	1.07	1.04	0.97
PR-619	DUB	48533	51353	55153	40685	41224	43043	0.95	0.98	1.05	0.93	0.95	1.02	0.99	0.96	0.97
Ferulic Acid	Others	55432	49290	49797	42752	42708	44524	1.05	0.96	0.94	0.95	0.93	1.00	0.99	0.96	0.97
Calcitriol	Others	49489	50126	49151	41377	40911	46631	1.01	0.95	0.94	0.92	0.89	1.01	0.97	0.94	0.97
Haloperidol	Others	50939	57105	56666	44380	48012	43140	0.94	1.07	1.05	0.97	1.03	0.98	1.02	0.99	0.97
Clozapine	5-HT Receptor	56567	49688	50909	45180	44358	42719	1.08	0.97	0.96	1.00	0.97	0.96	1.00	0.97	0.97

Gallamine Triethiodide	AChR	53533	53379	50173	47267	42023	42951	1.02	1.04	0.95	1.04	0.92	0.96	1.00	0.97	0.97
Buflomedil HCl	Others	51537	52682	51679	45178	43329	42742	0.98	1.03	0.98	1.00	0.94	0.96	1.00	0.97	0.97
Pyrazinamide	Others	54857	52634	55273	42867	48242	42781	1.01	0.99	1.03	0.93	1.04	0.97	1.01	0.98	0.97
Mosapride Citrate	5-HT Receptor	47341	55988	53199	42984	45889	46517	0.97	1.06	1.02	0.95	1.00	1.01	1.02	0.99	0.97
OG-L002	Histone Demethylase	54849	53248	51187	44090	40741	43792	1.08	1.01	0.97	1.01	0.94	1.04	1.02	0.99	0.97
Vinorelbine Tartrate	Microtubule Associated	12485	13415	14113	10030	10969	11264	0.25	0.26	0.27	0.23	0.25	0.27	0.26	0.25	0.97
Atracurium Besylate	Others	57081	53933	52882	45791	43767	45175	1.05	1.01	0.98	1.00	0.94	1.03	1.02	0.99	0.97
DMSO		52129	55060	50831	43873	45809	38045	1.02	1.05	0.97	1.00	1.05	0.90	1.01	0.98	0.97
Methocarbamol	Carbonic Anhydrase	56706	54670	58015	44307	49503	45585	1.05	1.03	1.08	0.97	1.06	1.04	1.05	1.02	0.97
Aloxistatin	Cysteine Protease	53644	51665	53188	43908	45263	44903	1.05	1.01	1.01	0.98	1.00	1.00	1.02	0.99	0.97
empty		47049	49082	52437	41720	42795	44202	0.96	0.93	1.00	0.92	0.94	0.96	0.97	0.94	0.97
Chlorprothixene	Dopamine Receptor	51972	48730	53435	39092	46238	41526	0.96	0.92	0.99	0.85	0.99	0.95	0.96	0.93	0.97
DMSO		49745	49678	53349	40532	42438	40400	0.98	0.95	1.02	0.93	0.98	0.96	0.98	0.95	0.97
Telotristat Etiprate (LX	Hydroxylase	50480	52650	53752	44656	44057	43482	0.96	1.03	1.02	0.99	0.96	0.97	1.00	0.97	0.97
DMSO		51641	55270	52597	41300	44251	43233	1.01	1.05	1.00	0.94	1.02	1.02	1.02	0.99	0.97
empty		49899	53331	52320	41652	39575	38059	0.96	0.98	1.00	0.97	0.94	0.94	0.98	0.95	0.97
Ritodrine HCl	Adrenergic Receptor	51687	50619	48579	45247	42041	39912	0.98	0.99	0.92	1.00	0.92	0.89	0.96	0.94	0.97
Broxyquinoline	Others	58647	57744	53450	45397	50139	41840	1.15	1.10	1.02	1.04	1.15	0.99	1.09	1.06	0.97
Tenoxicam	Others	48523	48002	51395	41831	41907	40926	0.92	0.94	0.97	0.92	0.91	0.92	0.94	0.92	0.97
Nitenpyram	AChR	54794	50245	50062	39310	42626	43409	1.08	0.96	0.95	0.90	0.98	1.03	0.99	0.97	0.97
Arecoline	AChR	57865	55223	51850	46994	46288	45792	1.10	1.08	0.98	1.04	1.01	1.03	1.05	1.02	0.97
Tie2 kinase inhibitor	Tie-2	58681	58902	58043	50642	48432	45614	1.08	1.11	1.08	1.10	1.04	1.04	1.09	1.06	0.97
Enalaprilat Dihydrate	RAAS	57191	55550	53235	47769	43204	45616	1.06	1.04	0.99	1.04	0.93	1.04	1.03	1.00	0.97
Voriconazole	P450 (e.g. CYP17)	50867	56024	54701	45622	47271	47222	1.04	1.06	1.05	1.01	1.03	1.02	1.05	1.02	0.97
GSK503	Histone Methyltransferase	51816	51060	52151	42641	43664	44990	1.01	1.00	0.99	0.95	0.96	1.00	1.00	0.97	0.97
Caffeic Acid Phenethyl I	NF-κB	44840	45780	47437	39103	37160	40618	0.87	0.89	0.90	0.87	0.82	0.90	0.89	0.87	0.97
ENMD-2076	Aurora Kinase,FLT3,VEGFR	24328	22901	23211	20505	19835	20881	0.50	0.43	0.44	0.45	0.43	0.45	0.46	0.45	0.97
Telbivudine	Reverse Transcriptase	51920	53647	52224	44993	43832	41262	0.96	1.01	0.97	0.98	0.94	0.94	0.98	0.95	0.97
QNZ (EVP4593)	NF-κB,TNF-alpha	44894	46975	43093	33789	37626	37672	0.88	0.89	0.82	0.77	0.86	0.89	0.87	0.84	0.97
Formestane	Aromatase	55470	55711	50851	47540	44120	45085	1.05	1.09	0.96	1.05	0.96	1.01	1.03	1.01	0.97
FH1(BRD-K4477)	Others	57291	56006	51791	48338	44473	47098	1.12	1.09	0.98	1.08	0.98	1.05	1.06	1.04	0.97
TPCA-1	IκB/IKK	44137	48783	45450	37083	36480	33897	0.85	0.90	0.87	0.86	0.86	0.83	0.87	0.85	0.97
AZD7762	Chk	30149	27491	26714	22251	22468	24694	0.56	0.52	0.50	0.48	0.48	0.56	0.52	0.51	0.97
VU 0364439	GluR	56692	51062	52861	44785	45358	45981	1.10	1.00	1.00	1.00	1.00	1.02	1.03	1.01	0.97
Cyclo(RGDyK)	Integrin	51961	46454	50778	41899	40920	43571	1.01	0.91	0.96	0.94	0.90	0.97	0.96	0.94	0.97
LDK378	ALK	48582	47950	45766	40752	38134	36351	0.95	0.91	0.87	0.93	0.88	0.86	0.91	0.89	0.97
BG45	HDAC	48021	50273	49428	41445	40698	43041	0.94	0.98	0.94	0.93	0.89	0.96	0.95	0.93	0.97
Sodium Nitrite	Others	49042	55723	51553	42129	39721	38082	0.94	1.03	0.99	0.98	0.94	0.94	0.99	0.96	0.97
CCG-1423	Rho	49636	46759	51051	39374	43400	42195	0.97	0.91	0.97	0.88	0.95	0.94	0.95	0.92	0.97
Daidzein	Others	55311	55990	51961	45734	43890	44955	1.02	1.05	0.97	1.00	0.94	1.02	1.01	0.99	0.97
Atazanavir Sulfate	HIV Protease	51424	54092	52157	43110	46383	47508	1.05	1.03	1.00	0.96	1.01	1.03	1.03	1.00	0.97
Articaine HCl	Others	48627	53264	51379	39454	37564	39160	0.93	0.98	0.99	0.92	0.89	0.96	0.97	0.94	0.97
Clobetasol propionate	Glucocorticoid Receptor	50173	47120	51407	42413	44146	39052	0.95	0.92	0.97	0.94	0.96	0.88	0.95	0.93	0.97
Mitotane	Others	55390	50747	50486	42862	44663	41696	1.02	0.95	0.94	0.93	0.96	0.95	0.97	0.95	0.97
DMSO		54744	58463	52966	44141	44721	41174	1.05	1.08	1.02	1.03	1.06	1.01	1.05	1.02	0.98
Penfluridol	Dopamine Receptor	41224	46395	50224	38574	34846	38074	0.81	0.88	0.96	0.88	0.80	0.90	0.88	0.86	0.98
Alprostadil	Others	49314	55297	53373	46633	46194	44311	1.01	1.05	1.02	1.03	1.01	0.96	1.03	1.00	0.98
Tenofovir	Reverse Transcriptase	45971	57389	50500	42063	44943	46482	0.94	1.09	0.97	0.93	0.98	1.01	1.00	0.97	0.98
BMS-707035	Integrase	45983	51710	51941	40303	42519	47140	0.94	0.98	1.00	0.89	0.93	1.02	0.97	0.95	0.98
Aloe-emodin	Others	56279	56161	53130	47595	47081	45298	1.07	1.10	1.01	1.05	1.03	1.02	1.06	1.03	0.98
GW2580	CSF-1R	50020	50553	53153	45569	40717	44064	0.97	0.99	1.01	1.02	0.90	0.98	0.99	0.97	0.98
UPF 1069	PARP	52071	55347	55432	48124	44907	45123	1.01	1.08	1.05	1.08	0.99	1.01	1.05	1.02	0.98
Carprofen	COX	54646	52788	50965	42430	42591	43346	1.07	1.00	0.97	0.97	0.98	1.02	1.02	0.99	0.98
Ethionamide	Others	55870	53965	51840	43600	44574	45208	1.03	1.01	0.96	0.95	0.96	1.03	1.00	0.98	0.98
DMSO		52230	52284	56101	44583	46816	44358	0.99	1.02	1.06	0.99	1.02	0.99	1.03	1.00	0.98
Betulinic acid	Topoisomerase	48646	51688	51239	44096	36695	34014	0.93	0.95	0.98	1.03	0.87	0.84	0.96	0.93	0.98
Kinetin	Others	50562	51474	49630	42723	41636	43855	0.96	1.01	0.94	0.94	0.91	0.98	0.97	0.95	0.98
RO4929097	Gamma-secretase,Beta Amyloid	57961	59415	58814	52261	44825	48326	1.07	1.12	1.09	1.14	0.96	1.10	1.09	1.07	0.98
Cefdinir	Others	61317	58670	53541	48660	47559	47048	1.13	1.10	1.00	1.06	1.02	1.07	1.08	1.05	0.98
Chenodeoxycholic Acid	Others	53030	48705	56488	43001	43422	44157	0.98	0.92	1.05	0.94	0.93	1.01	0.98	0.96	0.98
Caspofungin Acetate	Others	44411	48304	47334	37888	33843	34145	0.85	0.89	0.91	0.88	0.80	0.84	0.88	0.86	0.98
1-Hexadecanol	Others	52710	52871	53744	44361	43379	41467	1.04	1.01	1.02	1.01	1.00	0.98	1.02	1.00	0.98
CEP-32496	CSF-1R,Raf	56706	50785	57840	46282	46070	47968	1.11	0.99	1.10	1.03	1.01	1.07	1.06	1.04	0.98
Ramelteon	MT Receptor	49617	52974	53457	44015	46463	45235	1.02	1.00	1.02	0.98	1.02	0.98	1.01	0.99	0.98
Sparfloxacin	Others	58780	54162	52194	45565	46053	44774	1.09	1.02	0.97	0.99	0.99	1.02	1.02	1.00	0.98
AC480 (BMS-599626)	HER2,EGFR	44016	49168	49172	41823	40713	41178	0.90	0.93	0.94	0.93	0.89	0.89	0.93	0.90	0.98
Tranilast	Others	48347	55731	54638	49507	43806	44518	0.99	1.06	1.05	1.10	0.96	0.96	1.03	1.01	0.98
SF1670	Others	54670	54618	57016	45739	44950	44168	1.07	1.04	1.08	1.04	1.03	1.04	1.07	1.04	0.98

Trometamol	Others	56338	54784	56675	45908	45885	44322	1.11	1.04	1.08	1.05	1.05	1.05	1.08	1.05	0.98
Rutin	Others	56443	53784	53749	49493	45097	44145	1.07	1.05	1.02	1.09	0.98	0.99	1.05	1.02	0.98
DMSO		49835	55112	55745	47422	44798	47481	1.02	1.04	1.07	1.05	0.98	1.03	1.04	1.02	0.98
Ketoprofen	COX	56949	55322	55789	46989	45534	46310	1.05	1.04	1.04	1.02	0.98	1.05	1.04	1.02	0.98
GSK1838705A	ALK,IGF-1R	45409	48333	43408	39367	38219	38542	0.86	0.94	0.82	0.87	0.83	0.86	0.88	0.86	0.98
Omecamtiv mecarbil ((	ATPase	52723	50781	54480	47759	42267	43605	1.00	0.99	1.03	1.06	0.92	0.98	1.01	0.98	0.98
Sulfisoxazole	Others	54226	53348	51224	44905	40921	45278	1.00	1.00	0.95	0.98	0.88	1.03	0.99	0.96	0.98
ABT-199 (GDC-0199)	Bcl-2	54189	48905	48349	44498	41132	43082	1.06	0.95	0.92	1.00	0.90	0.96	0.98	0.95	0.98
Mubritinib (TAK 165)	HER2	48392	45505	51643	42023	43059	38087	0.92	0.89	0.98	0.93	0.94	0.85	0.93	0.91	0.98
Idarubicin HCl	Topoisomerase	5573	6291	6872	5693	5204	5381	0.11	0.12	0.13	0.13	0.11	0.12	0.12	0.12	0.98
Diclazuril	Others	59180	61134	60524	47685	52343	49507	1.09	1.15	1.13	1.04	1.12	1.13	1.12	1.10	0.98
ARN-509	Androgen Receptor	49380	48711	51116	40779	40776	36120	0.95	0.90	0.98	0.95	0.97	0.89	0.94	0.92	0.98
MGCD-265	c-Met,Tie-2,VEGFR	47368	51984	51589	43142	45512	42693	0.97	0.99	0.99	0.96	0.99	0.93	0.98	0.96	0.98
Rutaecarpine	Others	50294	50919	52236	45456	43869	40633	0.96	0.99	0.99	1.00	0.96	0.91	0.98	0.96	0.98
VX-809 (Lumacaftor)	CFTR	63054	54642	59055	49458	48840	47799	1.16	1.03	1.10	1.08	1.05	1.09	1.10	1.07	0.98
Risperidone	5-HT Receptor	57613	62032	56589	50591	48301	46904	1.06	1.17	1.05	1.10	1.04	1.07	1.09	1.07	0.98
DMSO		53945	57471	52114	45506	45716	41585	1.06	1.09	0.99	1.04	1.05	0.98	1.05	1.02	0.98
Spectinomycin HCl	Others	55009	49536	49062	41602	43639	44802	1.05	0.97	0.93	0.92	0.95	1.00	0.98	0.96	0.98
GSK1292263	GPR	59252	56647	53338	45511	46888	47507	1.09	1.06	0.99	0.99	1.01	1.08	1.05	1.03	0.98
INH6	Microtubule Associated	49460	52945	43612	41139	42288	40888	0.96	1.03	0.83	0.92	0.93	0.91	0.94	0.92	0.98
Terbinafine HCl	Others	50777	50826	50568	45079	43326	40530	0.97	0.99	0.96	1.00	0.94	0.91	0.97	0.95	0.98
AGI-6780	Dehydrogenase	50490	51930	51637	45284	40791	39087	0.99	0.99	0.98	1.03	0.94	0.92	0.99	0.97	0.98
Sodium salicylate	NF-κB	51792	51242	53002	40573	40977	39769	0.99	0.94	1.02	0.95	0.97	0.98	0.98	0.96	0.98
INO-1001	PARP	51600	53172	51838	47161	44495	44812	1.06	1.01	0.99	1.05	0.97	0.97	1.02	1.00	0.98
empty		54571	51611	52857	45942	44463	44852	1.06	1.01	1.00	1.03	0.98	1.00	1.02	1.00	0.98
Aminophylline	PDE	53890	55332	56274	47893	43058	45911	1.00	1.04	1.05	1.04	0.93	1.05	1.03	1.00	0.98
Etomidate	GABA Receptor	47977	54376	53163	42533	47558	45308	0.98	1.03	1.02	0.94	1.04	0.98	1.01	0.99	0.98
XAV-939	Wnt/beta-catenin	45286	49234	50092	40813	42313	42830	0.93	0.93	0.96	0.90	0.93	0.93	0.94	0.92	0.98
Almotriptan Malate	5-HT Receptor	53280	54604	53964	46265	42347	45237	0.98	1.03	1.00	1.01	0.91	1.03	1.00	0.98	0.98
BQU57	Others	48882	47857	49475	43299	40156	40899	0.95	0.93	0.94	0.97	0.88	0.91	0.94	0.92	0.98
Quizartinib (AC220)	FLT3	57310	54612	57437	46601	47613	45967	1.06	1.03	1.07	1.02	1.02	1.05	1.05	1.03	0.98
RG-7112	Mdm2	45945	48404	47896	37529	38249	39720	0.90	0.92	0.91	0.86	0.88	0.94	0.91	0.89	0.98
Arbidol HCl	Others	54446	52860	52932	45364	44652	42679	1.01	0.99	0.99	0.99	0.96	0.97	0.99	0.97	0.98
DMSO		54165	51214	53578	42301	40337	39696	1.04	0.94	1.03	0.99	0.96	0.98	1.00	0.98	0.98
Ozagrel HCl	P450 (e.g. CYP17)	59041	58167	52890	48360	43559	48770	1.09	1.09	0.98	1.05	0.94	1.11	1.06	1.03	0.98
Pilocarpine HCl	AChR	50985	52800	50373	41870	40917	42490	1.00	1.00	0.96	0.96	0.94	1.00	0.99	0.97	0.98
Iloperidone	5-HT Receptor	49239	55904	55413	47129	45548	47204	1.01	1.06	1.06	1.04	1.00	1.02	1.04	1.02	0.98
Nilvadipine	Calcium Channel	52776	51420	55424	42312	38641	40047	1.01	0.95	1.06	0.99	0.92	0.99	1.01	0.99	0.98
Orotic acid (6-Carboxyu	Others	51072	50653	53086	44347	42955	43971	0.97	0.99	1.00	0.98	0.94	0.99	0.99	0.97	0.98
Aspartame	Others	59331	53351	53582	46166	45793	45710	1.10	1.00	1.00	1.01	0.98	1.04	1.03	1.01	0.98
Etravirine (TMC125)	Reverse Transcriptase	50427	52586	45263	40191	176	36272	0.97	0.97	0.87	0.94	0.00	0.89	0.93	0.92	0.98
Flurbiprofen	Others	49501	48756	52863	40809	42937	41443	0.91	0.92	0.98	0.89	0.92	0.94	0.94	0.92	0.98
Amiodarone HCl	Potassium Channel,Autophagy	51959	54584	50505	44080	45378	40797	0.96	1.03	0.94	0.96	0.98	0.93	0.98	0.95	0.98
Thalidomide	E3 Ligase ,TNF-alpha	50636	54951	54641	46798	45888	47057	1.04	1.04	1.05	1.04	1.00	1.02	1.04	1.02	0.98
Amonafide	Topoisomerase	46489	52363	53677	45112	46962	40815	0.95	0.99	1.03	1.00	1.03	0.88	0.99	0.97	0.98
Temozolomide	Autophagy	47151	51844	49427	39740	45552	44239	0.97	0.98	0.95	0.88	1.00	0.96	0.97	0.95	0.98
Aniracetam	AMPA Receptor-kainate Receptor-NMDA R	47256	46289	50449	43574	41853	40278	0.97	0.88	0.97	0.97	0.91	0.87	0.94	0.92	0.98
Isradipine	Calcium Channel	56962	55207	51009	50049	43644	41638	1.05	1.04	0.95	1.09	0.94	0.95	1.01	0.99	0.98
CNX-774	BTK	52035	48278	42715	37607	40561	38303	1.02	0.92	0.81	0.86	0.93	0.91	0.92	0.90	0.98
TGX-221	PI3K	45279	44076	45406	39242	37874	40644	0.93	0.84	0.87	0.87	0.83	0.88	0.88	0.86	0.98
empty		55671	54347	53922	46567	43483	45748	1.03	1.02	1.00	1.01	0.93	1.04	1.02	1.00	0.98
Sulfamethazine	Others	49730	49684	50795	39559	41903	37952	0.95	0.92	0.98	0.92	0.99	0.93	0.95	0.93	0.98
Choline Chloride	Others	52546	53264	58128	44742	43030	45531	1.03	1.01	1.11	1.02	0.99	1.08	1.05	1.03	0.98
Metoprolol Tartrate	Adrenergic Receptor	49628	50440	44152	41977	39267	38355	0.92	0.95	0.82	0.91	0.84	0.87	0.90	0.88	0.98
Sunitinib	VEGFR,PDGFR,c-Kit	52078	49960	55221	49039	42492	42389	1.01	0.97	1.05	1.10	0.93	0.94	1.01	0.99	0.98
Bikinin	GSK-3	48161	46766	47599	40589	42367	38542	0.94	0.91	0.90	0.91	0.93	0.86	0.92	0.90	0.98
Ibuprofen	COX	56659	57827	59811	48157	51049	45429	1.05	1.09	1.11	1.05	1.10	1.03	1.08	1.06	0.98
BRD7552	Others	50007	50794	44297	43984	37586	42153	0.97	0.99	0.84	0.98	0.83	0.94	0.94	0.92	0.98
Nitrendipine	Calcium Channel,Autophagy	49430	46958	48001	39927	41859	40790	0.94	0.92	0.91	0.88	0.91	0.91	0.92	0.90	0.98
empty		53286	54512	50752	41073	43547	44375	1.05	1.04	0.97	0.94	1.00	1.05	1.02	1.00	0.98
Monomethyl auristatin	Others	13954	13850	12679	12471	11857	10204	0.27	0.27	0.24	0.28	0.26	0.23	0.26	0.26	0.98
SB-3CT	MMP	54897	52694	50203	47775	42231	44523	1.07	1.03	0.95	1.07	0.93	0.99	1.02	1.00	0.98
Maraviroc	CCR	57514	52701	54018	44173	47284	44724	1.06	0.99	1.01	0.96	1.02	1.02	1.02	1.00	0.98
Guaifenesin	Others	58970	53956	52102	46358	45579	44884	1.09	1.01	0.97	1.01	0.98	1.02	1.02	1.00	0.98
Nitazoxanide	Others	54797	57322	55067	45535	44464	48518	1.01	1.08	1.02	0.99	0.96	1.10	1.04	1.02	0.98
Quinine HCl Dihydrate	Potassium Channel	53602	43766	51864	43976	39879	42692	1.02	0.85	0.98	0.97	0.87	0.96	0.95	0.93	0.98
Mevastatin	HMG-CoA Reductase	49293	51100	50427	38645	41877	42179	0.97	0.97	0.96	0.88	0.96	1.00	0.97	0.95	0.98
CFTRinh-172	CFTR	51700	51283	50106	40263	40841	43476	1.02	0.98	0.95	0.92	0.94	1.03	0.98	0.96	0.98

10058-F4	c-Myc	57009	52316	51837	44241	45389	41721	1.12	1.00	0.99	1.01	1.04	0.99	1.03	1.01	0.98
DMSO		50178	49745	48730	41184	41213	44379	0.98	0.97	0.92	0.92	0.91	0.99	0.96	0.94	0.98
R547	CDK	35527	34773	40128	32371	31109	30262	0.68	0.68	0.76	0.72	0.68	0.68	0.70	0.69	0.98
Apigenin	P450 (e.g. CYP17)	54307	56381	49486	43720	47033	45406	1.03	1.10	0.94	0.97	1.02	1.02	1.02	1.00	0.98
DMSO		58849	60156	54602	46233	49083	43392	1.13	1.11	1.05	1.08	1.16	1.07	1.09	1.07	0.98
Danoprevir (ITMN-191)	HCV Protease	52568	58128	55084	41932	48942	54026	1.08	1.10	1.06	0.93	1.07	1.17	1.08	1.06	0.98
Nabumetone	COX	53050	50157	53423	42796	167	38213	1.02	0.92	1.03	1.00	0.00	0.94	0.99	0.97	0.98
Chrysin	Others	53220	57882	52508	46976	47725	44436	1.01	1.13	0.99	1.04	1.04	1.00	1.05	1.02	0.98
Demeclocycline HCl	Others	53608	51066	52595	42587	41683	43816	1.05	0.97	1.00	0.97	0.96	1.04	1.01	0.99	0.98
SKLB1002	VEGFR	50505	56135	50162	47117	40066	40569	0.99	1.07	0.95	1.08	0.92	0.96	1.00	0.99	0.98
Levodropropizine	Histamine Receptor	58015	51244	55452	46365	43460	44431	1.14	0.98	1.06	1.06	1.00	1.05	1.06	1.04	0.98
AG-1024	IGF-1R	51065	47784	52393	43031	46352	42982	1.05	0.91	1.00	0.95	1.01	0.93	0.99	0.97	0.98
GF109203X	PKC	54422	52702	52223	43347	43880	42665	1.07	1.00	0.99	0.99	1.01	1.01	1.02	1.00	0.98
Gemfibrozil	PPAR	55825	57009	57385	49266	45880	46177	1.03	1.07	1.07	1.07	0.99	1.05	1.06	1.04	0.98
4-Aminohippuric Acid	Others	49346	48662	49676	40520	36088	35901	0.95	0.90	0.95	0.94	0.85	0.88	0.93	0.91	0.98
Glyburide	Potassium Channel	55563	52203	54443	47764	42535	44316	1.03	0.98	1.01	1.04	0.91	1.01	1.01	0.99	0.98
WAY-100635 Maleate	5-HT Receptor	58501	53045	52933	48521	47045	44308	1.11	1.04	1.00	1.07	1.03	0.99	1.05	1.03	0.98
Deflazacort	Glucocorticoid Receptor	52224	49633	45156	42029	39966	40101	0.96	0.93	0.84	0.92	0.86	0.91	0.91	0.90	0.98
BIRB 796 (Doramapimc	p38 MAPK	58976	57785	55535	48337	44377	50217	1.09	1.09	1.03	1.05	0.95	1.14	1.07	1.05	0.98
Hydrocortisone	Glucocorticoid Receptor	51365	51630	46746	46120	41834	36641	0.95	0.97	0.87	1.00	0.90	0.83	0.93	0.91	0.98
P22077	DUB	47954	53337	49990	42322	41079	39957	0.94	1.01	0.95	0.97	0.94	0.94	0.97	0.95	0.98
Tiotropium Bromide hy	AChR	49294	52256	55069	45613	46811	40920	0.94	1.02	1.04	1.01	1.02	0.92	1.00	0.98	0.98
E3330	DNA/RNA Synthesis	51159	55044	50555	44748	44654	44592	1.00	1.07	0.96	1.00	0.98	0.99	1.01	0.99	0.98
E-64	Cysteine Protease	50082	54539	52428	43023	43237	47957	0.98	1.06	0.99	0.96	0.95	1.07	1.01	0.99	0.98
17-AAG (Tanespimycin)	HSP (e.g. HSP90)	15229	15656	17544	16021	12558	13775	0.31	0.30	0.34	0.36	0.27	0.30	0.31	0.31	0.98
Flufenamic acid	Others	51423	52357	52682	41521	44595	41568	1.01	1.00	1.00	0.95	1.02	0.98	1.00	0.99	0.98
DMSO		57553	57312	56338	45427	50765	46250	1.06	1.08	1.05	0.99	1.09	1.05	1.06	1.04	0.98
Curcumin	Others	52583	53099	50270	43689	42409	43546	0.97	1.00	0.94	0.95	0.91	0.99	0.97	0.95	0.98
GW9508	GPR	56669	54682	51388	46179	46198	46833	1.10	1.07	0.97	1.03	1.02	1.04	1.05	1.03	0.98
Esomeprazole Magnesi	Proton Pump	54680	55275	50695	47156	40470	45847	1.01	1.04	0.94	1.03	0.87	1.04	1.00	0.98	0.98
Bambuterol HCl	Adrenergic Receptor	53384	50918	52782	43738	41422	43101	1.05	0.97	1.00	1.00	0.95	1.02	1.01	0.99	0.98
Fasudil (HA-1077) HCl	ROCK,Autophagy	57564	57035	56750	50267	50862	41667	1.06	1.07	1.06	1.10	1.09	0.95	1.06	1.05	0.98
VX-680 (Tozasertib, MK	Aurora Kinase	44812	45026	44950	35084	41364	41786	0.92	0.85	0.86	0.78	0.90	0.91	0.88	0.86	0.98
Combretastatin A4	Microtubule Associated	14515	14921	14881	12442	12492	12965	0.28	0.29	0.28	0.28	0.27	0.29	0.29	0.28	0.98
TAE226 (NVP-TAE226)	FAK	38723	36742	38320	29745	28997	29187	0.74	0.68	0.74	0.69	0.69	0.72	0.72	0.71	0.98
Cyproheptadine HCl	Histamine Receptor	49880	56101	54349	44114	45006	44311	0.92	1.05	1.01	0.96	0.97	1.01	1.00	0.98	0.98
Mycophenolate Mofetil	Dehydrogenase	26375	31693	27406	24696	26780	23313	0.54	0.60	0.53	0.55	0.59	0.51	0.56	0.55	0.98
Crizotinib (PF-0234106(	ALK,c-Met	41583	43992	42806	32986	38885	40728	0.85	0.83	0.82	0.73	0.85	0.88	0.84	0.82	0.98
Benzbromarone	P450 (e.g. CYP17)	50296	52546	56610	43591	42131	44421	0.99	1.00	1.08	1.00	0.97	1.05	1.02	1.00	0.98
MK-2206 2HCl	Akt	39031	42357	39860	36699	34368	35161	0.80	0.80	0.76	0.81	0.75	0.76	0.79	0.78	0.98
Sotagliflozin (LX4211)	SGLT	57139	52766	52487	46973	44216	47771	1.11	1.03	1.00	1.05	0.97	1.06	1.05	1.03	0.98
TG101348 (SAR302503	JAK	44526	45895	45441	36715	34487	33723	0.85	0.85	0.87	0.86	0.82	0.83	0.86	0.84	0.98
H 89 2HCl	PKA,S6 Kinase	55248	53976	57471	47431	47147	44257	1.02	1.01	1.07	1.03	1.01	1.01	1.03	1.02	0.98
VX-222 (VCH-222, Lomi	HCV Protease	47920	53003	53423	44485	46076	44652	0.98	1.01	1.02	0.99	1.01	0.97	1.00	0.99	0.98
RI-1	Others	52638	53895	54042	43713	46537	47206	1.03	1.05	1.02	0.98	1.02	1.05	1.03	1.02	0.98
Ivabradine HCl	Adrenergic Receptor	59434	58721	56221	49489	46775	48851	1.10	1.10	1.05	1.08	1.01	1.11	1.08	1.07	0.98
Adapalene	Retinoid Receptor	48995	54191	52903	43600	44450	48786	1.00	1.03	1.01	0.97	0.97	1.06	1.01	1.00	0.98
Geldanamycin	HSP (e.g. HSP90),Autophagy	16113	15620	15886	14505	13648	12462	0.31	0.31	0.30	0.32	0.30	0.28	0.30	0.30	0.98
Prednisolone Acetate	Glucocorticoid Receptor	50239	46319	45865	39003	40461	41941	0.96	0.90	0.87	0.86	0.88	0.94	0.91	0.89	0.98
Akti-1/2	Akt	39477	43028	42516	34917	39538	32631	0.77	0.84	0.81	0.78	0.87	0.73	0.81	0.79	0.98
Clorsulon	Others	53476	53656	50096	47179	44156	42821	1.02	1.05	0.95	1.04	0.96	0.96	1.00	0.99	0.98
Indacaterol Maleate	Adrenergic Receptor	49050	49205	43008	39133	37879	34162	0.94	0.91	0.83	0.91	0.90	0.84	0.89	0.88	0.98
UM729	AhR	48701	48371	49452	40121	41842	43519	0.95	0.94	0.94	0.90	0.92	0.97	0.94	0.93	0.98
empty		48339	50769	49811	39455	42936	39374	0.95	0.97	0.95	0.90	0.99	0.93	0.95	0.94	0.98
Chlorpromazine HCl	Dopamine Receptor,Potassium Channel	47978	49646	51085	43625	44654	38681	0.91	0.97	0.97	0.96	0.97	0.87	0.95	0.93	0.98
Prednisolone	Glucocorticoid Receptor	52111	49984	50253	46589	42967	37571	0.96	0.94	0.94	1.02	0.92	0.86	0.95	0.93	0.98
Clinofibrate	HMG-CoA Reductase	56612	53628	53568	46891	47864	45043	1.08	1.05	1.01	1.04	1.04	1.01	1.05	1.03	0.98
PLX-4720	Raf	45911	50583	52513	43712	42068	44876	0.94	0.96	1.01	0.97	0.92	0.97	0.97	0.95	0.98
GW3965 HCl	Liver X Receptor	59529	49571	53802	46583	47825	44556	1.13	0.97	1.02	1.03	1.04	1.00	1.04	1.02	0.98
Carteolol HCl	Adrenergic Receptor	49405	55693	51104	44612	42185	40978	0.97	1.06	0.97	1.02	0.97	0.97	1.00	0.99	0.98
VE-822	ATM/ATR	48818	46863	42040	38498	37732	36555	0.96	0.89	0.80	0.88	0.87	0.86	0.88	0.87	0.98
NVP-BSK805 2HCl	JAK	54675	49200	53876	46600	45745	42271	1.04	0.96	1.02	1.03	1.00	0.95	1.01	0.99	0.99
Etizolam	Others	49978	52416	51103	39532	44912	41125	0.98	1.00	0.97	0.90	1.03	0.97	0.98	0.97	0.99
PFK15	Others	51464	54109	55702	44553	45409	41997	1.01	1.03	1.06	1.02	1.04	0.99	1.03	1.02	0.99
IOWH032	CFTR	49000	56103	48291	39757	42119	43521	0.96	1.07	0.92	0.91	0.97	1.03	0.98	0.97	0.99
Gynostemma Extract	Others	50241	49355	50364	43485	40219	44233	0.96	0.96	0.95	0.96	0.88	0.99	0.96	0.94	0.99
CHIR-124	Chk	54831	49733	43917	42933	42068	41787	1.04	0.97	0.83	0.95	0.92	0.94	0.95	0.93	0.99
DMSO		53562	54379	53852	44855	44145	43415	1.05	1.03	1.02	1.02	1.01	1.03	1.04	1.02	0.99

Celecoxib	COX	49170	51453	52471	46252	44132	43998	1.01	0.98	1.01	1.03	0.96	0.95	**1.00**	**0.98**	0.99
Valproic acid sodium sa	GABA Receptor,HDAC,Autophagy	50255	54188	59212	44026	51682	47922	1.03	1.03	1.13	0.98	1.13	1.04	**1.06**	**1.05**	0.99
Lidocaine	Histamine Receptor	49960	52962	52171	46575	45006	44585	1.02	1.00	1.00	1.03	0.98	0.97	**1.01**	**0.99**	0.99
Zafirlukast	Others	55199	57135	56708	45562	46861	48456	1.02	1.07	1.06	0.99	1.01	1.10	**1.05**	**1.03**	0.99
Avanafil	PDE	51675	50114	52789	42146	159	38191	0.99	0.92	1.01	0.98	0.00	0.94	**0.98**	***0.96***	0.99
S3I-201	STAT	48534	51055	52367	42190	46463	44812	0.99	0.97	1.00	0.94	1.02	0.97	**0.99**	**0.97**	0.99
Suplatast Tosylate	Others	59860	56104	57508	51273	47836	45616	1.11	1.05	1.07	1.12	1.03	1.04	**1.08**	**1.06**	0.99
Artemisinin	Others	49240	46988	53372	42532	43675	45307	1.01	0.89	1.02	0.94	0.95	0.98	**0.97**	**0.96**	0.99
Chlorocresol	Others	51159	52392	54085	41156	43268	44530	1.00	1.00	1.03	0.94	0.99	1.05	**1.01**	**1.00**	0.99
Flavoxate HCl	AChR	51687	53803	51355	43584	149	37927	0.99	0.99	0.99	1.02	0.00	0.93	**0.99**	***0.98***	0.99
Hexestrol	Estrogen/progestogen Receptor	49235	49698	49858	45157	40249	41656	0.94	0.97	0.94	1.00	0.88	0.93	**0.95**	**0.94**	0.99
Propafenone HCl	Sodium Channel	53889	50720	51494	45979	44450	42892	1.02	0.99	0.97	1.02	0.97	0.96	**1.00**	**0.98**	0.99
Tolcapone	Transferase	49877	51427	54126	41759	179	38986	0.96	0.95	1.04	0.97	0.00	0.96	**0.98**	***0.97***	0.99
Cysteamine HCl	Others	52541	52295	57196	46977	43209	42517	1.03	1.00	1.09	1.07	0.99	1.01	**1.04**	**1.02**	0.99
Repaglinide	Potassium Channel	53890	54259	55673	43901	53246	46914	1.10	1.03	1.07	0.97	1.16	1.02	**1.07**	**1.05**	0.99
LDC1267	TAM Receptor	44433	46288	41473	38676	37724	37065	0.87	0.90	0.79	0.86	0.83	0.83	**0.85**	**0.84**	0.99
Rivastigmine Tartrate	AChR	57035	57825	56724	47120	51009	45179	1.05	1.09	1.06	1.03	1.10	1.03	**1.07**	**1.05**	0.99
ML141	Rho	53774	49725	50395	40282	44619	47198	1.05	0.97	0.96	0.90	0.98	1.05	**0.99**	**0.98**	0.99
empty		50808	49849	50128	45178	43386	40826	0.99	0.97	0.95	1.01	0.95	0.91	**0.97**	**0.96**	0.99
Anacardic Acid	Histone Acetyltransferase	52364	46929	45252	39516	43919	40718	1.02	0.92	0.86	0.88	0.97	0.91	**0.93**	**0.92**	0.99
Quercetin	Sirtuin,PI3K,Src,PKC	50195	50780	48879	46620	41559	39954	0.95	0.99	0.92	1.03	0.91	0.90	**0.96**	**0.94**	0.99
IEM 1754 dihydrobroMi	GluR	46370	49902	48034	35935	39796	38902	0.89	0.92	0.92	0.84	0.94	0.96	**0.91**	***0.90***	0.99
Epiandrosterone	Androgen Receptor,Estrogen/progestogen	52521	50780	52721	42537	40020	38636	1.01	0.94	1.01	0.99	0.95	0.95	**0.98**	***0.97***	0.99
Ganciclovir	Others	53335	52367	52095	44546	43820	43372	0.98	0.98	0.97	0.97	0.94	0.99	**0.98**	**0.97**	0.99
Thiazovivin	ROCK	47682	52245	52323	41754	47228	44841	0.98	0.99	1.00	0.93	1.03	0.97	**0.99**	**0.98**	0.99
Nicorandil	Potassium Channel	52564	49638	50555	41900	42881	42719	0.97	0.93	0.94	0.91	0.92	0.97	**0.95**	**0.94**	0.99
SSR128129E	FGFR	49066	53360	50718	43470	40690	41337	0.96	1.02	0.96	0.99	0.94	0.98	**0.98**	**0.97**	0.99
Ribavirin	Others	54092	52145	54209	51137	42799	43217	1.03	1.02	1.03	1.13	0.93	0.97	**1.02**	**1.01**	0.99
Bleomycin Sulfate	DNA/RNA Synthesis	25270	27879	27951	23431	22692	25161	0.52	0.53	0.54	0.52	0.50	0.55	**0.53**	**0.52**	0.99
Cilostazol	PDE	51108	53464	56950	48879	49188	43887	1.05	1.01	1.09	1.08	1.08	0.95	**1.05**	**1.04**	0.99
A-1210477	Bcl-2	50664	49663	54526	43598	44984	44323	0.99	0.97	1.03	0.97	0.99	0.99	**1.00**	**0.98**	0.99
DMSO		57393	55801	55659	48415	46443	43689	1.13	1.06	1.06	1.11	1.07	1.03	**1.08**	**1.07**	0.99
Pomalidomide	TNF-alpha	58159	54927	56246	45753	44968	50489	1.07	1.03	1.05	1.00	0.97	1.15	**1.05**	**1.04**	0.99
DMSO		54239	52122	49743	43886	43278	43200	1.00	0.98	0.93	0.96	0.93	0.98	**0.97**	**0.96**	0.99
Berberine HCl	Others	45900	46178	47358	38968	37739	42486	0.87	0.90	0.90	0.86	0.82	0.95	**0.89**	**0.88**	0.99
AZD3514	Androgen Receptor	50465	52167	54464	43119	44618	41084	0.99	0.99	1.04	0.98	1.03	0.97	**1.01**	**0.99**	0.99
Megestrol Acetate	Androgen Receptor,Estrogen/progestogen	44635	45270	50528	39541	42210	41806	0.91	0.86	0.97	0.88	0.92	0.91	**0.91**	**0.90**	0.99
URB597	FAAH	54229	50465	52838	44657	45161	44895	1.03	0.99	1.00	0.99	0.98	1.01	**1.01**	**0.99**	0.99
Purvalanol A	CDK	46803	48138	50484	42183	42060	40627	0.91	0.94	0.96	0.94	0.92	0.91	**0.94**	**0.92**	0.99
GSK1324726A (I-BET72	Epigenetic Reader Domain	29742	32419	29174	24810	26861	26832	0.58	0.63	0.55	0.55	0.59	0.60	**0.59**	**0.58**	0.99
Tilmicosin	Others	53321	53731	51129	41897	44525	40352	1.02	0.99	0.98	0.98	1.05	0.99	**1.00**	***0.99***	0.99
Fenspiride HCl	PDE	54587	49936	54575	43373	39672	39517	1.05	0.92	1.05	1.01	0.94	0.97	**1.00**	***0.99***	0.99
Mestranol	Estrogen/progestogen Receptor	60468	56726	57165	49753	48851	47127	1.12	1.07	1.06	1.08	1.05	1.07	**1.08**	**1.07**	0.99
Methscopolamine	AChR	52733	53000	54716	45813	44630	43674	0.97	1.00	1.02	1.00	0.96	0.99	**1.00**	**0.98**	0.99
Methimazole	Others	52686	54907	54832	48344	43395	44034	0.97	1.03	1.02	1.05	0.93	1.00	**1.01**	**1.00**	0.99
Tolazoline HCl	Adrenergic Receptor	50146	52995	51356	46154	40878	39788	0.98	1.01	0.98	1.05	0.94	0.94	**0.99**	**0.98**	0.99
PP1	Src	44032	45655	49705	37572	39013	37735	0.86	0.87	0.95	0.86	0.90	0.89	**0.89**	**0.88**	0.99
WZ4002	EGFR	48102	48507	53757	47110	44720	40472	0.98	0.92	1.03	1.04	0.98	0.88	**0.98**	**0.97**	0.99
empty		51918	51945	50154	41374	48813	45600	1.06	0.98	0.96	0.92	1.07	0.99	**1.00**	**0.99**	0.99
LY2603618	Chk	50606	53499	50757	45069	43992	43560	0.96	1.04	0.96	1.00	0.96	0.98	**0.99**	**0.98**	0.99
LDN-57444	DUB	49600	48996	49475	41140	38380	41954	0.97	0.93	0.94	0.94	0.88	0.99	**0.95**	**0.94**	0.99
BTB06584	ATPase	52032	53914	46694	40324	45494	45538	1.01	1.05	0.89	0.90	1.00	1.01	**0.98**	**0.97**	0.99
Acetylcholine Chloride	AChR	51379	49063	51811	42785	42879	41640	0.95	0.92	0.96	0.93	0.92	0.95	**0.95**	**0.93**	0.99
TAK-438	Potassium Channel	52296	57255	51725	46292	48042	44397	1.02	1.12	0.98	1.04	1.06	0.99	**1.04**	**1.03**	0.99
CPI-360	Histone Methyltransferase	50911	48902	52768	42613	42171	46335	0.99	0.95	1.00	0.95	0.93	1.03	**0.98**	**0.97**	0.99
6-Thio-dG	DNA/RNA Synthesis	49548	45132	45582	43255	37488	39829	0.97	0.88	0.86	0.97	0.82	0.89	**0.90**	**0.89**	0.99
Ro-3306	CDK	45159	50183	50191	41681	41778	41648	0.88	0.98	0.95	0.93	0.92	0.93	**0.94**	**0.93**	0.99
LEE011	CDK	51695	49121	54824	43641	46439	43717	1.01	0.96	1.04	0.98	1.02	0.97	**1.00**	**0.99**	0.99
empty		51552	51399	54526	44044	47757	43112	0.98	1.00	1.03	0.97	1.04	0.97	**1.01**	**0.99**	0.99
Deferasirox	Others	52693	50314	48649	42217	42698	41938	0.97	0.95	0.91	0.92	0.92	0.95	**0.94**	**0.93**	0.99
Sulfacetamide Sodium	Autophagy	50742	53021	53821	41039	40558	40975	0.97	0.98	1.03	0.96	0.96	1.01	**0.99**	***0.98***	0.99
ZSTK474	PI3K	50116	51118	58738	46743	45787	48369	1.03	0.97	1.13	1.04	1.00	1.05	**1.04**	**1.03**	0.99
Dibutyryl-cAMP (Buclad	PKA	47321	50733	48179	41732	39811	44233	0.92	0.99	0.91	0.93	0.88	0.99	**0.94**	**0.93**	0.99
Mitoxantrone	Topoisomerase	14094	12318	12789	11315	9926	11503	0.26	0.23	0.24	0.25	0.21	0.26	**0.24**	**0.24**	0.99
Fluoxetine HCl	5-HT Receptor	48014	51928	52571	42357	45805	46237	0.98	0.98	1.01	0.94	1.00	1.00	**0.99**	**0.98**	0.99
Veliparib (ABT-888)	PARP	50284	51692	58181	44053	45344	51799	1.03	0.98	1.11	0.98	0.99	1.12	**1.04**	**1.03**	0.99
WH-4-023	Src	49389	50113	54645	44714	40680	47143	0.96	0.98	1.04	1.00	0.89	1.05	**0.99**	**0.98**	0.99
Gliclazide	Potassium Channel	51124	49138	50708	45376	41126	42852	0.97	0.96	0.96	1.00	0.90	0.96	**0.96**	**0.95**	0.99

LCZ696	RAAS	46656	48913	48203	39760	40259	43706	0.91	0.95	0.91	0.89	0.89	0.97	0.93	0.92	0.99
L-carnitine	Others	54315	49966	45242	43950	41820	42414	1.03	0.98	0.86	0.97	0.91	0.95	0.95	0.94	0.99
MPEP	GluR	51432	54530	52967	44123	40393	38776	0.99	1.01	1.02	1.03	0.96	0.95	1.00	0.99	0.99
(-)-p-Bromotetramisole	Others	54782	51844	49114	44155	42794	47155	1.07	1.01	0.93	0.99	0.94	1.05	1.00	0.99	0.99
UNC0379	Histone Methyltransferase	51898	53142	51023	44239	44754	45377	1.01	1.04	0.97	0.99	0.98	1.01	1.01	0.99	0.99
Ampicillin sodium	Others	49890	48430	49624	37936	38771	39123	0.96	0.89	0.95	0.88	0.92	0.96	0.93	0.92	0.99
TSU-68 (SU6668, Orant	PDGFR,FGFR,VEGFR	49542	50952	48856	43125	44563	44106	1.01	0.97	0.94	0.96	0.97	0.96	0.97	0.96	0.99
Osthole	Others	51428	51077	47983	41379	42870	44791	0.98	1.00	0.91	0.91	0.93	1.00	0.96	0.95	0.99
Indapamide	Others	56011	56641	54457	49215	46201	44594	1.03	1.06	1.01	1.07	0.99	1.02	1.04	1.03	0.99
empty		44371	49104	51673	43678	40988	43203	0.91	0.93	0.99	0.97	0.90	0.94	0.94	0.93	0.99
IU1	DUB	52291	53214	52110	42118	43793	43650	1.03	1.01	0.99	0.96	1.01	1.03	1.01	1.00	0.99
Edoxaban	Factor Xa	50149	51468	56170	45399	45203	39201	0.98	0.98	1.07	1.04	1.04	0.93	1.01	1.00	0.99
Aciclovir	Others	52525	50097	49953	41269	44980	41604	0.97	0.94	0.93	0.90	0.97	0.95	0.95	0.94	0.99
empty		53983	51902	55787	44482	45812	45132	1.00	0.98	1.04	0.97	0.98	1.03	1.00	0.99	0.99
IOX1	Histone Demethylase	53552	58276	57062	46598	48790	43536	1.05	1.11	1.09	1.06	1.12	1.03	1.08	1.07	0.99
Astragaloside A	Others	49755	47188	47461	44729	42351	36889	0.95	0.92	0.90	0.99	0.92	0.83	0.92	0.91	0.99
LY2874455	VEGFR,FGFR	11549	13308	11635	11316	9185	9523	0.23	0.25	0.22	0.26	0.21	0.23	0.23	0.23	0.99
SGI-1776 free base	Pim	50046	45669	44367	38285	40622	41294	0.95	0.89	0.84	0.85	0.89	0.93	0.89	0.89	0.99
Ranolazine	Calcium Channel	50876	50646	55193	46301	45111	40091	0.94	0.95	1.03	1.01	0.97	0.91	0.97	0.96	0.99
Dp44mT	Others	16364	15211	15778	13393	13889	13528	0.32	0.30	0.30	0.30	0.31	0.30	0.30	0.30	0.99
K-Ras(G12C) inhibitor 6	Rho	54393	50965	50601	44685	47382	42414	1.06	0.99	0.96	1.00	1.04	0.94	1.00	1.00	0.99
Hesperidin	Others	51115	50728	48127	42755	40870	45074	0.97	0.99	0.91	0.95	0.89	1.01	0.96	0.95	0.99
17-DMAG (Alvespimyci	HSP (e.g. HSP90)	17260	17935	20521	17310	15807	16022	0.35	0.34	0.39	0.38	0.35	0.35	0.36	0.36	0.99
20-Hydroxyecdysone	Others	51067	46886	47778	42057	41516	41494	0.97	0.92	0.90	0.93	0.90	0.93	0.93	0.92	0.99
Vincristine	Autophagy,Microtubule Associated	10744	12729	12916	11610	10152	10287	0.22	0.24	0.25	0.26	0.22	0.22	0.24	0.23	0.99
SB590885	Raf	61833	56732	64842	55920	52246	49238	1.18	1.11	1.23	1.24	1.14	1.10	1.17	1.16	0.99
Venlafaxine	5-HT Receptor	56149	55249	54511	50258	51013	45351	1.15	1.05	1.04	1.11	1.12	0.98	1.08	1.07	0.99
empty		48277	46489	50972	40978	44517	39670	0.92	0.91	0.96	0.91	0.97	0.89	0.93	0.92	0.99
Kartogenin	TGF-beta/Smad	45567	49867	44244	40145	39551	40794	0.89	0.97	0.84	0.90	0.87	0.91	0.90	0.89	0.99
Palmatine chloride	Others	50659	42654	50628	40972	40619	41889	0.96	0.83	0.96	0.91	0.89	0.94	0.92	0.91	0.99
Nitrofural	Others	52593	55195	51061	41578	44183	47354	0.97	1.04	0.95	0.91	0.95	1.08	0.99	0.98	0.99
Tacrolimus (FK506)	mTOR	45699	46521	48333	39796	38361	37586	0.90	0.89	0.92	0.91	0.88	0.89	0.90	0.89	0.99
DMSO		50229	50563	51279	42521	38174	37004	0.96	0.93	0.98	0.99	0.90	0.91	0.96	0.95	0.99
NVP-ADW742	IGF-1R	43045	51145	47263	39869	39168	45829	0.88	0.97	0.91	0.88	0.86	0.99	0.92	0.91	0.99
Quinapril HCl	RAAS	49377	51548	51534	45993	43538	41500	0.94	1.01	0.98	1.02	0.95	0.93	0.97	0.97	0.99
Nalidixic acid	Topoisomerase	51109	50048	50536	45865	40979	43442	0.97	0.98	0.96	1.01	0.89	0.97	0.97	0.96	0.99
Oxaprozin	COX	55808	53041	52251	43255	46158	43358	1.10	1.01	0.99	0.99	1.06	1.03	1.03	1.02	0.99
DMSO		52555	51345	53468	45380	46824	43044	1.00	1.00	1.01	1.00	1.02	0.97	1.00	1.00	0.99
BMS-794833	VEGFR,c-Met	53251	53224	49610	45616	41550	46927	1.01	1.04	0.94	1.01	0.91	1.05	1.00	0.99	0.99
Dabigatran Etexilate	Thrombin	57184	51069	53115	46034	44833	44509	1.06	0.96	0.99	1.00	0.96	1.01	1.00	0.99	0.99
A922500	Transferase	56181	51024	54384	47482	46432	44904	1.07	1.00	1.03	1.05	1.01	1.01	1.03	1.02	0.99
HJC0350	Others	53883	49697	46154	43159	43460	42694	1.05	0.97	0.88	0.97	0.96	0.95	0.97	0.96	0.99
MK-2048	Integrase	53217	51068	53402	41937	43799	40485	1.02	0.94	1.03	0.98	1.04	1.00	1.00	0.99	0.99
empty		50369	52971	55852	44395	46628	46330	0.98	1.03	1.06	0.99	1.03	1.03	1.02	1.02	0.99
Pilaralisib (XL147)	PI3K	46116	41770	45355	40967	37464	36545	0.90	0.82	0.86	0.92	0.82	0.81	0.86	0.85	0.99
empty		50483	56995	56666	51417	45948	47570	1.03	1.08	1.09	1.14	1.00	1.03	1.07	1.06	0.99
Polydatin	Phospholipase (e.g. PLA)	46863	49755	46287	41309	38856	42709	0.89	0.97	0.88	0.91	0.85	0.96	0.91	0.91	0.99
DMSO		51045	52600	51644	47345	45349	44656	1.05	1.00	0.99	1.05	0.99	0.97	1.01	1.00	0.99
BMS-536924	IGF-1R	33354	37734	38168	32495	33665	30328	0.68	0.72	0.73	0.72	0.74	0.66	0.71	0.70	0.99
Erdosteine	Others	53567	50143	51117	46185	39981	43765	0.99	0.94	0.95	1.01	0.86	1.00	0.96	0.95	0.99
empty		44746	49563	52047	43617	41967	43732	0.92	0.94	1.00	0.97	0.92	0.95	0.95	0.94	0.99
Elacridar (GF120918)	P-gp	48595	49452	50821	44940	40728	42831	0.95	0.97	0.96	1.00	0.90	0.95	0.96	0.95	0.99
Bergapten	Others	50153	49174	49621	42214	39705	40918	0.98	0.94	0.94	0.96	0.91	0.97	0.95	0.95	0.99
Serotonin HCl	5-HT Receptor	50029	51780	49020	42365	40840	41171	0.98	0.99	0.93	0.97	0.94	0.97	0.97	0.96	0.99
GDC-0980 (RG7422)	mTOR,PI3K	34120	32960	29308	26204	26911	29794	0.65	0.64	0.55	0.58	0.59	0.67	0.62	0.61	0.99
Ruxolitinib (INCB01842	JAK	46029	49373	51182	42719	43152	43767	0.94	0.94	0.98	0.95	0.94	0.95	0.95	0.95	0.99
DMSO		57659	53188	56646	48457	48245	44138	1.06	1.00	1.05	1.06	1.04	1.00	1.04	1.03	0.99
empty		56632	55824	56836	45593	50232	46496	1.05	1.05	1.06	0.99	1.08	1.06	1.05	1.04	0.99
AZD3264	IκB/IKK	47713	51576	51335	44713	41403	43989	0.93	1.01	0.97	1.00	0.91	0.98	0.97	0.96	0.99
Nepicastat (SYN-117) H	Hydroxylase	54975	53483	51231	47632	44886	44934	1.05	1.04	0.97	1.05	0.98	1.01	1.02	1.01	0.99
Dicloxacillin Sodium	Others	52525	54087	54897	43275	43913	41236	1.01	1.00	1.05	1.01	1.04	1.02	1.02	1.01	0.99
Rolipram	PDE	50736	53968	53102	45450	45106	49157	1.04	1.02	1.02	1.01	0.99	1.07	1.03	1.02	0.99
(-)-Blebbistatin	ATPase	53193	51528	51967	43941	44940	40523	1.04	0.98	0.99	1.00	1.03	0.96	1.00	1.00	0.99
Allopurinol	Others	55455	54967	49534	43895	43724	46736	1.02	1.03	0.92	0.96	0.94	1.06	0.99	0.99	0.99
AT13148	Akt,ROCK,PKA,S6 Kinase	45338	53406	45468	42622	41661	40448	0.88	1.04	0.86	0.95	0.92	0.90	0.93	0.92	0.99
empty		52398	51368	51832	45364	42032	47075	1.02	1.00	0.98	1.01	0.92	1.05	1.00	1.00	0.99
Nafamostat Mesylate	Serine Protease	50649	51489	53886	45126	43691	49424	1.04	0.98	1.03	1.00	0.96	1.07	1.02	1.01	0.99
Felodipine	Calcium Channel	56040	54389	54828	44256	48091	46596	1.03	1.02	1.02	0.96	1.03	1.06	1.03	1.02	0.99

Everolimus (RAD001)	mTOR	26801	29722	30137	26298	23376	27015	0.55	0.56	0.58	0.58	0.51	0.59	0.56	0.56	0.99
Alizapride HCl	Dopamine Receptor	50967	50522	48199	39305	41555	42701	1.00	0.96	0.92	0.90	0.95	1.01	0.96	0.95	0.99
VE-821	ATM/ATR	52340	46126	53068	45129	42106	43740	1.02	0.90	1.01	1.01	0.93	0.97	0.98	0.97	0.99
Lactulose	Others	51341	51306	54286	45876	48044	45179	1.05	0.97	1.04	1.02	1.05	0.98	1.02	1.02	0.99
Isotretinoin	Hydroxylase	44142	49383	51539	40998	43593	43835	0.90	0.94	0.99	0.91	0.95	0.95	0.94	0.94	0.99
AZD6738	ATM/ATR	48560	49364	47043	39231	42287	43954	0.95	0.96	0.89	0.88	0.93	0.98	0.93	0.93	0.99
Rosiglitazone HCl	PPAR	61363	54518	53464	47783	48534	46191	1.13	1.02	1.00	1.04	1.04	1.05	1.05	1.05	0.99
DMSO		50060	48039	49221	40700	42858	40466	0.92	0.90	0.92	0.89	0.92	0.92	0.91	0.91	0.99
Flumazenil	GABA Receptor	48371	48817	57068	43695	44294	48760	0.99	0.93	1.09	0.97	0.97	1.06	1.00	1.00	0.99
Eletriptan HBr	5-HT Receptor	47581	52988	52032	40762	40181	39164	0.91	0.98	1.00	0.95	0.95	0.96	0.96	*0.96*	0.99
empty		50026	47036	45050	37546	39793	36913	0.96	0.87	0.86	0.88	0.94	0.91	0.90	*0.89*	0.99
Sulfasalazine	Others	56985	57226	58126	48343	46608	50040	1.05	1.08	1.08	1.05	1.00	1.14	1.07	1.06	1.00
Phenothiazine	Dopamine Receptor	47968	52207	49259	40280	41662	41538	0.94	0.99	0.94	0.92	0.96	0.98	0.96	0.95	1.00
Nisoldipine	Calcium Channel	54523	60149	55883	48871	47882	46975	1.01	1.13	1.04	1.06	1.03	1.07	1.06	1.05	1.00
Ibutilide Fumarate	Sodium Channel	58184	55344	53386	48540	43989	47943	1.07	1.04	0.99	1.06	0.95	1.09	1.04	1.03	1.00
ID-8	Others	48152	48110	47490	37671	41930	39269	0.95	0.92	0.90	0.86	0.96	0.93	0.92	0.92	1.00
empty		50316	52096	51270	38628	48477	45567	0.96	1.02	0.97	0.85	1.06	1.02	0.98	0.98	1.00
Montelukast Sodium	Others	52594	51458	55104	45507	42903	43272	1.03	0.98	1.05	1.04	0.99	1.02	1.02	1.02	1.00
Aprepitant	Substance P	44995	51454	50903	43079	43148	44413	0.92	0.98	0.98	0.95	0.94	0.96	0.96	0.95	1.00
DMSO		54635	53070	53867	42752	44314	42020	1.05	0.98	1.03	1.00	1.05	1.03	1.02	*1.02*	1.00
Tioconazole	Others	53723	54623	56879	46212	46971	46098	0.99	1.03	1.06	1.01	1.01	1.05	1.03	1.02	1.00
Clofibric Acid	PPAR	54077	51114	50242	44711	43390	40681	1.06	0.97	0.96	1.02	1.00	0.96	1.00	0.99	1.00
CYT387	JAK	50245	43983	47761	40205	42103	40238	0.96	0.86	0.90	0.89	0.92	0.90	0.91	0.90	1.00
PF-04217903	c-Met	53629	52519	51390	45914	48120	46064	1.10	1.00	0.98	1.02	1.05	1.00	1.03	1.02	1.00
DMSO		52280	55365	50660	46666	46337	40705	0.97	1.04	0.94	1.02	1.00	0.93	0.98	0.98	1.00
AM1241	Cannabinoid Receptor	49458	53394	51435	46444	42891	40915	0.91	1.00	0.96	1.01	0.92	0.93	0.96	0.96	1.00
T0070907	PPAR	47721	47474	47995	40364	37957	34936	0.91	0.88	0.92	0.94	0.90	0.86	0.90	*0.90*	1.00
Trichlormethiazide	Others	51088	54184	48588	42251	43147	44344	0.94	1.02	0.90	0.92	0.93	1.01	0.96	0.95	1.00
Floxuridine	DNA/RNA Synthesis	54101	47942	48977	42286	48369	43895	1.11	0.91	0.94	0.94	1.06	0.95	0.99	0.98	1.00
MI-2 (Menin-MLL Inhibi	Histone Methyltransferase	46427	47850	46214	39886	40181	41810	0.90	0.93	0.88	0.89	0.88	0.93	0.90	0.90	1.00
Fenoprofen Calcium	Others	57717	51901	49463	45451	45915	42879	1.07	0.98	0.92	0.99	0.99	0.98	0.99	0.98	1.00
Ampicillin Trihydrate	Others	56151	51722	50626	44182	42934	44237	1.10	0.98	0.96	1.01	0.99	1.05	1.02	1.01	1.00
Tigecycline	Others	50126	49735	55391	47682	45570	44709	1.03	0.94	1.06	1.06	1.00	0.97	1.01	1.01	1.00
Mesna	Others	55885	51882	51570	45404	45657	43426	1.03	0.98	0.96	0.99	0.98	0.99	0.99	0.99	1.00
CID755673	Others	51325	52193	49810	43396	41528	42124	1.01	0.99	0.95	0.99	0.95	1.00	0.98	0.98	1.00
Naftopidil DiHCl	Adrenergic Receptor	48849	47750	50700	41520	45104	44443	1.00	0.91	0.97	0.92	0.99	0.96	0.96	0.96	1.00
Doxifluridine	Others	54590	56880	53071	46913	46766	45251	1.01	1.07	0.99	1.02	1.00	1.03	1.02	1.02	1.00
empty		52519	56055	55986	50218	45426	46597	1.00	1.09	1.06	1.11	0.99	1.04	1.05	1.05	1.00
Methylprednisolone	Glucocorticoid Receptor	48244	48977	49308	40693	41483	41484	0.89	0.92	0.92	0.89	0.89	0.94	0.91	0.91	1.00
Odanacatib (MK-0822)	Cysteine Protease	52748	51605	52223	45167	47126	47067	1.08	0.98	1.00	1.00	1.03	1.02	1.02	1.02	1.00
WYE-354	mTOR	47148	48654	51336	44056	42591	44144	0.97	0.92	0.98	0.98	0.93	0.96	0.96	0.95	1.00
Asenapine	Adrenergic Receptor,5-HT Receptor	47817	51943	52002	48138	44339	42296	0.98	0.98	1.00	1.07	0.97	0.92	0.99	0.98	1.00
TG101209	JAK,FLT3,c-RET	41135	43118	38106	35291	38290	32350	0.78	0.84	0.72	0.78	0.83	0.73	0.78	0.78	1.00
Teriflunomide	Dehydrogenase	49547	49076	48748	38961	40966	42166	0.97	0.93	0.93	0.89	0.94	1.00	0.94	0.94	1.00
Sodium Tauroursodeoxy	Others	48398	51251	48293	43311	40356	44768	0.94	1.00	0.92	0.97	0.89	1.00	0.95	0.95	1.00
Ritonavir	HIV Protease	50617	52038	50421	44920	44055	47255	1.04	0.99	0.97	1.00	0.96	1.02	1.00	0.99	1.00
EI1	Histone Methyltransferase	55205	47695	52757	44837	44200	46079	1.08	0.93	1.00	1.00	0.97	1.03	1.00	1.00	1.00
Cefoperazone	Others	60303	55282	60819	49927	48634	50305	1.11	1.04	1.13	1.09	1.05	1.15	1.09	1.09	1.00
Sorafenib Tosylate	VEGFR,Raf,PDGFR	48947	55525	54594	47035	47598	46705	1.00	1.05	1.05	1.04	1.04	1.01	1.03	1.03	1.00
OSI-906 (Linsitinib)	IGF-1R	40300	44928	48357	40429	39367	38857	0.83	0.85	0.93	0.90	0.86	0.84	0.87	0.87	1.00
Sitafloxacin Hydrate	Others	59458	56918	58104	50951	45129	51114	1.10	1.07	1.08	1.11	0.97	1.16	1.08	1.08	1.00
Bendamustine HCl	DNA/RNA Synthesis	45050	51467	55238	44639	44821	45314	0.92	0.98	1.06	0.99	0.98	0.98	0.99	0.98	1.00
Vildagliptin (LAF-237)	DPP-4	43844	50740	45943	38277	37099	35576	0.84	0.94	0.88	0.89	0.88	0.88	0.89	*0.88*	1.00
Cyclamic acid	Others	46272	50258	50070	40793	39213	36356	0.89	0.93	0.96	0.95	0.93	0.90	0.92	*0.92*	1.00
Nebivolol	Adrenergic Receptor	54520	56035	56316	49553	43552	47774	1.01	1.05	1.05	1.08	0.94	1.09	1.04	1.03	1.00
empty		47743	54892	52496	45865	44865	47145	0.98	1.04	1.01	1.02	0.98	1.02	1.01	1.01	1.00
GSK621	AMPK	48765	50540	48446	43337	42435	42630	0.95	0.99	0.92	0.97	0.93	0.95	0.95	0.95	1.00
Probucol	Others	55584	55527	56050	47046	47086	47123	1.03	1.04	1.04	1.03	1.01	1.07	1.04	1.04	1.00
empty		47136	49394	49639	42656	45069	42385	0.97	0.94	0.95	0.95	0.99	0.92	0.95	0.95	1.00
empty		55404	49243	48268	43497	45679	43796	1.08	0.96	0.92	0.97	1.00	0.98	0.99	0.98	1.00
Chlormezanone	Others	53904	58179	51516	42546	46468	49157	1.00	1.09	0.96	0.93	1.00	1.12	1.02	1.01	1.00
KU-0063794	mTOR	46103	48768	48735	44529	42550	40722	0.94	0.92	0.93	0.99	0.93	0.88	0.93	0.93	1.00
ERK5-IN-1	ERK	51845	47257	49741	43469	42565	43333	1.01	0.92	0.94	0.97	0.94	0.97	0.96	0.96	1.00
Piroxicam	COX	58344	59100	56768	51684	47565	48051	1.08	1.11	1.06	1.13	1.02	1.09	1.08	1.08	1.00
A-769662	AMPK	53305	47076	52706	44051	44079	44320	1.01	0.92	1.00	0.97	0.96	0.99	0.98	0.98	1.00
Mecarbinate	Others	58220	58784	53774	46865	47744	49702	1.07	1.10	1.00	1.02	1.03	1.13	1.06	1.06	1.00
TMP269	HDAC	54779	50157	49670	40733	42469	45129	1.08	0.95	0.95	0.93	0.98	1.07	0.99	0.99	1.00
Alfacalcidol	Others	48628	53511	53442	43461	50007	45039	1.00	1.01	1.02	0.96	1.09	0.98	1.01	1.01	1.00

AZD3463	ALK	39526	34657	35616	28733	31097	31380	0.78	0.66	0.68	0.66	0.71	0.74	**0.70**	**0.70**	1.00
Ponatinib (AP24534)	Bcr-Abl,PDGFR,VEGFR,FGFR	13273	12986	13375	10214	13038	12115	0.27	0.25	0.26	0.23	0.29	0.26	**0.26**	**0.26**	1.00
Irinotecan HCl Trihydrat	Topoisomerase	26687	26162	25611	21938	25404	20695	0.51	0.51	0.48	0.48	0.55	0.46	**0.50**	**0.50**	1.00
Spiramycin	Others	46177	54148	50294	41246	40754	38042	0.88	1.00	0.97	0.96	0.97	0.94	**0.95**	***0.95***	1.00
Benazepril HCl	RAAS	48837	56965	58435	42070	52670	51461	1.00	1.08	1.12	0.93	1.15	1.12	**1.07**	**1.07**	1.00
Zebularine	DNA Methyltransferase	50480	50034	52462	40989	40614	45361	0.99	0.95	1.00	0.94	0.93	1.07	**0.98**	**0.98**	1.00
SRPIN340	Others	52112	52755	52090	43826	44567	42030	1.02	1.00	0.99	1.00	1.02	0.99	**1.01**	**1.01**	1.00
Calcium D-Panthotenat	Others	48878	53139	54120	41892	43169	44523	0.96	1.01	1.03	0.96	0.99	1.05	**1.00**	**1.00**	1.00
ABC294640	S1P Receptor	48950	49018	50389	38082	44040	41106	0.96	0.93	0.96	0.87	1.01	0.97	**0.95**	**0.95**	1.00
Atropine	AChR	54226	51633	54164	42092	45905	47328	1.00	0.97	1.01	0.92	0.99	1.08	**0.99**	**0.99**	1.00
Losartan Potassium (Du	RAAS	48247	50993	56805	47418	44583	46998	0.99	0.97	1.09	1.05	0.97	1.02	**1.01**	**1.01**	1.00
Brompheniramine hydr	Histamine Receptor	50368	47716	50577	45666	41677	41489	0.96	0.93	0.96	1.01	0.91	0.93	**0.95**	**0.95**	1.00
RBC8	GTPases RalA/RalB	47322	48780	47731	41659	40622	42922	0.92	0.95	0.91	0.93	0.89	0.96	**0.93**	**0.93**	1.00
(R)-Nepicastat HCl	Hydroxylase	46225	47012	47887	38523	42349	36474	0.91	0.89	0.91	0.88	0.97	0.86	**0.90**	**0.91**	1.00
Silodosin	Adrenergic Receptor	53630	58024	58481	47288	50827	46091	0.99	1.09	1.09	1.03	1.09	1.05	**1.06**	**1.06**	1.00
Memantine HCl	AMPA Receptor-kainate Receptor-NMDA R	55616	53076	53446	46242	43962	46992	1.03	1.00	0.99	1.01	0.94	1.07	**1.01**	**1.01**	1.00
Melatonin	MT Receptor	49401	54501	50295	44552	47868	45124	1.01	1.03	0.96	0.99	1.05	0.98	**1.00**	**1.00**	1.00
Sulfanilamide	Others	54633	49674	52461	48186	45629	39190	1.01	0.93	0.98	1.05	0.98	0.89	**0.97**	**0.97**	1.00
GNF-5	Bcr-Abl	49529	54746	48994	44249	45254	44079	0.97	1.07	0.93	0.99	0.99	0.98	**0.99**	**0.99**	1.00
ZM 447439	Aurora Kinase	43832	46182	43793	38172	41570	39722	0.90	0.88	0.84	0.85	0.91	0.86	**0.87**	**0.87**	1.00
Sorafenib	Raf	56109	53246	53242	50141	44594	46917	1.09	1.04	1.01	1.12	0.98	1.05	**1.05**	**1.05**	1.00
Estrone	Estrogen/progestogen Receptor	55126	55601	52171	47629	47939	42645	1.02	1.05	0.97	1.04	1.03	0.97	**1.01**	**1.01**	1.00
A-205804	Integrin	43402	44403	44500	39983	35648	30050	0.83	0.82	0.85	0.93	0.84	0.74	**0.83**	***0.84***	1.00
Pefloxacin Mesylate Dil	Topoisomerase	48985	54559	54302	44589	41036	38768	0.94	1.01	1.04	1.04	0.97	0.95	**1.00**	***1.00***	1.00
Golvatinib (E7050)	VEGFR,c-Met	44991	46369	49066	37861	36871	36251	0.86	0.85	0.94	0.88	0.87	0.89	**0.89**	***0.89***	1.00
UNC669	Epigenetic Reader Domain	51778	50171	43876	43295	43145	40779	1.01	0.98	0.83	0.97	0.95	0.91	**0.94**	**0.94**	1.00
UNC-2025	TAM Receptor,FLT3	30333	29702	31025	25710	27610	26056	0.59	0.58	0.59	0.57	0.61	0.58	**0.59**	**0.59**	1.00
DMSO		51575	56742	53977	43762	51361	49815	1.06	1.08	1.03	0.97	1.12	1.08	**1.06**	**1.06**	1.00
Adefovir Dipivoxil	Reverse Transcriptase	38744	38853	34308	31586	31816	31495	0.72	0.73	0.64	0.69	0.68	0.72	**0.69**	**0.70**	1.00
Trimebutine	Opioid Receptor	58602	54840	54406	50959	46320	45113	1.08	1.03	1.01	1.11	1.00	1.03	**1.04**	**1.04**	1.00
GSK429286A	ROCK	53568	51968	56042	47327	48532	48592	1.10	0.99	1.07	1.05	1.06	1.05	**1.05**	**1.05**	1.00
empty		54010	50877	55732	45026	45418	43342	1.06	0.97	1.06	1.03	1.04	1.02	**1.03**	**1.03**	1.00
DMSO		51187	50886	45669	43011	41966	43954	1.00	0.99	0.87	0.96	0.92	0.98	**0.95**	**0.95**	1.00
Nelfinavir Mesylate	HIV Protease	51987	46890	51393	42443	40332	42414	1.02	0.89	0.98	0.97	0.93	1.00	**0.96**	**0.97**	1.00
Doxorubicin (Adriamyci	Autophagy,Topoisomerase	7042	6293	7561	6621	5419	6659	0.14	0.12	0.14	0.15	0.12	0.14	**0.14**	**0.14**	1.00
DMSO		52607	53848	55094	46086	42557	45903	1.03	1.02	1.05	1.05	0.98	1.09	**1.04**	**1.04**	1.00
YO-01027	Gamma-secretase	52814	48430	50625	44835	44877	42298	1.00	0.95	0.96	0.99	0.98	0.95	**0.97**	**0.97**	1.00
empty		48283	49896	51795	38961	40427	40230	0.92	0.92	0.99	0.91	0.96	0.99	**0.95**	***0.95***	1.00
DTP3	JNK	46261	46037	46449	39760	39369	41976	0.90	0.90	0.88	0.89	0.87	0.94	**0.89**	**0.90**	1.00
DMSO		57380	54754	54764	48876	49133	47121	1.09	1.07	1.04	1.08	1.07	1.06	**1.07**	**1.07**	1.00
b-AP15	DUB	13258	12640	12214	11218	9977	10591	0.26	0.24	0.23	0.26	0.23	0.25	**0.24**	**0.25**	1.00
empty		53687	52386	51375	43976	43325	43971	1.05	1.00	0.98	1.00	1.00	1.04	**1.01**	**1.01**	1.00
Penciclovir	Others	49244	48392	53136	46301	39261	40169	0.97	0.92	1.01	1.06	0.90	0.95	**0.97**	**0.97**	1.00
Bexarotene	Retinoid Receptor	53316	60569	58352	48998	48493	48799	0.98	1.14	1.09	1.07	1.04	1.11	**1.07**	**1.07**	1.00
LY2886721	BACE	55307	55756	54403	48788	45211	46497	1.02	1.05	1.01	1.06	0.97	1.06	**1.03**	**1.03**	1.00
Enzalutamide (MDV310	Androgen Receptor	46481	46952	49232	42656	41048	43952	0.95	0.89	0.94	0.95	0.90	0.95	**0.93**	**0.93**	1.00
Stavudine (d4T)	Reverse Transcriptase	48338	49970	50591	45683	42760	44758	0.99	0.95	0.97	1.01	0.93	0.97	**0.97**	**0.97**	1.00
Prucalopride Succinat	5-HT Receptor	49285	48083	48449	43217	38412	39999	0.97	0.91	0.92	0.99	0.88	0.95	**0.93**	**0.94**	1.00
AG-14361	PARP	53338	56561	55030	52157	47289	44092	1.01	1.10	1.04	1.15	1.03	0.99	**1.05**	**1.06**	1.00
Bromhexine HCl	Others	57355	58234	55954	52793	48733	44358	1.06	1.09	1.04	1.15	1.05	1.01	**1.07**	**1.07**	1.00
DMSO		55700	50334	57403	46936	51544	43685	1.06	0.98	1.09	1.04	1.12	0.98	**1.04**	**1.05**	1.00
Flutamide	Androgen Receptor	57173	55549	55806	45930	46163	50864	1.06	1.04	1.04	1.00	0.99	1.16	**1.05**	**1.05**	1.00
GSK2830371	Wip1 phosphatase	49005	48720	50290	44731	43512	41032	0.96	0.95	0.95	1.00	0.96	0.91	**0.95**	**0.96**	1.00
DMSO		52623	49407	52145	44146	44236	45723	1.00	0.96	0.99	0.98	0.96	1.03	**0.98**	**0.99**	1.00
Darapladib (SB-480848	Phospholipase (e.g. PLA)	46257	49843	42995	41502	37307	42778	0.90	0.97	0.82	0.93	0.82	0.95	**0.90**	**0.90**	1.00
SD-208	TGF-beta/Smad	52639	49122	48202	42702	46152	42273	1.03	0.96	0.91	0.95	1.01	0.94	**0.97**	**0.97**	1.00
Salicin	Others	51181	50246	50694	47111	45089	40267	0.97	0.98	0.96	1.04	0.98	0.90	**0.97**	**0.98**	1.00
Saxagliptin	DPP-4	56541	52143	55349	49642	44687	45089	1.04	0.98	1.03	1.08	0.96	1.03	**1.02**	**1.02**	1.00
Paroxetine HCl	5-HT Receptor,AChR	47485	42759	44870	38085	37418	33546	0.91	0.79	0.86	0.89	0.89	0.83	**0.85**	***0.86***	1.00
Wnt-C59 (C59)	Wnt/beta-catenin	52771	54512	53377	43241	47191	43643	1.04	1.04	1.02	0.99	1.08	1.03	**1.03**	**1.03**	1.00
HO-3867	STAT	51954	48862	46911	44204	42054	42914	1.01	0.95	0.89	0.99	0.92	0.96	**0.95**	**0.96**	1.00
empty		55398	55647	51303	46945	46665	44478	1.02	1.05	0.95	1.02	1.00	1.01	**1.01**	**1.01**	1.00
Triflusal	COX	45958	51375	45098	39016	37720	36320	0.88	0.95	0.87	0.91	0.89	0.89	**0.90**	***0.90***	1.00
GW0742	PPAR	52450	53779	54847	47524	45705	47538	1.02	1.05	1.04	1.06	1.00	1.06	**1.04**	**1.04**	1.00
Indirubin	GSK-3	48797	45919	51669	48832	40142	38439	0.93	0.90	0.98	1.08	0.87	0.86	**0.93**	**0.94**	1.00
PCI-34051	HDAC	51828	52196	48110	43188	42104	44005	0.96	0.98	0.90	0.94	0.90	1.00	**0.94**	**0.95**	1.00
DMSO		46431	49059	48420	41312	40242	38597	0.91	0.93	0.92	0.94	0.92	0.91	**0.92**	**0.93**	1.01
Diclofenac Sodium	COX	51033	52587	53624	43636	45018	45058	0.94	0.99	1.00	0.95	0.97	1.03	**0.98**	**0.98**	1.01

Argatroban	Thrombin	62180	61306	55815	50530	50620	51317	1.15	1.15	1.04	1.10	1.09	1.17	**1.11**	**1.12**	1.01
VER-49009	HSP (e.g. HSP90)	35366	37527	35302	29503	33705	31490	0.69	0.73	0.67	0.66	0.74	0.70	**0.70**	**0.70**	1.01
Darunavir Ethanolate	HIV Protease	57114	55210	48807	44840	47521	44761	1.05	1.04	0.91	0.98	1.02	1.02	**1.00**	**1.01**	1.01
SBI-0206965	Autophagy	45947	47879	46086	39291	40548	42573	0.90	0.93	0.87	0.88	0.89	0.95	**0.90**	**0.91**	1.01
Necrostatin-1	TNF-alpha	52857	51834	52111	45180	46634	45385	1.03	1.01	0.99	1.01	1.03	1.01	**1.01**	**1.02**	1.01
Lomustine	Others	49854	48499	49349	43013	39734	42840	0.92	0.91	0.92	0.94	0.85	0.98	**0.92**	**0.92**	1.01
DMSO		47484	49240	53093	42846	43512	38813	0.93	0.94	1.01	0.98	1.00	0.92	**0.96**	**0.97**	1.01
empty		53784	50364	51599	44916	46031	44759	1.02	0.98	0.98	0.99	1.00	1.00	**0.99**	**1.00**	1.01
NLG919	IDO	48039	50509	46069	41049	39133	40626	0.94	0.96	0.88	0.94	0.90	0.96	**0.93**	**0.93**	1.01
Fluorometholone Aceta	Glucocorticoid Receptor	40525	46326	45667	37270	36252	37094	0.80	0.88	0.87	0.85	0.83	0.88	**0.85**	**0.85**	1.01
Azathioprine	Rho	51900	53453	49889	44778	44142	43278	0.96	1.00	0.93	0.98	0.95	0.99	**0.96**	**0.97**	1.01
Cinchonidine	Others	60370	52781	46822	47620	46856	45098	1.15	1.03	0.89	1.05	1.02	1.01	**1.02**	**1.03**	1.01
A-438079 HCl	P2 Receptor	48809	47494	47553	40679	42826	42513	0.95	0.93	0.90	0.91	0.94	0.95	**0.93**	**0.93**	1.01
BLU9931	FGFR	40198	41980	41207	36325	35333	36405	0.78	0.82	0.78	0.81	0.78	0.81	**0.79**	**0.80**	1.01
empty		50070	49587	50546	43982	43014	44552	0.98	0.97	0.96	0.98	0.95	0.99	**0.97**	**0.97**	1.01
AMI-1	Histone Methyltransferase	49974	46250	50489	44887	40823	42735	0.97	0.90	0.96	1.00	0.90	0.95	**0.94**	**0.95**	1.01
GSK2801	Epigenetic Reader Domain	47567	49937	49024	38864	39628	43925	0.93	0.95	0.93	0.89	0.91	1.04	**0.94**	**0.95**	1.01
Pioglitazone	PPAR	53763	56243	52572	49247	47094	45591	1.02	1.10	0.99	1.09	1.03	1.02	**1.04**	**1.05**	1.01
Levosulpiride	Dopamine Receptor	55092	56877	52225	46119	47551	46276	1.02	1.07	0.97	1.00	1.02	1.05	**1.02**	**1.03**	1.01
DMH1	TGF-beta/Smad	47763	50164	46939	39074	41801	40290	0.94	0.95	0.89	0.89	0.96	0.95	**0.93**	**0.94**	1.01
Methoxsalen	Others	56080	53241	53980	45949	47944	45303	1.04	1.00	1.00	1.00	1.03	1.03	**1.01**	**1.02**	1.01
Xylometazoline HCl	Adrenergic Receptor	51194	53149	53433	43513	47693	46543	0.97	1.04	1.01	0.96	1.04	1.04	**1.01**	**1.01**	1.01
Dyphylline	PDE	49377	52047	50486	46166	45048	45202	1.01	0.99	0.97	1.02	0.98	0.98	**0.99**	**1.00**	1.01
Mycophenolic acid	Dehydrogenase	27388	24862	26181	22840	22824	22796	0.52	0.49	0.50	0.50	0.50	0.51	**0.50**	**0.50**	1.01
empty		50841	46724	52208	42254	46058	42444	0.97	0.91	0.99	0.93	1.00	0.95	**0.96**	**0.96**	1.01
empty		52664	46120	48802	42482	43228	40189	0.97	0.87	0.91	0.93	0.93	0.91	**0.92**	**0.92**	1.01
Leflunomide	Dehydrogenase	44964	48633	51992	44084	43271	43293	0.92	0.92	1.00	0.98	0.95	0.94	**0.95**	**0.95**	1.01
Trelagliptin	DPP-4	49363	47017	47536	40094	41957	44171	0.96	0.92	0.90	0.90	0.92	0.98	**0.93**	**0.93**	1.01
Naftopidil	Adrenergic Receptor	56083	51643	51653	45826	47377	42819	1.04	0.97	0.96	1.00	1.02	0.97	**0.99**	**1.00**	1.01
Pritelivir (BAY 57-1293)	Others	50636	46291	42716	38499	43726	40378	0.99	0.90	0.81	0.86	0.96	0.90	**0.90**	**0.91**	1.01
Nicardipine HCl	Calcium Channel	51847	50887	52619	40783	46760	42551	1.02	0.97	1.00	0.93	1.07	1.01	**1.00**	**1.00**	1.01
Quetiapine Fumarate	Dopamine Receptor	53196	53286	50526	45141	42497	46185	0.98	1.00	0.94	0.98	0.91	1.05	**0.97**	**0.98**	1.01
Hyodeoxycholic acid (HI	Others	51058	51256	48874	42652	44628	44824	0.97	1.00	0.92	0.94	0.97	1.01	**0.97**	**0.97**	1.01
SB273005	Integrin	49856	44567	37453	40548	37469	37810	0.97	0.87	0.71	0.91	0.82	0.84	**0.85**	**0.86**	1.01
empty		51047	49896	52698	41994	47828	44989	0.99	0.97	1.00	0.94	1.05	1.00	**0.99**	**1.00**	1.01
Raltitrexed	DNA/RNA Synthesis	46576	53191	49945	45133	44733	44544	0.95	1.01	0.96	1.00	0.98	0.97	**0.97**	**0.98**	1.01
DMSO		51228	48491	51812	42780	40949	43197	1.01	0.92	0.99	0.98	0.94	1.02	**0.97**	**0.98**	1.01
Sulbactam	Others	50810	48634	52784	44792	43822	41347	0.94	0.91	0.98	0.98	0.94	0.94	**0.94**	**0.95**	1.01
Furaltadone HCl	Others	56716	54980	57052	49012	49308	43189	1.11	1.05	1.09	1.12	1.13	1.02	**1.08**	**1.09**	1.01
Estriol	Estrogen/progestogen Receptor	47101	45963	48687	40885	41645	41327	0.90	0.90	0.92	0.90	0.91	0.93	**0.90**	**0.91**	1.01
DMSO		52834	54961	53325	47356	46048	44187	0.98	1.03	0.99	1.03	0.99	1.01	**1.00**	**1.01**	1.01
LDN-214117	TGF-beta/Smad	51367	46713	48848	41217	45012	42752	1.00	0.91	0.93	0.92	0.99	0.95	**0.95**	**0.95**	1.01
Oxybuprocaine HCl	Others	51314	51378	51015	40331	45286	43155	1.01	0.98	0.97	0.92	1.04	1.02	**0.99**	**0.99**	1.01
Cetrimonium Bromide (	Others	46854	49394	47541	39293	41600	39578	0.92	0.94	0.90	0.90	0.96	0.94	**0.92**	**0.93**	1.01
empty		50247	51331	51746	46793	43004	44219	0.96	1.00	0.98	1.03	0.94	0.99	**0.98**	**0.99**	1.01
Tolfenamic Acid	COX	55785	48883	53699	45431	47986	41885	1.03	0.92	1.00	0.99	1.03	0.95	**0.98**	**0.99**	1.01
Pramiracetam	Others	53371	47848	47047	44959	43310	41356	1.01	0.93	0.89	0.99	0.94	0.93	**0.95**	**0.95**	1.01
Diphenhydramine HCl	Histamine Receptor	55976	52903	56338	49311	47381	44442	1.03	0.99	1.05	1.07	1.02	1.01	**1.03**	**1.03**	1.01
SB-334867	OX Receptor	49030	49017	50288	43323	43322	43550	0.96	0.96	0.95	0.97	0.95	0.97	**0.96**	**0.96**	1.01
TDZD-8	GSK-3	47294	48348	45165	38815	38585	36039	0.91	0.89	0.87	0.90	0.91	0.89	**0.89**	***0.90***	1.01
GNF-5837	Trk receptor	49264	45676	48845	42109	44748	39397	0.96	0.89	0.93	0.94	0.98	0.88	**0.93**	**0.93**	1.01
LRRK2-IN-1	LRRK2	47980	53646	50064	45083	41886	46200	0.94	1.05	0.95	1.01	0.92	1.03	**0.98**	**0.99**	1.01
Capecitabine	DNA/RNA Synthesis	50368	56554	55481	48067	48218	49668	1.03	1.07	1.06	1.07	1.05	1.08	**1.06**	**1.07**	1.01
DMSO		49481	52812	53199	48033	45595	46163	1.01	1.00	1.02	1.06	1.00	1.00	**1.01**	**1.02**	1.01
Pefloxacin Mesylate	Others	49122	53842	50796	43996	44804	42612	0.91	1.01	0.95	0.96	0.96	0.97	**0.95**	**0.96**	1.01
Vanillin	Others	46760	49467	43984	38942	37080	35604	0.90	0.91	0.84	0.91	0.88	0.88	**0.88**	***0.89***	1.01
Budesonide	Glucocorticoid Receptor	47426	54426	46793	41631	45364	46716	0.97	1.03	0.90	0.92	0.99	1.01	**0.97**	**0.98**	1.01
AVL-292	BTK	35633	36050	38855	33775	31153	27833	0.70	0.69	0.74	0.77	0.72	0.66	**0.71**	**0.72**	1.01
Tebipenem Pivoxil	Others	52498	51442	50722	43260	42102	49850	1.00	1.00	0.96	0.96	0.92	1.12	**0.99**	**1.00**	1.01
FH535	Wnt/beta-catenin,PPAR	51272	44388	44531	39850	40252	43100	1.00	0.87	0.84	0.89	0.88	0.96	**0.90**	**0.91**	1.01
Nateglinide	Potassium Channel	55724	49356	48342	45751	42815	45606	1.06	0.96	0.91	1.01	0.93	1.02	**0.98**	**0.99**	1.01
Brivanib Alaninate (BM	VEGFR,FGFR	44706	49888	50273	43338	41602	45310	0.92	0.95	0.96	0.96	0.91	0.98	**0.94**	**0.95**	1.01
Ursolic Acid	Others	52147	48998	48336	45339	43772	41715	0.99	0.96	0.91	1.00	0.95	0.94	**0.95**	**0.96**	1.01
empty		44732	53442	50848	41340	41059	42450	0.88	1.02	0.97	0.94	0.94	1.00	**0.95**	**0.96**	1.01
Imatinib Mesylate (STI5	c-Kit,Bcr-Abl,PDGFR	50414	55449	55571	49636	51032	44465	1.03	1.05	1.06	1.10	1.12	0.96	**1.05**	**1.06**	1.01
empty		51797	49364	55936	42575	45434	43766	1.02	0.94	1.06	0.97	1.04	1.03	**1.01**	**1.02**	1.01
K-Ras(G12C) inhibitor 9	Rho	48811	53804	50867	44388	43838	46661	0.95	1.05	0.96	0.99	0.96	1.04	**0.99**	**1.00**	1.01
Finasteride	5-alpha Reductase	45038	46386	49287	40645	42098	43957	0.92	0.88	0.94	0.90	0.92	0.95	**0.92**	**0.92**	1.01

Aminoglutethimide	Aromatase	54288	54312	55272	47898	46955	45282	1.00	1.02	1.03	1.04	1.01	1.03	**1.02**	**1.03**	1.01
4µ8C	Others	51529	50624	54808	42932	41684	46996	1.01	0.96	1.04	0.98	0.96	1.11	**1.01**	**1.02**	1.01
GSK2656157	PERK	45273	51594	49744	41138	40950	40874	0.89	0.98	0.95	0.94	0.94	0.97	**0.94**	**0.95**	1.01
Lincomycin HCl	Others	52331	51044	50080	44422	46631	43363	1.00	1.00	0.95	0.98	1.02	0.97	**0.98**	**0.99**	1.01
Alibendol	Others	57586	53952	53171	47678	49550	43748	1.06	1.01	0.99	1.04	1.06	1.00	**1.02**	**1.03**	1.01
Sulindac	COX	49202	55028	52405	49391	41157	43425	0.91	1.03	0.98	1.08	0.88	0.99	**0.97**	**0.98**	1.01
Hesperadin	Aurora Kinase	12132	13460	15899	11821	11117	12505	0.22	0.25	0.30	0.26	0.24	0.28	**0.26**	**0.26**	1.01
L-(+)-Rhamnose Monoh	Others	54906	52964	51490	48767	42995	47728	1.04	1.03	0.97	1.08	0.94	1.07	**1.02**	**1.03**	1.01
ML324	Histone Demethylase	54440	53875	52827	43490	45620	46155	1.07	1.03	1.01	0.99	1.05	1.09	**1.03**	**1.04**	1.01
empty		50119	48964	56223	45917	43362	47156	0.98	0.96	1.07	1.03	0.95	1.05	**1.00**	**1.01**	1.01
Fluorouracil (5-Fluoraci	DNA/RNA Synthesis	41956	47134	46052	40697	42521	38398	0.86	0.89	0.88	0.90	0.93	0.83	**0.88**	**0.89**	1.01
Sumatriptan Succinate	5-HT Receptor	49289	53159	52820	45872	47127	46858	1.01	1.01	1.01	1.02	1.03	1.02	**1.01**	**1.02**	1.01
Racecadotril	Opioid Receptor	52213	49240	47966	49249	43319	38403	0.99	0.96	0.91	1.09	0.94	0.86	**0.95**	**0.96**	1.01
BIX 02188	MEK	50980	54075	54947	45684	44304	46834	0.94	1.02	1.02	1.00	0.95	1.07	**0.99**	**1.00**	1.01
Guanosine	Others	51071	46810	48197	42076	44171	41738	0.97	0.91	0.91	0.93	0.96	0.94	**0.93**	**0.94**	1.01
Verapamil HCl	Calcium Channel	52086	50244	55764	45110	44541	43157	1.02	0.96	1.06	1.03	1.02	1.02	**1.01**	**1.02**	1.01
Sulfathiazole	Others	49140	47117	48465	37752	39923	39277	0.94	0.87	0.93	0.88	0.95	0.97	**0.91**	***0.92***	1.01
Dutasteride	5-alpha Reductase	46020	48580	48713	43467	43333	42327	0.94	0.92	0.93	0.96	0.95	0.92	**0.93**	**0.94**	1.01
Defactinib (VS-6063, PF	FAK	46527	45819	44887	38944	40398	41435	0.91	0.89	0.85	0.87	0.89	0.92	**0.88**	**0.89**	1.01
Foretinib (GSK1363089	VEGFR,c-Met	34140	33533	35510	30925	28526	33645	0.70	0.64	0.68	0.69	0.62	0.73	**0.67**	**0.68**	1.01
Acetylcysteine	Others	59653	58010	57001	50412	49353	49701	1.10	1.09	1.06	1.10	1.06	1.13	**1.08**	**1.10**	1.01
Moxifloxacin HCl	Topoisomerase	53316	56242	55165	50362	50838	47272	1.09	1.07	1.06	1.12	1.11	1.02	**1.07**	**1.08**	1.01
CP-724714	EGFR,HER2	45739	53326	48966	48340	44625	40245	0.94	1.01	0.94	1.07	0.98	0.87	**0.96**	**0.97**	1.01
RepSox	TGF-beta/Smad	51299	50636	55861	45703	45130	41812	1.01	0.96	1.06	1.04	1.04	0.99	**1.01**	**1.02**	1.01
BML-190	Cannabinoid Receptor	50929	47379	48944	41198	35401	37375	0.98	0.87	0.94	0.96	0.84	0.92	**0.93**	***0.94***	1.01
Clemastine Fumarate	Histamine Receptor	54706	54190	52138	49181	40956	47560	1.01	1.02	0.97	1.07	0.88	1.08	**1.00**	**1.01**	1.01
Tolvaptan	Vasopressin Receptor	52402	48207	53635	50235	42419	42486	1.00	0.94	1.01	1.11	0.92	0.95	**0.98**	**1.00**	1.01
DMSO		53300	52368	52443	45422	49283	44522	1.04	1.02	0.99	1.02	1.08	0.99	**1.02**	**1.03**	1.01
Bosentan Hydrate	Endothelin Receptor	50215	51544	55123	43153	136	40509	0.96	0.95	1.06	1.01	0.00	1.00	**0.99**	***1.00***	1.01
empty		49273	53013	54028	46266	44547	40592	0.97	1.01	1.03	1.06	1.02	0.96	**1.00**	**1.01**	1.01
GSK2606414	PERK	45794	51675	48791	41414	42457	39039	0.90	0.98	0.93	0.95	0.98	0.92	**0.94**	**0.95**	1.01
Procaine HCl	Sodium Channel	47518	52721	49156	40234	40635	39322	0.91	0.97	0.94	0.94	0.96	0.97	**0.94**	***0.95***	1.01
Tyrphostin 9	EGFR	20148	16548	19169	15963	14899	13904	0.39	0.31	0.37	0.37	0.35	0.34	**0.35**	***0.36***	1.01
Alogliptin	DPP-4	53704	53262	50791	43433	39153	40693	1.03	0.98	0.98	1.01	0.93	1.00	**1.00**	***1.01***	1.01
PND-1186 (VS-4718)	FAK	38646	39435	38165	32871	37070	32492	0.75	0.77	0.72	0.74	0.81	0.72	**0.75**	**0.76**	1.01
empty		52478	53402	53390	47585	43687	48389	1.00	1.04	1.01	1.05	0.95	1.08	**1.02**	**1.03**	1.01
SU5402	FGFR,VEGFR	47205	47376	50549	43238	41509	43037	0.92	0.92	0.96	0.97	0.91	0.96	**0.93**	**0.95**	1.01
DMSO		49225	50583	51065	41769	46048	45130	0.96	0.99	0.97	0.93	1.01	1.01	**0.97**	**0.98**	1.01
empty		45297	47887	47387	39972	38741	39498	0.89	0.91	0.90	0.91	0.89	0.93	**0.90**	**0.91**	1.01
Timolol Maleate	Adrenergic Receptor	50001	50463	53059	44992	40821	43313	0.98	0.96	1.01	1.03	0.94	1.02	**0.98**	**1.00**	1.01
CAL-101 (Idelalisib, GS-	PI3K	48877	47000	48836	50423	37069	39462	0.93	0.92	0.92	1.11	0.81	0.88	**0.92**	**0.94**	1.01
WS3	IκB/IKK	17671	16328	15403	14446	14915	14208	0.34	0.32	0.29	0.32	0.33	0.32	**0.32**	**0.32**	1.01
Levetiracetam	Calcium Channel	46126	48781	48293	41459	42824	45022	0.94	0.92	0.93	0.92	0.94	0.98	**0.93**	**0.94**	1.01
Fidaxomicin	DNA/RNA Synthesis	44982	44961	51831	39696	40741	38827	0.88	0.86	0.99	0.91	0.94	0.92	**0.91**	**0.92**	1.01
GZD824	Bcr-Abl	14994	15860	15173	12930	12941	12852	0.29	0.30	0.29	0.30	0.30	0.30	**0.29**	**0.30**	1.01
Formononetin	Others	49695	54341	49675	47094	44507	43445	0.94	1.06	0.94	1.04	0.97	0.97	**0.98**	**1.00**	1.01
Remodelin	Histone Acetyltransferase	53815	50584	46646	45261	42829	45126	1.05	0.99	0.88	1.01	0.94	1.01	**0.97**	**0.99**	1.01
ADL5859 HCl	Opioid Receptor	45835	48145	54702	41302	45030	47888	0.94	0.91	1.05	0.92	0.98	1.04	**0.97**	**0.98**	1.01
PF-04418948	Others	50793	48442	49621	43795	38760	42753	1.00	0.92	0.94	1.00	0.89	1.01	**0.95**	**0.97**	1.01
empty		49327	52065	45248	37952	44594	40156	0.94	0.96	0.87	0.88	1.06	0.99	**0.92**	***0.94***	1.01
Rimantadine	Others	51773	51031	56203	45781	43704	46758	0.96	0.96	1.05	1.00	0.94	1.06	**0.99**	**1.00**	1.01
JNJ-7777120	Histamine Receptor	45998	47897	46493	38601	39697	36343	0.88	0.88	0.89	0.90	0.94	0.90	**0.89**	***0.90***	1.01
PP2	Src	51511	48052	46373	43085	43064	36912	1.01	0.91	0.88	0.98	0.99	0.87	**0.94**	**0.95**	1.01
Phenoxybenzamine HCl	Adrenergic Receptor	50285	48226	48429	44178	43954	40947	0.96	0.94	0.92	0.98	0.96	0.92	**0.94**	**0.95**	1.01
DMSO		45653	51857	49896	41239	40562	37470	0.87	0.96	0.96	0.96	0.96	0.92	**0.93**	***0.94***	1.01
Diacerein	Others	49442	48263	52461	42320	41615	42487	0.97	0.92	1.00	0.97	0.96	1.00	**0.96**	**0.98**	1.01
Bay 11-7085	IκB/IKK	30041	32357	33325	27543	26635	30211	0.59	0.63	0.63	0.62	0.59	0.67	**0.62**	**0.62**	1.01
empty		46065	51529	53235	44409	45157	46582	0.94	0.98	1.02	0.98	0.99	1.01	**0.98**	**0.99**	1.01
Sinomenine	Others	50024	45213	52620	43927	45761	40186	0.95	0.88	1.00	0.97	1.00	0.90	**0.94**	**0.96**	1.01
AZD1981	GPR	55595	56928	53914	47854	45833	46504	1.09	1.08	1.03	1.09	1.05	1.10	**1.07**	**1.08**	1.01
Amantadine HCl	Dopamine Receptor	58534	48081	50243	46856	44848	46011	1.11	0.94	0.95	1.04	0.98	1.03	**1.00**	**1.01**	1.01
Etodolac	COX	46889	48417	52516	45252	43131	45158	0.96	0.92	1.01	1.00	0.94	0.98	**0.96**	**0.97**	1.01
CW069	Microtubule Associated	56694	53015	51865	43445	49842	49355	1.10	1.03	0.98	0.97	1.10	1.10	**1.04**	**1.06**	1.01
Sertraline HCl	5-HT Receptor	51643	51599	50975	41426	43182	40919	0.99	0.95	0.98	0.97	1.02	1.01	**0.97**	***0.99***	1.01
Thiamphenicol	Others	51140	50485	49781	44546	43335	45144	0.97	0.99	0.94	0.98	0.94	1.01	**0.97**	**0.98**	1.01
CP-673451	PDGFR	60647	57262	57863	51788	47498	51441	1.12	1.08	1.08	1.13	1.02	1.17	**1.09**	**1.11**	1.01
DMSO		48254	54499	50634	42284	40907	39632	0.92	1.00	0.97	0.99	0.97	0.98	**0.97**	***0.98***	1.01
Glipizide	Others	58741	59535	55254	49305	48554	51005	1.08	1.12	1.03	1.07	1.04	1.16	**1.08**	**1.09**	1.01

empty		53599	52468	52596	45224	46511	48307	1.04	1.02	1.00	1.01	1.02	1.08	**1.02**	**1.04**	1.01
Troxerutin	Others	49252	48217	49856	45818	41829	41867	0.94	0.94	0.94	1.01	0.91	0.94	**0.94**	**0.95**	1.01
Digoxin	Sodium Channel	6890	7086	6768	5998	5765	5726	0.14	0.13	0.13	0.14	0.13	0.14	**0.13**	**0.13**	1.01
empty		51872	47432	50059	41371	43672	40944	1.02	0.90	0.95	0.94	1.00	0.97	**0.96**	**0.97**	1.01
Phenacetin	COX	56666	52248	54233	49609	47194	46631	1.08	1.02	1.03	1.10	1.03	1.05	**1.04**	**1.06**	1.01
GKT137831	Others	50066	50216	48249	41385	43438	40443	0.98	0.96	0.92	0.94	1.00	0.96	**0.95**	**0.97**	1.01
AT7867	S6 Kinase,Akt	48741	51510	48530	45115	41434	41273	0.90	0.97	0.90	0.98	0.89	0.94	**0.92**	**0.94**	1.01
LY3009120	Raf	31715	34300	32459	28611	28444	29906	0.62	0.67	0.62	0.64	0.63	0.67	**0.63**	**0.64**	1.01
NSC 405020	MMP	53037	49941	53448	45838	48431	43866	1.03	0.97	1.01	1.03	1.06	0.98	**1.01**	**1.02**	1.02
KN-62	CaMK	47988	49450	46645	41790	40024	45445	0.94	0.96	0.88	0.93	0.88	1.01	**0.93**	**0.94**	1.02
Formoterol Hemifumar	Adrenergic Receptor	53807	51797	48505	44097	46083	42284	0.99	0.97	0.90	0.96	0.99	0.96	**0.96**	**0.97**	1.02
G-749	FLT3	43001	38956	37739	35687	36911	33207	0.84	0.76	0.72	0.80	0.81	0.74	**0.77**	**0.78**	1.02
PAC-1	Caspase	51807	50379	50681	40162	43111	41531	0.99	0.93	0.97	0.94	1.02	1.02	**0.96**	***0.98***	1.02
GNE-9605	LRRK2	51881	48553	45728	43820	42221	43156	1.01	0.95	0.87	0.98	0.93	0.96	**0.94**	**0.96**	1.02
Clopidogrel	P2 Receptor	48129	51487	51604	45518	42977	48344	0.99	0.98	0.99	1.01	0.94	1.05	**0.98**	**1.00**	1.02
DMSO		54222	54944	51110	45697	40733	40118	1.04	1.01	0.98	1.07	0.96	0.99	**1.01**	***1.03***	1.02
DMSO		54375	56753	55279	46444	51081	48985	1.03	1.11	1.05	1.03	1.11	1.10	**1.06**	**1.08**	1.02
empty		45157	48846	48964	39416	45254	41688	0.88	0.95	0.93	0.88	0.99	0.93	**0.92**	**0.94**	1.02
BAF312 (Siponimod)	S1P Receptor	50064	49874	52526	44309	41914	42421	0.98	0.95	1.00	1.01	0.96	1.00	**0.98**	**0.99**	1.02
K02288	TGF-beta/Smad	47103	50671	50405	45256	42781	42883	0.92	0.99	0.96	1.01	0.94	0.96	**0.95**	**0.97**	1.02
Epalrestat	Others	57073	49077	51527	48494	44184	42871	1.05	0.92	0.96	1.06	0.95	0.98	**0.98**	**0.99**	1.02
A-803467	Sodium Channel	51209	52230	54247	45687	40213	38855	0.98	0.96	1.04	1.07	0.95	0.96	**1.00**	***1.01***	1.02
GSK650394	Others	44910	47034	46909	36586	41489	39063	0.88	0.89	0.89	0.84	0.95	0.92	**0.89**	**0.90**	1.02
DMSO		47804	52977	56433	49584	45358	47243	0.98	1.00	1.08	1.10	0.99	1.02	**1.02**	**1.04**	1.02
Beta-Lapachone	Topoisomerase	42710	41945	38478	36382	34830	32846	0.84	0.80	0.73	0.83	0.80	0.78	**0.79**	**0.80**	1.02
Propylthiouracil	Others	48424	51690	48703	43439	42809	41792	0.89	0.97	0.91	0.95	0.92	0.95	**0.92**	**0.94**	1.02
PHT-427	Akt,PDK-1	54490	55481	49044	44032	46315	46387	1.01	1.04	0.91	0.96	1.00	1.06	**0.99**	**1.00**	1.02
Ipratropium Bromide	AChR	56865	52902	56471	46482	48131	48303	1.05	0.99	1.05	1.01	1.03	1.10	**1.03**	**1.05**	1.02
Novobiocin Sodium	Topoisomerase	51649	50318	50827	44411	44060	46110	0.98	0.98	0.96	0.98	0.96	1.03	**0.98**	**0.99**	1.02
AZD7545	Others	48234	52017	46142	42159	42412	45002	0.94	1.02	0.87	0.94	0.93	1.00	**0.94**	**0.96**	1.02
empty		55249	52732	54805	47138	48003	48879	1.08	1.03	1.04	1.05	1.06	1.09	**1.05**	**1.07**	1.02
empty		54038	52373	46898	45777	41192	44871	1.00	0.98	0.87	1.00	0.89	1.02	**0.95**	**0.97**	1.02
ML167	CDK	43879	49917	48649	41708	42119	42208	0.86	0.97	0.92	0.93	0.93	0.94	**0.92**	**0.93**	1.02
Vatalanib (PTK787) 2HC	c-Kit,VEGFR	45122	52284	51876	43735	43053	48376	0.92	0.99	0.99	0.97	0.94	1.05	**0.97**	**0.99**	1.02
Ifenprodil Tartrate	GluR	49857	55805	50285	41599	40361	41829	0.96	1.03	0.97	0.97	0.96	1.03	**0.98**	***1.00***	1.02
Lornoxicam	COX	59733	58666	58749	50994	50801	50658	1.10	1.10	1.09	1.11	1.09	1.15	**1.10**	**1.12**	1.02
OSI-930	CSF-1R,VEGFR,c-Kit	44773	50826	50229	45429	42323	44280	0.92	0.96	0.96	1.01	0.93	0.96	**0.95**	**0.96**	1.02
Nutlin-3b	Mdm2	53268	51366	48350	46386	44693	44394	1.04	1.00	0.92	1.04	0.98	0.99	**0.99**	**1.00**	1.02
Isoxazole 9 (ISX-9)	Others	50358	50324	53194	46163	45272	44733	0.98	0.98	1.01	1.03	1.00	1.00	**0.99**	**1.01**	1.02
DMXAA (Vadimezan)	VDA	58199	52424	53022	47982	46949	45946	1.07	0.99	0.99	1.05	1.01	1.05	**1.02**	**1.03**	1.02
Azelnidipine	Calcium Channel	51273	54805	49116	44546	166	38686	0.98	1.01	0.94	1.04	0.00	0.95	**0.98**	***1.00***	1.02
Obeticholic Acid	FXR	47114	49022	50590	42280	41453	46103	0.92	0.96	0.96	0.95	0.91	1.03	**0.94**	**0.96**	1.02
EUK 134	Beta Amyloid	46713	47765	50137	40127	41044	41014	0.92	0.91	0.95	0.92	0.94	0.97	**0.93**	**0.94**	1.02
Fluconazole	P450 (e.g. CYP17)	51921	53570	51935	46912	47824	48124	1.06	1.02	1.00	1.04	1.05	1.04	**1.02**	**1.04**	1.02
Naltrexone HCl	Opioid Receptor	55253	52613	58748	49339	49291	44960	1.02	0.99	1.09	1.08	1.06	1.02	**1.03**	**1.05**	1.02
KC7F2	HIF	51010	47959	50264	44308	45524	42322	0.99	0.94	0.95	0.99	1.00	0.94	**0.96**	**0.98**	1.02
Olaparib (AZD2281, Ku-	PARP	53395	51551	50838	47228	47678	46597	1.09	0.98	0.97	1.05	1.04	1.01	**1.01**	**1.03**	1.02
Phenazopyridine HCl	Others	51279	51002	54989	44607	47950	40491	1.01	0.97	1.05	1.02	1.10	0.96	**1.01**	**1.03**	1.02
DMSO		53401	53181	57390	48619	49487	47089	1.04	1.04	1.09	1.09	1.09	1.05	**1.06**	**1.07**	1.02
Embelin	IAP	48717	47821	46881	39126	39589	42532	0.96	0.91	0.89	0.89	0.91	1.01	**0.92**	**0.94**	1.02
SB505124	TGF-beta/Smad	51162	54354	50740	45798	40182	51773	0.97	1.06	0.96	1.01	0.88	1.16	**1.00**	**1.02**	1.02
Semagacestat (LY4501:	Gamma-secretase	56906	53909	53004	51711	46079	43468	1.05	1.01	0.99	1.13	0.99	0.99	**1.02**	**1.04**	1.02
BIX 02189	MEK	51740	53447	51940	44943	44825	45608	0.96	1.00	0.97	0.98	0.96	1.04	**0.98**	**0.99**	1.02
Atglistatin	Others	51381	49475	44808	42950	42246	43965	1.00	0.97	0.85	0.96	0.93	0.98	**0.94**	**0.96**	1.02
Chlorquinaldol	Others	46562	52978	47621	43087	41270	40129	0.91	1.01	0.91	0.98	0.95	0.95	**0.94**	**0.96**	1.02
1-Azakenpaullone	GSK-3	52415	52176	51701	46560	44992	40801	1.03	0.99	0.98	1.06	1.03	0.96	**1.00**	**1.02**	1.02
Enoxacin	Topoisomerase	52591	48926	51752	45439	43640	43028	0.97	0.92	0.96	0.99	0.94	0.98	**0.95**	**0.97**	1.02
Salmeterol Xinafoate	Adrenergic Receptor	44231	52022	48200	41184	41212	39763	0.87	0.99	0.92	0.94	0.95	0.94	**0.93**	**0.94**	1.02
Benzocaine	Sodium Channel	50399	51666	54324	43217	46854	42272	0.99	0.98	1.03	0.99	1.08	1.00	**1.00**	**1.02**	1.02
Spironolactone	Androgen Receptor	47950	47369	52268	41605	40092	37710	0.92	0.87	1.00	0.97	0.95	0.93	**0.93**	***0.95***	1.02
Sitagliptin phosphate m	DPP-4	45012	48226	45485	39010	39273	35467	0.86	0.89	0.87	0.91	0.93	0.87	**0.87**	***0.89***	1.02
VGX-1027	Others	55381	48143	48741	47292	44782	42954	1.08	0.94	0.92	1.06	0.98	0.96	**0.98**	**1.00**	1.02
Tropisetron	5-HT Receptor	57625	49944	52520	44824	46439	46689	1.06	0.94	0.98	0.98	1.00	1.06	**0.99**	**1.01**	1.02
Tianeptine sodium	5-HT Receptor	47910	50718	50123	44798	43738	46612	0.98	0.96	0.96	0.99	0.96	1.01	**0.97**	**0.99**	1.02
JNJ-38877605	c-Met	49075	50462	53250	45390	46295	47149	1.00	0.96	1.02	1.01	1.01	1.02	**0.99**	**1.01**	1.02
MetoclopraMide HCl	Dopamine Receptor	47901	46314	52494	43036	41671	39591	0.94	0.88	1.00	0.98	0.96	0.94	**0.94**	**0.96**	1.02
Vitamin D2	Others	52460	53042	51665	41084	43398	43260	1.01	0.98	0.99	0.96	1.03	1.07	**0.99**	***1.01***	1.02
Chloroxine	Others	53458	52988	49529	43416	44830	46323	0.99	1.00	0.92	0.95	0.96	1.05	**0.97**	**0.99**	1.02

empty		52000	48451	49946	43754	44944	44814	1.01	0.95	0.95	0.98	0.99	1.00	0.97	0.99	1.02
DMSO		53997	54567	52863	45768	45195	45838	1.06	1.04	1.01	1.05	1.04	1.08	1.03	1.06	1.02
Tubastatin A	HDAC	47961	50799	49442	45016	41391	45111	0.93	0.99	0.94	1.01	0.91	1.01	0.95	0.97	1.02
AGI-5198	Dehydrogenase	50965	55773	55504	43350	50415	43718	1.00	1.06	1.06	0.99	1.16	1.03	1.04	1.06	1.02
Pramipexole	Dopamine Receptor	52559	49009	51183	43759	46756	44553	1.00	0.96	0.97	0.97	1.02	1.00	0.97	1.00	1.02
NSC 319726	p53	16724	17088	15498	15390	12561	13871	0.33	0.33	0.29	0.35	0.29	0.33	0.32	0.32	1.02
EPZ-6438	Histone Methyltransferase	48753	46030	45785	41428	39381	38460	0.96	0.88	0.87	0.95	0.90	0.91	0.90	0.92	1.02
DMSO		54316	54786	54416	47547	49001	44790	1.00	1.03	1.01	1.04	1.05	1.02	1.02	1.04	1.02
ETP-46464	ATM/ATR,mTOR	51275	44701	48527	43557	43177	41593	1.00	0.87	0.92	0.97	0.95	0.93	0.93	0.95	1.02
RGFP966	HDAC	49696	49222	50225	44060	42310	40182	0.98	0.94	0.96	1.01	0.97	0.95	0.96	0.98	1.02
Streptozotocin (STZ)	Others	44364	51454	53632	44160	47300	44295	0.91	0.98	1.03	0.98	1.03	0.96	0.97	0.99	1.02
AZD2932	VEGFR,c-Kit,PDGFR,FLT3	46646	45417	50406	41241	39510	40072	0.92	0.86	0.96	0.94	0.91	0.95	0.91	0.93	1.02
DMSO		50486	45538	50695	44065	43174	43052	0.98	0.89	0.96	0.99	0.95	0.96	0.94	0.96	1.02
Dapoxetine HCl	5-HT Receptor	50890	51466	51090	43933	47482	41353	0.94	0.97	0.95	0.96	1.02	0.94	0.95	0.97	1.02
Ferrostatin-1 (Fer-1)	Ferroptosis	49066	50575	53322	44704	41633	43390	0.96	0.96	1.01	1.02	0.96	1.03	0.98	1.00	1.02
Phenytoin	Sodium Channel	49942	47861	46714	44887	40457	42533	0.95	0.93	0.88	0.99	0.88	0.95	0.92	0.94	1.02
Trospium chloride	AChR	46386	45083	46734	43286	39361	39646	0.88	0.88	0.88	0.96	0.86	0.89	0.88	0.90	1.02
TAK-632	Raf	51890	45435	45757	41264	41089	39226	1.02	0.86	0.87	0.94	0.94	0.93	0.92	0.94	1.02
Ki16425	LPA Receptor	49446	53408	52369	45377	49777	46166	1.01	1.01	1.00	1.01	1.09	1.00	1.01	1.03	1.02
Teniposide	Topoisomerase	9068	11175	10238	8022	9013	9324	0.17	0.21	0.19	0.17	0.19	0.21	0.19	0.19	1.02
Pazopanib	PDGFR,VEGFR,c-Kit	44557	44584	44253	38685	36802	33275	0.85	0.82	0.85	0.90	0.87	0.82	0.84	*0.86*	1.02
DMSO		52559	54346	51233	45953	48778	46045	1.02	1.06	0.97	1.03	1.07	1.03	1.02	1.04	1.02
Deltarasin	PDE	51949	51927	50913	44359	45408	41841	1.02	0.99	0.97	1.01	1.04	0.99	0.99	1.02	1.02
SGC 0946	Histone Methyltransferase	47326	49148	49522	43519	41545	39053	0.93	0.94	0.94	0.99	0.95	0.92	0.94	0.96	1.02
2-Methoxyestradiol (2-I	HIF	24425	26324	26748	23817	24394	22403	0.50	0.50	0.51	0.53	0.53	0.49	0.50	0.52	1.02
NH125	CaMK	22067	20228	24302	18273	21348	19650	0.43	0.39	0.46	0.41	0.47	0.44	0.43	0.44	1.02
empty		50686	51042	49485	44248	44488	45929	0.99	1.00	0.94	0.99	0.98	1.02	0.97	1.00	1.02
UM171	Others	44303	45196	43197	36211	40193	41808	0.86	0.88	0.82	0.81	0.88	0.93	0.85	0.87	1.02
EHT 1864	Rho	46366	47647	44097	41247	41714	40082	0.90	0.93	0.84	0.92	0.92	0.89	0.89	0.91	1.02
Clofarabine	DNA/RNA Synthesis	11727	11788	11894	11049	10179	11089	0.24	0.22	0.23	0.24	0.22	0.24	0.23	0.24	1.02
U0126-EtOH	MEK	49099	45669	49058	44800	43561	43006	1.01	0.87	0.94	0.99	0.95	0.93	0.94	0.96	1.02
Betamipron	Others	48330	51127	51341	43846	42206	42132	0.95	0.97	0.98	1.00	0.97	1.00	0.97	0.99	1.02
Carbidopa	Others	50424	50513	53706	49165	40322	44445	0.93	0.95	1.00	1.07	0.87	1.01	0.96	0.98	1.02
4E1RCat	Others	49035	52185	45010	41214	45844	43390	0.96	1.02	0.85	0.92	1.01	0.97	0.94	0.97	1.02
Amoxicillin Sodium	Others	54543	50486	52292	47787	46695	45131	1.04	0.99	0.99	1.06	1.02	1.01	1.00	1.03	1.02
Sulfamethoxazole	Others	52063	49499	49203	44579	43297	42796	0.96	0.93	0.92	0.97	0.93	0.97	0.94	0.96	1.02
Clotrimazole	Others	54222	53814	51116	50588	41199	46078	1.00	1.01	0.95	1.10	0.89	1.05	0.99	1.01	1.02
Verdinexor (KPT-335)	CRM1	20184	23979	18894	18809	17606	19833	0.39	0.47	0.36	0.42	0.39	0.44	0.41	0.42	1.02
DMSO		52954	54268	52780	46733	48343	43771	0.98	1.02	0.98	1.02	1.04	1.00	0.99	1.02	1.02
BMS-833923	Hedgehog/Smoothened	44268	49433	45406	40830	38871	38666	0.87	0.94	0.86	0.93	0.89	0.91	0.89	0.91	1.02
Rifaximin	DNA/RNA Synthesis	49356	48842	45463	41618	40736	42150	0.91	0.92	0.85	0.91	0.88	0.96	0.89	0.91	1.02
Chlorothiazide	Others	52006	50469	52429	43850	48631	42013	0.96	0.95	0.98	0.96	1.05	0.96	0.96	0.99	1.03
Pralatrexate	DHFR	45428	48967	51612	43227	47038	43024	0.93	0.93	0.99	0.96	1.03	0.93	0.95	0.97	1.03
KRN 633	VEGFR,PDGFR	53739	54729	50953	49178	40302	48606	0.99	1.03	0.95	1.07	0.87	1.11	0.99	1.01	1.03
Icariin	PDE	50250	45540	49069	43967	41573	43060	0.96	0.89	0.93	0.97	0.91	0.97	0.92	0.95	1.03
PF-543	S1P Receptor	46402	51171	50602	43536	41330	41289	0.91	0.97	0.96	0.99	0.95	0.98	0.95	0.97	1.03
Lamivudine	Reverse Transcriptase	52577	50707	49448	44779	45004	42781	0.97	0.95	0.92	0.98	0.97	0.97	0.95	0.97	1.03
GDC-0879	Raf	47870	59608	54629	46026	52025	49875	0.98	1.13	1.05	1.02	1.14	1.08	1.05	1.08	1.03
Meglumine	Others	51645	51761	55730	46040	45580	46466	0.95	0.97	1.04	1.00	0.98	1.06	0.99	1.01	1.03
DMSO		55081	53375	57278	46052	51588	50293	1.07	1.04	1.09	1.03	1.13	1.12	1.07	1.09	1.03
SMI-4a	Pim	51476	50333	53637	45222	46515	47002	1.00	0.98	1.02	1.01	1.02	1.05	1.00	1.03	1.03
R406	FLT3,Syk	52135	47301	48604	45402	44453	41806	0.99	0.92	0.92	1.00	0.97	0.94	0.94	0.97	1.03
Eprosartan Mesylate	RAAS	50055	50062	50385	42132	45253	39283	0.96	0.92	0.97	0.98	1.07	0.97	0.95	*0.97*	1.03
PX-478 2HCl	HIF	48918	48160	47922	42626	42202	44708	0.95	0.94	0.91	0.95	0.93	1.00	0.93	0.96	1.03
empty		50231	50657	48006	42055	45331	45147	0.96	0.99	0.91	0.93	0.99	1.01	0.95	0.98	1.03
CX-6258 HCl	Pim	29788	30327	29105	25331	25311	25473	0.59	0.58	0.55	0.58	0.58	0.60	0.57	0.59	1.03
NSC 23766	Rho	50742	52792	51820	46985	45929	45932	0.99	1.03	0.98	1.05	1.01	1.02	1.00	1.03	1.03
DMSO		51278	50340	50445	45245	43636	46461	0.98	0.98	0.95	1.00	0.95	1.04	0.97	1.00	1.03
Lovastatin	HMG-CoA Reductase	52021	53192	49341	45166	47283	42100	0.96	1.00	0.92	0.98	1.02	0.96	0.96	0.99	1.03
DMSO		49076	53347	54280	49455	47510	46441	1.00	1.01	1.04	1.10	1.04	1.01	1.02	1.05	1.03
Bromosporine	Epigenetic Reader Domain	49203	56666	53224	45422	43025	47221	0.97	1.08	1.01	1.04	0.99	1.12	1.02	1.05	1.03
Phenytoin sodium	Sodium Channel	52366	47897	47365	45034	45837	40680	1.00	0.94	0.90	1.00	1.00	0.91	0.94	0.97	1.03
URMC-099	MLK,LRRK,Abl,VEGFR/FLT	47673	49237	53829	46103	45336	43298	0.93	0.96	1.02	1.03	1.00	0.96	0.97	1.00	1.03
Carvedilol	Adrenergic Receptor	53670	51874	51355	44173	46803	45515	0.99	0.98	0.96	0.96	1.01	1.04	0.97	1.00	1.03
Ramipril	RAAS	52582	49757	46291	46541	41311	41484	0.97	0.94	0.86	1.01	0.89	0.94	0.92	0.95	1.03
SB202190 (FHPI)	p38 MAPK	49018	52255	50753	43407	48415	47544	1.00	0.99	0.97	0.96	1.06	1.03	0.99	1.02	1.03
Tolbutamide	Potassium Channel	47814	45237	47941	40910	43758	40980	0.91	0.88	0.91	0.90	0.95	0.92	0.90	0.93	1.03
Menadione	Others	53207	52023	55185	46105	47050	46441	0.98	0.98	1.03	1.00	1.01	1.06	1.00	1.02	1.03

Rilpivirine	Reverse Transcriptase	46621	53891	52481	42993	44590	43019	0.92	1.03	1.00	0.98	1.02	1.02	**0.98**	**1.01**	1.03
PTC-209	Others	23061	25695	21452	21320	19121	22415	0.45	0.50	0.41	0.48	0.42	0.50	**0.45**	**0.47**	1.03
Ivermectin	Others	48439	51575	48647	44664	45245	46441	0.99	0.98	0.93	0.99	0.99	1.01	**0.97**	**1.00**	1.03
Luteolin	PDE	50110	55331	47820	46417	44669	45664	0.95	1.08	0.90	1.03	0.97	1.02	**0.98**	**1.01**	1.03
Albendazole Oxide	Others	49189	49464	50160	42909	40147	46319	0.91	0.93	0.93	0.93	0.86	1.05	**0.92**	**0.95**	1.03
GW9662	PPAR	41651	48432	49951	39004	39716	36885	0.80	0.89	0.96	0.91	0.94	0.91	**0.88**	***0.91***	1.03
Candesartan	RAAS	57260	58402	56947	52628	49383	48340	1.06	1.10	1.06	1.15	1.06	1.10	**1.07**	**1.10**	1.03
Otilonium Bromide	AChR	45001	48493	49323	41378	37104	36146	0.86	0.89	0.95	0.96	0.88	0.89	**0.90**	***0.93***	1.03
ZLN005	Others	44302	42750	46899	39534	39899	40490	0.86	0.83	0.89	0.88	0.88	0.90	**0.86**	**0.89**	1.03
Fludarabine	STAT,DNA/RNA Synthesis	47853	52912	56940	50028	50279	44141	0.98	1.00	1.09	1.11	1.10	0.96	**1.02**	**1.05**	1.03
EX 527 (Selisistat)	Sirtuin	54540	55757	51861	49216	47183	44965	1.01	1.05	0.97	1.07	1.01	1.02	**1.01**	**1.04**	1.03
empty		46000	50895	49680	46238	43204	44946	0.94	0.97	0.95	1.02	0.94	0.97	**0.95**	**0.98**	1.03
DMSO		56232	52891	53603	48795	49518	46883	1.07	1.03	1.01	1.08	1.08	1.05	**1.04**	**1.07**	1.03
Ketorolac	COX	53756	54722	55323	46395	50085	46348	0.99	1.03	1.03	1.01	1.08	1.06	**1.02**	**1.05**	1.03
Potassium Iodide	Others	58804	53080	56156	48127	50288	48025	1.09	1.00	1.05	1.05	1.08	1.09	**1.04**	**1.07**	1.03
D 4476	Casein Kinase	46716	47964	44811	40556	42721	41787	0.91	0.94	0.85	0.91	0.94	0.93	**0.90**	**0.93**	1.03
Dronedarone HCl	Potassium Channel,Sodium Channel,Calciur	52230	51464	55334	49239	47996	41600	0.96	0.97	1.03	1.07	1.03	0.95	**0.99**	**1.02**	1.03
Oxiracetam	Others	52367	45758	52384	44340	41444	43085	1.03	0.87	1.00	1.01	0.95	1.02	**0.97**	**0.99**	1.03
Inosine	Others	49093	49391	47888	44936	41489	44244	0.93	0.96	0.91	0.99	0.90	0.99	**0.93**	**0.96**	1.03
FPH1 (BRD-6125)	Others	52818	49392	52821	45452	50718	42857	1.03	0.96	1.00	1.02	1.11	0.95	**1.00**	**1.03**	1.03
Avobenzone	Others	50445	49374	47470	42656	44893	40973	0.93	0.93	0.88	0.93	0.96	0.93	**0.91**	**0.94**	1.03
AEBSF HCl	Serine Protease	50576	53687	46893	42838	44850	47983	0.99	1.05	0.89	0.96	0.99	1.07	**0.97**	**1.00**	1.03
DMSO		53094	52418	56973	45540	52985	47221	1.03	1.02	1.08	1.02	1.16	1.05	**1.05**	**1.08**	1.03
Regorafenib (BAY 73-4!	c-RET,VEGFR	45048	49382	50399	41537	45870	45668	0.92	0.94	0.97	0.92	1.00	0.99	**0.94**	**0.97**	1.03
CP-91149	Phosphorylase	44401	52308	50359	38642	38992	41113	0.85	0.96	0.97	0.90	0.92	1.01	**0.93**	***0.96***	1.03
VER155008	HSP (e.g. HSP90)	43843	43357	45282	38367	41480	39124	0.85	0.85	0.86	0.86	0.91	0.87	**0.85**	**0.88**	1.03
Levosimendan	Others	46526	46256	47979	42037	42982	40903	0.88	0.90	0.91	0.93	0.94	0.92	**0.90**	**0.93**	1.03
Desvenlafaxine Succina	5-HT Receptor	48386	50432	45805	41540	44720	37143	0.93	0.93	0.88	0.97	1.06	0.91	**0.91**	***0.94***	1.03
AMG-517	TRPV	50072	49320	50649	47855	40717	40233	0.98	0.94	0.96	1.09	0.94	0.95	**0.96**	**0.99**	1.03
Medetomidine HCl	Adrenergic Receptor	46053	46961	53127	40969	40045	38598	0.88	0.87	1.02	0.96	0.95	0.95	**0.92**	***0.95***	1.03
FLI-06	Gamma-secretase	50497	45709	50293	46574	40604	44373	0.98	0.89	0.95	1.04	0.89	0.99	**0.94**	**0.97**	1.03
AT7519	CDK	21217	25592	21012	19654	19805	19839	0.39	0.48	0.39	0.43	0.43	0.45	**0.42**	**0.44**	1.03
PF-2545920	PDE	52884	49243	47947	45877	41220	47126	1.01	0.96	0.91	1.01	0.90	1.06	**0.96**	**0.99**	1.03
Pexidartinib (PLX3397)	CSF-1R,c-Kit	46911	50447	50278	41714	47200	43793	0.91	0.98	0.95	0.93	1.04	0.98	**0.95**	**0.98**	1.03
PF-477736	Chk	26295	28151	28725	23065	22998	22198	0.50	0.52	0.55	0.54	0.54	0.55	**0.52**	***0.54***	1.03
Terbinafine	Others	56010	57625	51402	49658	50272	44574	1.03	1.08	0.96	1.08	1.08	1.01	**1.02**	**1.06**	1.03
EHop-016	Rho	49404	48618	50588	42015	42171	43363	0.97	0.93	0.96	0.96	0.97	1.03	**0.95**	**0.98**	1.03
Prilocaine	Others	52475	53089	49163	45191	44180	45902	0.97	1.00	0.92	0.98	0.95	1.04	**0.96**	**0.99**	1.03
Clevidipine Butyrate	Calcium Channel	50647	52228	55527	45143	44336	48922	0.94	0.98	1.03	0.98	0.95	1.11	**0.98**	**1.02**	1.03
BMS-265246	CDK	45724	45460	46068	40339	41445	38355	0.84	0.85	0.86	0.88	0.89	0.87	**0.85**	**0.88**	1.03
FLLL32	JAK	47850	43448	37030	31338	44556	34473	0.94	0.83	0.70	0.72	1.02	0.82	**0.82**	**0.85**	1.03
Domperidone	Dopamine Receptor	47315	48686	45714	41400	43004	42623	0.90	0.95	0.86	0.92	0.94	0.96	**0.91**	**0.94**	1.03
Brimonidine Tartrate	Adrenergic Receptor	46415	52171	50780	43535	43104	41614	0.91	0.99	0.97	0.99	0.99	0.98	**0.96**	**0.99**	1.03
Bumetanide	Others	49653	52044	52345	45533	48603	47866	1.02	0.99	1.00	1.01	1.06	1.04	**1.00**	**1.04**	1.03
Balofloxacin	Others	52968	53679	56242	47743	48752	46149	0.98	1.01	1.05	1.04	1.05	1.05	**1.01**	**1.05**	1.03
KPT-276	CRM1	27138	33982	31225	28716	25051	25537	0.53	0.65	0.59	0.66	0.58	0.60	**0.59**	**0.61**	1.03
Azithromycin	Autophagy	52315	53006	53050	47960	47173	43638	0.97	1.00	0.99	1.04	1.01	0.99	**0.98**	**1.02**	1.03
KU-60019	ATM/ATR	51439	53213	48382	45139	45023	43945	0.95	1.00	0.90	0.98	0.97	1.00	**0.95**	**0.98**	1.04
UMI-77	Bcl-2	45592	51563	45039	41395	42202	44574	0.89	1.01	0.85	0.93	0.93	0.99	**0.92**	**0.95**	1.04
Epacadostat (INCB0243	IDO	48491	48781	49477	43984	42347	45861	0.95	0.95	0.94	0.98	0.93	1.02	**0.95**	**0.98**	1.04
PCI-24781 (Abexinostat	HDAC	44402	49615	48554	43274	41725	46510	0.91	0.94	0.93	0.96	0.91	1.01	**0.93**	**0.96**	1.04
BRD4770	Histone Methyltransferase	48524	46549	48770	42584	41594	45397	0.95	0.91	0.92	0.95	0.91	1.01	**0.93**	**0.96**	1.04
empty		49841	48740	48995	42424	45258	45374	0.97	0.95	0.93	0.95	0.99	1.01	**0.95**	**0.98**	1.04
empty		51629	53171	52392	44392	42544	41428	0.99	0.98	1.01	1.03	1.01	1.02	**0.99**	***1.03***	1.04
Hydroxyurea	DNA/RNA Synthesis	53424	52476	55593	42111	47866	51421	0.99	0.99	1.03	0.92	1.03	1.17	**1.00**	**1.04**	1.04
XEN445	Others	49080	52004	48262	45521	44227	44942	0.96	1.01	0.92	1.02	0.97	1.00	**0.96**	**1.00**	1.04
PFI-2	Histone Methyltransferase	53784	50459	54058	47016	46588	42828	1.06	0.96	1.03	1.07	1.07	1.01	**1.01**	**1.05**	1.04
Ulipristal	Estrogen/progestogen Receptor	48678	49946	49001	43616	37507	37128	0.93	0.92	0.94	1.02	0.89	0.91	**0.93**	***0.97***	1.04
Valsartan	RAAS	51803	49604	48989	40894	46273	44785	0.96	0.93	0.91	0.89	0.99	1.02	**0.93**	**0.97**	1.04
JIB-04	Histone Demethylase	16123	17548	16046	14713	14578	13554	0.32	0.33	0.31	0.34	0.33	0.32	**0.32**	**0.33**	1.04
CZC24832	PI3K	46440	48935	48822	38102	44095	41980	0.91	0.93	0.93	0.87	1.01	0.99	**0.92**	**0.96**	1.04
Ursodiol	Others	52041	50281	51753	46033	41851	47218	0.96	0.95	0.96	1.00	0.90	1.07	**0.96**	**0.99**	1.04
DMSO		55513	52675	57700	47113	49313	49176	1.02	0.99	1.07	1.03	1.06	1.12	**1.03**	**1.07**	1.04
BRL-15572	5-HT Receptor	52206	48504	49753	45126	44860	45277	0.99	0.95	0.94	1.00	0.98	1.02	**0.96**	**1.00**	1.04
VER-50589	HSP (e.g. HSP90)	13127	17021	14669	13365	13827	13291	0.26	0.33	0.28	0.30	0.30	0.30	**0.29**	**0.30**	1.04
Nepafenac	COX	43359	49531	48174	39911	45083	45405	0.89	0.94	0.92	0.88	0.99	0.98	**0.92**	**0.95**	1.04
AT9283	Aurora Kinase,JAK,Bcr-Abl	25642	26656	29460	24483	23654	27483	0.52	0.51	0.56	0.54	0.52	0.60	**0.53**	**0.55**	1.04
PD-1/PD-L1 inhibitor 2	PD-1/PD-L1	50782	49495	49961	43594	46755	45407	0.99	0.97	0.95	0.97	1.03	1.01	**0.97**	**1.00**	1.04

Letrozole	Aromatase	43704	50879	52385	46704	46176	42891	0.89	0.96	1.00	1.04	1.01	0.93	**0.95**	**0.99**	1.04
SC79	Akt	47306	51175	49775	43060	44153	46783	0.92	1.00	0.94	0.96	0.97	1.04	**0.95**	**0.99**	1.04
4EGI-1	Others	47099	48559	47054	44543	40322	44104	0.92	0.95	0.89	1.00	0.89	0.98	**0.92**	**0.96**	1.04
Rebamipide	Others	56286	51836	51858	47501	44638	48425	1.04	0.97	0.97	1.03	0.96	1.10	**0.99**	**1.03**	1.04
ESI-09	Others	52421	49094	51177	45460	47492	45169	1.02	0.96	0.97	1.02	1.04	1.01	**0.98**	**1.02**	1.04
empty		49761	54632	52946	46152	46273	43429	0.98	1.04	1.01	1.05	1.06	1.03	**1.01**	**1.05**	1.04
Rifabutin	Others	48794	51477	49771	46211	43722	42143	0.90	0.97	0.93	1.01	0.94	0.96	**0.93**	**0.97**	1.04
Vidarabine	DNA/RNA Synthesis	52835	51754	48447	48323	46225	40281	0.98	0.97	0.90	1.05	0.99	0.92	**0.95**	**0.99**	1.04
NU1025	PARP	48486	46540	49405	44517	42874	43207	0.94	0.91	0.94	1.00	0.94	0.96	**0.93**	**0.97**	1.04
Pregnenolone	Estrogen/progestogen Receptor	51134	53184	51079	43645	46189	46847	0.94	1.00	0.95	0.95	0.99	1.07	**0.96**	**1.00**	1.04
Rosuvastatin Calcium	HMG-CoA Reductase	49008	47323	52186	45902	40020	47743	0.93	0.92	0.99	1.01	0.87	1.07	**0.95**	**0.99**	1.04
Protionamide	Others	54521	48668	48520	42617	44671	46105	1.01	0.91	0.90	0.93	0.96	1.05	**0.94**	**0.98**	1.04
Brivanib (BMS-540215)	VEGFR,FGFR	47913	51728	55193	47288	46315	49838	0.98	0.98	1.06	1.05	1.01	1.08	**1.01**	**1.05**	1.04
NVP-BVU972	c-Met	51394	51899	50156	44835	39218	39344	0.98	0.96	0.96	1.05	0.93	0.97	**0.97**	***1.01***	1.04
Diosmetin	P450 (e.g. CYP17)	53315	48494	49751	47419	46066	43159	1.01	0.95	0.94	1.05	1.00	0.97	**0.97**	**1.01**	1.04
Cilnidipine	Calcium Channel	47446	48240	53294	45487	46044	46609	0.97	0.91	1.02	1.01	1.01	1.01	**0.97**	**1.01**	1.04
Cryptotanshinone	STAT	44526	42822	44591	42800	40832	35402	0.85	0.84	0.84	0.95	0.89	0.79	**0.84**	**0.88**	1.04
FG-4592	HIF	44985	43389	46338	40229	42736	42120	0.92	0.82	0.89	0.89	0.93	0.91	**0.88**	**0.91**	1.04
DMSO		44893	50412	49103	43552	47204	43080	0.92	0.96	0.94	0.97	1.03	0.93	**0.94**	**0.98**	1.04
Laquinimod	Others	47446	52769	55438	45781	42138	39671	0.91	0.97	1.06	1.07	1.00	0.98	**0.98**	***1.02***	1.04
WS6	IκB/IKK	55522	54722	51419	49263	48573	48638	1.08	1.07	0.97	1.10	1.07	1.08	**1.04**	**1.08**	1.04
Forskolin	cAMP	50884	48124	48476	44273	47043	41776	0.97	0.94	0.92	0.98	1.03	0.94	**0.94**	**0.98**	1.04
Nicaraven	Others	50613	46917	48267	41780	45581	38876	0.99	0.89	0.92	0.95	1.05	0.92	**0.94**	**0.97**	1.04
Arbutin	Others	53263	46734	49663	45677	45171	44134	1.01	0.91	0.94	1.01	0.98	0.99	**0.96**	**0.99**	1.04
Almorexant HCl	OX Receptor	52284	54807	53779	46793	47719	50647	0.99	1.07	1.02	1.03	1.04	1.14	**1.03**	**1.07**	1.04
Mexiletine HCl	Sodium Channel	48565	49869	49014	42109	41321	44072	0.95	0.95	0.93	0.96	0.95	1.04	**0.95**	**0.98**	1.04
Danusertib (PHA-73935	FGFR,Aurora Kinase,c-RET,Bcr-Abl	26533	27986	27198	23980	24933	26983	0.54	0.53	0.52	0.53	0.55	0.58	**0.53**	**0.55**	1.04
ZCL278	Rho	49079	57811	50382	44349	47746	43923	0.96	1.10	0.96	1.01	1.10	1.04	**1.01**	**1.05**	1.04
Azaperone	Others	49898	47378	46359	37787	40754	45660	0.98	0.90	0.88	0.86	0.94	1.08	**0.92**	**0.96**	1.04
OAC1	Others	44212	49241	48121	41941	40788	39791	0.87	0.94	0.92	0.96	0.94	0.94	**0.91**	**0.95**	1.04
GNE-7915	LRRK2	48483	47566	45941	42506	44313	42022	0.94	0.93	0.87	0.95	0.97	0.94	**0.91**	**0.95**	1.04
Diosmin	Others	53670	45632	45843	42572	43210	45286	1.02	0.89	0.87	0.94	0.94	1.02	**0.93**	**0.97**	1.04
Flucytosine	Others	50343	46814	49609	42587	42811	44042	0.93	0.88	0.92	0.93	0.92	1.00	**0.91**	**0.95**	1.04
Florfenicol	Others	45320	47086	53569	46313	40975	39202	0.89	0.90	1.02	1.06	0.94	0.93	**0.94**	**0.98**	1.04
Nimesulide	COX	52925	52995	54522	49204	46375	46069	0.98	1.00	1.01	1.07	1.00	1.05	**1.00**	**1.04**	1.04
Acipimox	Others	50151	47625	49890	43066	44169	43110	0.93	0.90	0.93	0.94	0.95	0.98	**0.92**	**0.96**	1.04
CK-636	Microtubule Associated	44865	50055	47496	45400	42538	41330	0.87	0.98	0.90	1.02	0.94	0.92	**0.92**	**0.96**	1.04
DMSO		48119	51890	55545	48060	46352	46703	0.94	1.01	1.05	1.07	1.02	1.04	**1.00**	**1.04**	1.04
Temocapril HCl	RAAS	53091	55229	52682	48741	47502	45978	0.98	1.04	0.98	1.06	1.02	1.05	**1.00**	**1.04**	1.04
INH1	Microtubule Associated	49142	48492	49954	42385	43734	47849	0.96	0.95	0.95	0.95	0.96	1.07	**0.95**	**0.99**	1.04
Dabrafenib (GSK21184:	Raf	44213	44457	45576	38966	35834	34909	0.85	0.82	0.87	0.91	0.85	0.86	**0.85**	***0.88***	1.04
VX-745	p38 MAPK	45885	50016	48319	43108	42973	48060	0.94	0.95	0.93	0.96	0.94	1.04	**0.94**	**0.98**	1.04
Divalproex Sodium	Autophagy	51833	49145	52782	46057	45775	44002	0.96	0.92	0.98	1.00	0.98	1.00	**0.95**	**1.00**	1.04
UNC2881	TAM Receptor	41118	43931	41808	34769	39366	35836	0.81	0.84	0.80	0.79	0.90	0.85	**0.81**	**0.85**	1.04
Rufinamide	Sodium Channel	42240	44525	46303	42053	43745	37931	0.86	0.84	0.89	0.93	0.96	0.82	**0.87**	**0.90**	1.04
(S)-crizotinib	Others	46781	46527	46249	39135	46194	41507	0.91	0.91	0.88	0.88	1.02	0.92	**0.90**	**0.94**	1.04
Nimodipine	Autophagy,Calcium Channel	53713	46621	51618	46249	42953	44929	0.99	0.88	0.96	1.01	0.92	1.02	**0.94**	**0.98**	1.04
CPI-203	Epigenetic Reader Domain	35204	38620	37111	33779	30355	32033	0.69	0.73	0.71	0.77	0.70	0.76	**0.71**	**0.74**	1.04
Cediranib (AZD2171)	VEGFR	41019	47314	46346	46744	38616	39697	0.84	0.90	0.89	1.04	0.84	0.86	**0.87**	**0.91**	1.04
empty		52612	48538	55349	46759	48586	46771	1.03	0.95	1.05	1.05	1.07	1.04	**1.01**	**1.05**	1.04
OSU-03012 (AR-12)	PDK-1	45328	52898	50468	46988	45140	46049	0.93	1.00	0.97	1.04	0.99	1.00	**0.97**	**1.01**	1.04
DMSO		52007	47467	48925	43734	44551	42846	0.96	0.89	0.91	0.95	0.96	0.98	**0.92**	**0.96**	1.04
DCC-2036 (Rebastinib)	Bcr-Abl	50733	50123	48284	44804	46359	43852	0.96	0.98	0.91	0.99	1.01	0.98	**0.95**	**0.99**	1.04
Zalcitabine	Reverse Transcriptase	54978	50039	50118	46214	49300	41773	1.02	0.94	0.93	1.01	1.06	0.95	**0.96**	**1.01**	1.04
Cytisine	AChR	49086	44321	51507	44465	42667	43910	0.93	0.87	0.97	0.98	0.93	0.98	**0.92**	**0.97**	1.04
Zonisamide	Sodium Channel	46940	51469	52352	45240	49426	45612	0.96	0.98	1.00	1.00	1.08	0.99	**0.98**	**1.02**	1.04
PIK-93	PI3K	42273	46852	48894	42010	42982	43417	0.87	0.89	0.94	0.93	0.94	0.94	**0.90**	**0.94**	1.05
BTZ043 Racemate	Others	43212	50293	51343	45721	44190	44771	0.88	0.95	0.98	1.01	0.97	0.97	**0.94**	**0.98**	1.05
Roxatidine Acetate HCl	Histamine Receptor	49222	49884	48711	43698	42880	44152	0.91	0.94	0.91	0.95	0.92	1.01	**0.92**	**0.96**	1.05
Hesperetin	TGF-beta/Smad,Histamine Receptor	50577	56105	51605	47279	48370	47866	0.96	1.10	0.98	1.05	1.05	1.07	**1.01**	**1.06**	1.05
Proparacaine HCl	Sodium Channel	51731	49544	49446	43427	47578	42458	0.96	0.93	0.92	0.95	1.02	0.97	**0.94**	**0.98**	1.05
Chlorpheniramine Male	Histamine Receptor	50265	50792	49973	45310	46378	42075	0.93	0.95	0.93	0.99	1.00	0.96	**0.94**	**0.98**	1.05
Orphenadrine Citrate	AChR	55447	52651	48407	43953	47224	47249	1.02	0.99	0.90	0.96	1.01	1.08	**0.97**	**1.02**	1.05
Xanthohumol	COX	46389	47720	49575	40015	45168	45580	0.90	0.93	0.94	0.89	0.99	1.02	**0.93**	**0.97**	1.05
Amfenac Sodium Monc	COX	48155	50512	48307	44923	43090	39770	0.95	0.96	0.92	1.03	0.99	0.94	**0.94**	**0.99**	1.05
Pramoxine HCl	Others	48487	49276	50686	41289	39254	40502	0.93	0.91	0.97	0.96	0.93	1.00	**0.94**	***0.98***	1.05
empty		56096	55302	55738	49094	50056	48829	1.04	1.04	1.04	1.07	1.08	1.11	**1.04**	**1.09**	1.05
TAK-700 (Orteronel)	P450 (e.g. CYP17)	49641	53126	55108	49065	50300	47792	1.02	1.01	1.06	1.09	1.10	1.04	**1.03**	**1.07**	1.05

PF-431396	FAK	41794	40742	40034	36220	36947	38466	0.81	0.80	0.76	0.81	0.81	0.86	0.79	0.83	1.05
empty		49205	47853	52084	44052	41962	38331	0.94	0.88	1.00	1.03	0.99	0.94	0.94	0.99	1.05
Voglibose	Others	50476	52973	52134	45051	44931	40807	0.97	0.98	1.00	1.05	1.06	1.00	0.98	1.03	1.05
DBeQ	p97	51665	51289	53591	48151	44615	43464	1.01	0.98	1.02	1.10	1.03	1.03	1.00	1.05	1.05
Naringin Dihydrochalco	Others	46562	44775	46948	41375	40661	43403	0.89	0.87	0.89	0.91	0.89	0.97	0.88	0.92	1.05
PF-04620110	Transferase	49532	51638	50764	43027	46171	43035	0.97	0.98	0.97	0.98	1.06	1.02	0.97	1.02	1.05
Palbociclib (PD-033299:	CDK	36896	38921	40582	34549	36367	37792	0.76	0.74	0.78	0.77	0.80	0.82	0.76	0.79	1.05
Temsirolimus (CCI-779,	mTOR	29709	26357	27448	24782	25630	27811	0.61	0.50	0.53	0.55	0.56	0.60	0.54	0.57	1.05
Bilobalide	Others	44749	44480	46465	44076	39182	39952	0.85	0.87	0.88	0.97	0.85	0.90	0.87	0.91	1.05
Linifanib (ABT-869)	VEGFR,PDGFR,CSF-1R	45293	46392	49003	43864	43025	44542	0.93	0.88	0.94	0.97	0.94	0.97	0.92	0.96	1.05
empty		52728	54460	50706	45470	49774	44927	0.97	1.02	0.94	0.99	1.07	1.02	0.98	1.03	1.05
Rifapentine	Others	49818	48552	50113	44747	43726	43253	0.92	0.91	0.93	0.97	0.94	0.98	0.92	0.97	1.05
Cyclocytidine HCl	DNA/RNA Synthesis	30203	29203	34923	26176	28794	28700	0.56	0.55	0.65	0.57	0.62	0.65	0.59	0.61	1.05
I-BET-762	Epigenetic Reader Domain	41987	46776	48964	40435	40005	39472	0.82	0.89	0.93	0.92	0.92	0.93	0.88	0.93	1.05
Albendazole	Microtubule Associated	27124	31188	24969	25463	24840	23718	0.50	0.59	0.46	0.55	0.53	0.54	0.52	0.54	1.05
empty		49118	52061	45818	46219	43352	38713	0.96	0.99	0.87	1.06	1.00	0.92	0.94	0.99	1.05
Metronidazole	DNA/RNA Synthesis	55350	53725	49977	46338	50276	44762	1.02	1.01	0.93	1.01	1.08	1.02	0.99	1.04	1.05
Oxytetracycline (Terran	Others	49894	49335	50718	43117	44032	45939	0.92	0.93	0.94	0.94	0.95	1.05	0.93	0.98	1.05
UNC1999	Histone Methyltransferase	47823	48222	49008	41399	40375	44610	0.94	0.92	0.93	0.95	0.93	1.05	0.93	0.98	1.05
empty		50883	50655	46781	44457	43917	41090	1.00	0.96	0.89	1.02	1.01	0.97	0.95	1.00	1.05
Lansoprazole	Proton Pump	46528	49499	45782	39668	46375	46793	0.95	0.94	0.88	0.88	1.01	1.01	0.92	0.97	1.05
Milciclib (PHA-848125)	CDK	31344	33757	36017	29235	27688	26763	0.60	0.62	0.69	0.68	0.66	0.66	0.64	0.67	1.05
FR 180204	ERK	50214	54275	47736	46471	44448	48295	0.98	1.06	0.91	1.04	0.98	1.08	0.98	1.03	1.05
DMSO		46615	48493	46553	38860	40143	39499	0.89	0.89	0.89	0.91	0.95	0.97	0.89	0.94	1.05
MI-3 (Menin-MLL Inhibi	Histone Methyltransferase	44100	44876	43209	41261	37252	42397	0.86	0.88	0.82	0.92	0.82	0.94	0.85	0.90	1.05
Vecuronium Bromide	Others	52518	52691	53749	48636	49773	50685	1.08	1.00	1.03	1.08	1.09	1.10	1.03	1.09	1.05
Mianserin HCl	Histamine Receptor	48284	51205	52766	46467	47747	48469	0.99	0.97	1.01	1.03	1.04	1.05	0.99	1.04	1.05
EW-7197	TGF-beta/Smad	43253	50493	45986	43093	39864	44897	0.84	0.99	0.87	0.96	0.88	1.00	0.90	0.95	1.05
Gatifloxacin	Topoisomerase	47001	50516	50142	47384	45210	45753	0.96	0.96	0.96	1.05	0.99	0.99	0.96	1.01	1.05
DMSO		50687	50714	51231	47111	46052	42794	0.94	0.95	0.95	1.03	0.99	0.97	0.95	1.00	1.05
Erastin	Ferroptosis	48118	45765	47211	39251	41366	42637	0.94	0.87	0.90	0.90	0.95	1.01	0.90	0.95	1.05
Levonorgestrel	Estrogen/progestogen Receptor	53928	54799	55486	49697	48926	47586	1.00	1.03	1.03	1.08	1.05	1.08	1.02	1.07	1.05
TTNPB (Arotinoid Acid)	Retinoid Receptor	46259	46568	41780	38057	37655	41871	0.91	0.89	0.79	0.87	0.87	0.99	0.86	0.91	1.05
Mercaptopurine (6-MP)	DNA/RNA Synthesis	44900	46267	51528	41338	46709	45742	0.92	0.88	0.99	0.92	1.02	0.99	0.93	0.98	1.05
Cinacalcet HCl	CaSR	46944	46782	50289	47028	46769	41259	0.96	0.89	0.96	1.04	1.02	0.89	0.94	0.99	1.05
Hyoscyamine	AChR	49281	50404	47777	43180	39600	38630	0.94	0.93	0.92	1.01	0.94	0.95	0.93	0.98	1.05
R406 (free base)	Syk	51850	50055	45680	44363	43405	43647	0.96	0.94	0.85	0.97	0.93	0.99	0.92	0.96	1.05
Alverine Citrate	Others	47130	47484	49885	40739	152	39390	0.90	0.88	0.96	0.95	0.00	0.97	0.91	0.96	1.05
LY2119620	AChR	53063	54467	47750	46852	47571	47933	1.03	1.06	0.91	1.05	1.05	1.07	1.00	1.05	1.05
Ciprofibrate	PPAR	48229	44020	53948	45468	44174	43624	0.92	0.86	1.02	1.01	0.96	0.98	0.93	0.98	1.05
Hydrochlorothiazide	Others	50534	47742	51411	45248	44018	44158	0.93	0.90	0.96	0.99	0.95	1.01	0.93	0.98	1.05
CPI-613	Dehydrogenase	50915	49930	49548	42677	42507	40826	0.98	0.92	0.95	0.99	1.01	1.01	0.95	1.00	1.05
8-Bromo-cAMP	PKA	50296	49789	48745	46500	44291	45683	0.98	0.97	0.92	1.04	0.97	1.02	0.96	1.01	1.05
Napabucasin	STAT	533	579	511	505	494	490	0.01	0.01	0.01	0.01	0.01	0.01	0.01	0.01	1.05
CH5183284 (Debio-134:	FGFR	42117	44074	44261	40090	41576	38030	0.82	0.86	0.84	0.90	0.91	0.85	0.84	0.89	1.05
Bicalutamide	Androgen Receptor	44632	46926	48361	43202	43342	44960	0.91	0.89	0.93	0.96	0.95	0.97	0.91	0.96	1.05
CTEP (RO4956371)	GluR	44657	48117	50777	41749	40202	38062	0.86	0.89	0.97	0.97	0.95	0.94	0.91	0.96	1.05
DMSO		52698	50220	50344	42878	41874	42271	1.01	0.93	0.97	1.00	0.99	1.04	0.97	1.02	1.05
DMSO		49025	45826	48648	44796	42900	43430	0.93	0.89	0.92	0.99	0.93	0.97	0.92	0.97	1.06
Roflumilast	PDE	49462	50586	48394	44491	43438	44560	0.91	0.95	0.90	0.97	0.93	1.01	0.92	0.97	1.06
Equol	Estrogen/progestogen Receptor	49637	47379	49288	43403	44626	45701	0.94	0.93	0.93	0.96	0.97	1.02	0.93	0.99	1.06
Rucaparib (AG-014699,	PARP	41489	49534	48725	41397	46500	43331	0.85	0.94	0.93	0.92	1.02	0.94	0.91	0.96	1.06
DAPT (GSI-IX)	Gamma-secretase,Beta Amyloid	52679	48616	49513	47210	44562	46122	1.00	0.95	0.94	1.04	0.97	1.03	0.96	1.02	1.06
DMSO		49343	52214	50432	45974	46900	50298	1.01	0.99	0.97	1.02	1.03	1.09	0.99	1.04	1.06
KPT-330	CRM1	27061	26015	26079	23165	22572	23737	0.53	0.49	0.50	0.53	0.52	0.56	0.51	0.54	1.06
SB225002	CXCR	30650	28307	27827	26041	24737	29014	0.60	0.55	0.53	0.58	0.54	0.65	0.56	0.59	1.06
empty		52710	54779	48815	47383	45872	46507	0.97	1.03	0.91	1.03	0.99	1.06	0.97	1.03	1.06
TAME	APC,E3 Ligase	52375	50876	51760	48342	43873	49678	1.00	0.99	0.98	1.07	0.96	1.11	0.99	1.05	1.06
Triapine	DNA/RNA Synthesis	48518	44885	43963	42729	44668	39028	0.95	0.88	0.83	0.96	0.98	0.87	0.89	0.94	1.06
Vismodegib (GDC-0449	Hedgehog/Smoothened	47095	50571	55277	46929	49610	47443	0.96	0.96	1.06	1.04	1.08	1.03	0.99	1.05	1.06
Pranlukast	Others	51651	50635	46601	44906	44503	43797	0.95	0.95	0.87	0.98	0.96	1.00	0.92	0.98	1.06
SN-38	Topoisomerase	6241	6313	5837	5546	5029	5576	0.12	0.12	0.11	0.13	0.12	0.13	0.12	0.12	1.06
DMSO		49359	52009	53456	47450	45735	42829	0.97	0.99	1.02	1.08	1.05	1.01	0.99	1.05	1.06
Masitinib (AB1010)	PDGFR,c-Kit	52842	53444	55114	47735	51338	53171	1.08	1.01	1.06	1.06	1.12	1.15	1.05	1.11	1.06
Dacomitinib (PF299804	EGFR	42934	45113	48232	39634	37521	36366	0.82	0.83	0.93	0.92	0.89	0.90	0.86	0.91	1.06
KPT-185	CRM1	17993	22419	21231	19730	17404	17004	0.35	0.43	0.40	0.45	0.40	0.40	0.39	0.42	1.06
Gossypol	Dehydrogenase	42778	44730	46576	40066	38745	44074	0.81	0.87	0.88	0.89	0.84	0.99	0.86	0.91	1.06
SB203580	p38 MAPK	48247	50580	54243	47461	49457	47444	0.99	0.96	1.04	1.05	1.08	1.03	1.00	1.05	1.06

Valnemulin HCl	Others	50380	49585	49577	43578	44662	43302	0.99	0.94	0.94	1.00	1.03	1.02	0.96	1.02	1.06
Phenindione	Others	54650	54025	48720	47089	47645	46298	1.01	1.02	0.91	1.03	1.02	1.05	0.98	1.03	1.06
Tetracycline HCl	Others	47972	47592	45960	44171	44562	41186	0.91	0.93	0.87	0.98	0.97	0.92	0.90	0.96	1.06
SNS-032 (BMS-387032)	CDK	13600	13303	13807	12231	12619	13618	0.28	0.25	0.26	0.27	0.28	0.30	0.27	0.28	1.06
BAPTA-AM	Others	48069	45323	41552	41298	40632	42477	0.94	0.88	0.79	0.92	0.89	0.95	0.87	0.92	1.06
Apixaban	Factor Xa	53874	50496	55802	48798	49672	45169	0.99	0.95	1.04	1.06	1.07	1.03	0.99	1.05	1.06
NPS-1034	TAM Receptor,c-Met	41500	40439	41557	36181	39920	37723	0.81	0.79	0.79	0.81	0.88	0.84	0.80	0.84	1.06
DMSO		46678	50982	53173	47006	45062	46879	0.91	0.99	1.01	1.05	0.99	1.04	0.97	1.03	1.06
Ipriflavone (Osteofix)	Others	48434	46236	49557	46091	44065	42239	0.92	0.90	0.94	1.02	0.96	0.95	0.92	0.98	1.06
Doxycycline Hyclate	Others	50443	46907	52212	43952	44771	42956	0.99	0.89	0.99	1.00	1.03	1.02	0.96	1.02	1.06
Fludarabine Phosphate	DNA/RNA Synthesis	44720	48426	49832	42978	45891	46135	0.92	0.92	0.95	0.95	1.00	1.00	0.93	0.99	1.06
Methotrexate	DHFR	42835	42961	44754	41811	41621	39931	0.88	0.81	0.86	0.93	0.91	0.87	0.85	0.90	1.06
Enzastaurin (LY317615)	PKC	46343	50941	54811	46619	47944	49006	0.95	0.97	1.05	1.03	1.05	1.06	0.99	1.05	1.06
Nexturastat A	HDAC	48303	49991	48019	45969	45690	43345	0.94	0.98	0.91	1.03	1.00	0.97	0.94	1.00	1.06
empty		51810	55400	48164	45066	42373	41698	0.99	1.02	0.92	1.05	1.00	1.03	0.98	*1.04*	1.06
Deoxyarbutin	Others	55030	50033	50692	45219	46866	45244	1.08	0.95	0.96	1.03	1.08	1.07	1.00	1.06	1.06
OF-1	Epigenetic Reader Domain	46996	46369	47770	43776	41615	44845	0.92	0.90	0.91	0.98	0.91	1.00	0.91	0.96	1.06
Doxapram HCl	Others	48865	49157	49102	41722	40845	40503	0.94	0.91	0.94	0.97	0.97	1.00	0.93	*0.99*	1.06
Nystatin (Fungicidin)	Others	48198	52469	52984	48274	45951	43852	0.89	0.99	0.99	1.05	0.99	1.00	0.95	1.01	1.06
LDN-212854	TGF-beta/Smad	44585	50891	44842	40833	42593	40237	0.88	0.97	0.85	0.93	0.98	0.95	0.90	0.95	1.06
Omeprazole	Autophagy,Proton Pump	46568	48401	47668	43583	43975	47436	0.95	0.92	0.91	0.97	0.96	1.03	0.93	0.99	1.06
LY411575	Gamma-secretase	48868	49738	49514	43598	42528	39297	0.94	0.92	0.95	1.02	1.01	0.97	0.93	*0.99*	1.06
Myricetin	Others	45755	48658	46762	43999	45578	40469	0.87	0.95	0.88	0.97	0.99	0.91	0.90	0.96	1.06
Tenovin-6	p53,Sirtuin	44055	50573	48876	41887	42700	41987	0.87	0.96	0.93	0.96	0.98	0.99	0.92	0.98	1.06
PTC-209 HBr	Others	17958	18424	18599	16757	16968	17108	0.35	0.36	0.35	0.37	0.37	0.38	0.35	0.38	1.06
LY2811376	Beta Amyloid,BACE	51290	54226	53584	48307	47682	47122	0.95	1.02	1.00	1.05	1.02	1.07	0.99	1.05	1.06
Dovitinib (TKI258) Lacta	FGFR,VEGFR,c-Kit,PDGFR,FLT3	40531	35783	39001	34273	34918	37429	0.79	0.70	0.74	0.77	0.77	0.83	0.74	0.79	1.06
Silibinin	Others	50469	46060	47370	46704	40835	44907	0.96	0.90	0.90	1.03	0.89	1.01	0.92	0.98	1.06
STF-31	Others	26389	24039	26537	25305	21359	24472	0.51	0.47	0.50	0.57	0.47	0.55	0.50	0.53	1.06
RKI-1447	ROCK	44509	44529	44757	38580	39280	40298	0.87	0.85	0.85	0.88	0.90	0.95	0.86	0.91	1.06
Sulfadiazine	Others	50508	52224	48890	44839	46385	45245	0.93	0.98	0.91	0.98	1.00	1.03	0.94	1.00	1.06
Amfebutamone HCl	AChR,Dopamine Receptor	48038	45083	48279	45857	40816	43655	0.91	0.88	0.91	1.01	0.89	0.98	0.90	0.96	1.06
Probenecid	TRPV	49085	51172	51626	43596	44720	41598	0.94	0.94	0.99	1.02	1.06	1.02	0.96	*1.02*	1.06
TG100713	PI3K	41011	41619	42731	34723	36142	35566	0.79	0.77	0.82	0.81	0.86	0.88	0.79	*0.84*	1.07
Dinitolmide	Others	55135	55353	50863	46497	53025	43400	1.08	1.05	0.97	1.06	1.22	1.03	1.03	1.10	1.07
Furosemide	Others	55025	56926	56303	50950	50437	50381	1.02	1.07	1.05	1.11	1.08	1.15	1.04	1.11	1.07
Bethanechol chloride	AChR	43091	49701	48762	42723	46096	42112	0.82	0.97	0.92	0.94	1.00	0.94	0.90	0.96	1.07
Stattic	STAT	31914	36533	36269	30909	31701	30116	0.63	0.70	0.69	0.71	0.73	0.71	0.67	0.72	1.07
JNJ-26854165 (Serdem	E3 Ligase ,p53	44652	50548	48114	43672	46188	46342	0.91	0.96	0.92	0.97	1.01	1.00	0.93	0.99	1.07
Esomeprazole Sodium	ATPase	48561	46577	50404	49951	38896	45585	0.92	0.91	0.95	1.10	0.85	1.02	0.93	0.99	1.07
Morin Hydrate	Others	47340	41732	50082	43378	41112	44045	0.90	0.81	0.95	0.96	0.90	0.99	0.89	0.95	1.07
DMSO		46831	45770	50757	42682	45609	48221	0.96	0.87	0.97	0.95	1.00	1.05	0.93	1.00	1.07
Edaravone	Others	47731	48245	48249	45593	45148	46572	0.98	0.91	0.92	1.01	0.99	1.01	0.94	1.00	1.07
Artemether	Others	43591	44101	46646	41094	40979	42166	0.83	0.86	0.88	0.91	0.89	0.95	0.86	0.92	1.07
DMSO		42589	49374	49829	39843	42451	43291	0.84	0.94	0.95	0.91	0.98	1.02	0.91	0.97	1.07
Avasimibe	P450 (e.g. CYP17)	47475	46672	46615	43033	43213	43998	0.90	0.91	0.88	0.95	0.94	0.99	0.90	0.96	1.07
Didanosine	Reverse Transcriptase	57510	52366	55795	49180	48988	51442	1.06	0.98	1.04	1.07	1.05	1.17	1.03	1.10	1.07
Dimesna	Others	45598	50064	45871	46797	39685	48310	0.93	0.95	0.88	1.04	0.87	1.05	0.92	0.98	1.07
JSH-23	NF-κB	51067	49699	50392	45544	50001	45106	1.00	0.97	0.96	1.02	1.10	1.00	0.97	1.04	1.07
Liothyronine Sodium	Others	43804	50467	49348	40846	43835	42736	0.86	0.96	0.94	0.93	1.01	1.01	0.92	0.98	1.07
Ki8751	c-Kit,VEGFR,PDGFR	43644	43224	53277	42951	46059	44507	0.89	0.82	1.02	0.95	1.01	0.96	0.91	0.97	1.07
Anastrozole	Aromatase	47218	47313	46286	45697	45420	43182	0.97	0.90	0.89	1.01	0.99	0.94	0.92	0.98	1.07
UK 383367	Procollagen C Proteinase	52769	51309	47885	47099	46788	46969	1.00	1.00	0.91	1.04	1.02	1.05	0.97	1.04	1.07
Kaempferol	Others	48327	47562	50390	46282	42090	47082	0.92	0.93	0.95	1.02	0.92	1.06	0.93	1.00	1.07
Tivozanib (AV-951)	PDGFR,c-Kit,VEGFR	39854	42014	42807	36203	41483	41272	0.82	0.80	0.82	0.80	0.91	0.89	0.81	0.87	1.07
Dovitinib (TKI-258, CHIR	FLT3,VEGFR,FGFR,c-Kit,PDGFR	47301	46863	50523	44188	47599	46316	0.97	0.89	0.97	0.98	1.04	1.00	0.94	1.01	1.07
JNJ-7706621	Aurora Kinase,CDK	38133	44592	46567	40419	40692	42020	0.78	0.85	0.89	0.90	0.89	0.91	0.84	0.90	1.07
Nintedanib (BIBF 1120)	VEGFR,PDGFR,FGFR	44680	45710	46623	41940	44019	44836	0.91	0.87	0.89	0.93	0.96	0.97	0.89	0.95	1.07
Cladribine	DNA/RNA Synthesis	19151	20221	19388	19255	18523	18291	0.39	0.38	0.37	0.43	0.40	0.40	0.38	0.41	1.07
Aliskiren Hemifumarate	RAAS	50734	47739	51714	47686	41746	49814	0.96	0.93	0.98	1.05	0.91	1.12	0.96	1.03	1.07
Bazedoxifene Acetate	Estrogen/progestogen Receptor	51673	53459	50703	48978	43591	52049	0.98	1.04	0.96	1.08	0.95	1.17	1.00	1.07	1.07
Butoconazole nitrate	Others	49238	45565	52567	45696	44411	43545	0.91	0.86	0.98	1.00	0.95	0.99	0.91	0.98	1.07
ML347	TGF-beta/Smad	44403	46308	46668	42890	40506	38957	0.87	0.88	0.89	0.98	0.93	0.92	0.88	0.94	1.07
Alisertib (MLN8237)	Aurora Kinase	27674	29048	32424	26621	29487	29040	0.57	0.55	0.62	0.59	0.64	0.63	0.58	0.62	1.07
Gramine	Others	50929	46370	46709	45729	42666	45336	0.97	0.91	0.88	1.01	0.93	1.02	0.92	0.99	1.07
Famotidine	Histamine Receptor	54446	49610	53338	49456	48353	45069	1.01	0.93	0.99	1.08	1.04	1.03	0.98	1.05	1.07
SecinH3	hCyh,drosophila steppke,yGea2-S7	45579	45073	47540	44180	42182	42522	0.89	0.88	0.90	0.99	0.93	0.95	0.89	0.95	1.07
MPI-0479605	Kinesin	38877	39324	37492	32740	37309	37995	0.76	0.77	0.71	0.73	0.82	0.85	0.75	0.80	1.07

DMSO		49879	43194	48303	44011	44172	43161	0.95	0.84	0.91	0.97	0.96	0.97	**0.90**	**0.97**	1.07
MG149	Histone Acetyltransferase	49803	47785	47705	43795	43298	48589	0.97	0.93	0.90	0.98	0.95	1.08	**0.94**	**1.00**	1.07
CCT129202	Aurora Kinase	43641	50975	51102	45575	46153	47452	0.89	0.97	0.98	1.01	1.01	1.03	**0.95**	**1.02**	1.07
Zosuquidar (LY335979)	P-gp	44792	49102	49841	41397	49045	47132	0.92	0.93	0.95	0.92	1.07	1.02	**0.93**	**1.00**	1.07
DMSO		57108	53161	49364	49823	47171	48066	1.05	1.00	0.92	1.09	1.01	1.09	**0.99**	**1.06**	1.07
Pexmetinib (ARRY-614)	Tie-2,p38 MAPK	45291	41943	44354	41507	41764	39752	0.88	0.82	0.84	0.93	0.92	0.89	**0.85**	**0.91**	1.07
Sodium Danshensu	P450 (e.g. CYP17)	48385	44700	45597	46580	39766	42728	0.92	0.87	0.86	1.03	0.87	0.96	**0.89**	**0.95**	1.07
Ledipasvir (GS5885)	HCV Protease	44842	46759	43508	43052	38627	44651	0.87	0.91	0.82	0.96	0.85	0.99	**0.87**	**0.94**	1.07
DMSO		48694	50133	52631	44625	43756	46772	0.96	0.95	1.00	1.02	1.01	1.11	**0.97**	**1.04**	1.08
AZD1480	JAK	32038	30630	27624	25001	27245	31869	0.61	0.60	0.52	0.55	0.59	0.71	**0.58**	**0.62**	1.08
Meloxicam	COX	51201	47731	51479	46827	49230	41057	0.95	0.90	0.96	1.02	1.06	0.93	**0.93**	**1.00**	1.08
MI-773 (SAR405838)	Mdm2,p53	46202	44452	42600	41615	42420	40870	0.90	0.87	0.81	0.93	0.93	0.91	**0.86**	**0.92**	1.08
empty		45001	52854	47945	42151	41947	40502	0.86	0.97	0.92	0.98	0.99	1.00	**0.92**	***0.99***	1.08
Axitinib	c-Kit,PDGFR,VEGFR	33417	37232	36802	34807	34400	33938	0.68	0.71	0.71	0.77	0.75	0.74	**0.70**	**0.75**	1.08
Amuvatinib (MP-470)	FLT3,PDGFR,c-Kit	44331	50224	46359	44725	45895	44720	0.91	0.95	0.89	0.99	1.00	0.97	**0.92**	**0.99**	1.08
Daunorubicin HCl	Topoisomerase	4818	4357	5269	4378	3880	3849	0.09	0.08	0.10	0.10	0.09	0.09	**0.09**	***0.10***	1.08
empty		49944	49962	50094	44914	47792	47500	0.95	0.98	0.95	0.99	1.04	1.07	**0.96**	**1.03**	1.08
Dimethyl Fumarate	Others	48586	45169	48794	42269	46579	44446	0.92	0.88	0.92	0.93	1.02	1.00	**0.91**	**0.98**	1.08
TP-0903	TAM Receptor	39789	38923	41911	38276	37768	37237	0.78	0.76	0.79	0.86	0.83	0.83	**0.78**	**0.84**	1.08
ZM 336372	Raf	50740	52091	50224	44383	44076	42669	0.97	0.96	0.96	1.03	1.04	1.05	**0.97**	***1.04***	1.08
Mirtazapine	5-HT Receptor	51878	50769	51936	48317	47476	45538	0.96	0.95	0.97	1.05	1.02	1.04	**0.96**	**1.04**	1.08
BS-181 HCl	CDK	55207	49717	53829	51329	53017	41093	1.02	0.93	1.00	1.12	1.14	0.94	**0.99**	**1.06**	1.08
DMSO		51185	49658	48292	41213	43672	43558	0.98	0.92	0.93	0.96	1.03	1.07	**0.94**	***1.02***	1.08
VX-702	p38 MAPK	41456	43470	44717	38406	40685	37402	0.81	0.83	0.85	0.88	0.93	0.88	**0.83**	**0.90**	1.08
Sodium Phenylbutyrate	HDAC	50731	48530	52943	43227	47904	45704	1.00	0.92	1.01	0.99	1.10	1.08	**0.98**	**1.06**	1.08
empty		48424	48499	48563	44602	44496	47347	0.92	0.95	0.92	0.99	0.97	1.06	**0.93**	**1.01**	1.08
ABT-263 (Navitoclax)	Bcl-2	36944	40148	43190	37779	40928	37283	0.76	0.76	0.83	0.84	0.89	0.81	**0.78**	**0.85**	1.08
Simvastatin	HMG-CoA Reductase	45330	44397	38846	36793	37092	43774	0.84	0.83	0.72	0.80	0.80	1.00	**0.80**	**0.87**	1.08
Benserazide HCl	Others	50851	50565	47225	48646	48263	42872	0.97	0.99	0.89	1.08	1.05	0.96	**0.95**	**1.03**	1.08
Toltrazuril	Others	48387	54763	48694	45143	41463	41559	0.93	1.01	0.93	1.05	0.98	1.02	**0.96**	***1.04***	1.08
Tolperisone HCl	Sodium Channel	43584	52442	50293	45427	41722	44525	0.86	1.00	0.96	1.04	0.96	1.05	**0.94**	**1.02**	1.08
FRAX597	PAK	50762	48123	46725	42954	43223	45074	1.00	0.92	0.89	0.98	0.99	1.07	**0.93**	**1.01**	1.09
Azilsartan	RAAS	46093	48441	48116	40389	39889	41151	0.88	0.89	0.92	0.94	0.94	1.01	**0.90**	***0.98***	1.09
Cabozantinib (XL184, BI	Tie-2,TAM Receptor,FLT3,VEGFR,c-Met,c-K	39711	45373	43534	42230	41429	40697	0.81	0.86	0.83	0.94	0.91	0.88	**0.84**	**0.91**	1.09
(+)-Usniacin	Others	51264	49376	51750	50239	47877	45363	0.97	0.96	0.98	1.11	1.04	1.02	**0.97**	**1.06**	1.09
NU6027	CDK	44926	48767	45430	40914	43965	40750	0.88	0.93	0.86	0.93	1.01	0.96	**0.89**	**0.97**	1.09
OTX015	Epigenetic Reader Domain	37914	36724	34839	34747	33213	35628	0.74	0.72	0.66	0.78	0.73	0.79	**0.71**	**0.77**	1.09
Cerdulatinib (PRT06207	JAK	40902	41928	41400	38930	39469	39192	0.80	0.82	0.78	0.87	0.87	0.87	**0.80**	**0.87**	1.09
CEP-18770 (Delanzomit	Proteasome	888	670	814	757	805	747	0.02	0.01	0.02	0.02	0.02	0.02	**0.02**	**0.02**	1.09
SAR245409 (XL765)	PI3K,mTOR	54676	45916	48534	46568	45410	45250	1.01	0.86	0.90	1.01	0.98	1.03	**0.93**	**1.01**	1.09
PHA-767491	CDK	38160	39642	42827	36000	37344	33212	0.73	0.73	0.82	0.84	0.88	0.82	**0.76**	***0.83***	1.09
Pyridostigmine Bromid	AChR	54568	50636	48428	46563	48632	46322	1.01	0.95	0.90	1.01	1.05	1.05	**0.95**	**1.04**	1.09
RVX-208	Epigenetic Reader Domain	50890	51524	49264	44100	49191	43944	1.00	0.98	0.94	1.01	1.13	1.04	**0.97**	**1.06**	1.09
MNS (3,4-Methylenedic	p97	43778	47287	49165	40738	43524	42577	0.86	0.90	0.94	0.93	1.00	1.01	**0.90**	**0.98**	1.09
Z-FA-FMK	Cysteine Protease	47559	48270	48513	45530	47362	44015	0.93	0.94	0.92	1.02	1.04	0.98	**0.93**	**1.01**	1.09
Rupatadine Fumarate	Histamine Receptor	47544	46624	44266	41257	129	38302	0.91	0.86	0.85	0.96	0.00	0.94	**0.87**	***0.95***	1.09
Chloroambucil	DNA/RNA Synthesis	41860	46266	48897	41933	37050	45016	0.82	0.88	0.93	0.96	0.85	1.06	**0.88**	**0.96**	1.09
Aloperine	Others	46970	38248	45950	40171	39676	43991	0.89	0.75	0.87	0.89	0.86	0.99	**0.84**	**0.91**	1.09
FIIN-2	FGFR	41548	37682	44742	39906	39556	38168	0.81	0.74	0.85	0.89	0.87	0.85	**0.80**	**0.87**	1.09
SRT1720	Sirtuin	39031	42662	42599	40849	39052	40921	0.80	0.81	0.82	0.91	0.85	0.89	**0.81**	**0.88**	1.09
empty		45223	42617	49189	43154	45081	41894	0.88	0.83	0.93	0.97	0.99	0.93	**0.88**	**0.96**	1.09
MCB-613	Others	30142	28205	34025	28241	31231	28346	0.59	0.55	0.65	0.63	0.69	0.63	**0.59**	**0.65**	1.09
Dropropizine	Others	47469	53051	47210	43765	44145	46183	0.93	1.01	0.90	1.00	1.01	1.09	**0.95**	**1.04**	1.09
TWS119	GSK-3	37939	41294	39162	33928	38949	36751	0.70	0.78	0.73	0.74	0.84	0.84	**0.74**	**0.80**	1.09
Atorvastatin Calcium	HMG-CoA Reductase	52251	53073	48991	46147	46242	50316	0.96	1.00	0.91	1.01	0.99	1.15	**0.96**	**1.05**	1.09
L-Thyroxine	Others	47522	49691	48931	46600	45627	46590	0.90	0.97	0.93	1.03	0.99	1.04	**0.93**	**1.02**	1.10
Loperamide HCl	Autophagy,Opioid Receptor	45275	42175	46296	43715	41151	42132	0.86	0.82	0.88	0.97	0.90	0.94	**0.85**	**0.94**	1.10
Cepharanthine	Others	43579	44105	48228	40915	42352	40539	0.86	0.84	0.92	0.93	0.97	0.96	**0.87**	**0.96**	1.10
AZD4547	FGFR	43450	41119	41802	39594	36521	33586	0.83	0.76	0.80	0.92	0.87	0.83	**0.80**	***0.88***	1.10
JTC-801	Opioid Receptor	48880	46071	48615	42351	42721	40728	0.94	0.85	0.93	0.99	1.01	1.00	**0.91**	***1.00***	1.10
PF-4708671	S6 Kinase	49523	49526	51195	45846	45540	51607	0.94	0.97	0.97	1.01	0.99	1.16	**0.96**	**1.05**	1.10
Nifuroxazide	STAT	45559	48572	43721	44440	41955	39655	0.89	0.92	0.83	1.01	0.96	0.94	**0.88**	**0.97**	1.10
Orlistat	Others	52652	48735	52478	46399	47723	49032	0.97	0.92	0.98	1.01	1.03	1.12	**0.95**	**1.05**	1.10
MS436	Epigenetic Reader Domain	44770	46079	46660	43686	40520	41581	0.88	0.88	0.89	1.00	0.93	0.98	**0.88**	**0.97**	1.10
empty		54785	51033	45809	44948	47699	48583	1.01	0.96	0.85	0.98	1.02	1.11	**0.94**	**1.04**	1.10
PA-824	Others	41111	49623	51658	46902	46805	45845	0.84	0.94	0.99	1.04	1.02	0.99	**0.92**	**1.02**	1.10
DMSO		42040	45101	47431	41584	44834	45774	0.86	0.86	0.91	0.92	0.98	0.99	**0.87**	**0.96**	1.10
Buspirone HCl	5-HT Receptor	46594	48504	48317	42051	45742	43671	0.92	0.92	0.92	0.96	1.05	1.03	**0.92**	**1.01**	1.10

Zaltoprofen	COX	46451	48930	43553	43298	38371	37611	0.89	0.90	0.84	1.01	0.91	0.93	0.88	0.97	1.10
SU9516	CDK	47773	50612	46458	46058	44931	48316	0.93	0.99	0.88	1.03	0.99	1.08	0.93	1.03	1.11
PH-797804	p38 MAPK	47999	47677	47247	41331	41902	41835	0.92	0.88	0.91	0.96	0.99	1.03	0.90	1.00	1.11
Azilsartan Medoxomil	RAAS	48166	51085	51104	45112	41401	42449	0.92	0.94	0.98	1.05	0.98	1.05	0.95	1.05	1.11
MK-2461	FGFR,PDGFR,c-Met	40036	41506	37707	35731	34572	33749	0.77	0.77	0.72	0.83	0.82	0.83	0.75	0.83	1.11
AZ 960	JAK	17514	21142	19284	18620	18344	18745	0.33	0.41	0.36	0.41	0.40	0.42	0.37	0.41	1.11
(S)-10-Hydroxycamptot	Topoisomerase	4883	4624	4617	4470	4735	4371	0.09	0.09	0.09	0.10	0.10	0.10	0.09	0.10	1.11
Dyngo-4a	Dynamin	42294	40677	47017	40924	37685	41125	0.83	0.77	0.89	0.93	0.87	0.97	0.83	0.92	1.11
PF-573228	FAK	48041	47149	49506	47171	45027	43718	0.89	0.89	0.92	1.03	0.97	1.00	0.90	1.00	1.11
RAF265 (CHIR-265)	VEGFR,Raf	47280	42419	43429	43651	40301	44027	0.90	0.83	0.82	0.96	0.88	0.99	0.85	0.94	1.11
Peficitinib (ASP015K, JN	JAK	41690	42155	45425	40333	40683	44083	0.81	0.82	0.86	0.90	0.89	0.98	0.83	0.93	1.11
DMSO		49482	47284	49751	46415	50088	45058	0.94	0.92	0.94	1.03	1.09	1.01	0.94	1.04	1.11
Andarine	Androgen Receptor	42209	49664	45215	44396	44721	47034	0.86	0.94	0.87	0.98	0.98	1.02	0.89	0.99	1.12
Erythromycin	Others	49506	54267	52931	47095	51749	49198	0.91	1.02	0.99	1.03	1.11	1.12	0.97	1.09	1.12
Indole-3-carbinol	Others	42422	46103	48337	44795	40066	47621	0.81	0.90	0.91	0.99	0.87	1.07	0.87	0.98	1.12
BAY 11-7082	E2 conjugating,IκB/IKK	41676	40298	37618	36161	37248	34311	0.80	0.74	0.72	0.84	0.88	0.84	0.75	0.84	1.12
MG-101 (ALLN)	Cysteine Protease	30349	23639	21432	24070	25177	24298	0.59	0.46	0.41	0.54	0.55	0.54	0.49	0.54	1.12
TAI-1	Microtubule Associated	42603	38424	37545	44509	34884	36054	0.83	0.75	0.71	1.00	0.77	0.80	0.76	0.86	1.12
Nilotinib (AMN-107)	Bcr-Abl	44810	51890	49291	47400	48326	49977	0.92	0.98	0.94	1.05	1.06	1.08	0.95	1.06	1.12
Monobenzone	Others	51338	49247	51641	48859	47469	48212	0.95	0.93	0.96	1.06	1.02	1.10	0.94	1.06	1.12
CUDC-101	EGFR,HER2,HDAC	44356	43191	45368	45092	43667	44318	0.91	0.82	0.87	1.00	0.95	0.96	0.87	0.97	1.12
Chromocarb	Others	46131	47268	49926	45395	45683	42943	0.91	0.90	0.95	1.04	1.05	1.02	0.92	1.03	1.13
Tenovin-1	E3 Ligase ,p53	45848	44258	45953	44489	46008	42816	0.89	0.86	0.87	0.99	1.01	0.95	0.88	0.99	1.13
Carbamazepine	Sodium Channel,Autophagy	47221	48791	46288	45762	43748	46182	0.87	0.92	0.86	1.00	0.94	1.05	0.88	1.00	1.13
STF-118804	NAMPT	18693	19149	19271	19015	17046	17497	0.37	0.36	0.37	0.43	0.39	0.41	0.37	0.41	1.13
Puerarin	Others	48217	44299	47810	47081	46756	43424	0.92	0.87	0.90	1.04	1.02	0.97	0.90	1.01	1.13
DMSO		48302	55600	55053	48516	45602	46026	0.93	1.02	1.06	1.13	1.08	1.13	1.00	1.13	1.13
Mitoxantrone HCl	Topoisomerase	9001	9837	8262	7747	9440	9391	0.17	0.19	0.16	0.17	0.21	0.21	0.17	0.20	1.13
Topotecan HCl	Topoisomerase	3909	4970	4507	4748	4308	4406	0.08	0.09	0.09	0.11	0.09	0.10	0.09	0.10	1.13
VR23	Proteasome	40544	34427	36842	39380	29812	40891	0.79	0.67	0.70	0.88	0.66	0.91	0.72	0.82	1.13
Dioscin	Others	33185	32435	35945	36245	32739	30813	0.63	0.63	0.68	0.80	0.71	0.69	0.65	0.74	1.13
KW-2449	Bcr-Abl,Aurora Kinase,FLT3	44292	45143	48172	42626	43367	49552	0.84	0.88	0.91	0.94	0.95	1.11	0.88	1.00	1.14
PD128907 HCl	Dopamine Receptor	48131	49909	48701	47804	44522	52429	0.92	0.97	0.92	1.06	0.97	1.18	0.94	1.07	1.14
AZD5438	CDK	47059	46158	44557	47068	44363	44624	0.89	0.90	0.84	1.04	0.97	1.00	0.88	1.00	1.14
Lenvatinib (E7080)	VEGFR	40579	46008	43214	46427	43228	42165	0.83	0.87	0.83	1.03	0.95	0.91	0.84	0.96	1.14
PD173074	VEGFR,FGFR	39365	37951	39195	41562	38797	38462	0.81	0.72	0.75	0.92	0.85	0.83	0.76	0.87	1.14
4SC-202	HDAC	25987	26561	24369	36951	22502	17215	0.51	0.52	0.46	0.83	0.49	0.38	0.50	0.57	1.15
Lonafarnib	Transferase	39302	36575	40865	36807	37104	33879	0.75	0.67	0.78	0.86	0.88	0.83	0.74	0.85	1.15
MI-2 (MALT1 inhibitor)	Others	33309	39565	35024	30600	42613	35246	0.65	0.77	0.66	0.68	0.94	0.79	0.70	0.80	1.15
Irinotecan	Topoisomerase	15456	15500	16010	16000	15334	17088	0.32	0.29	0.31	0.35	0.34	0.37	0.31	0.35	1.16
BMH-21	DNA/RNA Synthesis	47830	26346	46303	41411	41086	38665	0.93	0.51	0.88	0.93	0.90	0.86	0.77	0.90	1.16
Tipifarnib	Transferase	37473	40929	43127	44591	46660	34008	0.77	0.78	0.83	0.99	1.02	0.74	0.79	0.92	1.16
CYC116	VEGFR,Aurora Kinase	41911	43726	36869	43769	48906	34780	0.86	0.83	0.71	0.97	1.07	0.75	0.80	0.93	1.17
BX-912	PDK-1	34681	39852	35191	47515	34684	33005	0.71	0.76	0.67	1.05	0.76	0.72	0.71	0.84	1.18
SB216763	GSK-3	55126	56002	58744	85178	48382	45374	1.13	1.06	1.13	1.89	1.06	0.98	1.11	1.31	1.18
BMN 673	PARP	24718	25977	27304	24639	27823	24822	0.49	0.49	0.52	0.56	0.64	0.59	0.50	0.60	1.19
PHA-680632	Aurora Kinase	34006	35540	36215	48996	32447	31873	0.70	0.67	0.69	1.09	0.71	0.69	0.69	0.83	1.20
Fluvastatin Sodium	HMG-CoA Reductase	35254	35439	37034	39324	37403	33991	0.65	0.67	0.69	0.86	0.80	0.77	0.67	0.81	1.21
GSK256066	PDE	52035	49776	48100	40489	81244	39708	0.99	0.97	0.91	0.90	1.77	0.89	0.96	1.19	1.24
MG-132	Proteasome	1234	1098	1024	1288	1259	1129	0.02	0.02	0.02	0.03	0.03	0.03	0.02	0.03	1.26
Plinabulin (NPI-2358)	VDA	52233	54292	14469	45535	46642	45610	1.07	1.03	0.28	1.01	1.02	0.99	0.79	1.01	1.27
Oprozomib (ONX 0912)	Proteasome	1002	880	944	1002	1076	935	0.02	0.02	0.02	0.02	0.02	0.02	0.02	0.02	1.28
Amitriptyline HCl	5-HT Receptor	51947	48033	47996	39449	38961	62745	1.00	0.89	0.92	0.92	0.92	1.55	0.93	1.23	1.32
Reversine	Adenosine Receptor,Aurora Kinase	39299	35148	37165	45682	42641	40639	0.77	0.69	0.70	1.02	0.94	0.91	0.72	0.95	1.33
AT13387	HSP (e.g. HSP90)	17924	21764	22585	41243	17186	18392	0.37	0.41	0.43	0.91	0.38	0.40	0.40	0.56	1.39
Belinostat (PXD101)	HDAC	18328	21488	20613	49572	14034	15875	0.38	0.41	0.39	1.10	0.31	0.34	0.39	0.58	1.49
Aurora A Inhibitor I	Aurora Kinase	27412	26894	28051	42807	45835	21311	0.56	0.51	0.54	0.95	1.00	0.46	0.54	0.80	1.50
Volasertib (BI 6727)	PLK	15932	18836	16516	46354	11662	11275	0.30	0.37	0.31	1.02	0.25	0.25	0.33	0.51	1.56
AUY922 (NVP-AUY922)	HSP (e.g. HSP90)	17439	17768	17407	43644	16600	14769	0.36	0.34	0.33	0.97	0.36	0.32	0.34	0.55	1.61
Crystal Violet	Others	53867	19103	15139	34301	43961	41225	0.99	0.36	0.28	0.75	0.94	0.94	0.55	0.88	1.61
NSC697923	E2 conjugating	7272	10926	9714	11949	15655	11470	0.14	0.21	0.18	0.27	0.36	0.27	0.18	0.30	1.69
BIIB021	HSP (e.g. HSP90)	17839	20729	23176	42772	42919	16757	0.37	0.39	0.44	0.95	0.94	0.36	0.40	0.75	1.87
Gemcitabine	DNA/RNA Synthesis,Autophagy	14172	13268	15607	47045	12755	11627	0.26	0.25	0.29	1.03	0.27	0.26	0.27	0.52	1.95
Epothilone A	Microtubule Associated	12071	13768	11726	48075	9921	9218	0.25	0.26	0.22	1.07	0.22	0.20	0.24	0.49	2.02
Ispinesib (SB-715992)	Kinesin	17493	17296	19480	44449	49146	12150	0.36	0.33	0.37	0.99	1.07	0.26	0.35	0.77	2.19
Gambogic Acid	Caspase,Bcl-2	4950	3761	1898	3472	7727	9695	0.09	0.07	0.04	0.08	0.17	0.22	0.07	0.15	2.27
Obatoclax Mesylate (G)	Autophagy,Bcl-2	13473	13275	12110	42081	51115	11984	0.28	0.25	0.23	0.93	1.12	0.26	0.25	0.77	3.04
Camptothecin	Topoisomerase	8248	9477	10295	46156	42899	8347	0.17	0.18	0.20	1.02	0.94	0.18	0.18	0.71	3.92

Celastrol	Proteasome	5188	4285	4049	46333	5777	4381	0.11	0.08	0.08	1.03	0.13	0.09	**0.09**	**0.42**	4.71
LAQ824 (Dacinostat)	HDAC	7377	6953	7369	42862	44840	3704	0.15	0.13	0.14	0.95	0.98	0.08	**0.14**	**0.67**	4.74
Elesclomol (STA-4783)	HSP (e.g. HSP90)	2624	5140	2709	46767	1817	2128	0.05	0.10	0.05	1.04	0.04	0.05	**0.07**	**0.37**	5.53

Figure 2 and 3 uncropped western blot

Fig. 2D

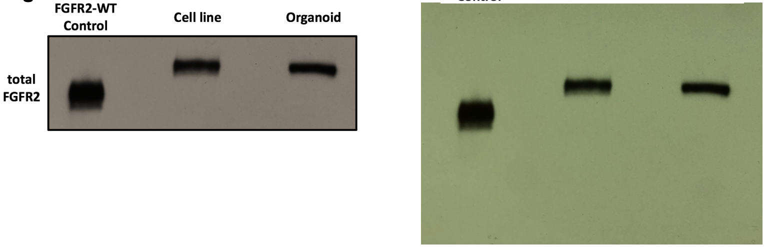

Fig. 3A

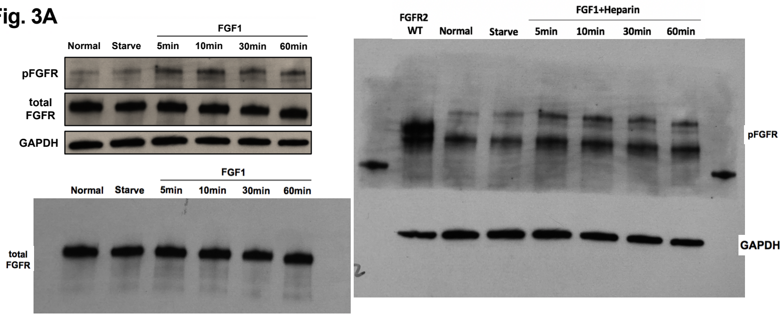

Fig. 3B

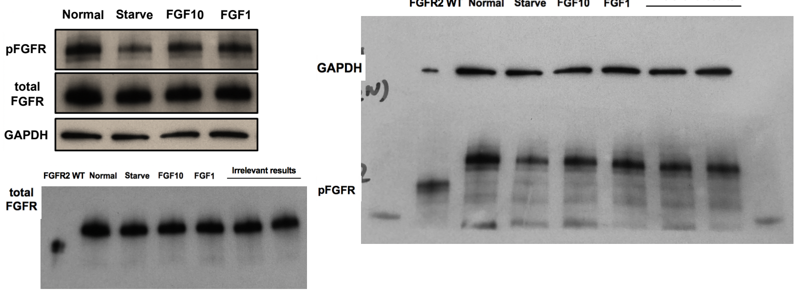

Fig. 3C

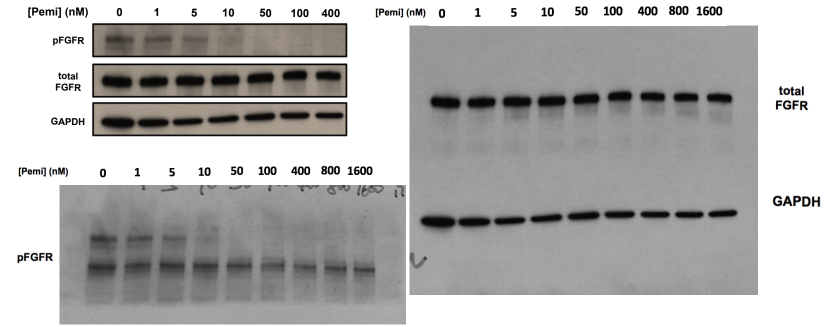

Fig. 3D

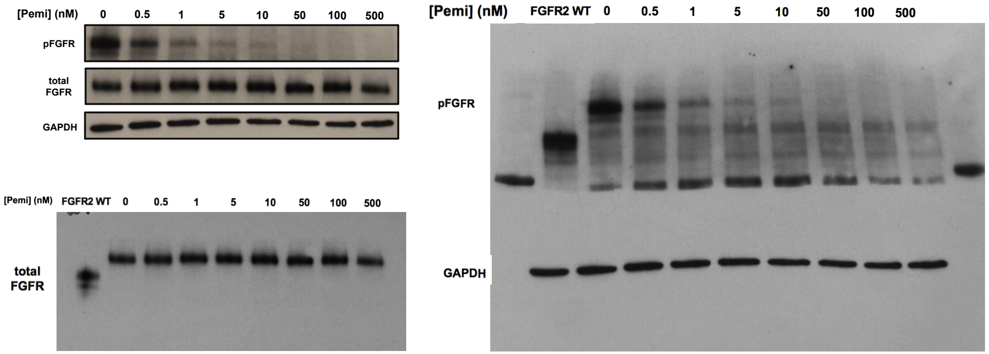

Figure 7 uncropped western blot

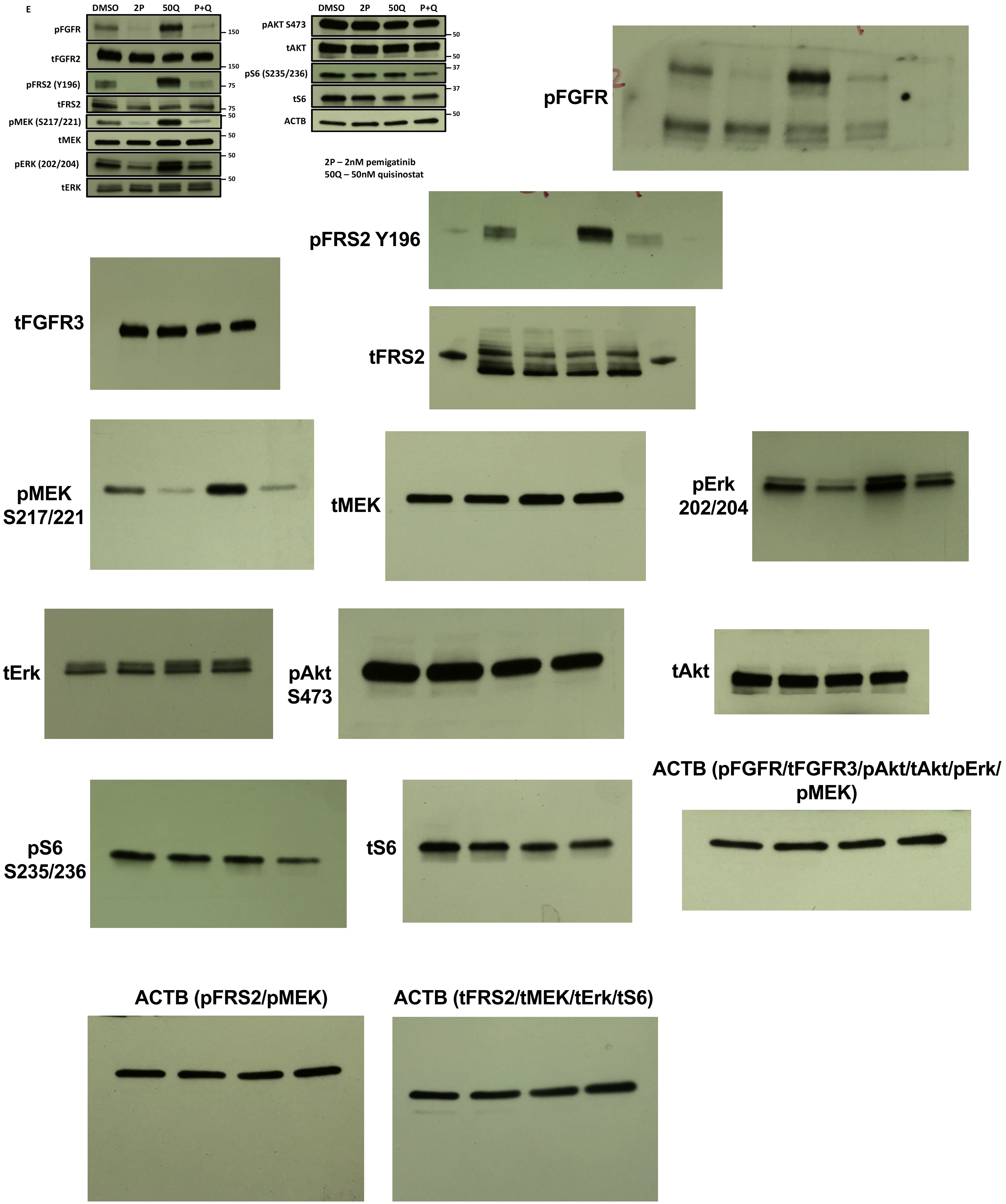

Supplement: Supplementary file 1 — Supplementary Material [file 41698_2022_320_MOESM1_ESM.pdf]
