# Supplementary material for: Leveraging patient derived models of FGFR2 fusion positive intrahepatic cholangiocarcinoma to identify synergistic therapies
Source: NPJ Precis Oncol. 2022 Oct 23;6:75. doi: 10.1038/s41698-022-00320-5 (PMC9588766; doi:10.1038/s41698-022-00320-5)
Supplement: Supplementary file 2 — REPORTING SUMMARY [file 41698_2022_320_MOESM2_ESM.pdf]

nature portfolio

Corresponding author(s): Michael Lidsky, Kris Wood

Last updated by author(s): Aug 19, 2022

Reporting Summary

Nature Portfolio wishes to improve the reproducibility of the work that we publish. This form provides structure for consistency and transparency in reporting. For further information on Nature Portfolio policies, see our Editorial Policies and the Editorial Policy Checklist.

Statistics

For all statistical analyses, confirm that the following items are present in the figure legend, table legend, main text, or Methods section.

n/a	Confirmed	
☐	☒	The exact sample size (*n*) for each experimental group/condition, given as a discrete number and unit of measurement
☐	☒	A statement on whether measurements were taken from distinct samples or whether the same sample was measured repeatedly
☐	☒	The statistical test(s) used AND whether they are one- or two-sided<br>*Only common tests should be described solely by name; describe more complex techniques in the Methods section.*
☒	☐	A description of all covariates tested
☐	☒	A description of any assumptions or corrections, such as tests of normality and adjustment for multiple comparisons
☐	☒	A full description of the statistical parameters including central tendency (e.g. means) or other basic estimates (e.g. regression coefficient) AND variation (e.g. standard deviation) or associated estimates of uncertainty (e.g. confidence intervals)
☐	☒	For null hypothesis testing, the test statistic (e.g. *F*, *t*, *r*) with confidence intervals, effect sizes, degrees of freedom and *P* value noted<br>*Give P values as exact values whenever suitable.*
☒	☐	For Bayesian analysis, information on the choice of priors and Markov chain Monte Carlo settings
☒	☐	For hierarchical and complex designs, identification of the appropriate level for tests and full reporting of outcomes
☐	☒	Estimates of effect sizes (e.g. Cohen's *d*, Pearson's *r*), indicating how they were calculated

Our web collection on statistics for biologists contains articles on many of the points above.

Software and code

Policy information about availability of computer code

Data collection

The code to analyze the PDC DNA-Seq and RNA-Seq libraries (for inferring structural variants and gene fusion events) and Sanger sequencing data is available through a source code repository (https://gitlab.oit.duke.edu/dcibioinformatics/pubs/lidsky-fgfr2-fusion-paper). The repository also provides the container recipes to reproduce the computational environment used for the analyses. Complete listing of the programs, including software version, used for these analyses is provided in the methods section of the paper. The somatic SNVs analysis was conducted by the Genomics Operation Core at Memorial Sloan Kettering Cancer Center. The reference providing the reference for the latter analysis has been provided in the paper.

Data analysis

The quality of the DNA-Seq and RNA-seq data from the PDC was evaluated using FastQC (https://www.bioinformatics.babraham.ac.uk/projects/fastqc/) version 0.11.9. The DNA sequencing libraries were aligned to the hg38 reference sequence obtained from the GATK v0 bundle using the BWA-MEM algorithm version 0.17.7. The resulting reads were sorted by coordinate and deduplicated using picard tools (https://broadinstitute.github.io/picard/) version 2.26.10. The base quality scores of resulting bam file were recalibrated using the BaseRecalibrator tool from GATK version 4.1.4.1. For the latter step, SNP and indel annotation files from the GATK hg38 v0 bundle were used. DELLY was used to identify structural variants in DNA-Seq data from these bam files. Specifically, structural variants were identified from the recalibrated bam files with delly2, version 0.9.1, and the BCF files generated by delly2 were annotated with sansa, version 0.0.8. Somatic single-nucleotide variants (SNVs) from the DNA-Seq data using b37 genomic coordinates were generated by the Marie-Josée and Henry R. Kravis Center for Molecular Oncology.
STAR-Fusion48,49 version 1.10.1 was used to identify gene fusions and predict coding sequences of fusion transcripts. The inferred versions were validated in silico using FusionInspector version 2.6.0. The plug and play version of the Trinity Cancer Transcriptome Analysis Toolkit (CTAT) resource bundle built against GENCODE version 37 (March 01, 2021) annotation was used.
Primers for validating the genomic breakpoints were designed by first predicting the sequences of the translocated chromosomes flanking the breakpoints with custom R code using the Rsamtools package. These sequences were then used to design primers with Primer3Plus and

Primer-BLAST. Breakpoints were confirmed by assembling Sanger sequencing results on the predicted sequences using SeqMan UltraTM (DNASTAR, Inc. Madison, Wisconsin USA). Chromatograms of breakpoints were generated with custom R code using the sangerseqR package.

For manuscripts utilizing custom algorithms or software that are central to the research but not yet described in published literature, software must be made available to editors and reviewers. We strongly encourage code deposition in a community repository (e.g. GitHub). See the Nature Portfolio guidelines for submitting code & software for further information.

Data

Policy information about availability of data

All manuscripts must include a data availability statement. This statement should provide the following information, where applicable:
- Accession codes, unique identifiers, or web links for publicly available datasets
- A description of any restrictions on data availability
- For clinical datasets or third party data, please ensure that the statement adheres to our policy

The PDC DNA-Seq and RNA-Seq libraries and Sanger sequencing data are available through BioProject accession number PRJNA854934.

Human research participants

Policy information about studies involving human research participants and Sex and Gender in Research.

Reporting on sex and gender	N/A
Population characteristics	N/A
Recruitment	N/A
Ethics oversight	N/A

Note that full information on the approval of the study protocol must also be provided in the manuscript.

Field-specific reporting

Please select the one below that is the best fit for your research. If you are not sure, read the appropriate sections before making your selection.

☒ Life sciences ☐ Behavioural & social sciences ☐ Ecological, evolutionary & environmental sciences

For a reference copy of the document with all sections, see nature.com/documents/nr-reporting-summary-flat.pdf

Life sciences study design

All studies must disclose on these points even when the disclosure is negative.

Sample size	For in vivo experiments comparing FGFR2 inhibition vs control (vehicle), a power analysis using linear mixed models and implemented via powerlmm in R56 indicated a sample size of 8 mice would be adequate to detect a 50% reduction in tumor volume in the treatment group relative to control (power = 0.80, alpha = 0.05). For the experiments evaluating a combined effect of FGFR2 inhibition and HDAC inhibition, 12 mice per arm were required to detect a 1/4 reduction in rate of increase in tumor volume in the combination therapy relative to monotherapy (power = 0.80, alpha = 0.05).
Data exclusions	No data were excluded. The analyses described were intention-to-treat.
Replication	All in vitro experiments were performed in replicate (triplicate), including technical and biological replicates where appropriate.
Randomization	For in vivo experiments, xenografts were measured 3x/week until tumor volume reached ~125 mm3, at which time mice were randomized to treatment arms.
Blinding	Blinding was not performed because of the design of the in vivo experiments. Mice required treatment 5 days per week with measurements of tumors up to 3x/week. We did minimize inter-observer bias by having all aspects of these experiments executed by a single author (AL).

Reporting for specific materials, systems and methods

We require information from authors about some types of materials, experimental systems and methods used in many studies. Here, indicate whether each material, system or method listed is relevant to your study. If you are not sure if a list item applies to your research, read the appropriate section before selecting a response.

Materials & experimental systems

n/a	Involved in the study
☐	☒ Antibodies
☐	☒ Eukaryotic cell lines
☒	☐ Palaeontology and archaeology
☐	☒ Animals and other organisms
☒	☐ Clinical data
☒	☐ Dual use research of concern

Methods

n/a	Involved in the study
☒	☐ ChIP-seq
☐	☒ Flow cytometry
☒	☐ MRI-based neuroimaging

Antibodies

Antibodies used

IHC: monoclonal mouse anti-human antibodies to CK7 (Clone OV-TL 12/30) and CK19 (Clone RCK108) from Dako (Santa Clara, CA), and CK20 (Ks20.8, Cat# PA0022), CA19-9 (C241:5:1:4, Cat# PA0424), and CDX2 (EP25, Cat# PA0375) from Leica Biosystems (Newcastle, United Kingdom).

WB (purchased from CST, unless stated otherwise): phospho-FGFR (#3476), FGFR2 (#23328), GAPDH (#3683), phospho-FRS2 Y196 (#3864), FRS2 (R&D, #MAB4069), phospho-MEK S217/221 (#9154), MEK (#4694), phospho-ERK T202/Y204 (#4370), ERK (#4695), phospho-AKT S473 (#4060), AKT(#4691), phospho-S6 S235/236 (#4858), S6 (#2317)

Validation

IHC antibodies were validated by the Duke Histology Core of the Department of Pathology. These antibodies were either used clinically or validated by the vendor from which they were purchased.

WB antibodies: All antibodies were commercially purchased with validation data shared by the vendor.

Eukaryotic cell lines

Policy information about cell lines and Sex and Gender in Research

Cell line source(s)

DUC18828 is a patient-derived cell line, as described in the manuscript. ICC cell lines RBE and SSP-25 were obtained from Lawrence Kwong, PhD, at MD Anderson Cancer Center. RT4 cells were generously gifted by Dr. Darryl Martin's lab (Yale University, New Haven, CT). SUM185PE (Catalog No: 0003008) was purchased from BioIVT (Wesbury, NY, USA).

Authentication

STR validation was performed for DUC18828, RBE, and SSP-25.

Mycoplasma contamination

All cell lines were tested for mycoplasma and confirmed to be negative for contamination.

Commonly misidentified lines
(See ICLAC register)

N/A

Animals and other research organisms

Policy information about studies involving animals; ARRIVE guidelines recommended for reporting animal research, and Sex and Gender in Research

Laboratory animals

8 week old female NSG mice (Jackson Laboratories)

Wild animals

N/A

Reporting on sex

Data are not relevant to the sex of the mice used.

Field-collected samples

N/A

Ethics oversight

Duke IACUC

Note that full information on the approval of the study protocol must also be provided in the manuscript.

Flow Cytometry

Plots

Confirm that:

☒ The axis labels state the marker and fluorochrome used (e.g. CD4-FITC).

☒ The axis scales are clearly visible. Include numbers along axes only for bottom left plot of group (a 'group' is an analysis of identical markers).

☒ All plots are contour plots with outliers or pseudocolor plots.

☒ A numerical value for number of cells or percentage (with statistics) is provided.

Methodology

Sample preparation	Patient-derived DUC18828 cells were seeded in 10-cm dishes at 1:10 ratio for 2 days after reaching ~70-80% confluence. Media was then refreshed to include pemigatinib and/or quisinostat at desired concentrations. Cells are treated for ~48 hours before analysis. For Ki-67 analyses, cells were harvested, pelleted, washed, and fixed in 70% ethanol for 30 minutes at 4C. After fixation, cells were washed twice with flow buffer (1x DPBS, +0.5% BSA) and stained by AlexaFluor 488 conjugated Ki-67 antibody (1:50, CST, #11882) for 30 minutes. Then the cells were washed twice and finally resuspended in flow buffer with 100ug/ml RNase A (QIAGEN, #1031301) and 50 ug/ml propidium iodide (PI, Sigma-Aldrich, P4170). For apoptosis analyses, cell culture media was collected in 15 mL tubes and cells were dissociated by 0.25% trypsin. Both the cell culture media and dissociated cells were pelleted at 2000 rpm at room temperature for 5 minutes. Then the pellets were washed twice in ice-cold 1x DPBS supplied with 1% HI-FBS. Cells were stained with Annexin V-FITC early apoptosis detection kit (CST, Cat# 6592) according to manufacturer's protocol.<br>Organoids were split 1:5-1:10 in 24-well plates and cultured in ICC assay media for 3 days after reaching near-confluence. Media was then refreshed to include pemigatinib and/or quisinostat at desired concentration, and the organoids were treated for ~72 hours before analysis. To harvest organoids, matrigel was first disrupted in 1 mL/50 uL ice-cold 1x DPBS and transferred to a 15 mL tube pre-filled with 10 mL ice-cold 1x DPBS, and the organoid was pelleted at 2000 rpm for 5 minutes at 4C. Patient-derived DUC18828 organoids were then digested into single cells by TrypLE Express at 37C for 10 minutes. Digested organoids were washed twice in ice-cold flow buffer and samples were prepared as described above for Ki-67 and apoptosis analyses.
Instrument	Flow cytometry data were collected by BD LSRII system.
Software	Flow cytometry data were collected by BD LSRII system and data were analyzed by FlowJo.
Cell population abundance	Cell population abundance is designated as shown in the figure and results section.
Gating strategy	For apoptosis analysis, cells were first gated by FSC-SSC for living single cells and cell debris, and then gated by AnnexinV-FITC and propidium iodide signal, as examplified in the supplementary figure. The positive and negative boundaries are defined by no stain and single stain controls.<br><br>For Ki-67 analysis, cells were first gated by FSC-SSC for single cells, and then gated by propidium iodide positive population, and then gated by Ki-67-AF488 signaling, as examplified in the supplementary figure. The positive and negative boundaries are defined by no stain and single stain controls.

☒ Tick this box to confirm that a figure exemplifying the gating strategy is provided in the Supplementary Information.
